# Supplementary material for: Saccharin disrupts bacterial cell envelope stability and interferes with DNA replication dynamics
Source: EMBO Mol Med. 2025 Apr 1;17(5):993–1017. doi: 10.1038/s44321-025-00219-1 (PMC12081710; doi:10.1038/s44321-025-00219-1)
Supplement: Supplementary file 1 — Appendix [file 44321_2025_219_MOESM1_ESM.pdf]

Saccharin disrupts bacterial cell envelope stability and interferes with DNA replication dynamics.

Rubén de Dios, Kavita Gadar, Chris R Proctor, Evgenia Maslova, Jie Han, Mohamed Soliman, Dominika Krawiel, Emma L. Dunbar, Bhupender Singh, Stelinda Peros, Tom Killelea, Anna-Luisa Warnke, Marius M Haugland, Edward L. Bolt, Christian S. Lentz, Christian J. Rudolph, Ronan R McCarthy

APPENDIX

Appendix Table of Contents

1. Appendix Figure S1 Pg 2
2. Appendix Figure S2 Pg 3
3. Appendix Figure S3 Pg 4
4. Appendix Figure S4 Pg 5
5. Appendix Figure S5 Pg 6
6. Appendix Figure S6 Pg 7
7. Appendix Figure S7 Pg 8
8. Appendix Figure S8 Pg 9
9. Appendix Figure S9 Pg 10
10. Appendix Figure S10 Pg 11
11. Appendix Figure S11 Pg 12
12. Appendix Figure S12 Pg 13
13. Appendix Figure S13 Pg 14
14. Appendix Figure S14 Pg 15
15. Appendix Table S1 Pg 16

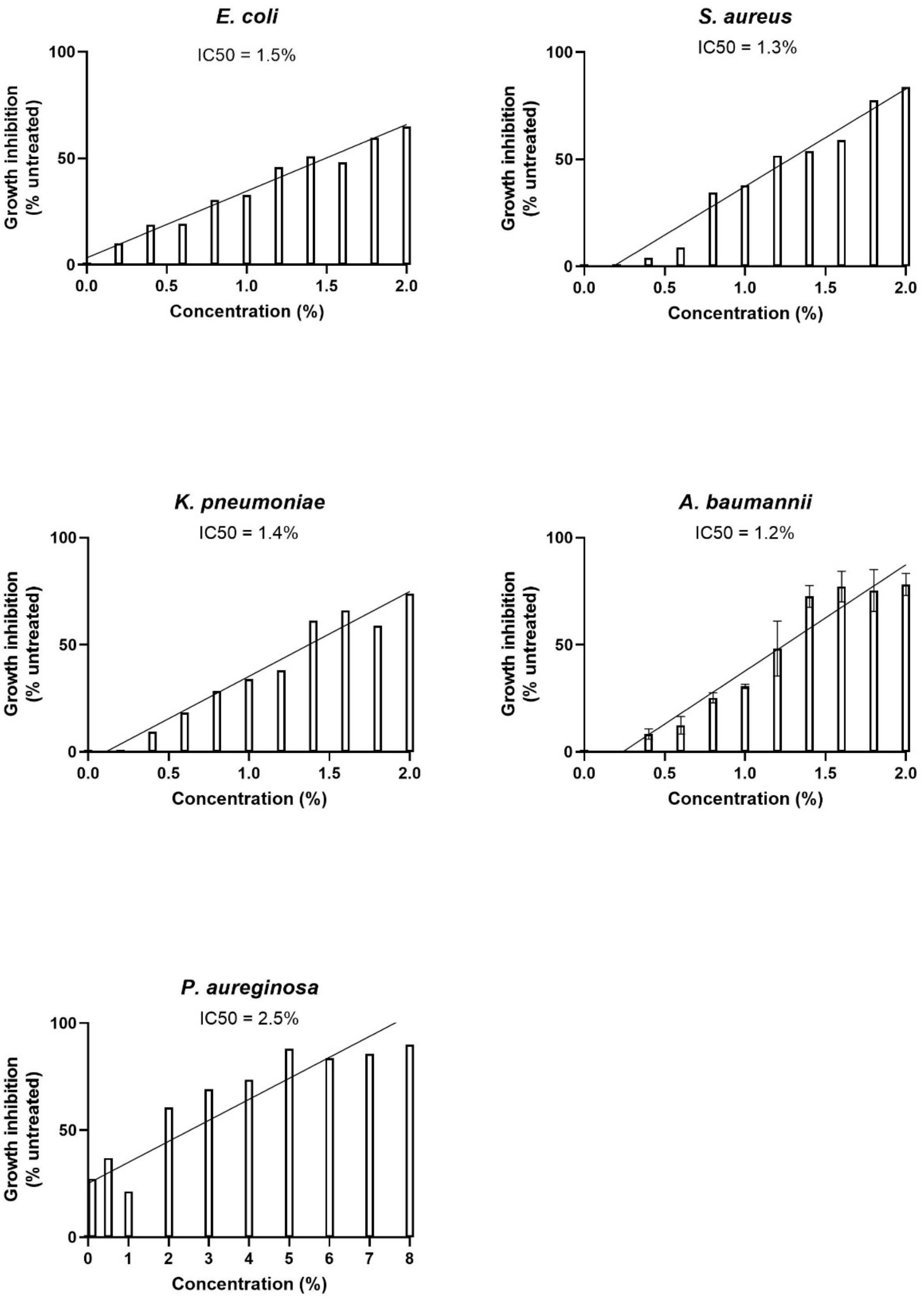

Appendix Figure S1: The saccharin concentration that inhibits 50% of the growth (IC50) of *E. coli*, *S. aureus*, *K. pneumoniae*, *A. baumannii* and *P. aeruginosa* were calculated.

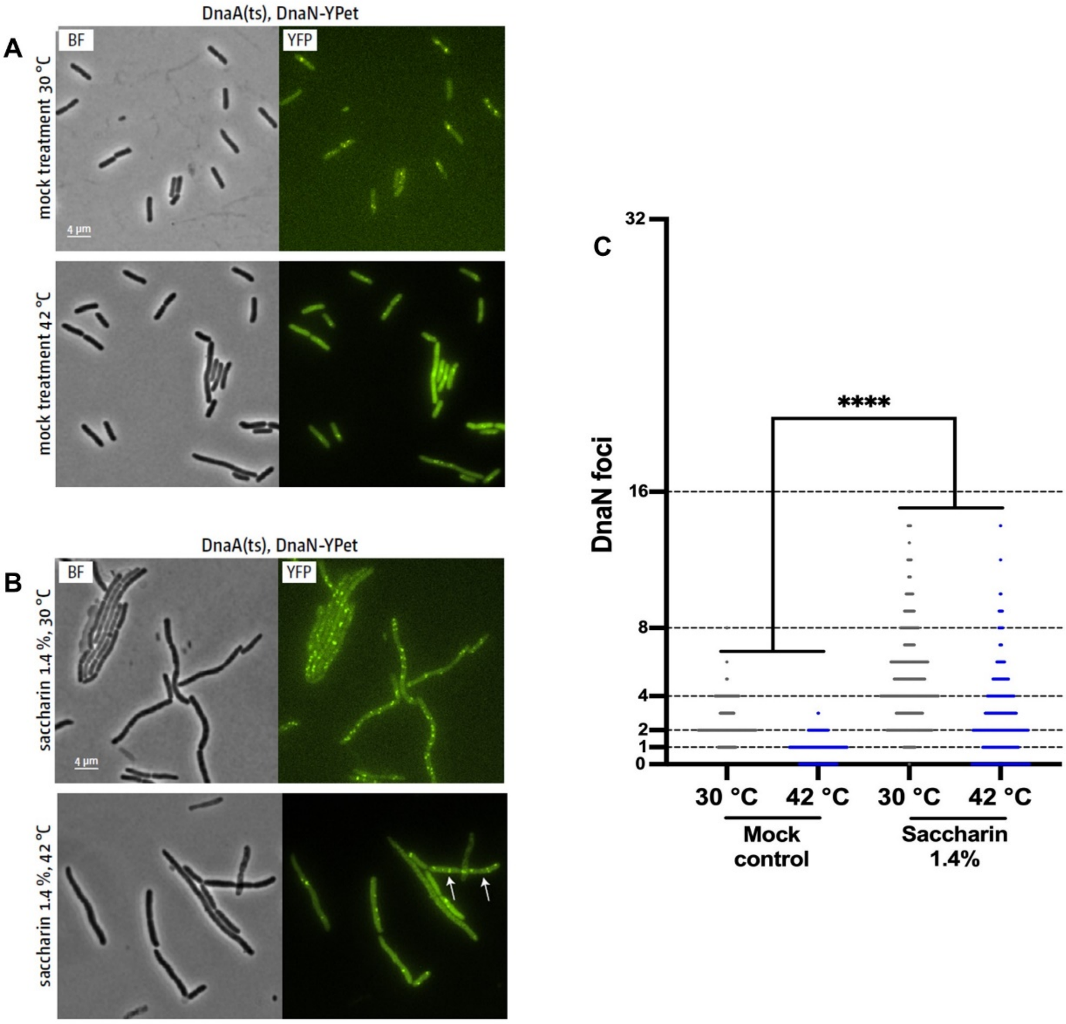

Appendix Figure S2: Behaviour of a fluorescently labelled DnaN reporter in an *E. coli* derivative encoding a thermosensitive allele of DnaA (*dnaA(ts)*). The results show an increment in the number of DnaN foci comparing a mock control (A) to cells treated with 1.4% saccharin (B) at both permissive (30 ºC) and restrictive temperature (42 ºC, which prevents replisome formation at *ori*). This showed that saccharin is triggering DNA synthesis away from the *ori*, with a significant increase in DnaN foci. The strain used was JD1459. (C). The number of cells analysed is 342 and 337 for the mock-treated-samples at 30 ºC and 42 ºC, respectively, and 339 and 320 for the saccharin-treated samples at 30 ºC and 42 ºC. Data were analysed by Mann-Whitney test comparing treated versus mock-treated groups at each temperature. Significance is indicated as **** = $p \leq 0.0001$. The images shown are representative examples from at least three biological replicates.

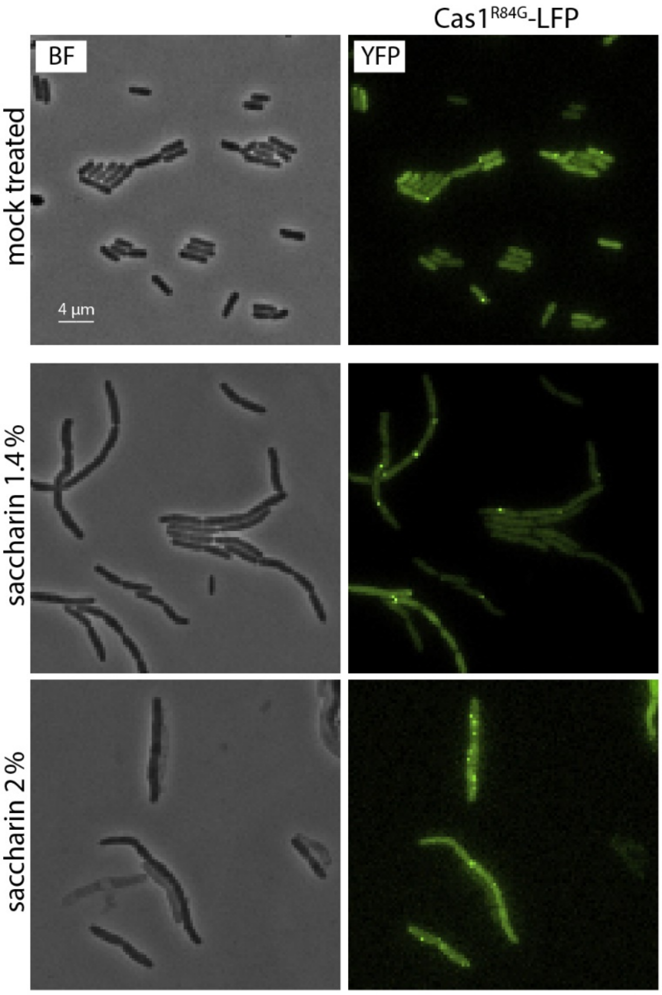

Appendix Figure S3: The dramatic amplification of Cas1-LFP foci following treatment with saccharin requires the ability of Cas1 to bind DNA. *E. coli* MG1655 cells carrying plasmid pTK136 (Killelea *et al*., 2023) were grown to early exponential growth phase in LB broth (Miller). Expression of $Cas1^{R84G}$-LFP and Cas2 was induced by the addition of 0.1% arabinose for 60 min before visualization. As shown recently (Killelea *et al*., 2023), $Cas1^{R84G}$ is observed only in a smaller number of cells and shows a distinctly aberrant polar pattern of localisation. Treatment with saccharin does not trigger the same dramatic increase in the number of wild type Cas1 foci (see Figure 6), highlighting that DNA binding is required for the amplification of Cas1-LFP-Cas2 foci. The images shown are representative examples from two biological replicates.

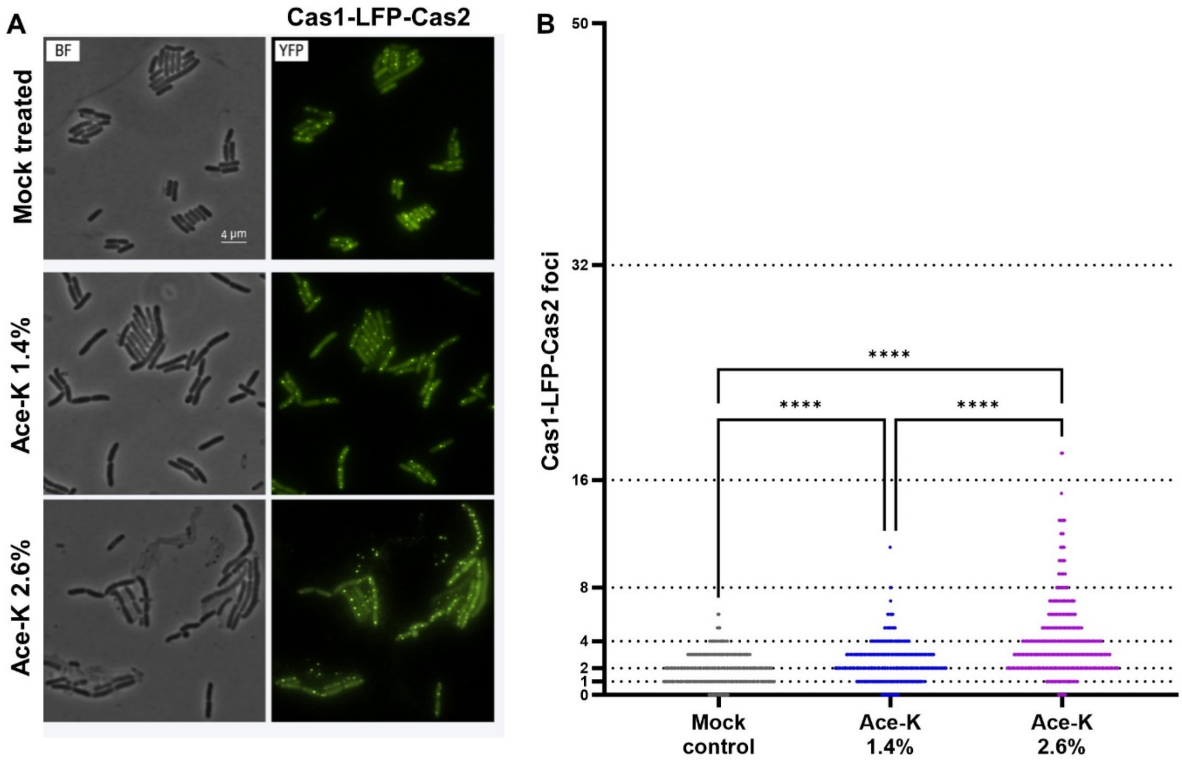

Appendix Figure S4: Cas1-LFP foci behaviour alters following treatment with ace-K. This effect, shown to be dose-dependent, was not as pronounced as that observed for saccharin, indicating a distinct signature for each sweetener regarding their impact on DNA replication. **A)** *E. coli* MG1655 cells were grown to early exponential growth phase in LB broth (Miller) and expression of Cas1-linker-eYFP (called Cas1-LFP for simplicity) and Cas2 induced by the addition of 0.1% arabinose for 60 min before visualization. The strain used was JD1708. The images shown are representative examples from a minimum of three biological replicates. **B)** Quantification of Cas1-LFP-Cas2 following ace-K treatment as from images shown in A). Shown are foci distributions highlighting the concentration-dependent increase of foci following treatment. The number of cells analysed is 602 for the mock-treated samples, 606 for the samples treated with 1.4% ace-K and 439 for the samples treated with 2% ace-K. Results shown in panel B were analysed by Kruskal-Wallis test with Dunn's correction. Significance is indicated as **** = p ≤0.0001.

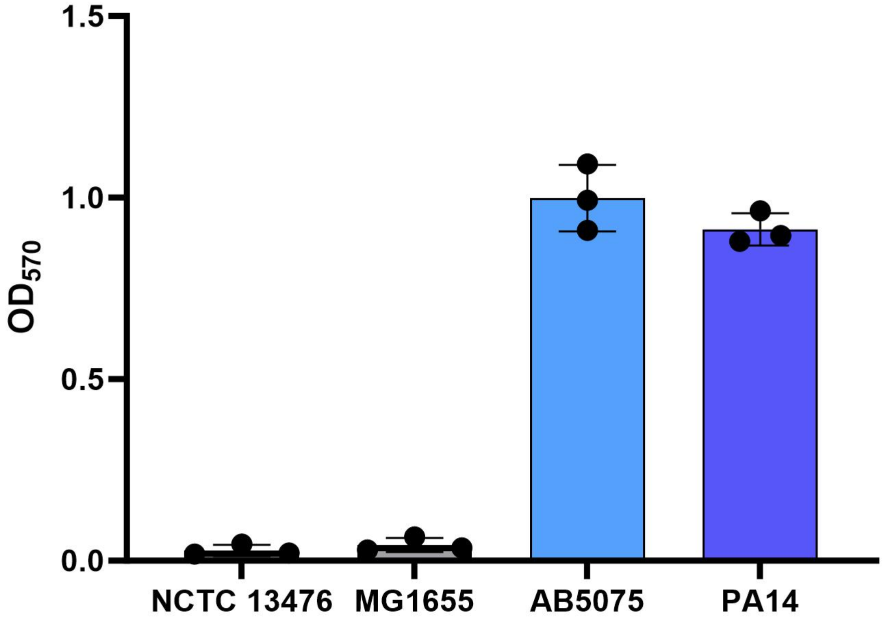

Appendix Figure S5: Biofilm formation of *E. coli* compared to *A. baumannii* AB5075 and *P. aeruginosa* PA14. Due to the negligible biofilm formation levels measured for the *E. coli* strains used in this work (MG1655 and NCTC 13476) in the conditions tested, we only measured the effect of saccharin on biofilm formation in AB5075 and PA14. This provided a better resolution for testing any potential anti-biofilm effect.

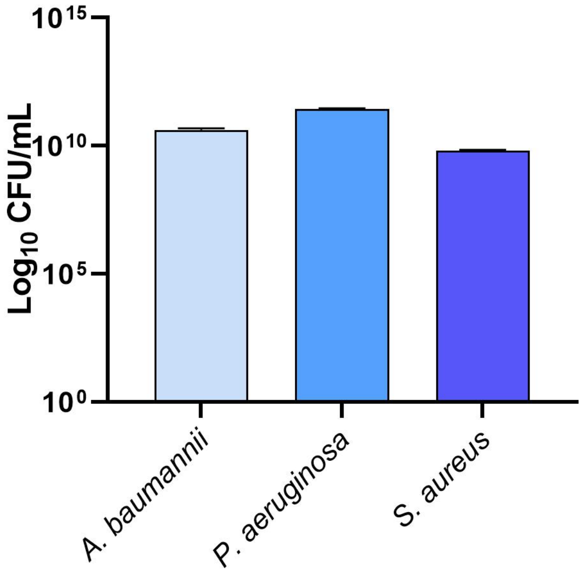

Appendix Figure S6. Viable cell counts of polymicrobial biofilms starting with 1:1:1 OD_{600} proportions of *A. baumannii*, *P. aeruginosa* and *S. aureus* and grown for 24 h in LB. Cells embedded in the biofilms were mechanically resuspended, serially diluted and plated on selective media for each species, including LB agar supplemented with gentamycin (20 mg/L), *Pseudomonas* isolation agar and mannitol salt agar for *A. baumannii*, *P. aeruginosa* and *S. aureus*, respectively.

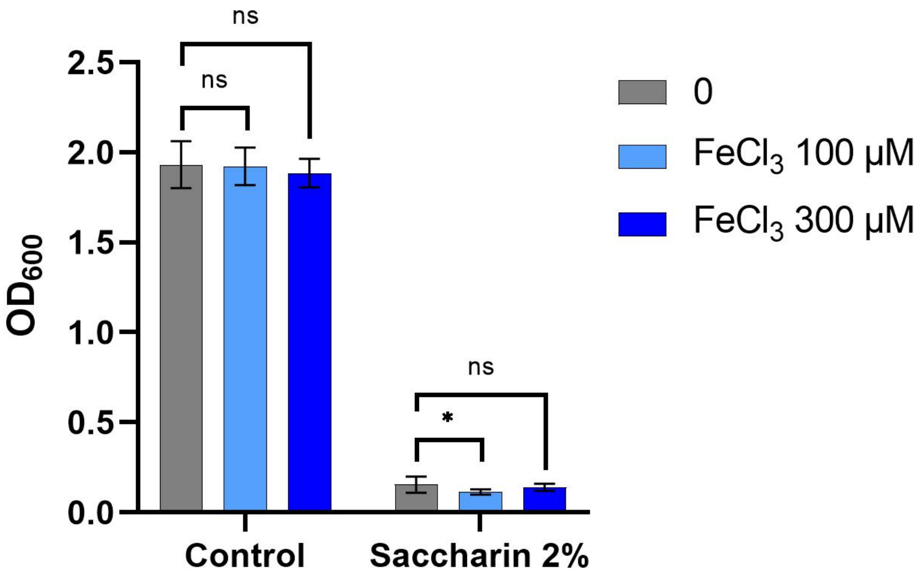

Appendix Figure S7: Iron supplementation does not rescue saccharin growth inhibition in *A. baumannii* AB5075. Multiple genes involved in iron homeostasis and/or related to iron binding are differentially regulated in the presence of saccharin. To assess if saccharin was interfering with iron acquisition/metabolism, we hypothesised that an iron supplementation would relieve the growth inhibition exerted by saccharin. To test this, we supplemented saccharin treated cells, compared to an untreated control, with 0, 100 and 300 µM $FeCl_3$, which are higher than those considered iron-replete conditions (Sheldon and Skaar, 2020). As a result, we could not see any significant recovery in growth in saccharin-treated cultures (2%) in the presence of $FeCl_3$ compared to the control culture not supplemented with $FeCl_3$. Average values of 3 biological replicates ± S. D. are represented. Statistical analysis was performed by t-test between iron-supplemented and the corresponding non-supplemented control (* = $p \leq 0.05$; ns = non-significant).

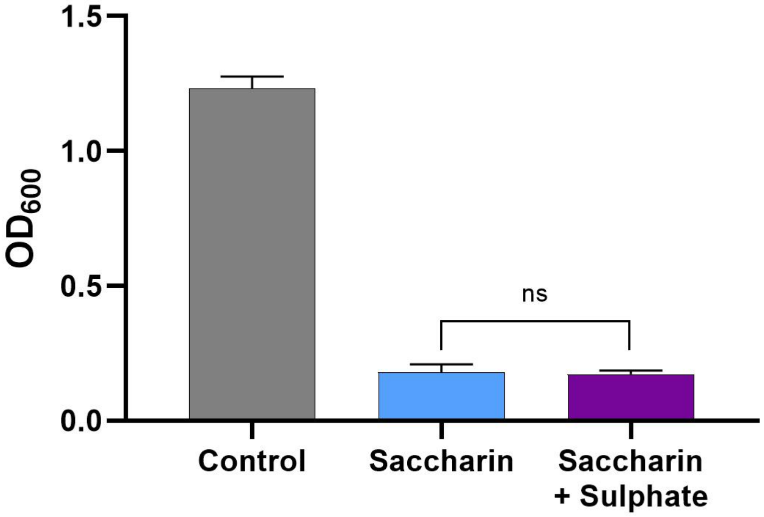

Appendix Figure S8: Sulphur supplementation does not rescue saccharin growth inhibition in *A. baumannii* AB5075. Multiple genes involved in sulphur metabolism and uptake are differentially regulated in the presence of saccharin. To assess if saccharin was interfering with sulphur acquisition/metabolism, we hypothesised that a sulphate supplementation would relieve the growth inhibition exerted by saccharin. To test this, we supplemented saccharin treated cells, compared to an untreated control, with 5 mM K_2SO_4, which is in excess with respect to that used in M9 minimal media (1 mM). As a result, we could not see any significant recovery in growth in saccharin-treated cultures (2%) in the presence of K_2SO_4 compared to the control culture not supplemented with K_2SO_4. Average values of 3 biological replicates ± S. D. are represented. Statistical analysis was performed by t-test between iron-supplemented and the corresponding non-supplemented control (ns = non-significant).

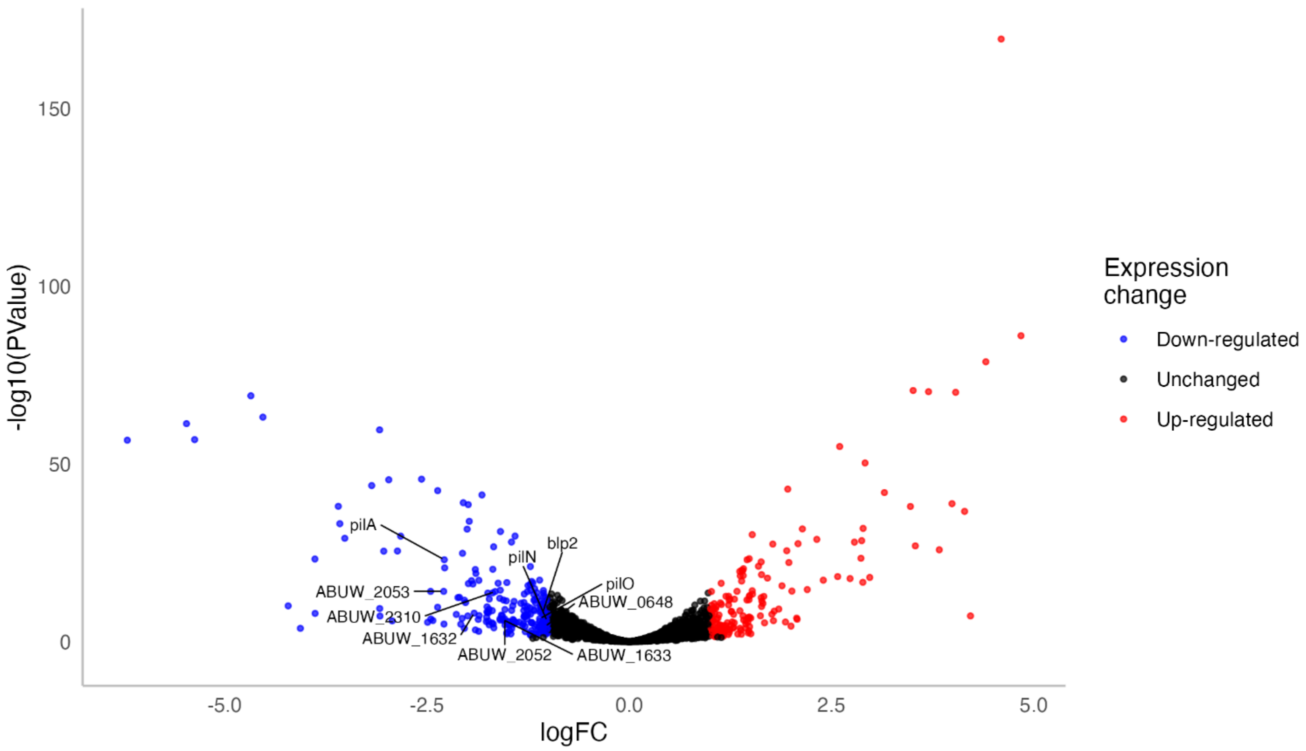

Appendix Figure S9: Saccharin produces an impact on different functional groups of genes in MDR *A. baumannii* AB5075. Volcano plot representing dRNA-seq results comparing cells treated with 1% saccharin to a mock treatment. According to the dRNA-seq results, 165 genes were upregulated (red) and 215 were downregulated (blue). ABUW_0607, an outlier due to low p-value, was included in this representation.

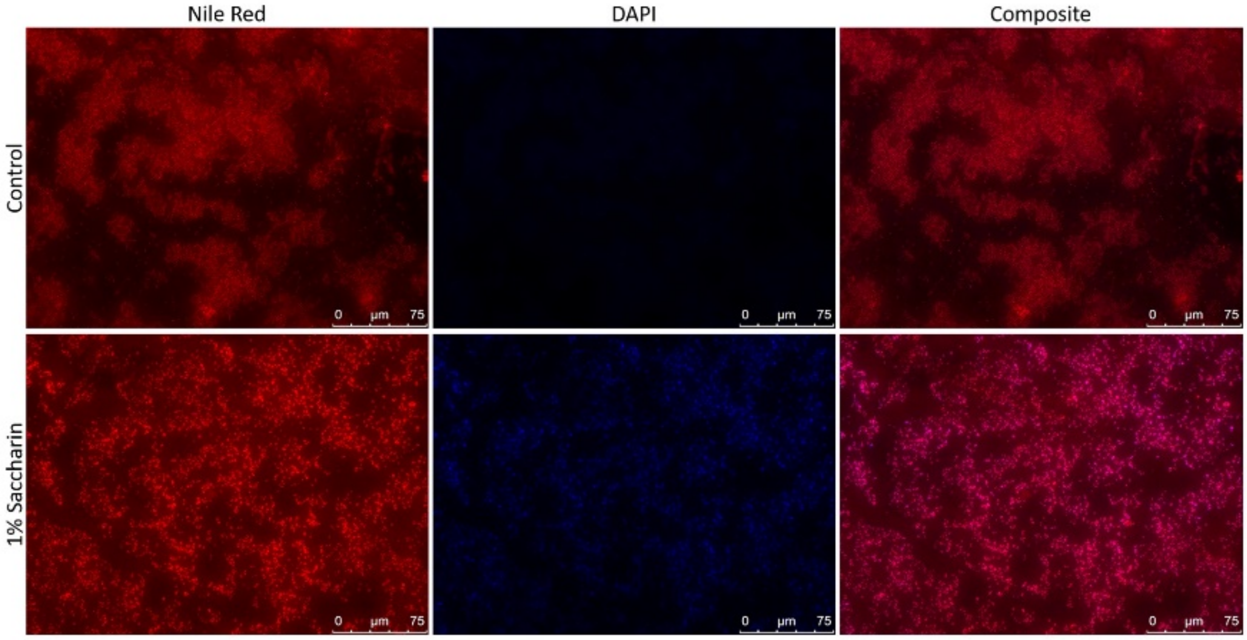

Appendix Figure S10: Differential fluorescence microscopy using DAPI and Nile red. AB5075 treated with a vehicle control showed extensive staining of the membrane with Nile red and minimal uptake od DAPI. When grown in the presence of 1% saccharin a marked increase in DAPI staining was observed. This is indicative of a permeabilized membrane. Representative images out of 3 independent replicates are shown.

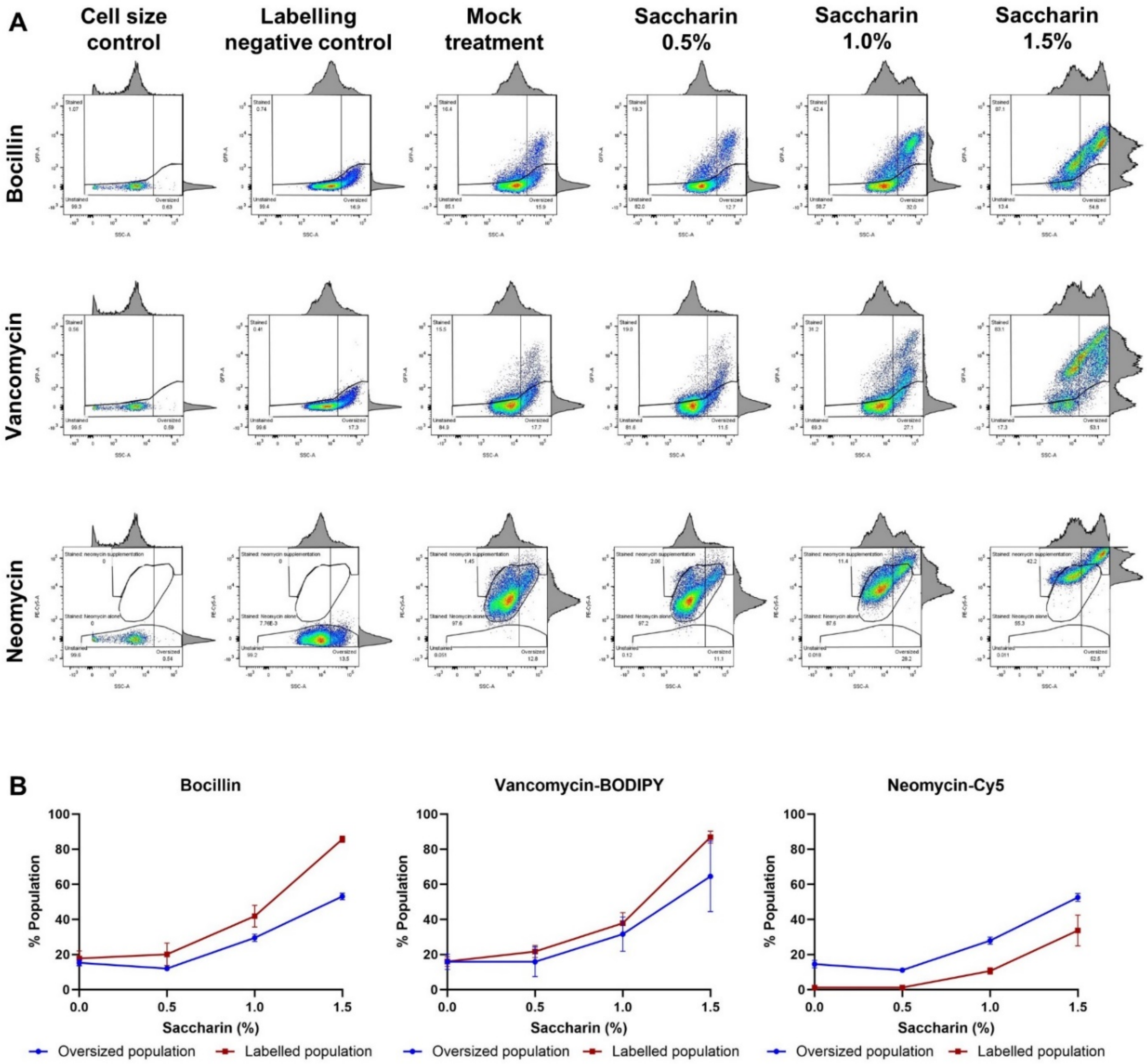

Appendix Figure S11: A) Gating controls used to analyse the results of flow cytometry experiments and results obtained with the full range of saccharin concentrations (mock treatment, 0.5%, 1% and 1.5%). As a control for gating *A. baumannii* AB5075 oversized cells with respect to regular-size cells, we performed a parallel flow cytometry experiment (as explained in the Methods section) with cells coming from a stationary phase culture (cells not actively growing) and set the threshold as the size of 99% of the events counted by the flow cytometer. As a control for gating fluorescent antibiotic (Bocillin, Vancomycin-BODIPY or Neomycin-Cy5) labelled *A. baumannii* AB5075 cells with respect to unstained cells, we performed a parallel flow cytometry experiment (as explained in the Methods section) with exponential phase cells without the addition of the respective probe and set the threshold as the fluroescence of 99% of the events counted by the flow cytometer. **B)** The percentage of *A. baumannii* AB5075 population in exponential phase with an increased size with respect to the size control shown in panel A is represented by the blue line trend. The effect of saccharin on cell size shows a dose-dependent response. The *A. baumannii* AB5075 population in exponential phase which were labelled with each fluorescent antibiotic with respect to the labelling negative control shown in panel A is represented by the blue line trend. The effect of saccharin on fluorescent antibiotic labelling shows a dose-dependent response. The images shown in panel A are representative examples of 3 biological replicates. The result shown in panel B represent the average of 3 biological replicates ± S.D.

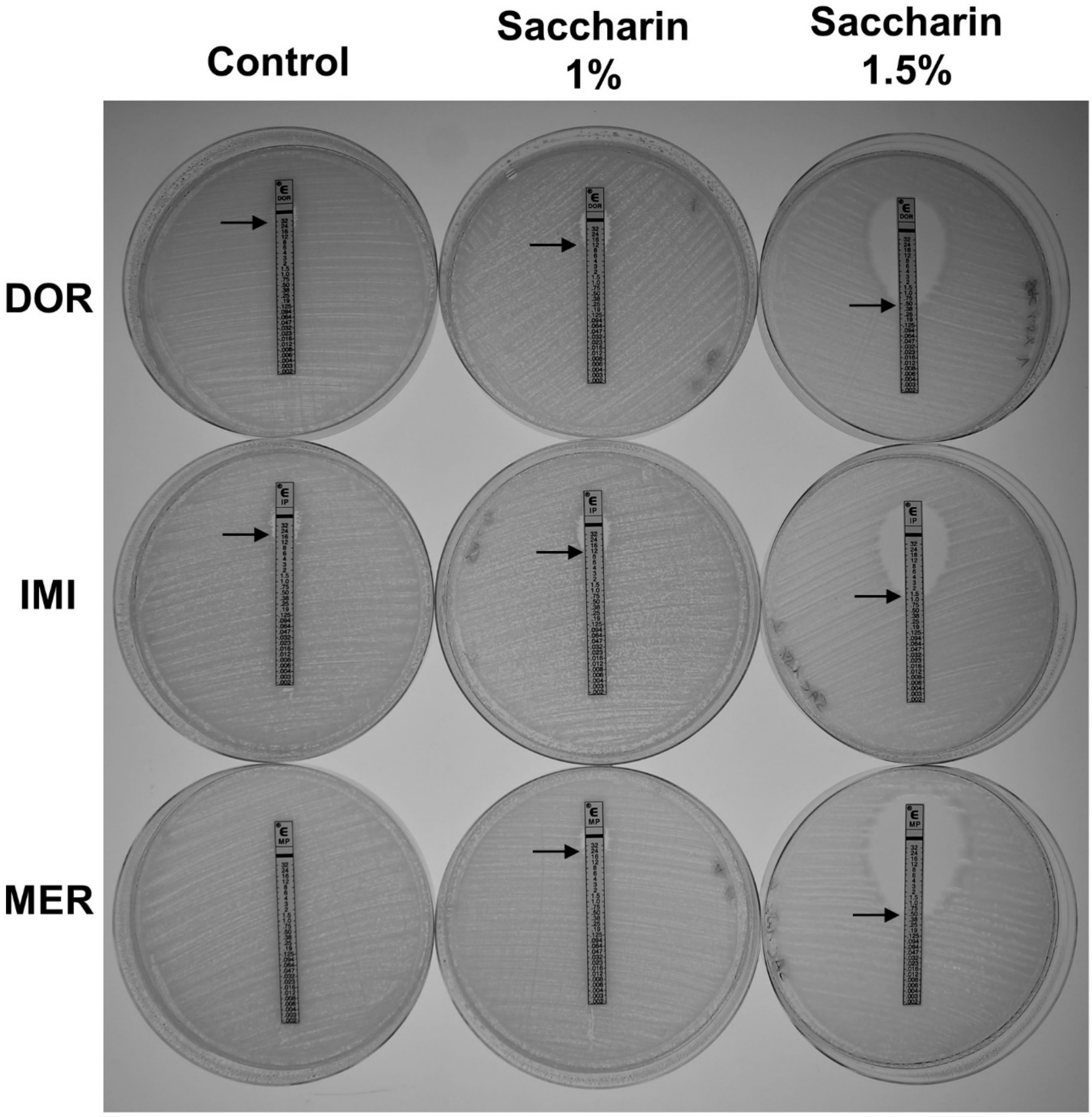

Appendix Figure S12: Minimum inhibitory concentration test of the carbapenems doripenem (DOR), imipenem (IMI) and meropenem (MER) for *A. baumannii* AB5075 measured with Etest strips. The assays were performed on cation-adjusted Mueller-Hinton agar (see Methods section) supplemented with 1% or 1.5% saccharin on a vehicle control. The arrows indicate the intersection between the zone of inhibition and the Etest strip, which was taken as the minimum inhibitory concentration reading as per the manufacturer's instructions. The image shown is a representative example of 3 biological replicates.

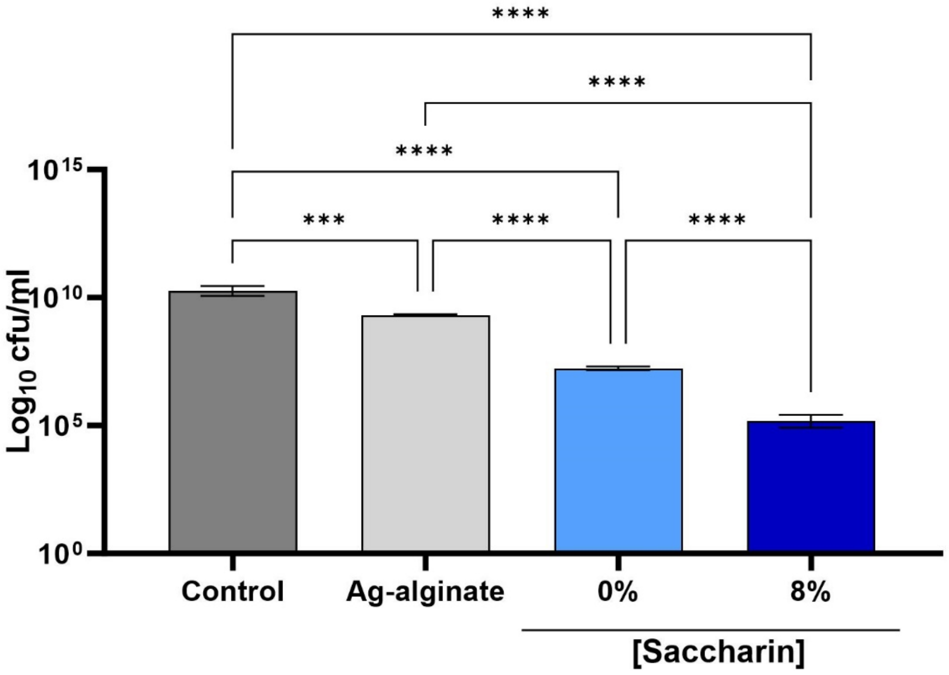

Appendix Figure S13: Saccharin shows therapeutic potential in topical treatments: Saccharin-loaded hydrogels produce a reduction in the bacterial numbers compared to a vehicle control and a commercial silver-alginate dressing when applied for 1 h on an *A. baumannii* AB5075 3.5 h grown (early stage) colony biofilm on an agar plate.

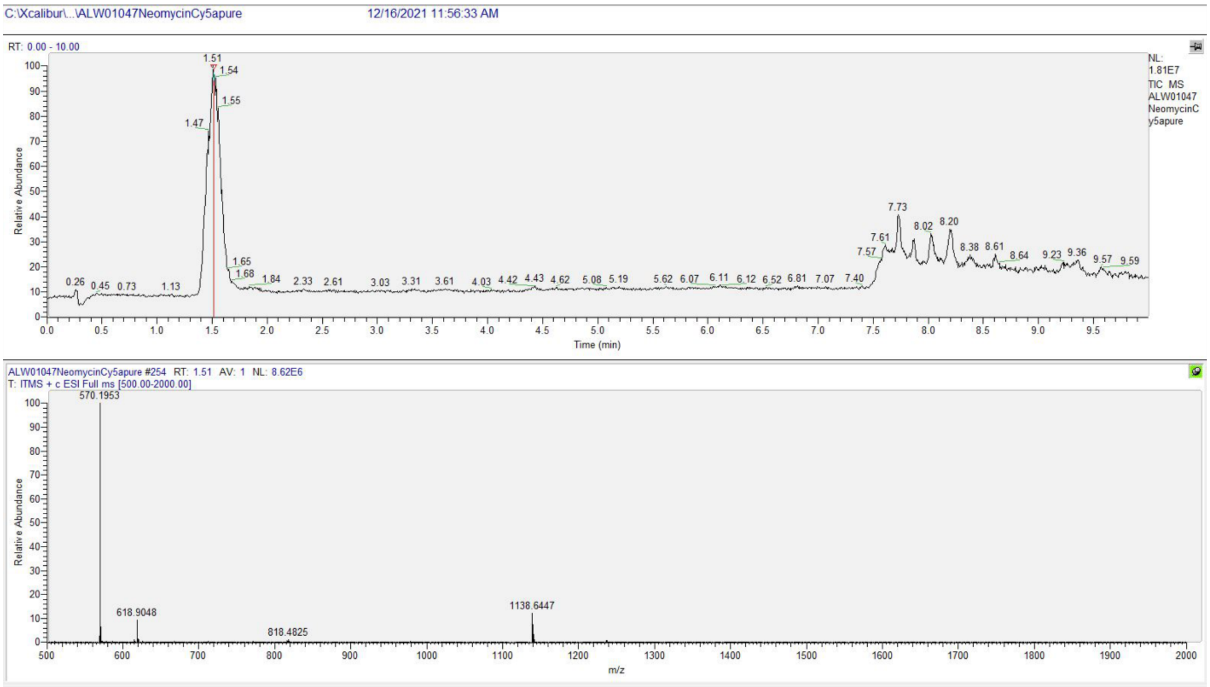

Appendix Figure S14. HPLC-HRMS analysis of neomycin-Cy5 (SI-6).

Appendix Table S1. Exact p-values obtained for the statistical comparisons performed in this work.

**Figure panel and comparison**	**Statistical test and p-value**
Figure 2B	Mann-Whitney test
Saccharin vs. control	<0.0001
Figure 2D	Mann-Whitney test
Saccharin vs. control	<0.0001
Figure 2F	Kruskal-Wallis test (Dunn's correction)
Mock control vs. Saccharin 1.4%	<0.0001
Mock control vs. Saccharin 2%	<0.0001
Saccharin 1.4% vs. Saccharin 2%	0.0002
Figure 3	Kruskal-Wallis test (Dunn's correction)
WT vs. Δ*priB*	>0.9999
WT vs. Δ*priC*	>0.9999
WT vs. WT	<0.0001
WT vs. Δ*priB*	<0.0001
WT vs. Δ*priC*	<0.0001
Δ*priB* vs. Δ*priC*	>0.9999
Δ*priB* vs. WT	<0.0001
Δ*priB* vs. Δ*priB*	<0.0001
Δ*priB* vs. Δ*priC*	<0.0001
Δ*priC* vs. WT	<0.0001
Δ*priC* vs. Δ*priB*	<0.0001
Δ*priC* vs. Δ*priC*	<0.0001
WT vs. Δ*priB*	<0.0001
WT vs. Δ*priC*	<0.0001
Δ*priB* vs. Δ*priC*	>0.9999
Figure 4A	Two-way ANOVA (Sidak's correction)
Mock treated - Saccharin	
0	>0.9999
0.1	0.0985
0.5	0.661
1	0.3096
2	<0.0001
3	<0.0001
4	<0.0001
5	<0.0001
6	<0.0001
7	<0.0001
8	<0.0001
Figure 4B	Two-way ANOVA (Sidak's correction)
Mock treated - Saccharin	
0	0.2242
0.1	>0.9999
0.5	>0.9999

1	>0.9999
2	<0.0001
3	<0.0001
4	<0.0001
5	<0.0001
6	<0.0001
7	<0.0001
8	<0.0001
Figure 4C	Two-way ANOVA (Sidak's correction)
Mock treated - Saccharin	
0	0.6936
0.1	0.54
0.5	0.0014
1	0.0036
2	<0.0001
3	<0.0001
4	<0.0001
5	<0.0001
6	<0.0001
7	<0.0001
8	<0.0001
Figure 4D	Two-way ANOVA (Sidak's correction)
Mock treated - Saccharin	
0	0.9939
0.1	>0.9999
0.5	>0.9999
1	>0.9999
2	<0.0001
3	<0.0001
4	<0.0001
5	<0.0001
6	<0.0001
7	<0.0001
8	<0.0001
Figure 4E	Two-way ANOVA (Sidak's correction)
Mock treated - Saccharin	
0	>0.9999
0.1	>0.9999
0.5	0.9998
1	0.8968
2	0.0033
3	0.0001
4	<0.0001
5	<0.0001

6	<0.0001
7	<0.0001
8	<0.0001
Figure 5A	Two-way ANOVA (Sidak's correction)
Mock treated - Saccharin	
0	0.9992
0.1	0.5461
0.5	0.6757
1	>0.9999
2	<0.0001
3	<0.0001
4	<0.0001
5	<0.0001
6	<0.0001
7	<0.0001
8	<0.0001
Figure 5B	Two-way ANOVA (Sidak's correction)
Mock treated - Saccharin	
0	0.9999
0.1	0.0001
0.5	<0.0001
1	<0.0001
2	<0.0001
3	<0.0001
4	<0.0001
5	<0.0001
6	<0.0001
7	<0.0001
8	<0.0001
Figure 5C	One-way ANOVA (Dunnett's correction)
Mock treated vs. *A. baumannii*	0.0003
Mock treated vs. *P. aeruginosa*	<0.0001
Figure 5D	One-way ANOVA (Dunnett's correction)
0 vs. 0.25	0.9172
0 vs. 0.5	<0.0001
0 vs. 1	<0.0001
Figure 5E	Unpaired t-test
Saccharin vs. control	0.0002
Figure 5F	Unpaired t-test
Saccharin vs. control	<0.0001
Figure 5G	One-way ANOVA (Tukey's correction)
Mock treated vs. Saccharin	<0.0001
Mock treated vs. Saccharin + Cations	<0.0001

Saccharin vs. Saccharin + Cations	0.0007
Figure 6C	Two-way ANOVA (Tukey's correction)
Doripenem	
Control vs. Saccharin 1%	0.1278
Control vs. Saccharin 1.5%	<0.0001
Saccharin 1% vs. Saccharin 1.5%	0.0003
Imipenem	
Control vs. Saccharin 1%	0.5695
Control vs. Saccharin 1.5%	<0.0001
Saccharin 1% vs. Saccharin 1.5%	<0.0001
Meropenem	
Control vs. Saccharin 1%	0.5695
Control vs. Saccharin 1.5%	<0.0001
Saccharin 1% vs. Saccharin 1.5%	<0.0001
Figure 7A	One-way ANOVA (Tukey's correction)
Control vs. Ag-alginate	0.0005
Control vs. 0%	<0.0001
Control vs. 8%	<0.0001
Ag-alginate vs. 0%	<0.0001
Ag-alginate vs. 8%	<0.0001
0% vs. 8%	<0.0001
Figure 7B	Unpaired t-test
Saccharin vs. control	<0.0001
