# Supplementary figures and images for: Saccharin disrupts bacterial cell envelope stability and interferes with DNA replication dynamics (part 1 of 2)
Source: EMBO Mol Med. 2025 Apr 1;17(5):993–1017. doi: 10.1038/s44321-025-00219-1 (PMC12081710; doi:10.1038/s44321-025-00219-1)

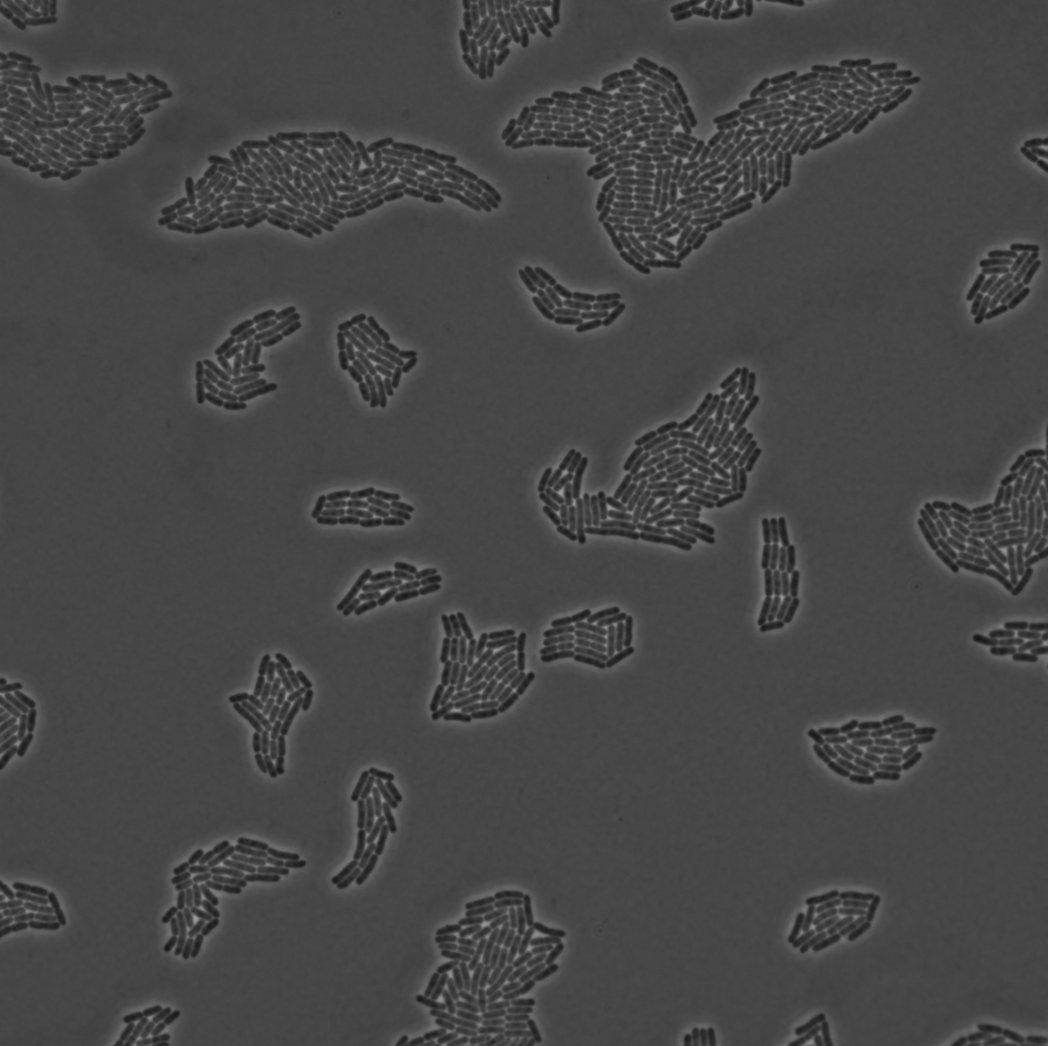

Supplement: Supplementary file 13 — Source data Fig. 1 [file 44321_2025_219_MOESM13_ESM.zip › 1Ai/mg1655 mu 0 percent saccharinc1c1t21.tif]

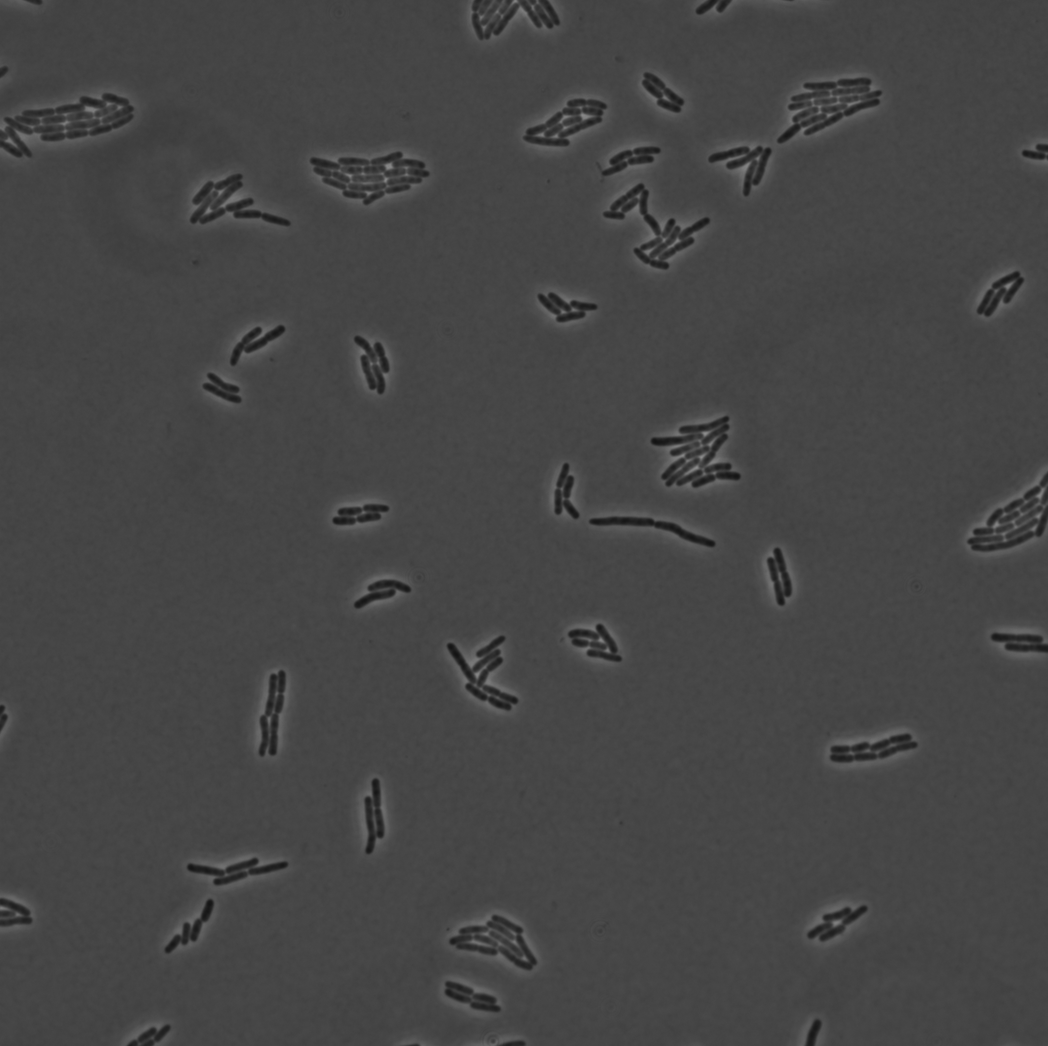

Supplement: Supplementary file 13 — Source data Fig. 1 [file 44321_2025_219_MOESM13_ESM.zip › 1Ai/mg1655 mu 0 percent saccharinc1c1t09.tif]

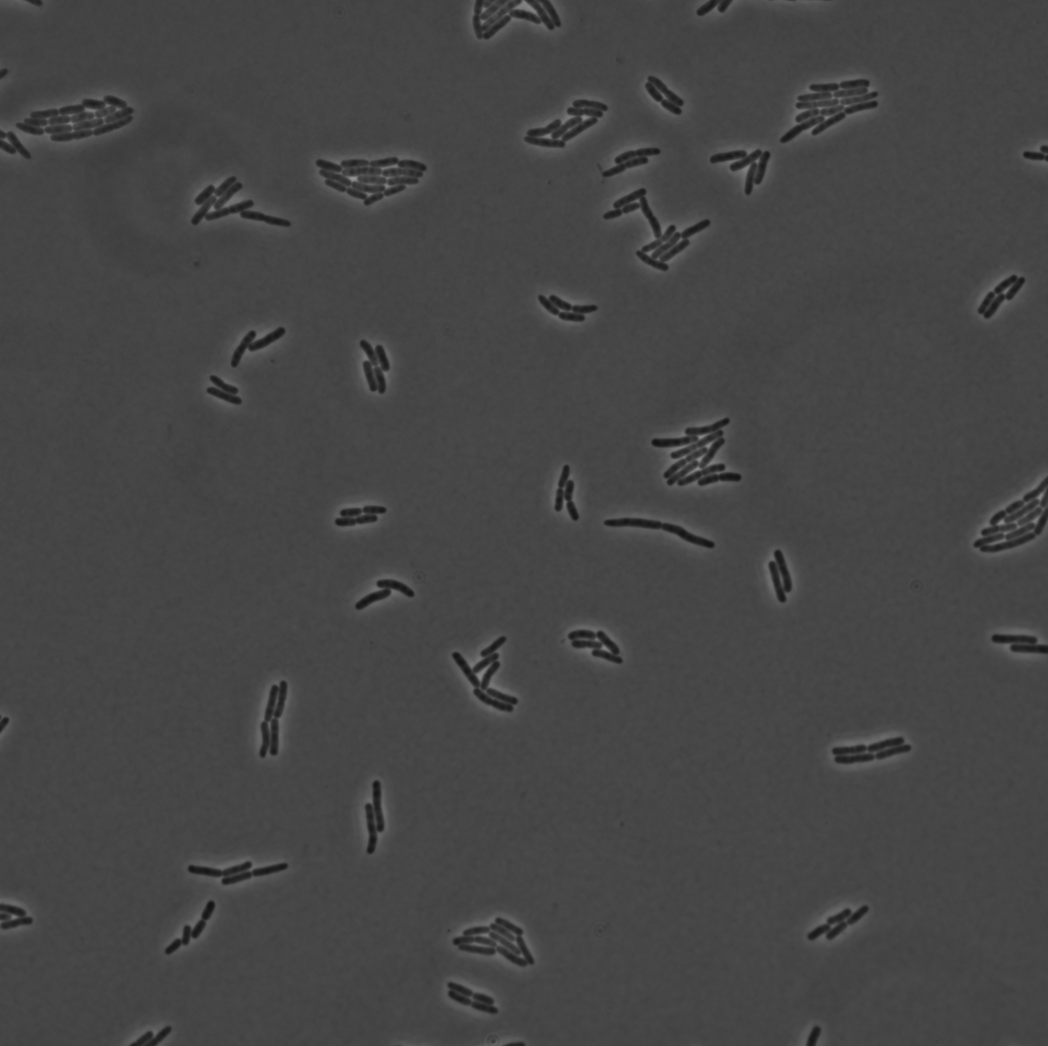

Supplement: Supplementary file 13 — Source data Fig. 1 [file 44321_2025_219_MOESM13_ESM.zip › 1Ai/mg1655 mu 0 percent saccharinc1c1t08.tif]

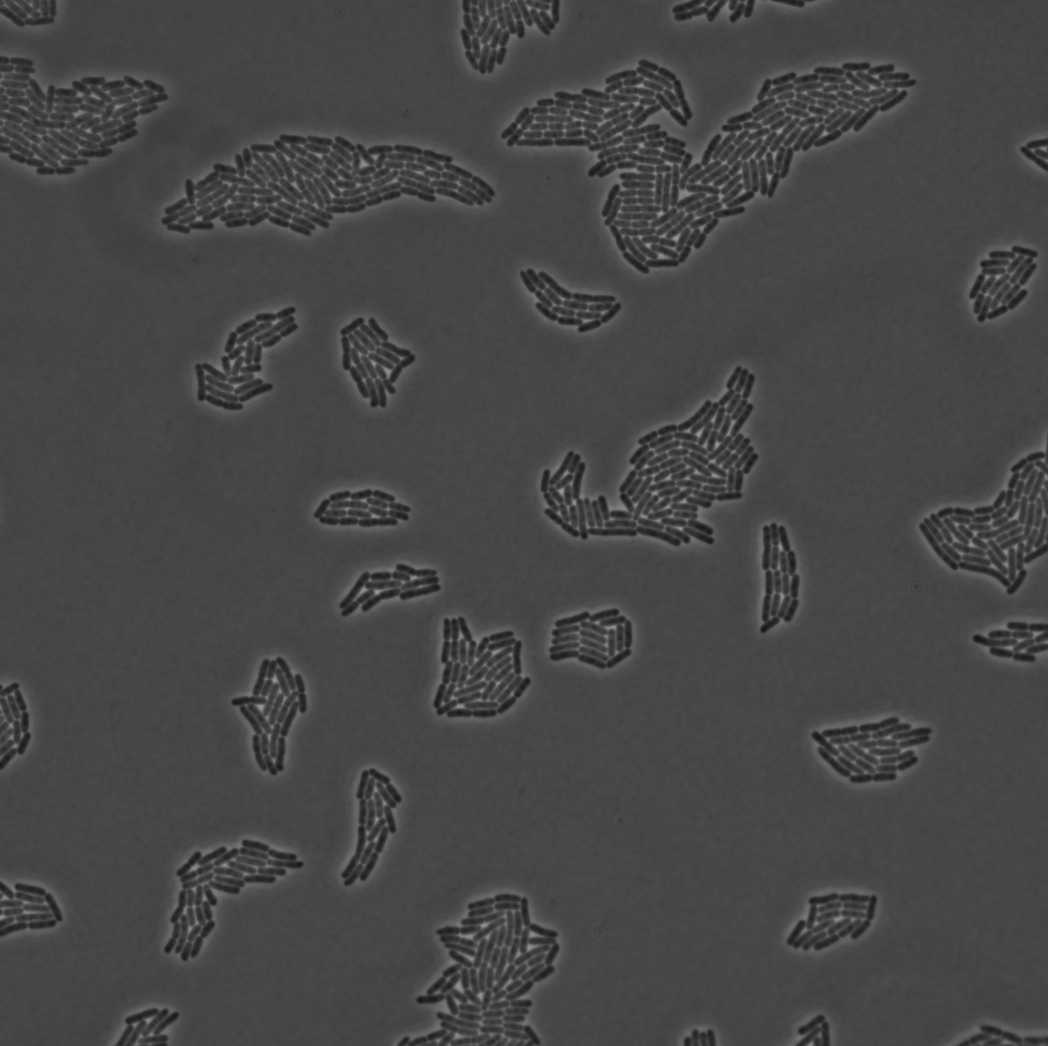

Supplement: Supplementary file 13 — Source data Fig. 1 [file 44321_2025_219_MOESM13_ESM.zip › 1Ai/mg1655 mu 0 percent saccharinc1c1t20.tif]

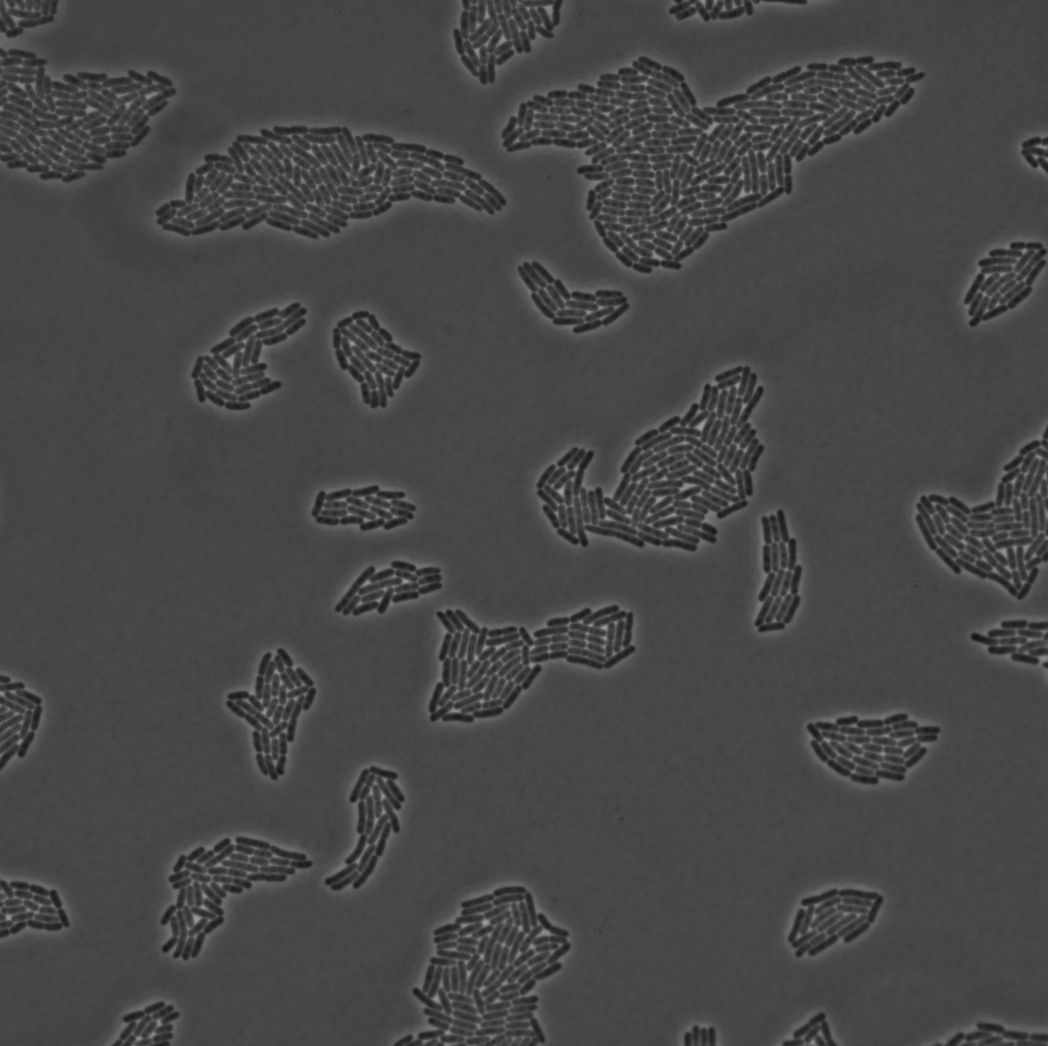

Supplement: Supplementary file 13 — Source data Fig. 1 [file 44321_2025_219_MOESM13_ESM.zip › 1Ai/mg1655 mu 0 percent saccharinc1c1t22.tif]

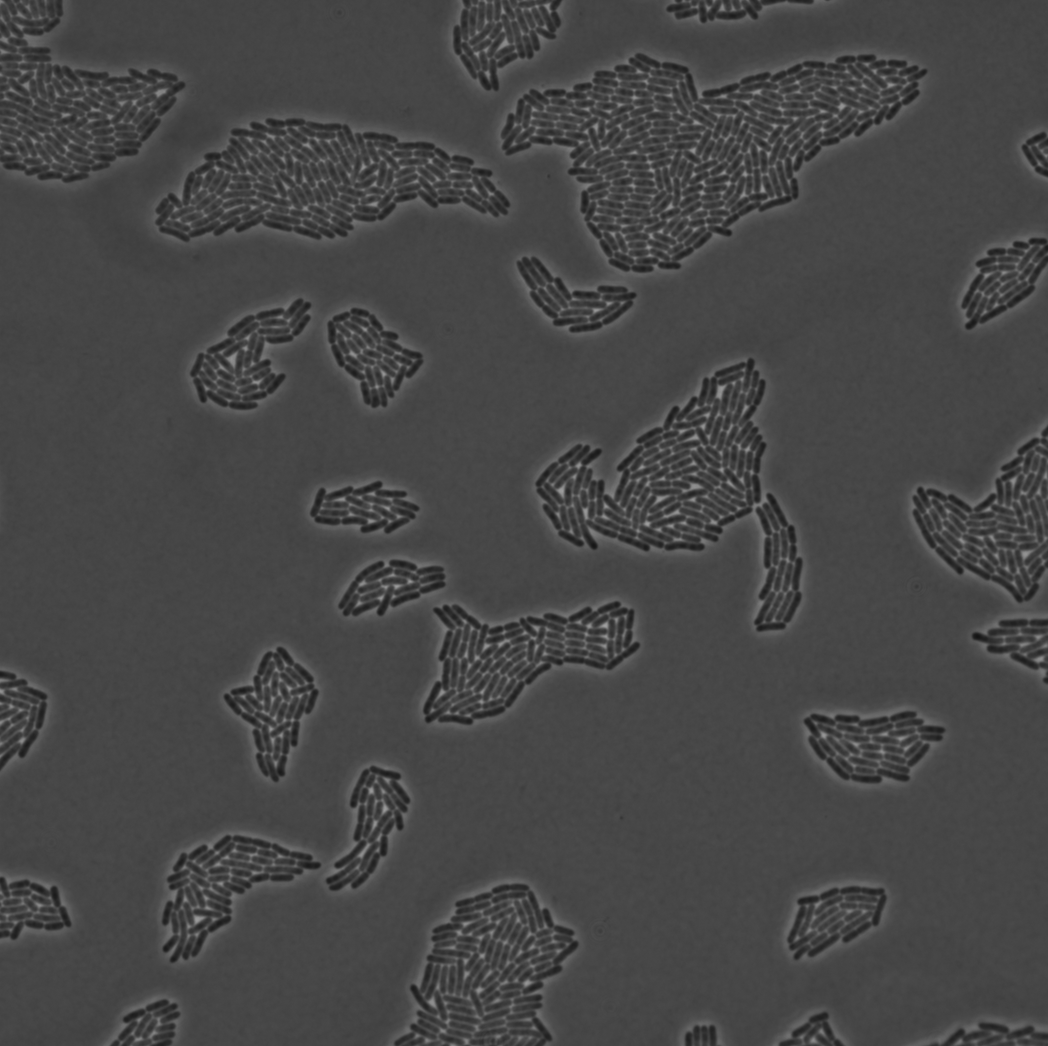

Supplement: Supplementary file 13 — Source data Fig. 1 [file 44321_2025_219_MOESM13_ESM.zip › 1Ai/mg1655 mu 0 percent saccharinc1c1t23.tif]

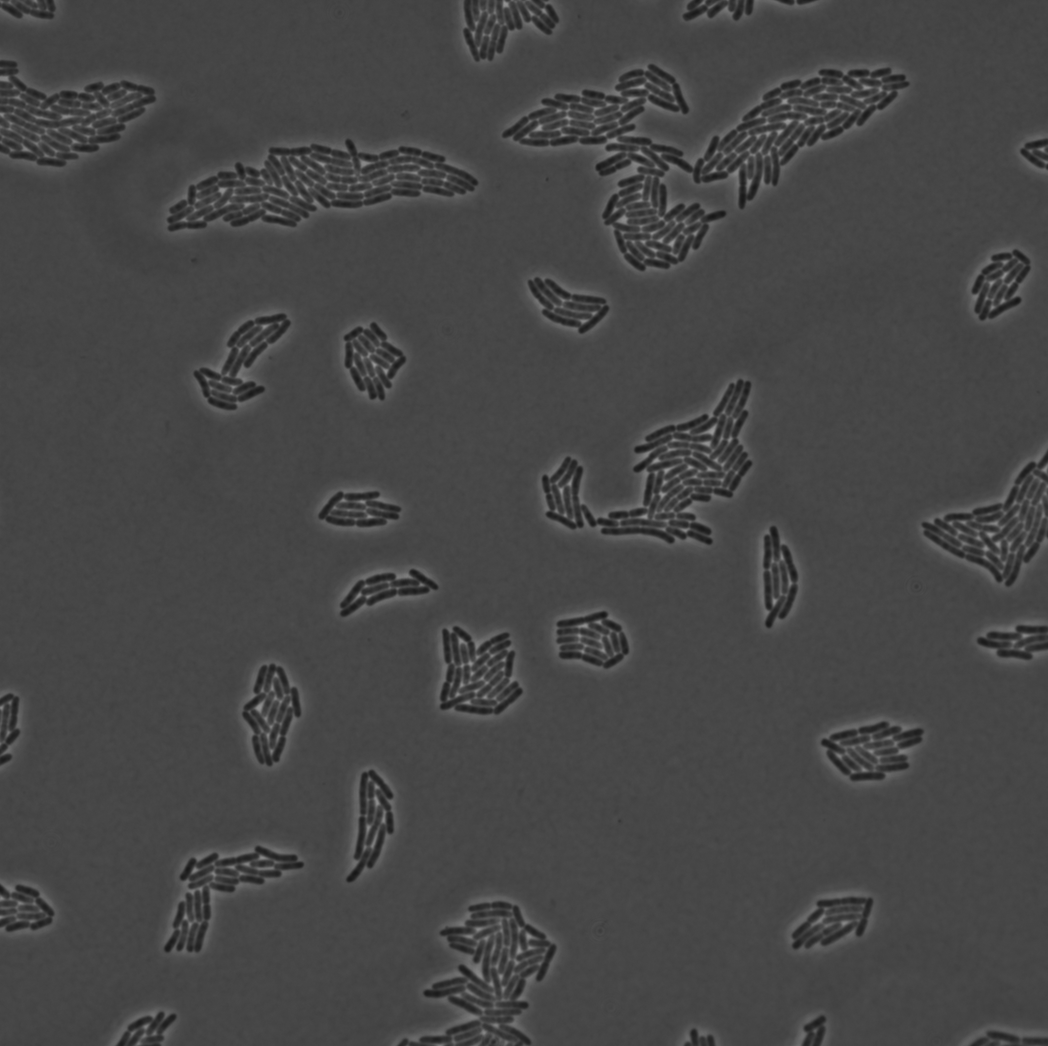

Supplement: Supplementary file 13 — Source data Fig. 1 [file 44321_2025_219_MOESM13_ESM.zip › 1Ai/mg1655 mu 0 percent saccharinc1c1t18.tif]

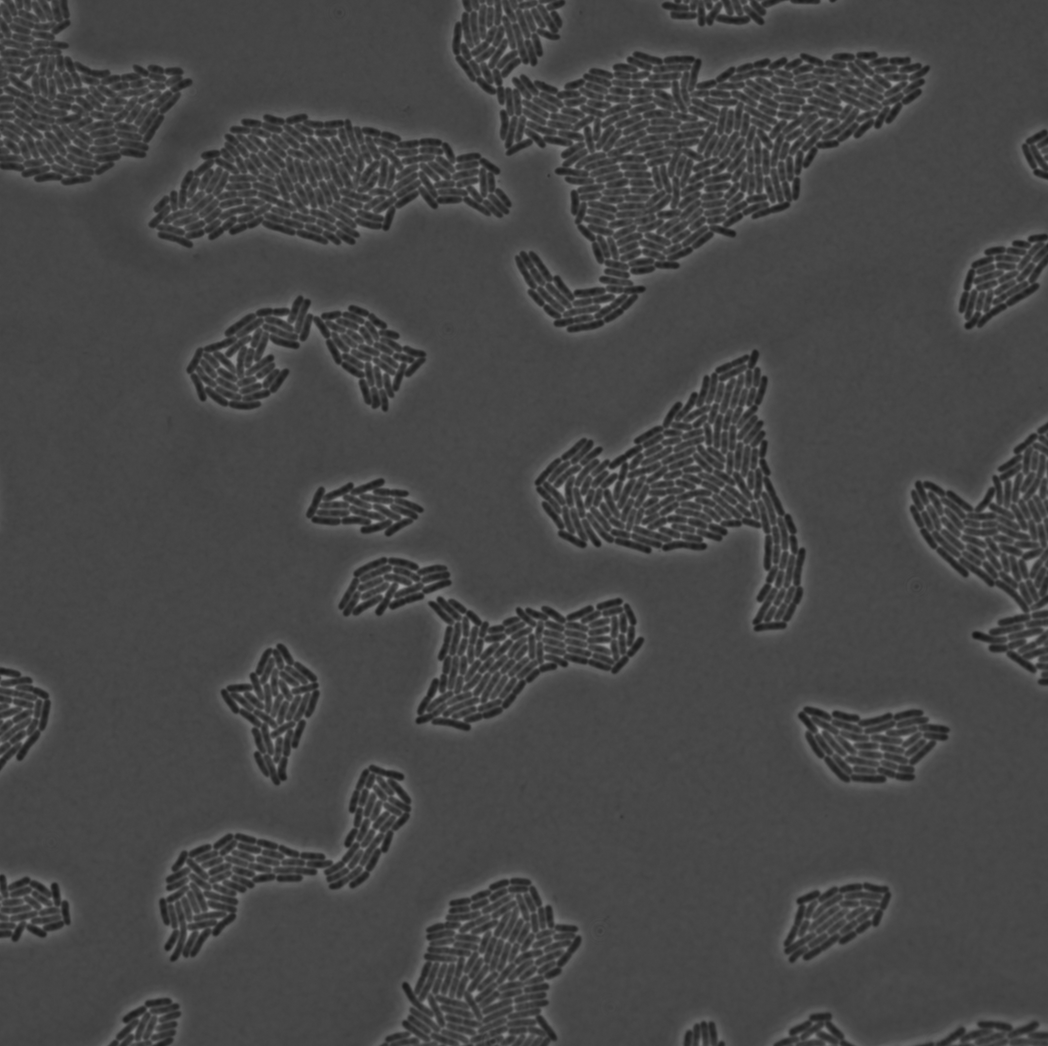

Supplement: Supplementary file 13 — Source data Fig. 1 [file 44321_2025_219_MOESM13_ESM.zip › 1Ai/mg1655 mu 0 percent saccharinc1c1t24.tif]

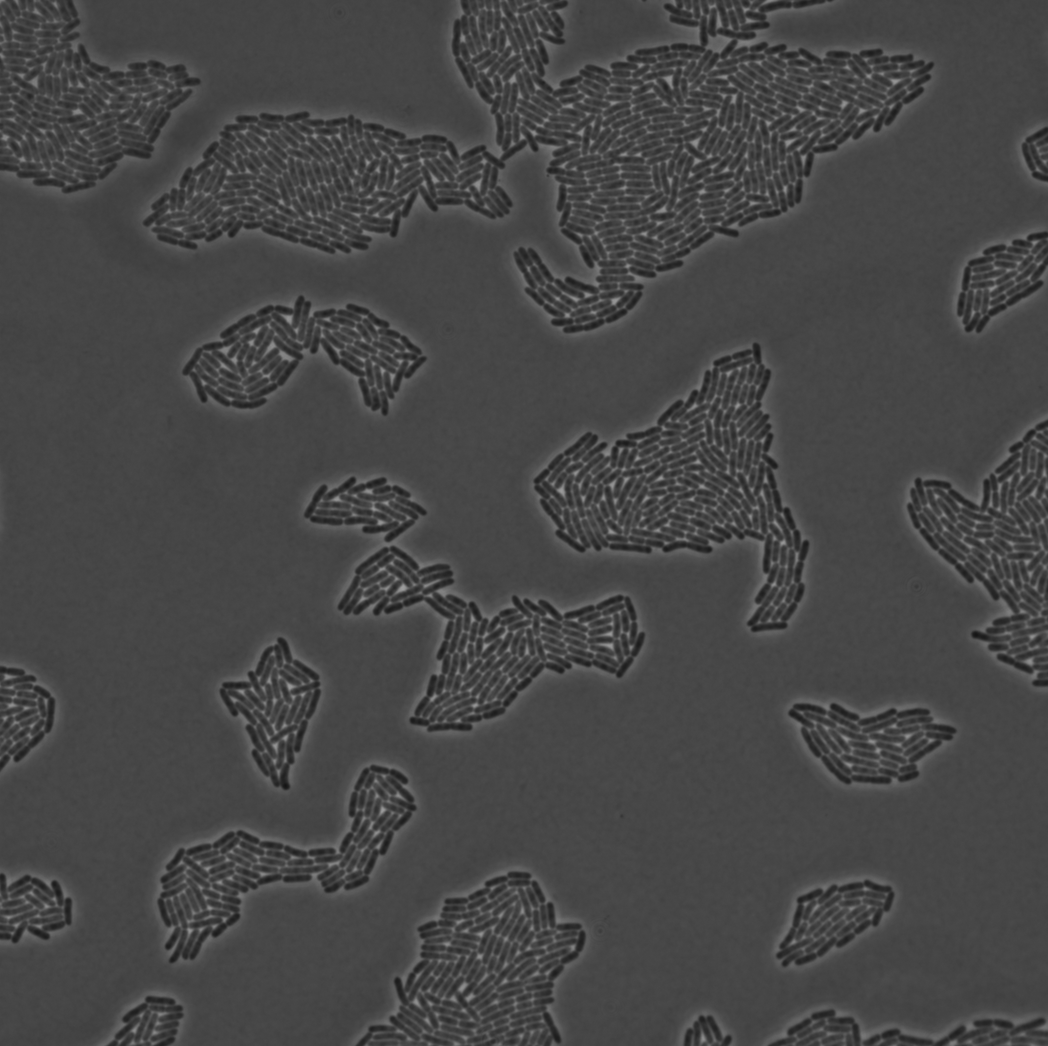

Supplement: Supplementary file 13 — Source data Fig. 1 [file 44321_2025_219_MOESM13_ESM.zip › 1Ai/mg1655 mu 0 percent saccharinc1c1t25.tif]

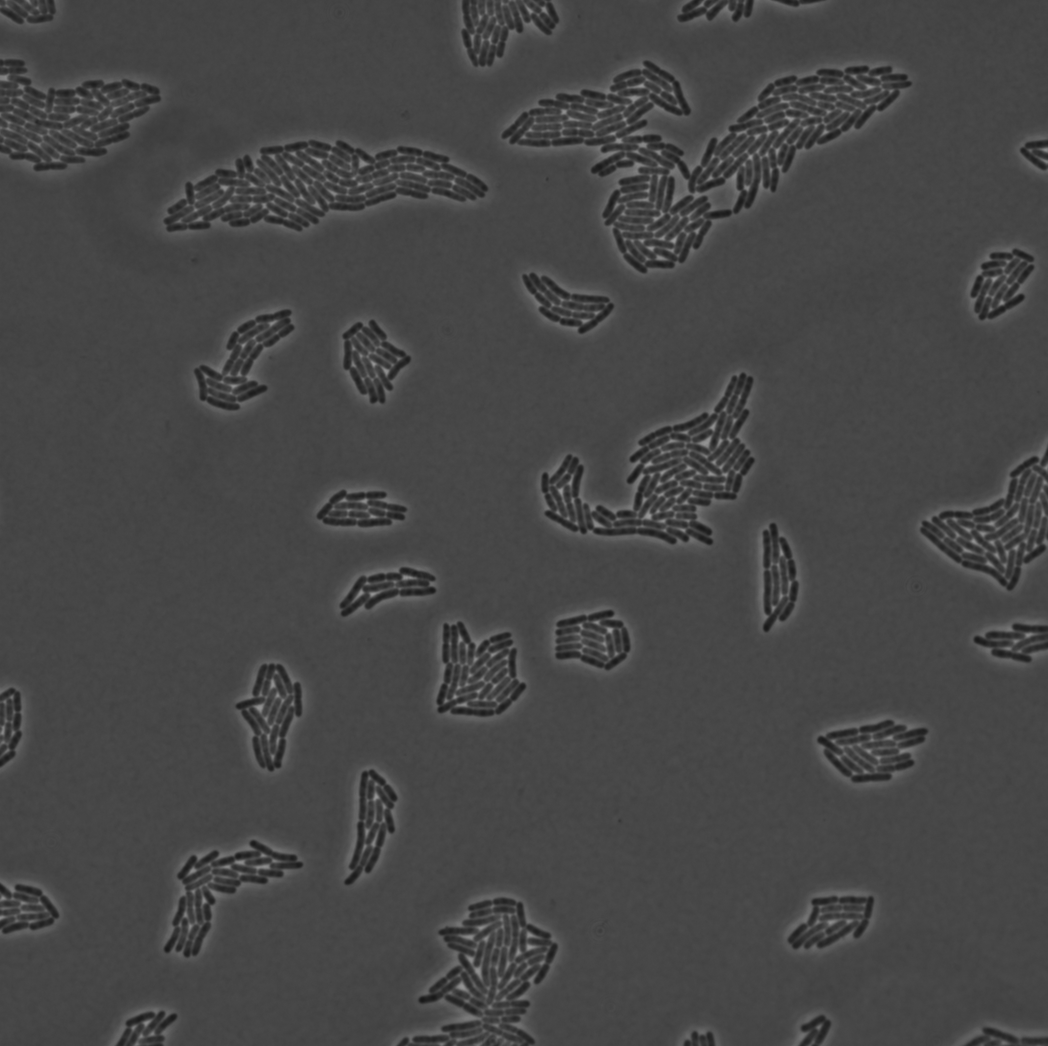

Supplement: Supplementary file 13 — Source data Fig. 1 [file 44321_2025_219_MOESM13_ESM.zip › 1Ai/mg1655 mu 0 percent saccharinc1c1t19.tif]

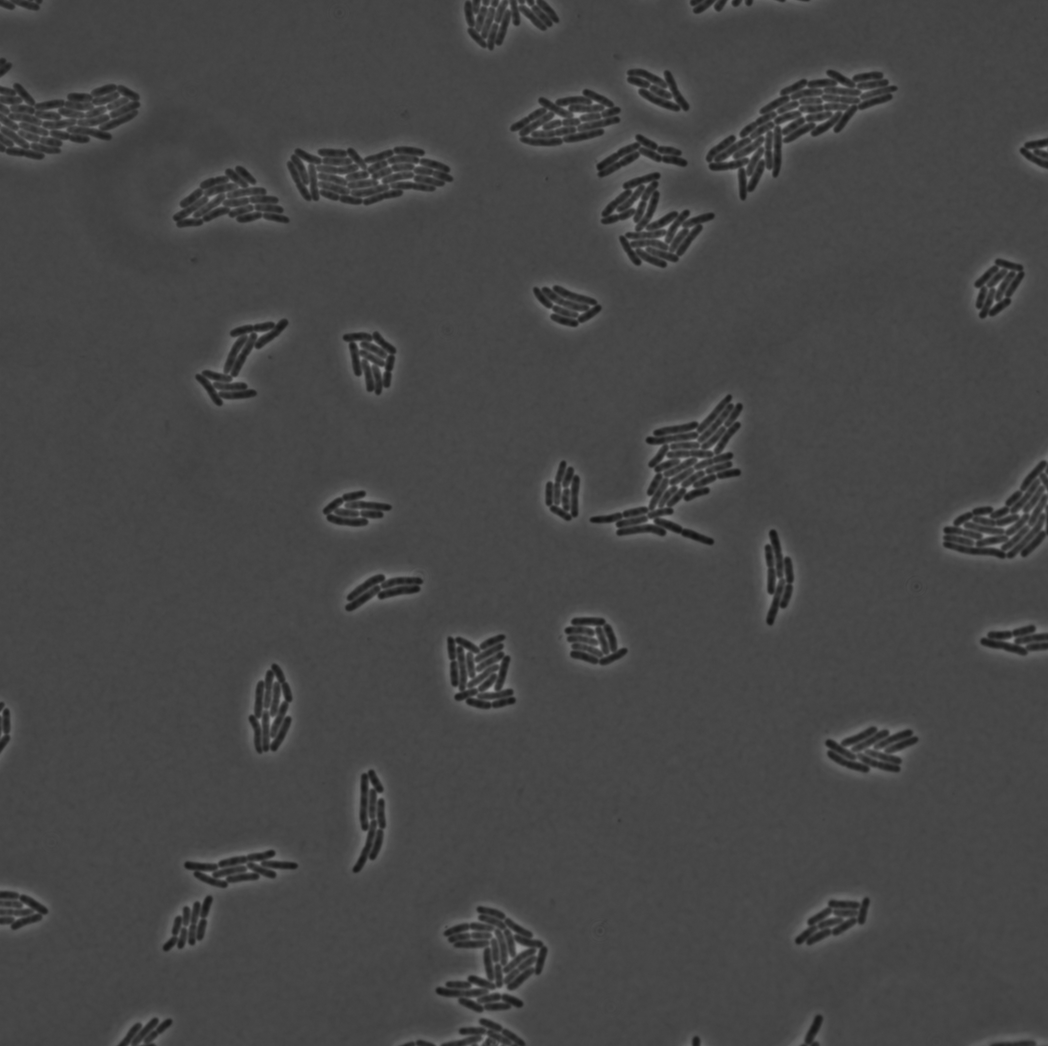

Supplement: Supplementary file 13 — Source data Fig. 1 [file 44321_2025_219_MOESM13_ESM.zip › 1Ai/mg1655 mu 0 percent saccharinc1c1t14.tif]

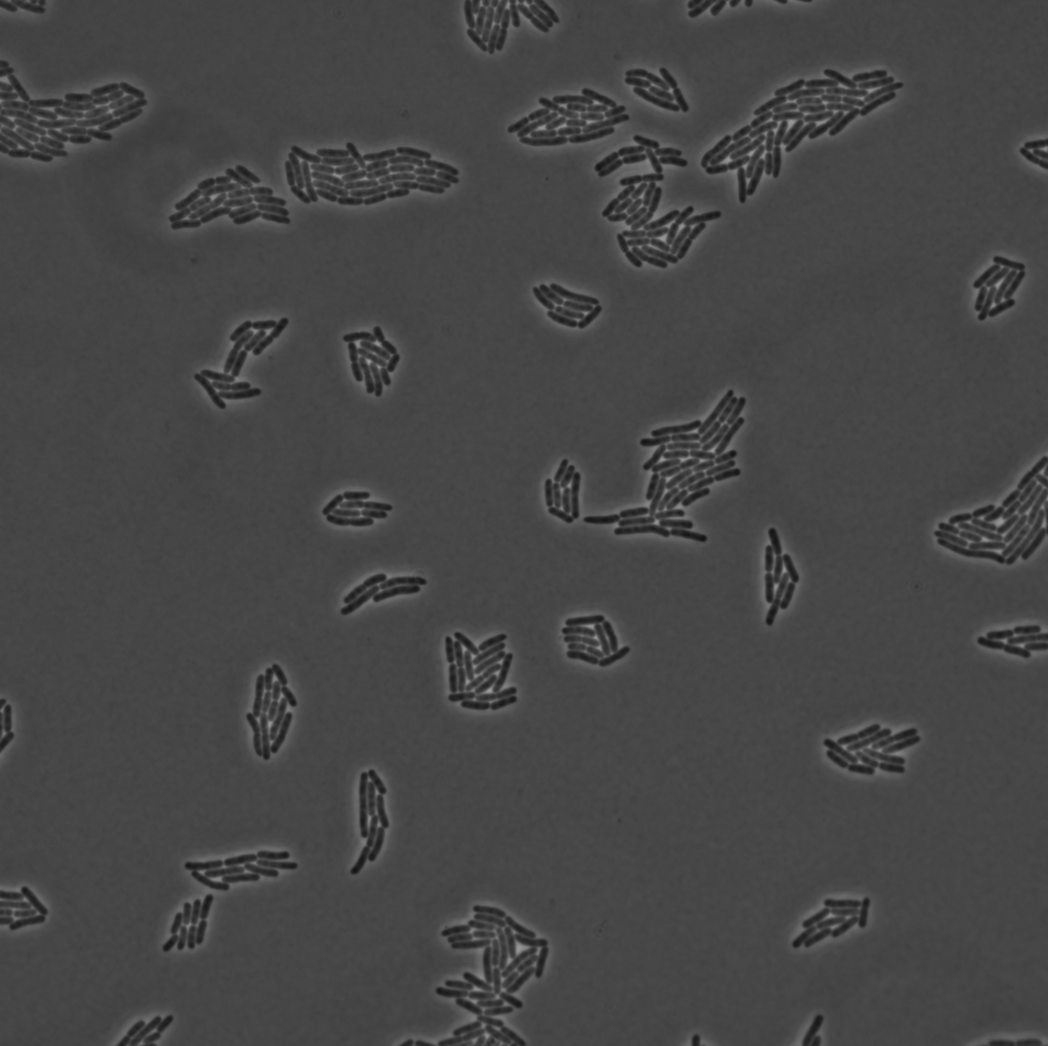

Supplement: Supplementary file 13 — Source data Fig. 1 [file 44321_2025_219_MOESM13_ESM.zip › 1Ai/mg1655 mu 0 percent saccharinc1c1t15.tif]

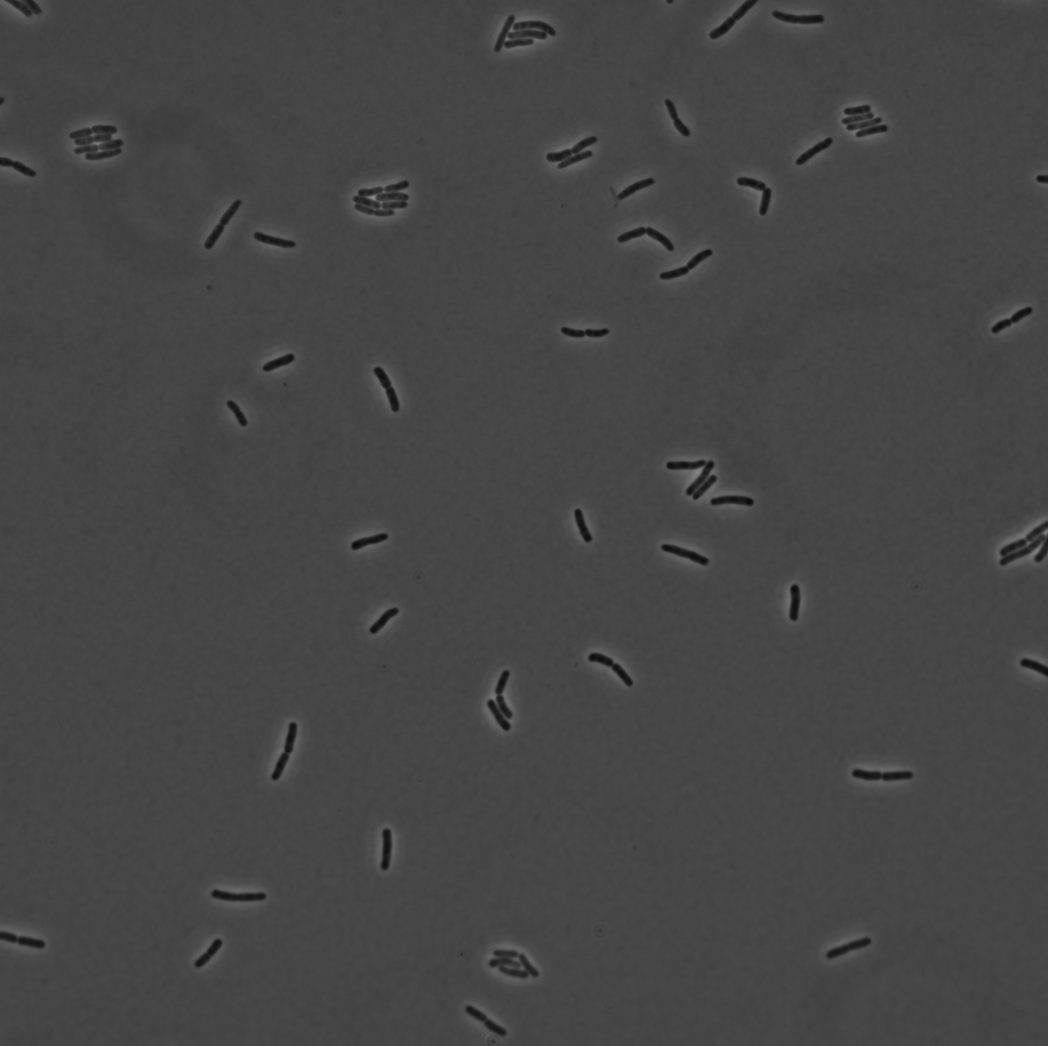

Supplement: Supplementary file 13 — Source data Fig. 1 [file 44321_2025_219_MOESM13_ESM.zip › 1Ai/mg1655 mu 0 percent saccharinc1c1t01.tif]

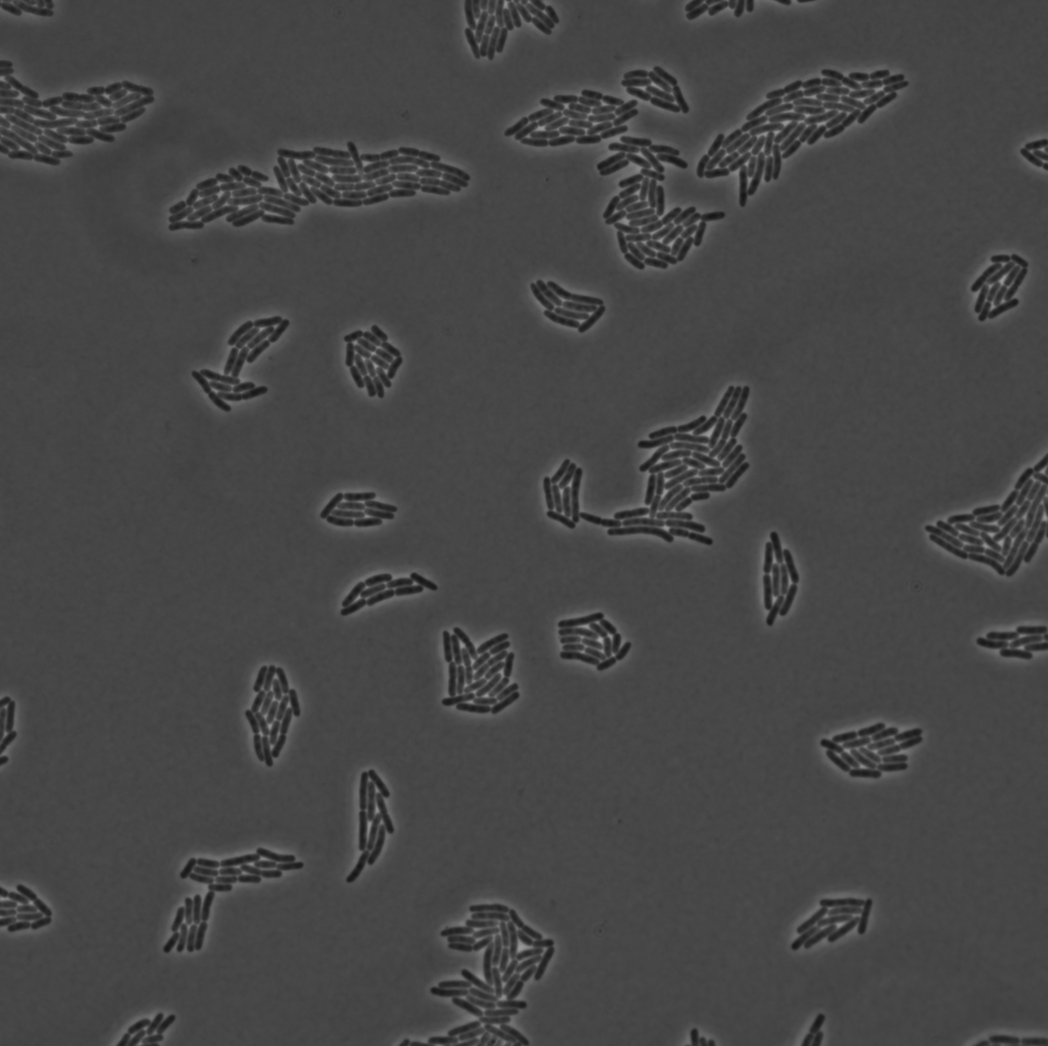

Supplement: Supplementary file 13 — Source data Fig. 1 [file 44321_2025_219_MOESM13_ESM.zip › 1Ai/mg1655 mu 0 percent saccharinc1c1t17.tif]

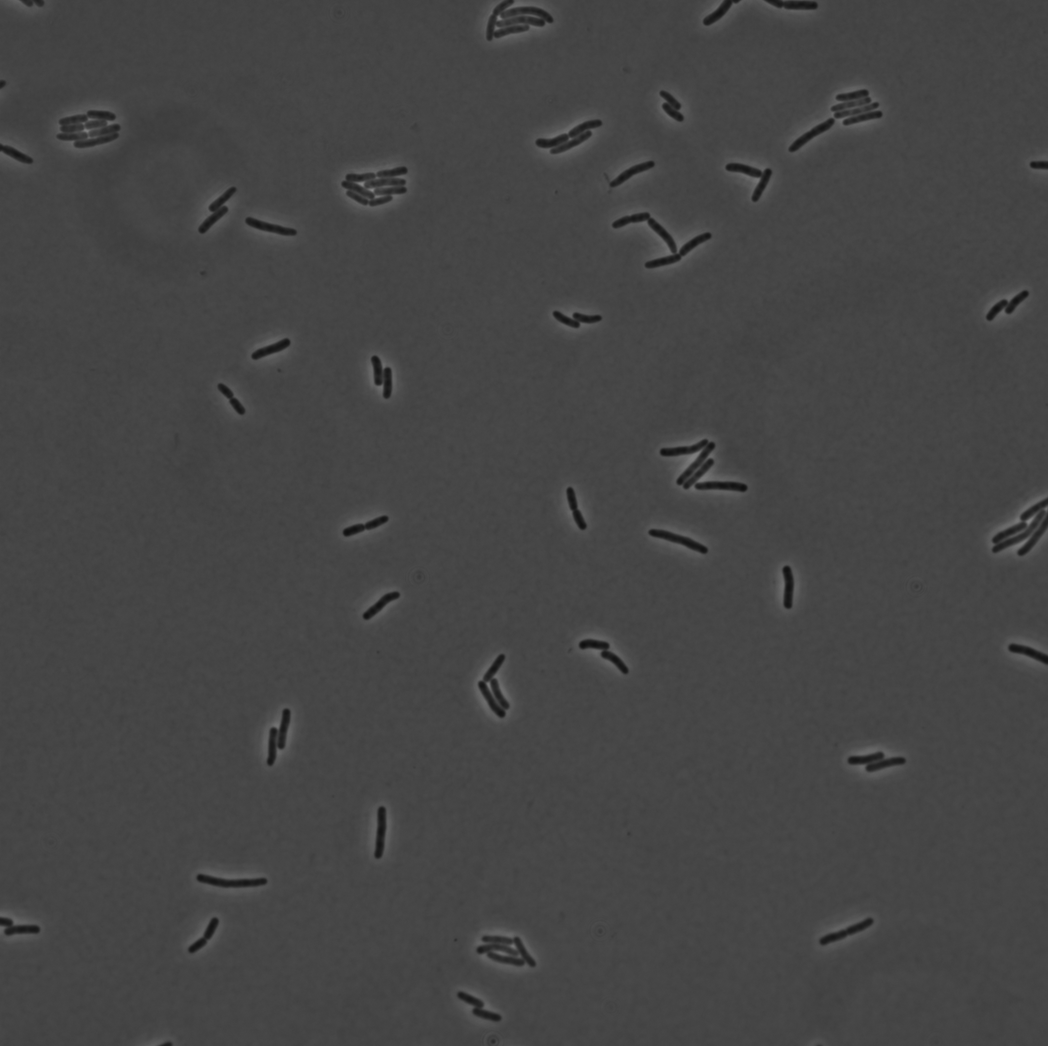

Supplement: Supplementary file 13 — Source data Fig. 1 [file 44321_2025_219_MOESM13_ESM.zip › 1Ai/mg1655 mu 0 percent saccharinc1c1t03.tif]

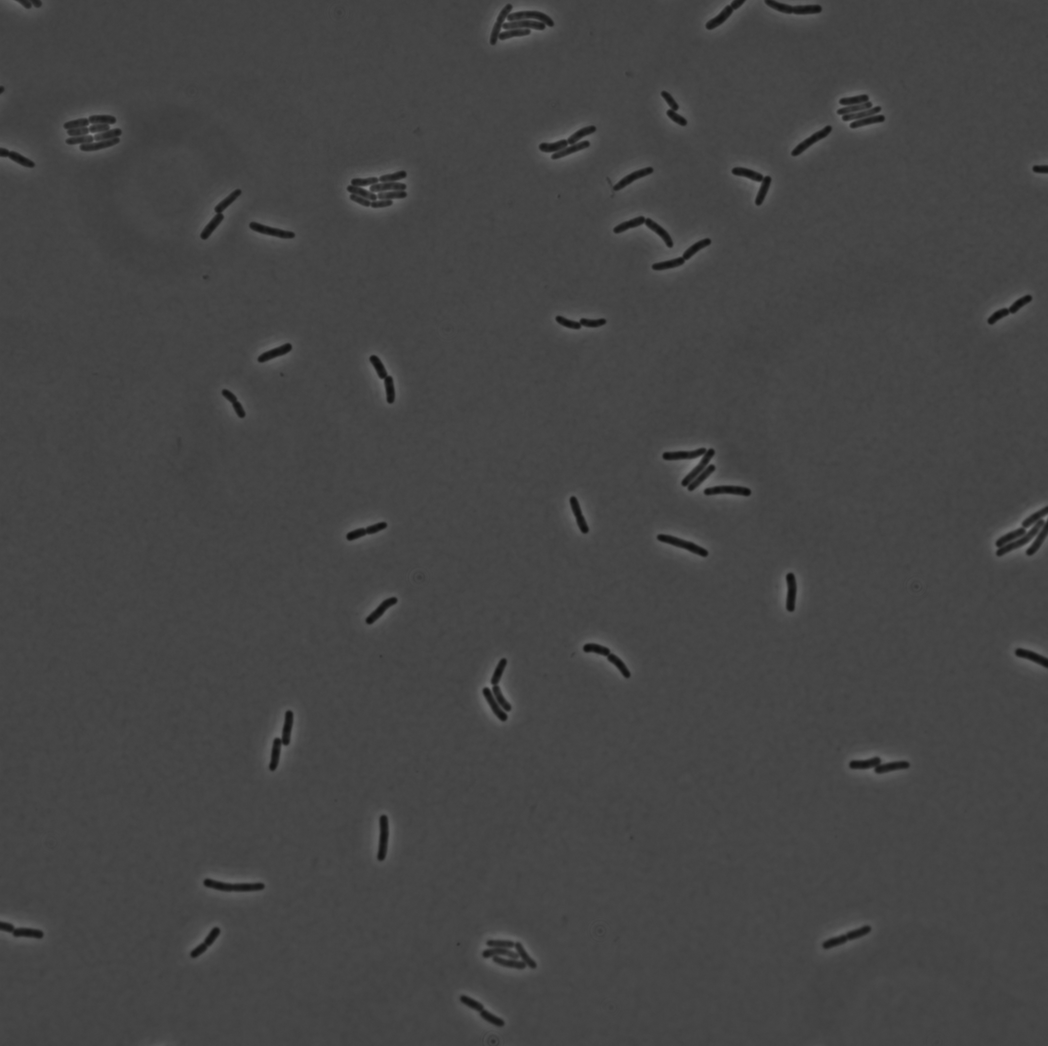

Supplement: Supplementary file 13 — Source data Fig. 1 [file 44321_2025_219_MOESM13_ESM.zip › 1Ai/mg1655 mu 0 percent saccharinc1c1t02.tif]

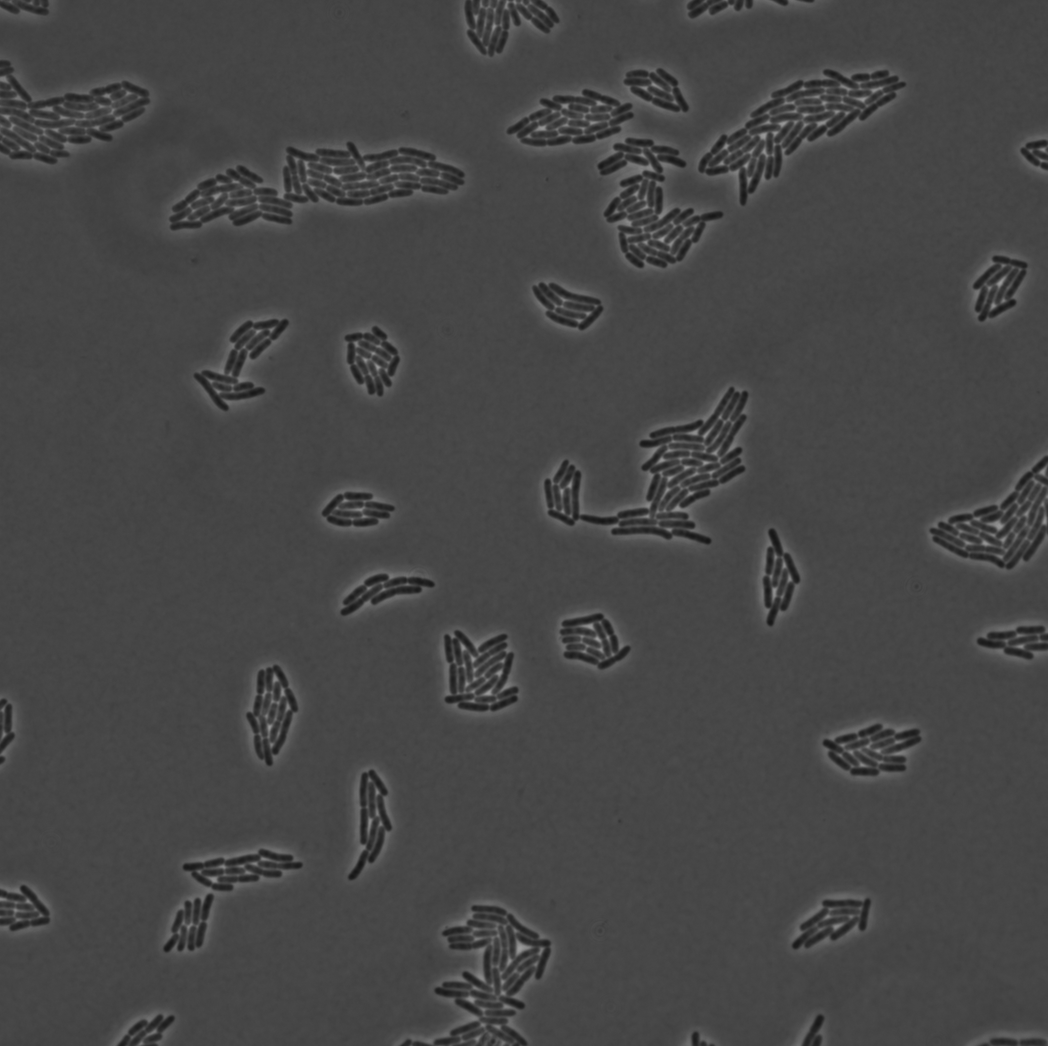

Supplement: Supplementary file 13 — Source data Fig. 1 [file 44321_2025_219_MOESM13_ESM.zip › 1Ai/mg1655 mu 0 percent saccharinc1c1t16.tif]

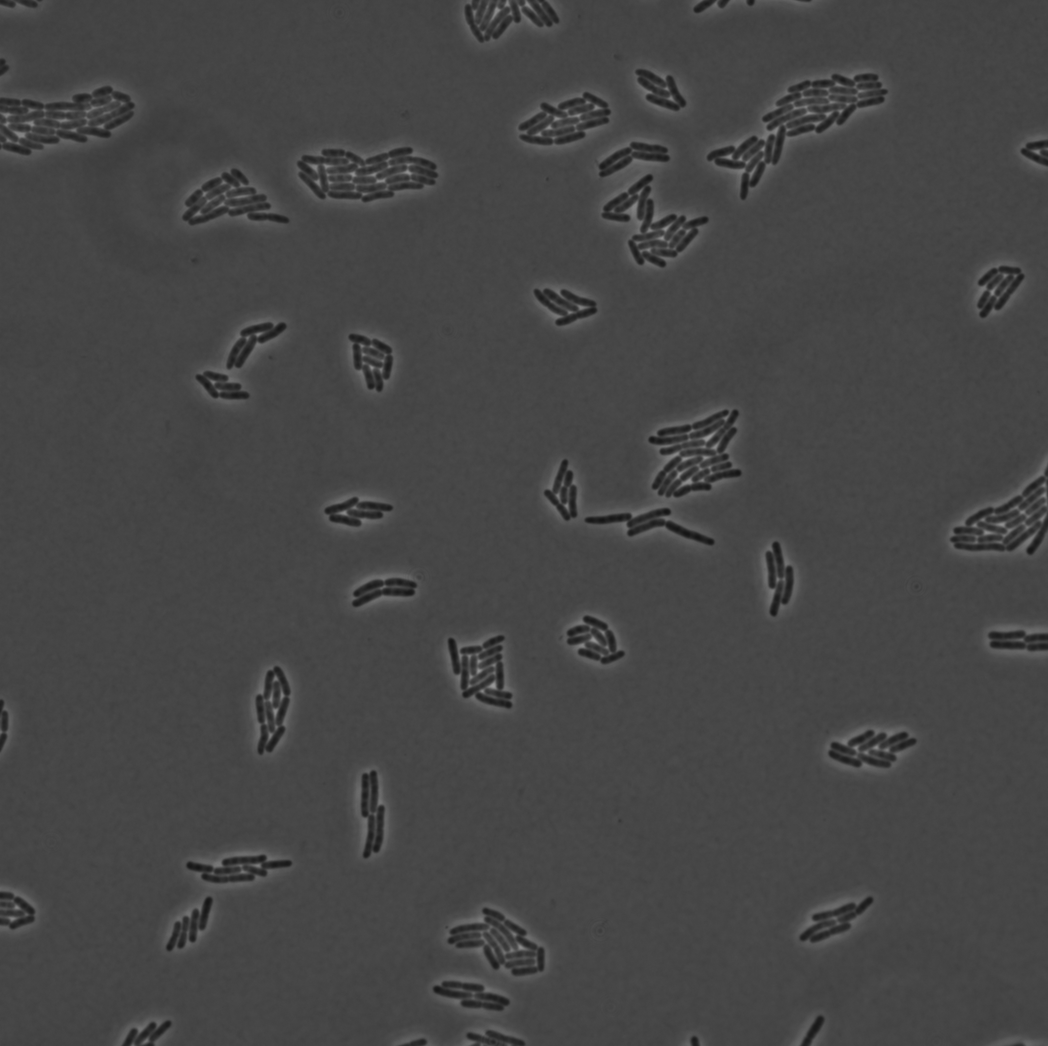

Supplement: Supplementary file 13 — Source data Fig. 1 [file 44321_2025_219_MOESM13_ESM.zip › 1Ai/mg1655 mu 0 percent saccharinc1c1t12.tif]

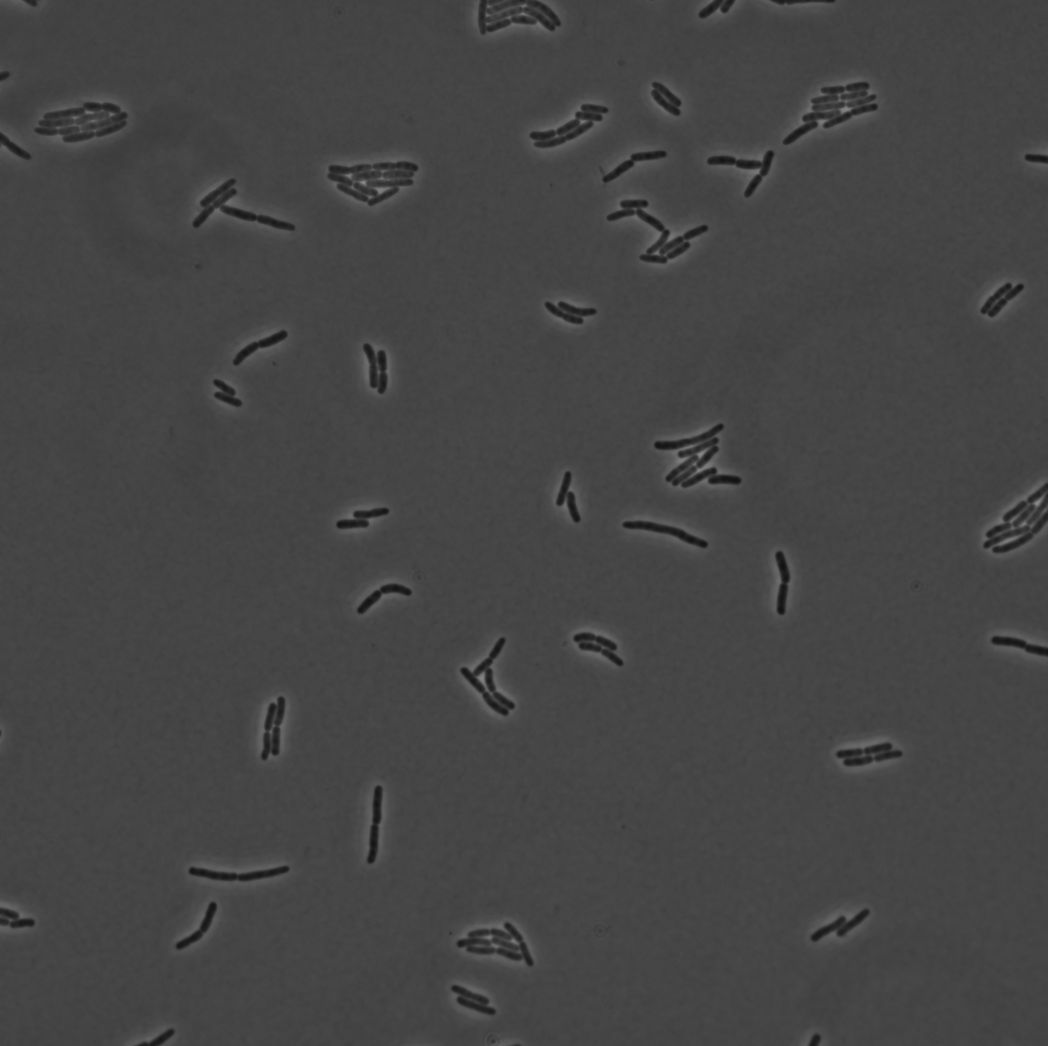

Supplement: Supplementary file 13 — Source data Fig. 1 [file 44321_2025_219_MOESM13_ESM.zip › 1Ai/mg1655 mu 0 percent saccharinc1c1t06.tif]

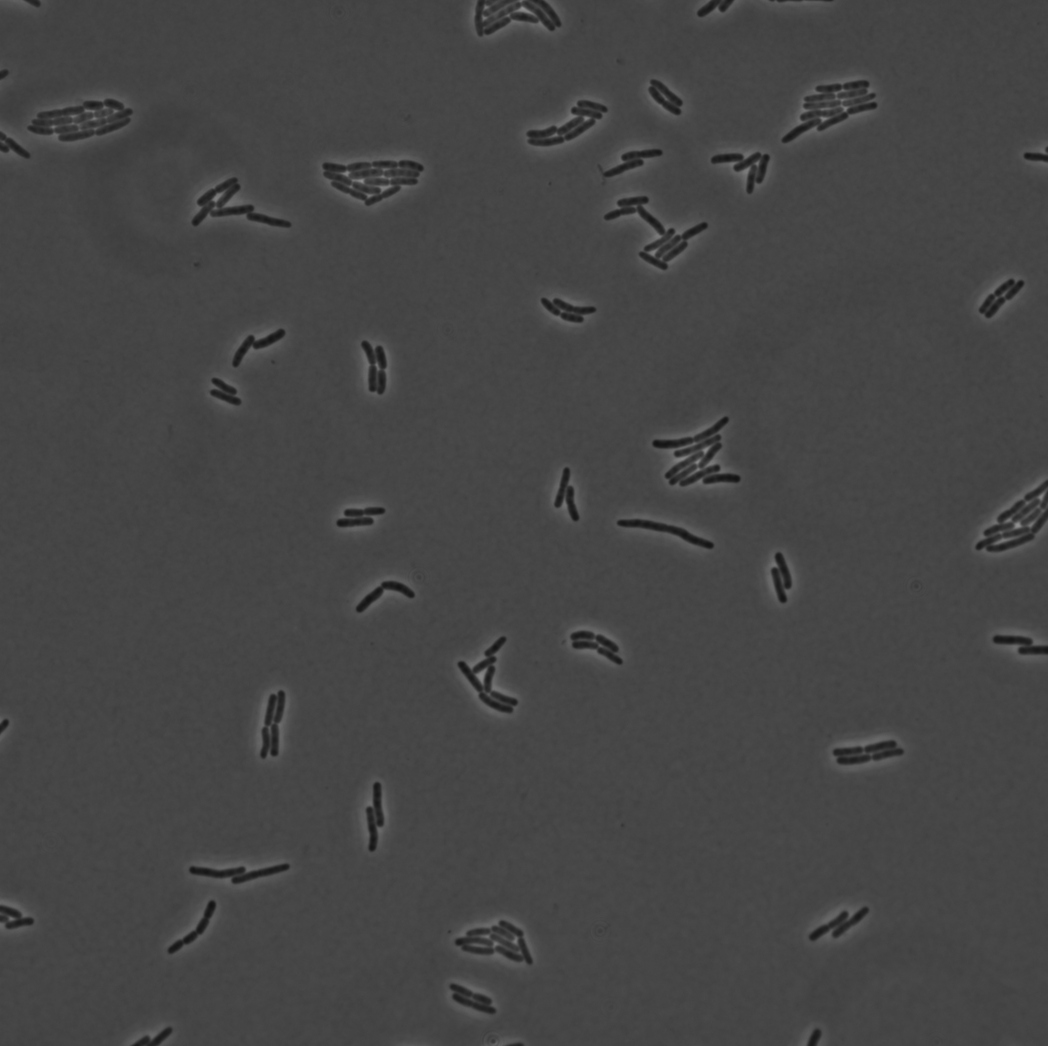

Supplement: Supplementary file 13 — Source data Fig. 1 [file 44321_2025_219_MOESM13_ESM.zip › 1Ai/mg1655 mu 0 percent saccharinc1c1t07.tif]

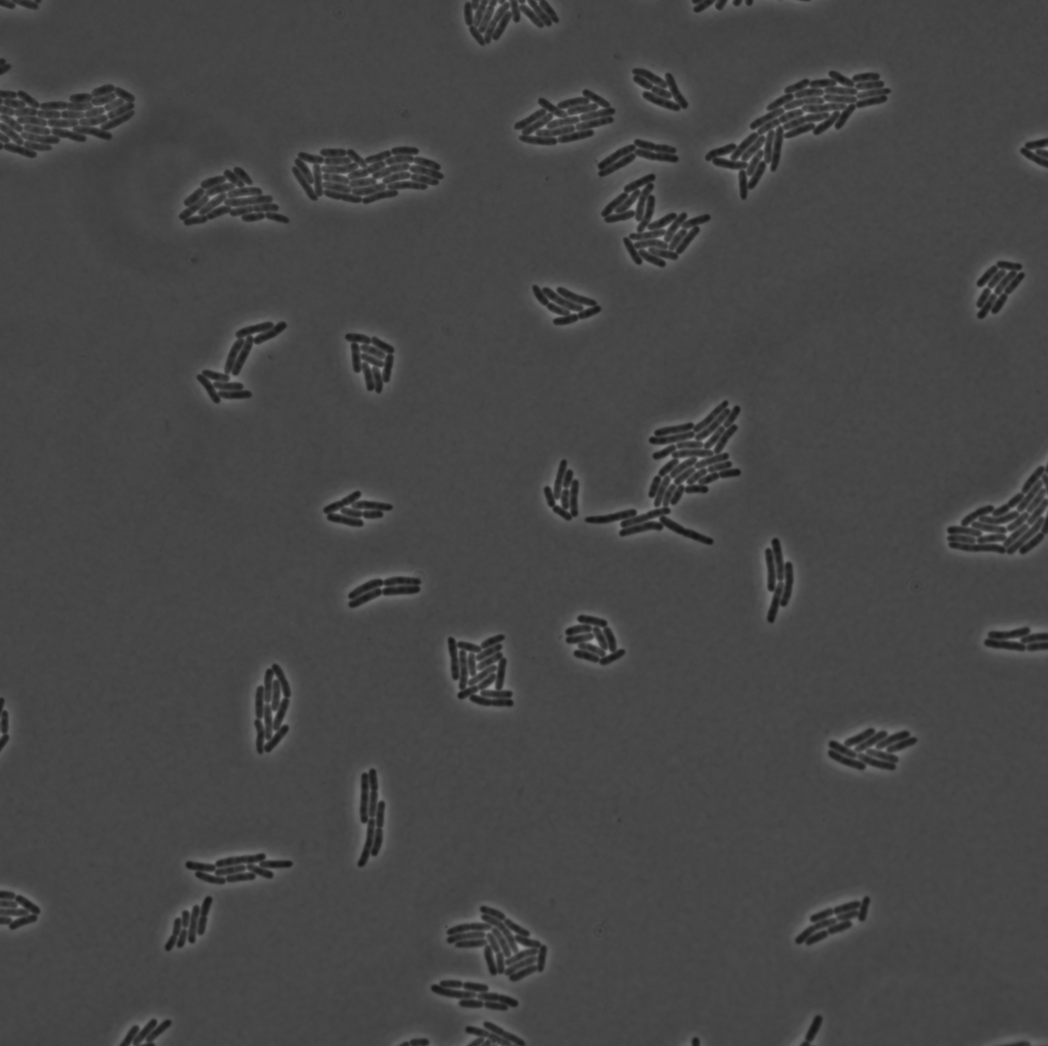

Supplement: Supplementary file 13 — Source data Fig. 1 [file 44321_2025_219_MOESM13_ESM.zip › 1Ai/mg1655 mu 0 percent saccharinc1c1t13.tif]

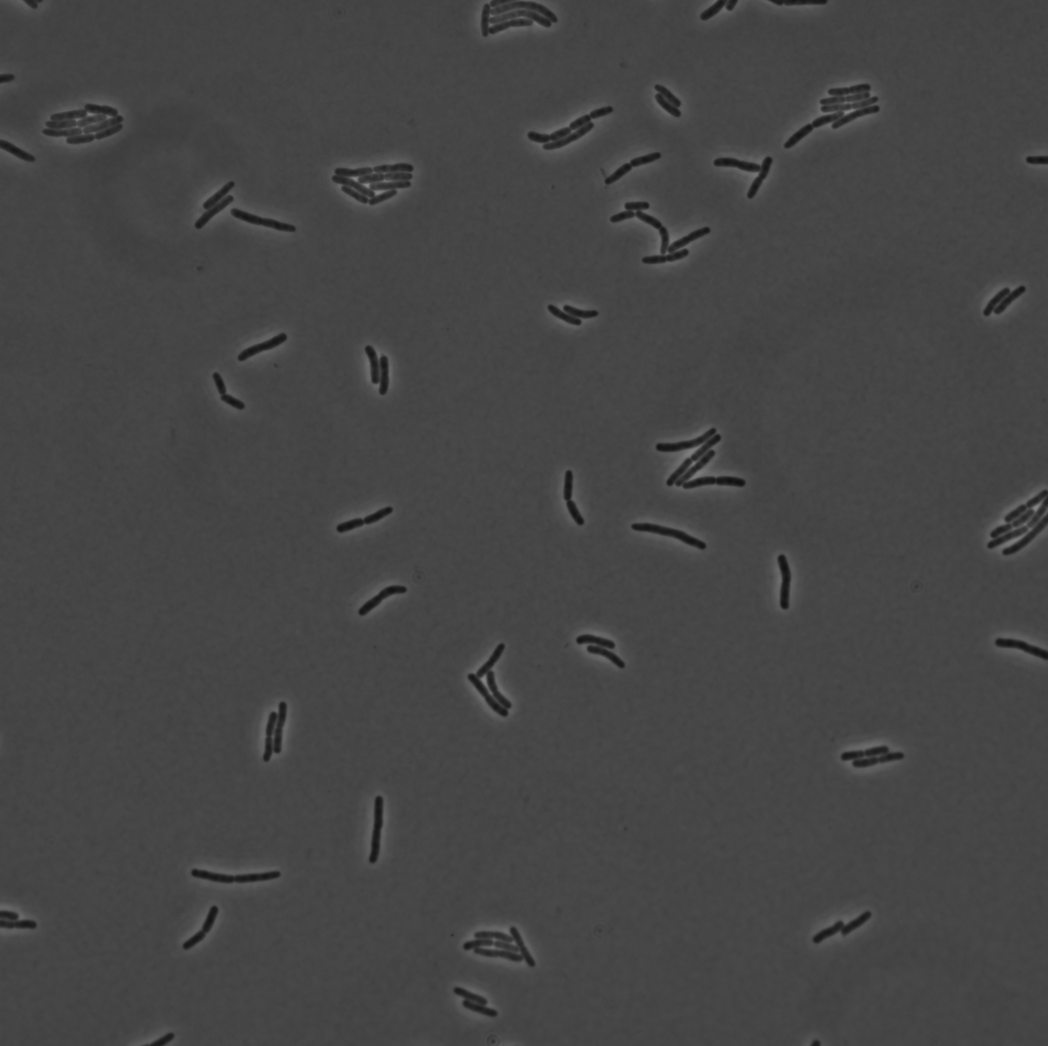

Supplement: Supplementary file 13 — Source data Fig. 1 [file 44321_2025_219_MOESM13_ESM.zip › 1Ai/mg1655 mu 0 percent saccharinc1c1t05.tif]

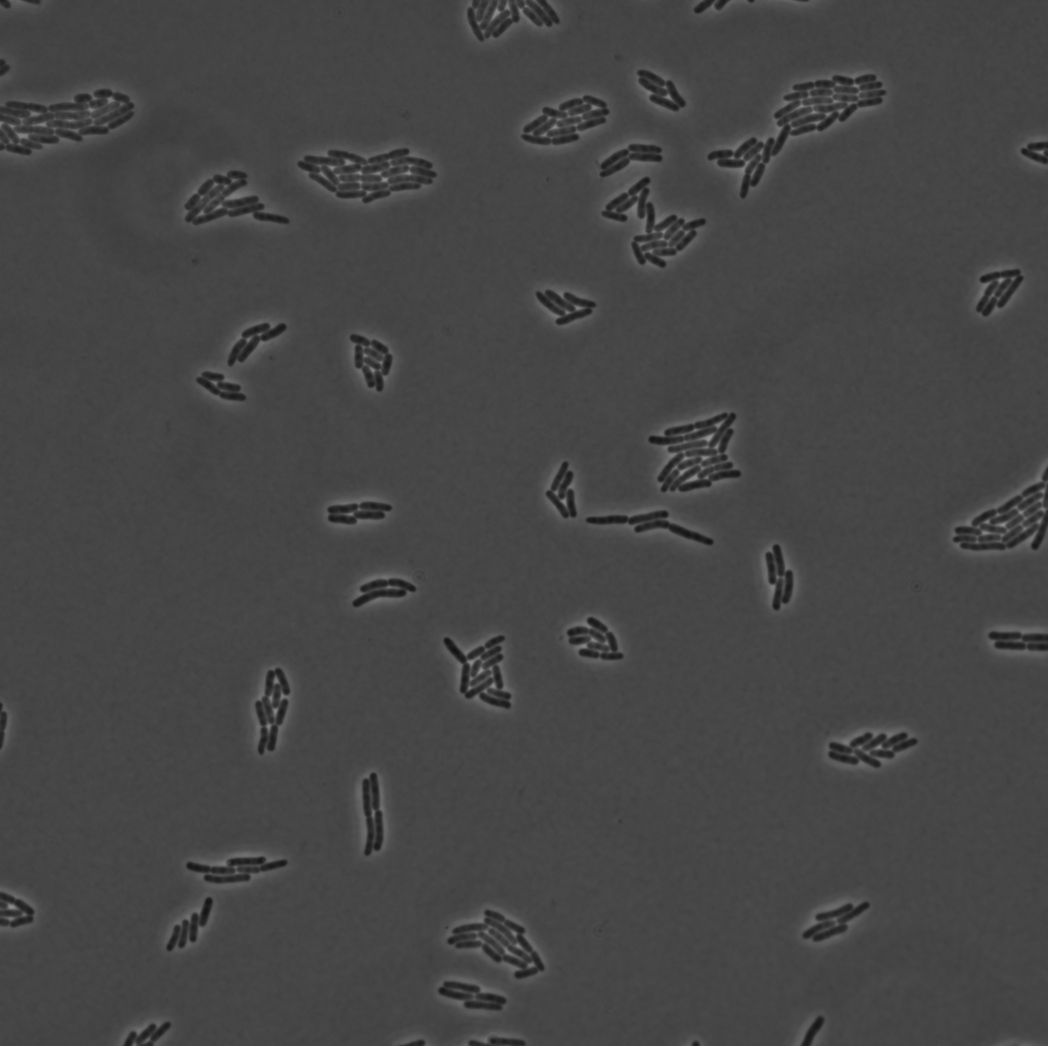

Supplement: Supplementary file 13 — Source data Fig. 1 [file 44321_2025_219_MOESM13_ESM.zip › 1Ai/mg1655 mu 0 percent saccharinc1c1t11.tif]

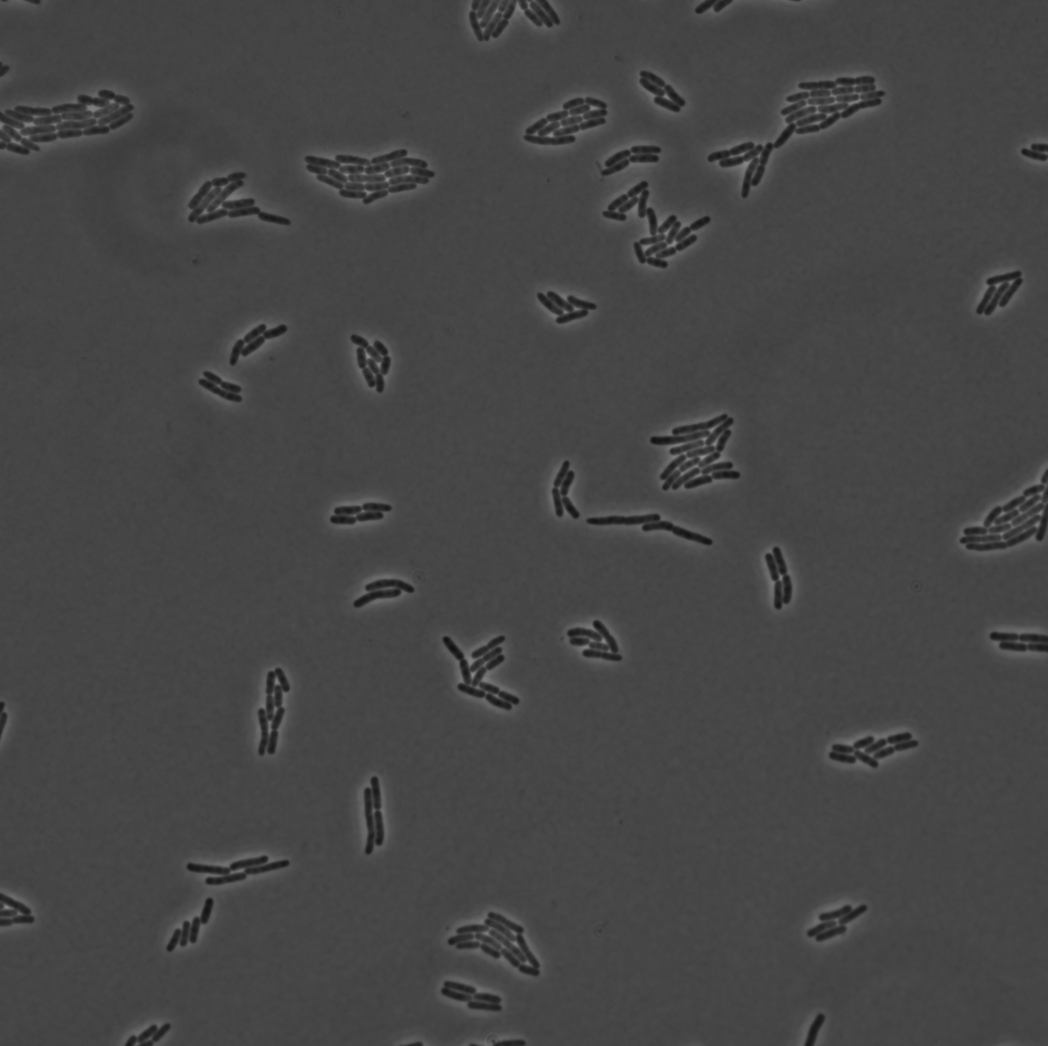

Supplement: Supplementary file 13 — Source data Fig. 1 [file 44321_2025_219_MOESM13_ESM.zip › 1Ai/mg1655 mu 0 percent saccharinc1c1t10.tif]

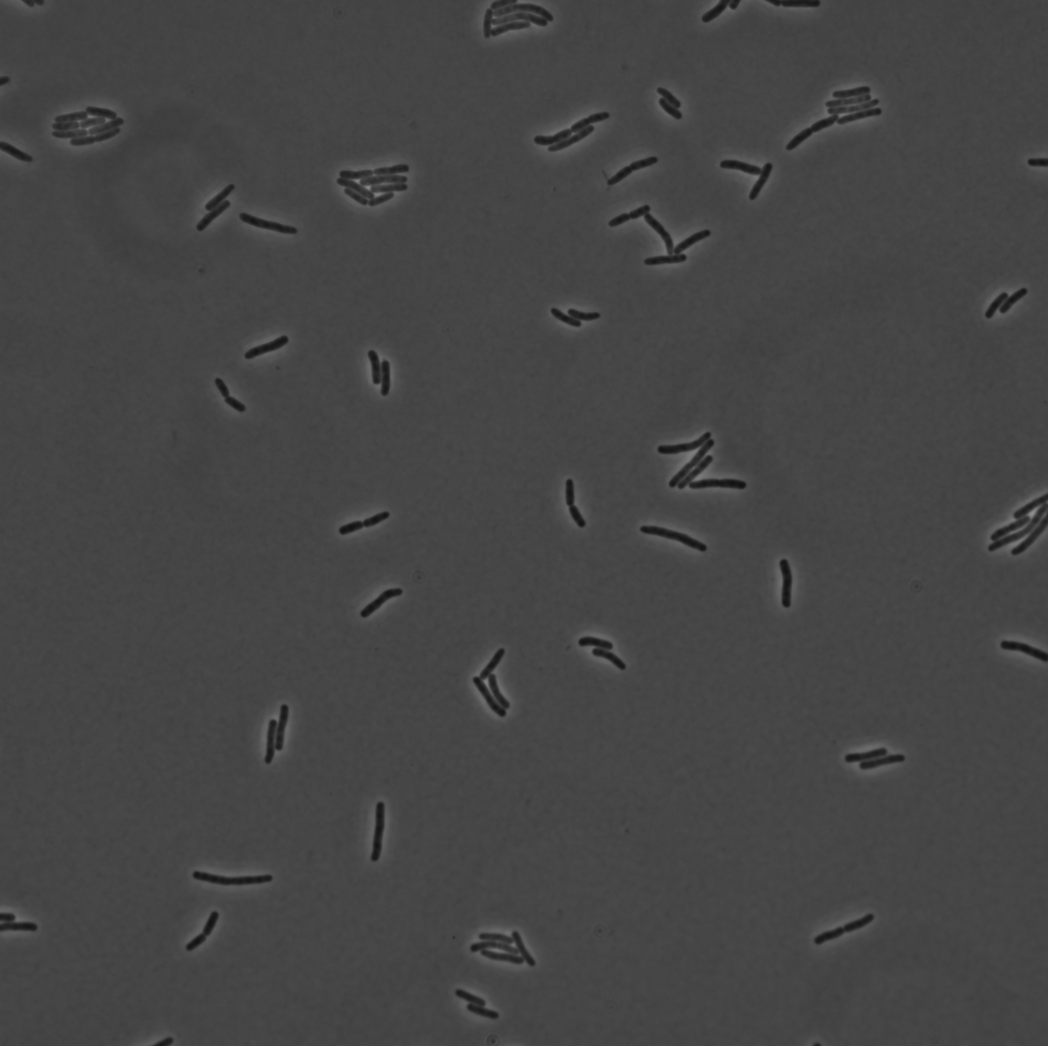

Supplement: Supplementary file 13 — Source data Fig. 1 [file 44321_2025_219_MOESM13_ESM.zip › 1Ai/mg1655 mu 0 percent saccharinc1c1t04.tif]

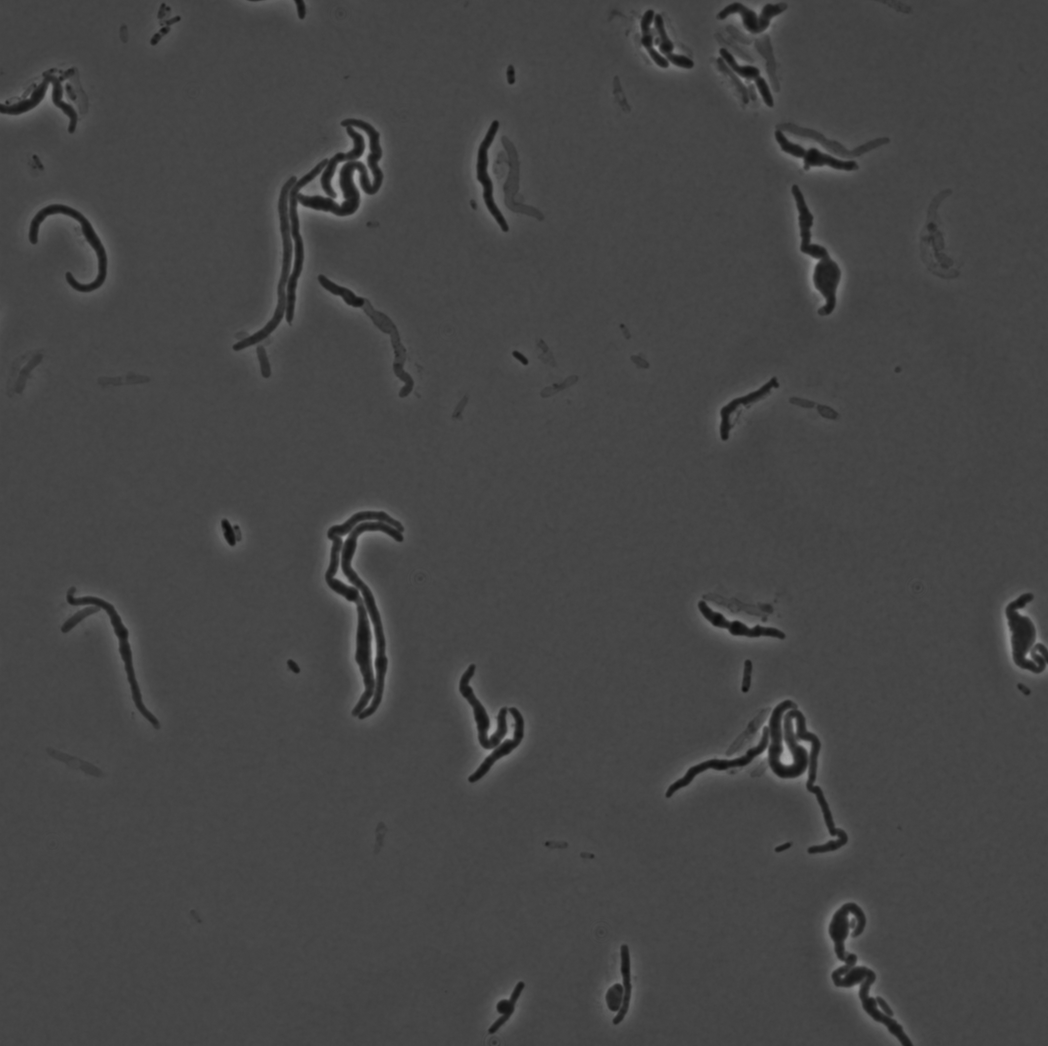

Supplement: Supplementary file 13 — Source data Fig. 1 [file 44321_2025_219_MOESM13_ESM.zip › 1Aii/rce849 mu 1_4 percent saccharin002c1t25.tif]

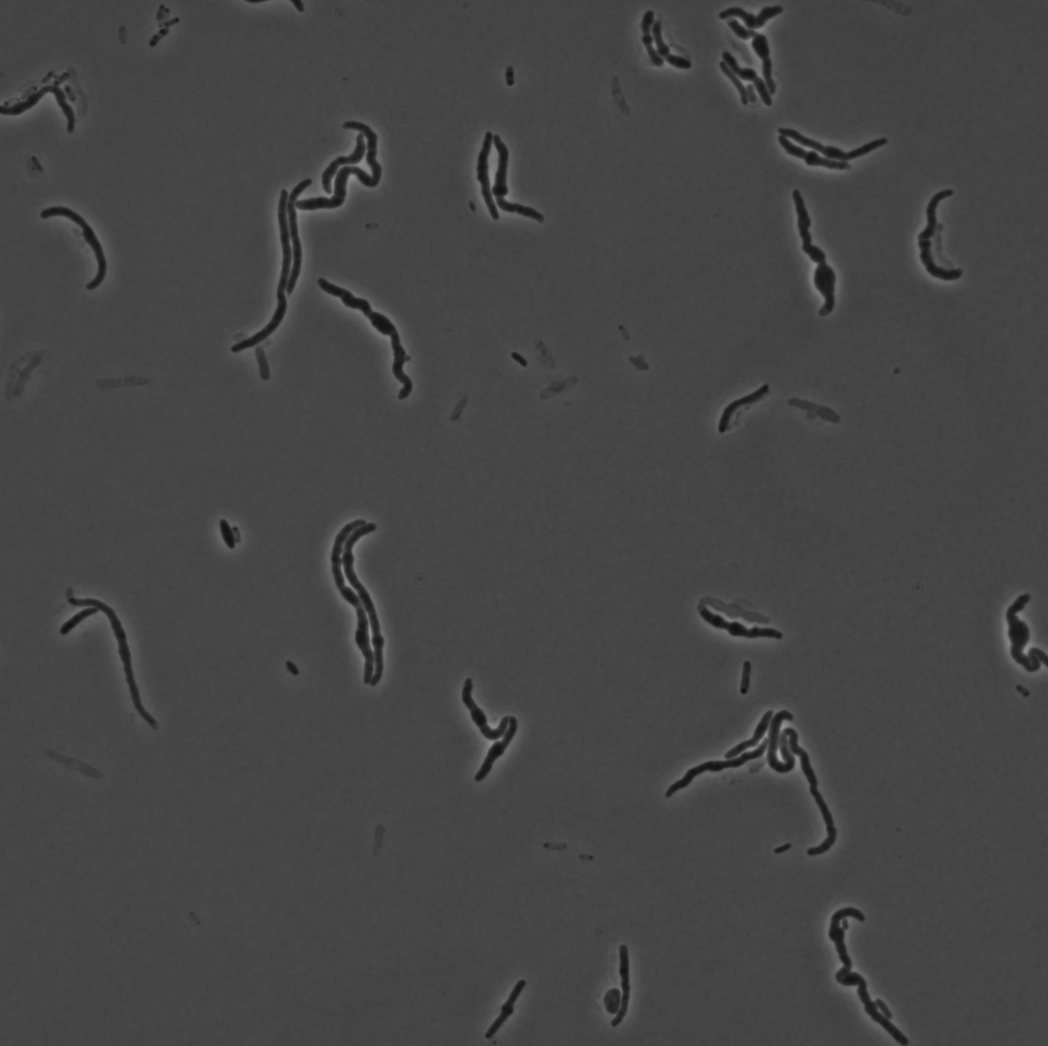

Supplement: Supplementary file 13 — Source data Fig. 1 [file 44321_2025_219_MOESM13_ESM.zip › 1Aii/rce849 mu 1_4 percent saccharin002c1t19.tif]

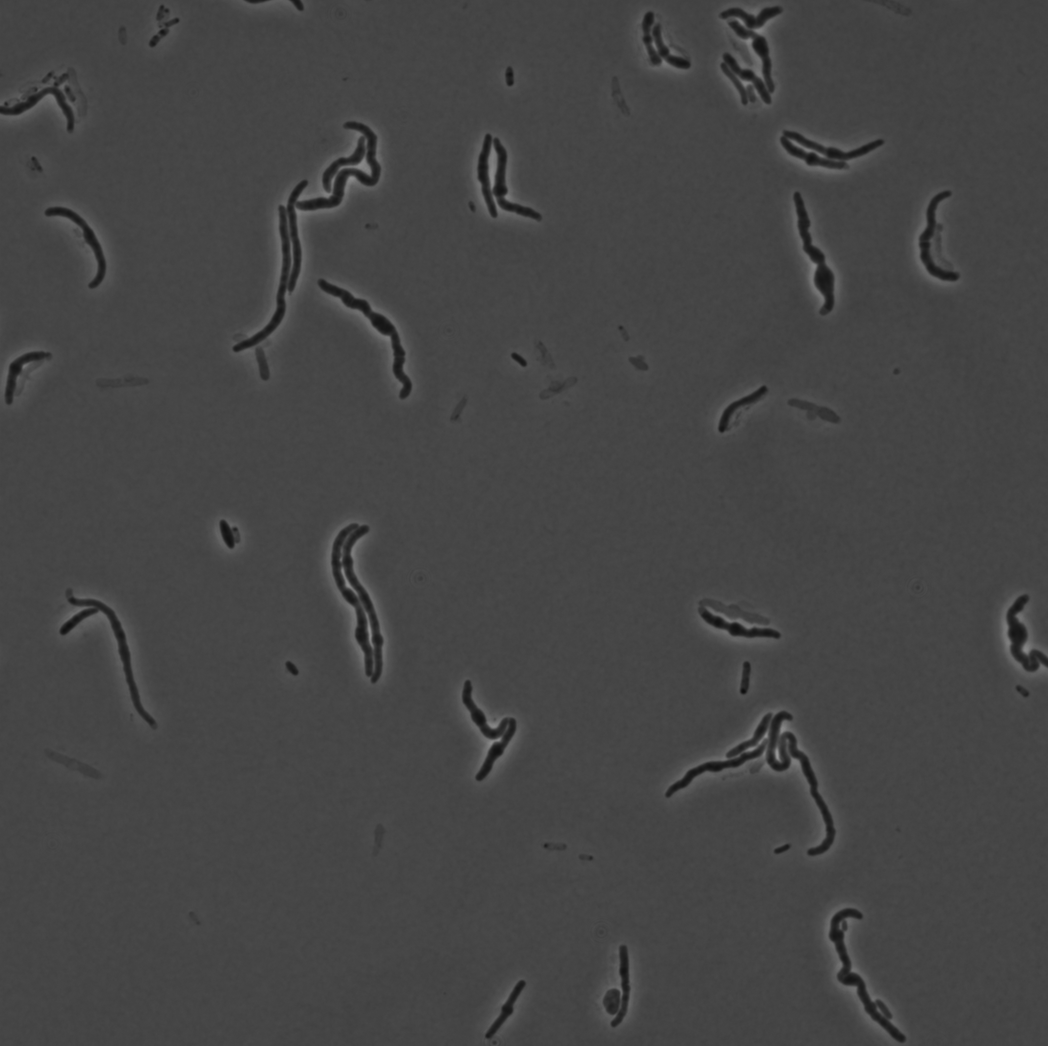

Supplement: Supplementary file 13 — Source data Fig. 1 [file 44321_2025_219_MOESM13_ESM.zip › 1Aii/rce849 mu 1_4 percent saccharin002c1t18.tif]

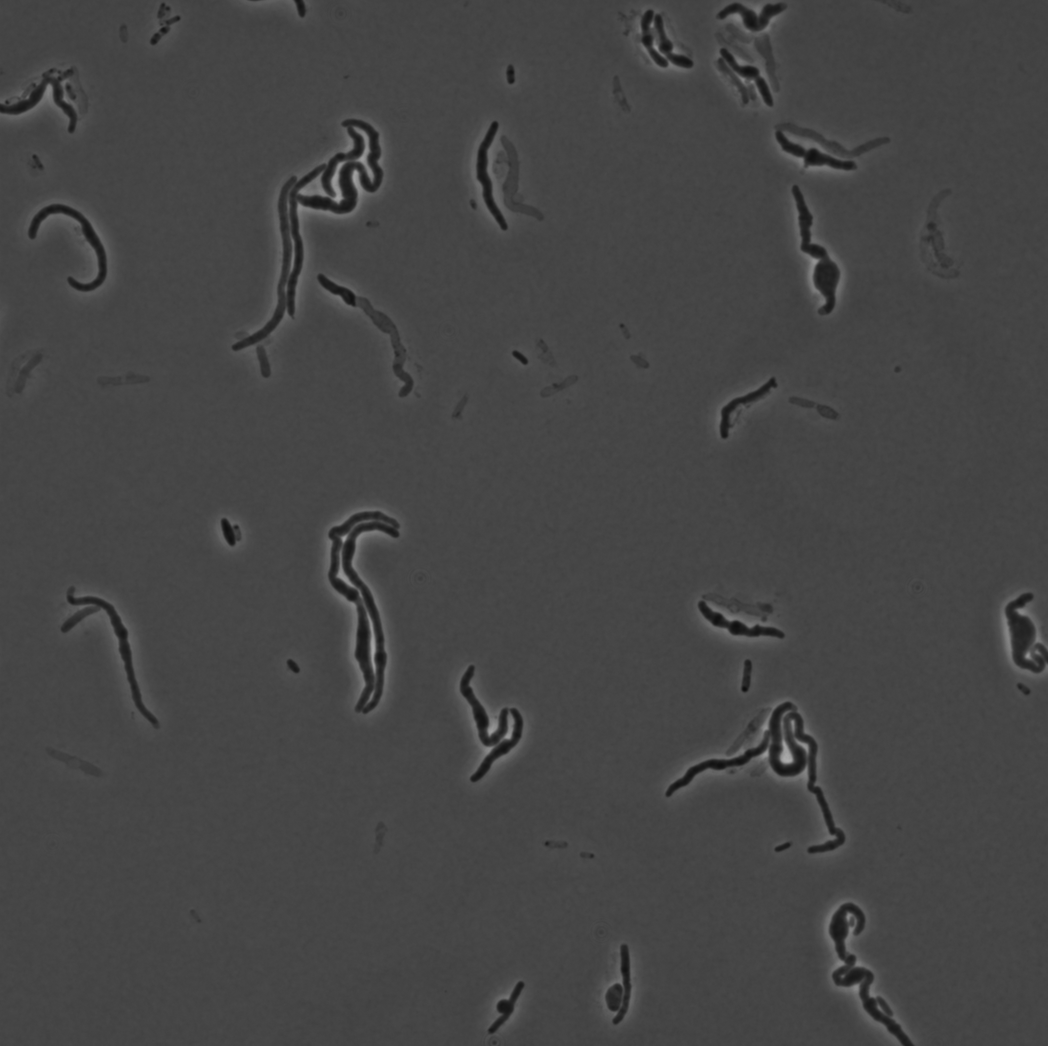

Supplement: Supplementary file 13 — Source data Fig. 1 [file 44321_2025_219_MOESM13_ESM.zip › 1Aii/rce849 mu 1_4 percent saccharin002c1t24.tif]

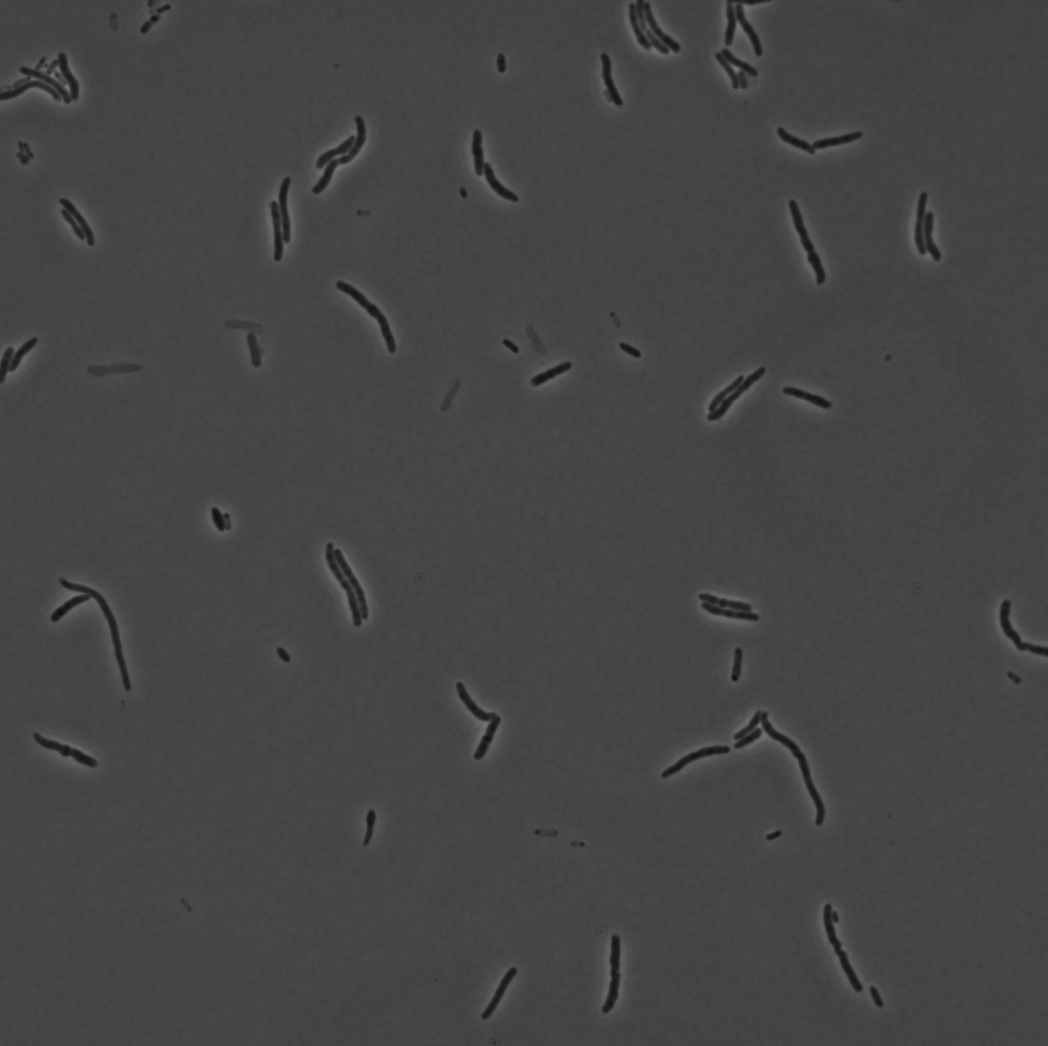

Supplement: Supplementary file 13 — Source data Fig. 1 [file 44321_2025_219_MOESM13_ESM.zip › 1Aii/rce849 mu 1_4 percent saccharin002c1t08.tif]

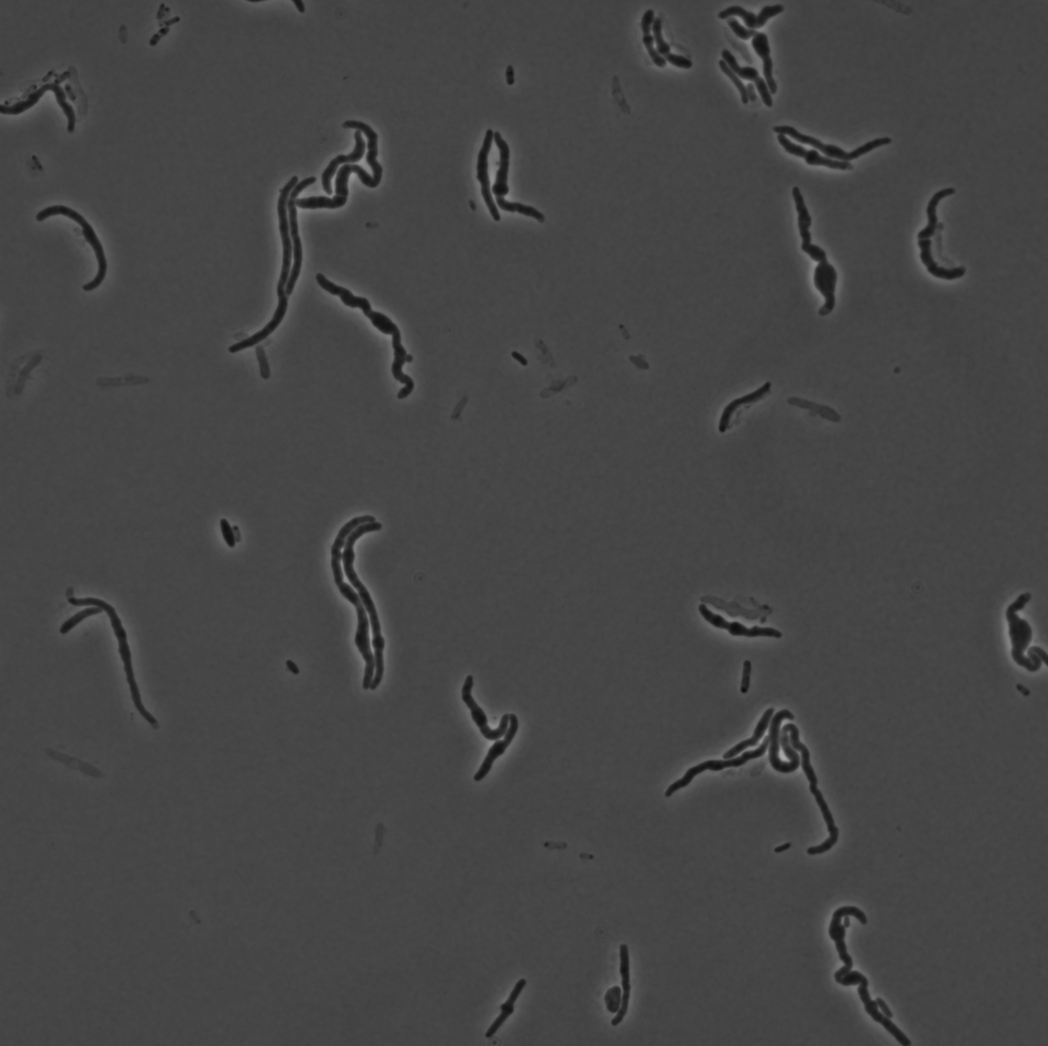

Supplement: Supplementary file 13 — Source data Fig. 1 [file 44321_2025_219_MOESM13_ESM.zip › 1Aii/rce849 mu 1_4 percent saccharin002c1t20.tif]

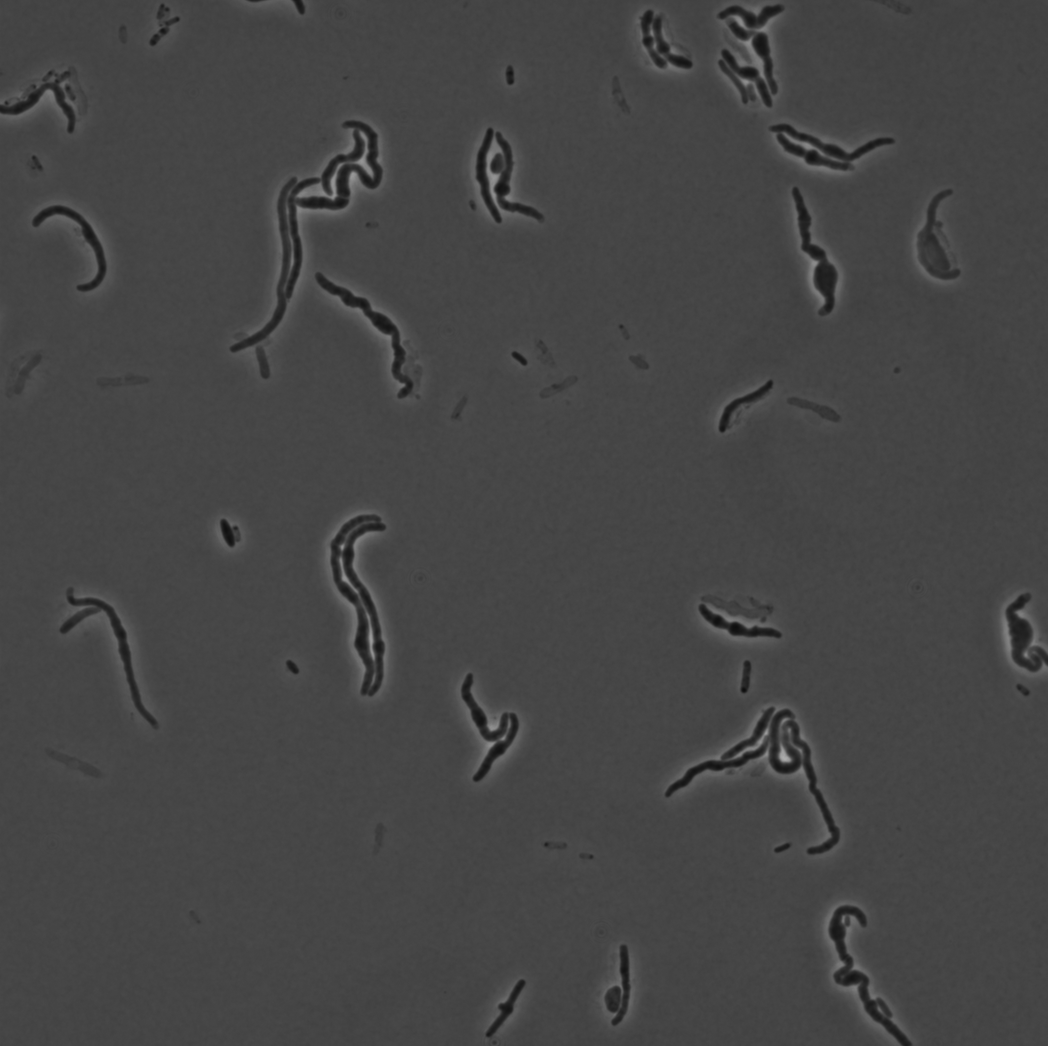

Supplement: Supplementary file 13 — Source data Fig. 1 [file 44321_2025_219_MOESM13_ESM.zip › 1Aii/rce849 mu 1_4 percent saccharin002c1t21.tif]

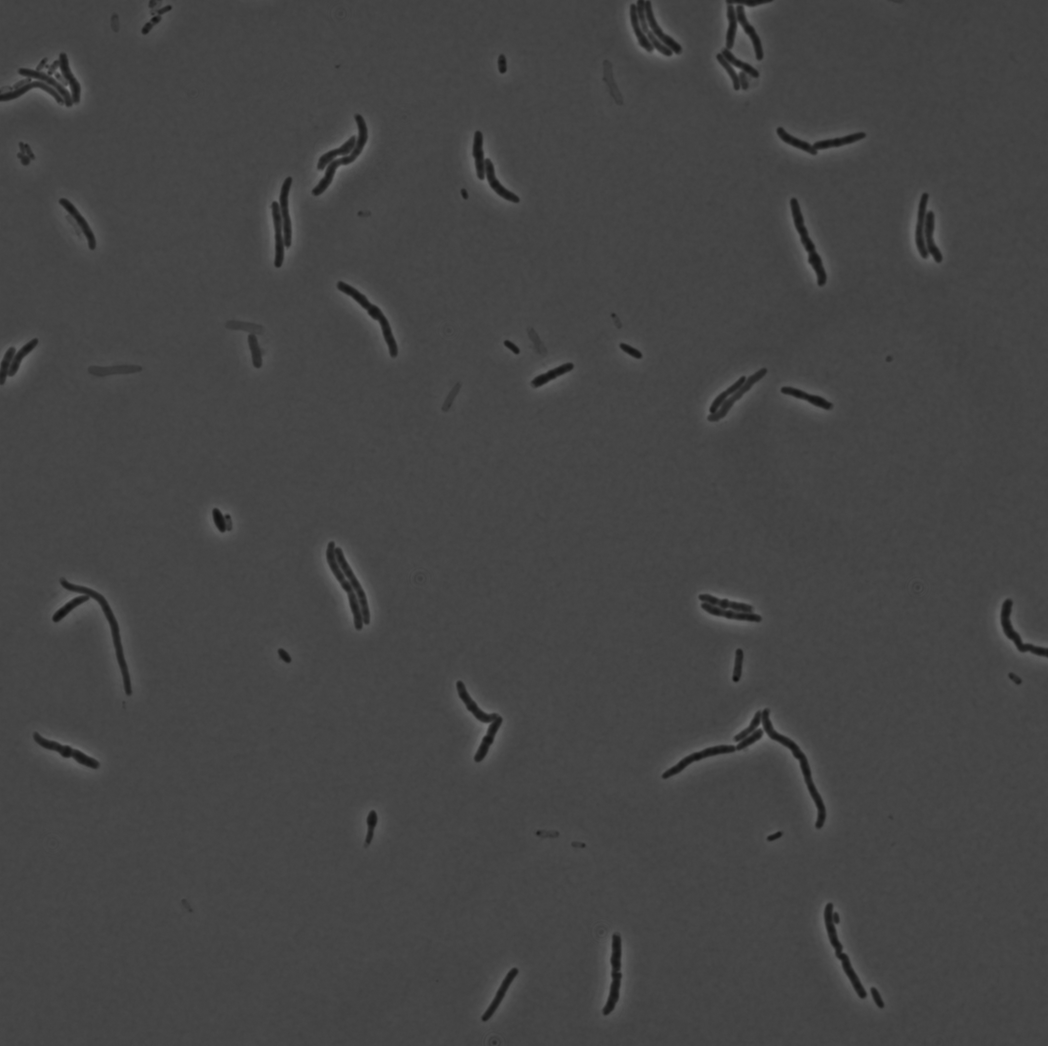

Supplement: Supplementary file 13 — Source data Fig. 1 [file 44321_2025_219_MOESM13_ESM.zip › 1Aii/rce849 mu 1_4 percent saccharin002c1t09.tif]

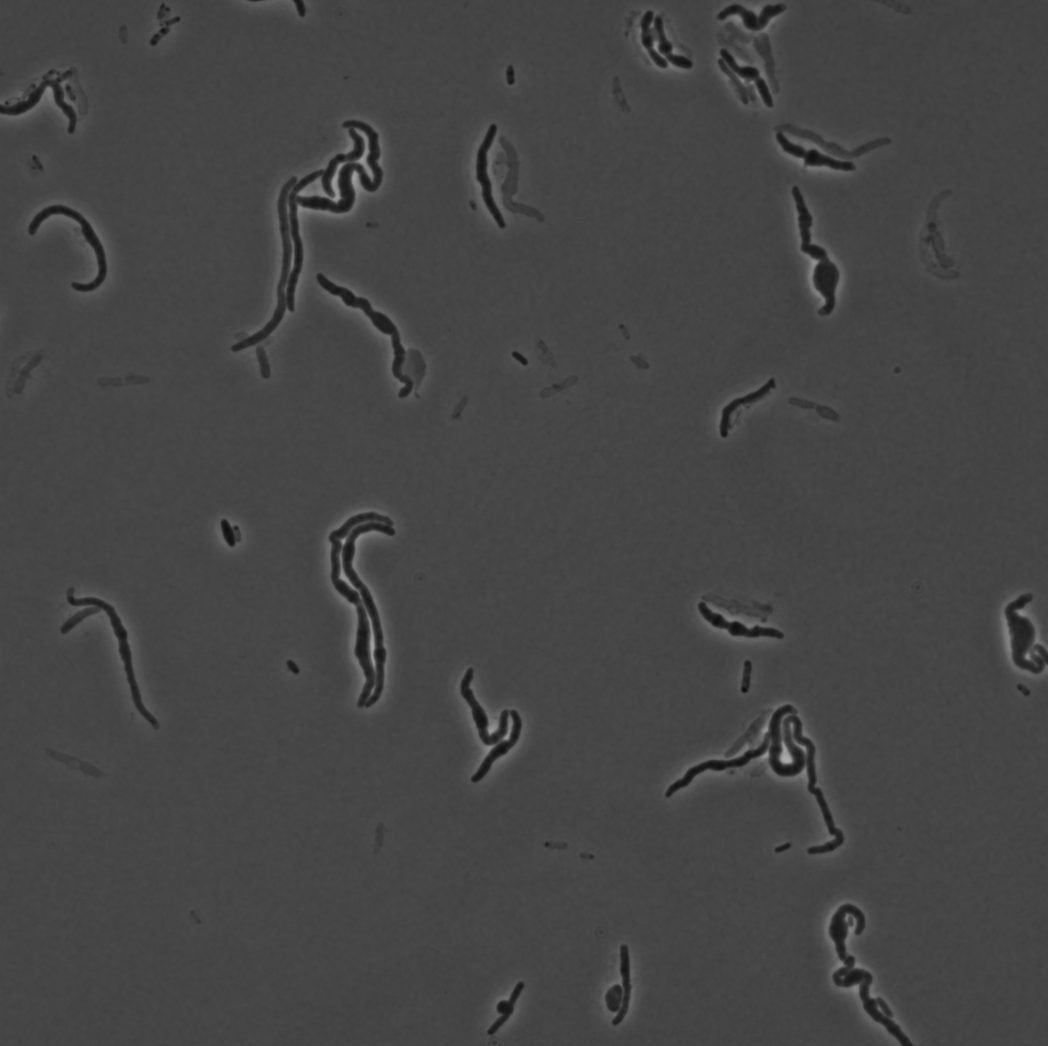

Supplement: Supplementary file 13 — Source data Fig. 1 [file 44321_2025_219_MOESM13_ESM.zip › 1Aii/rce849 mu 1_4 percent saccharin002c1t23.tif]

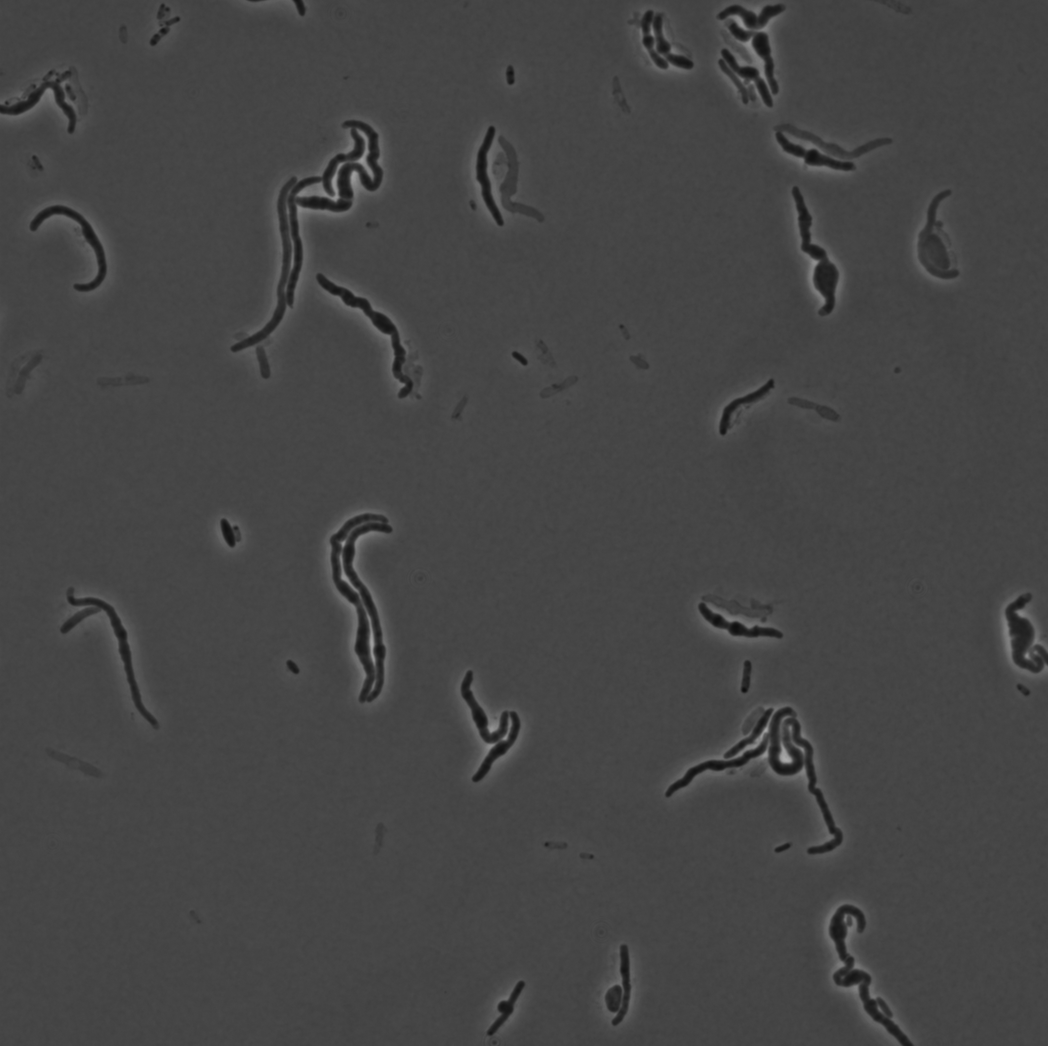

Supplement: Supplementary file 13 — Source data Fig. 1 [file 44321_2025_219_MOESM13_ESM.zip › 1Aii/rce849 mu 1_4 percent saccharin002c1t22.tif]

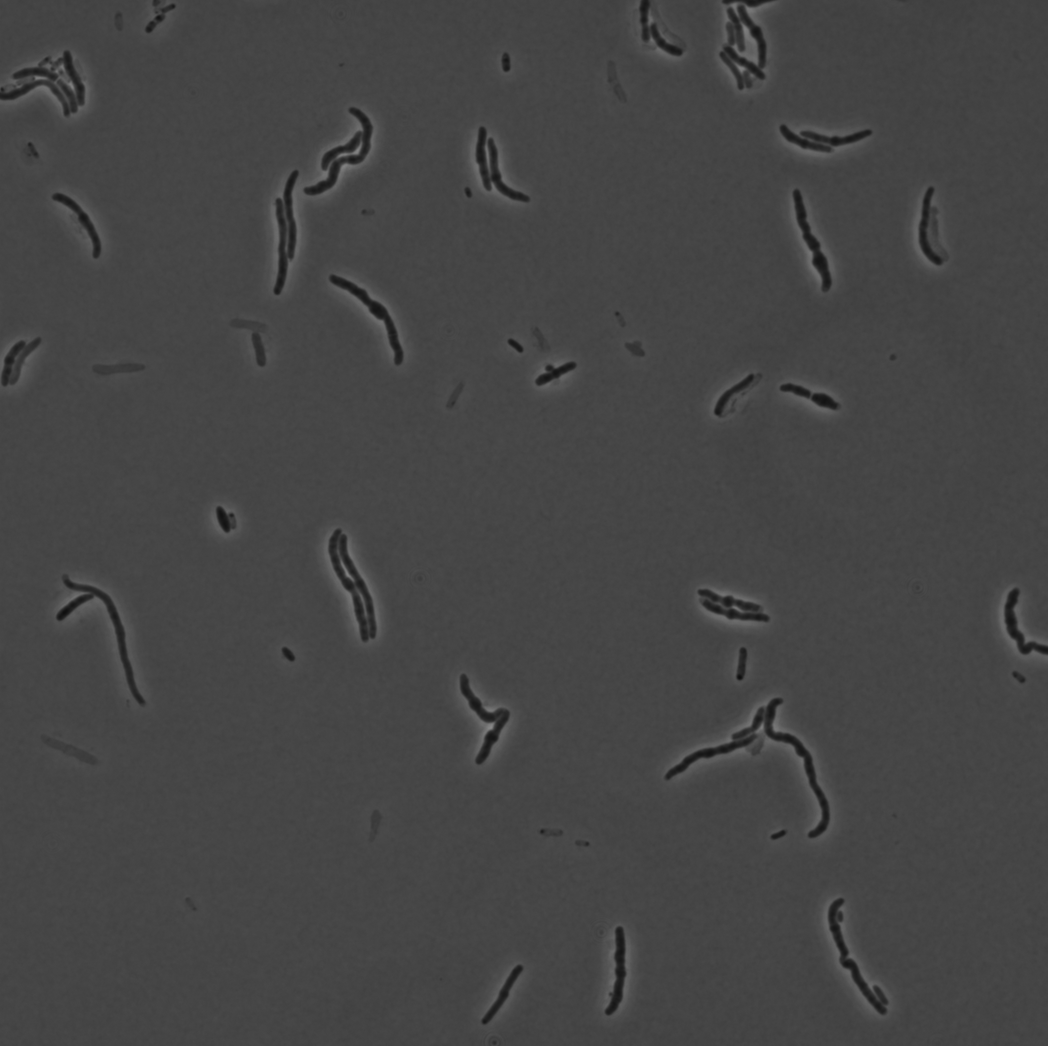

Supplement: Supplementary file 13 — Source data Fig. 1 [file 44321_2025_219_MOESM13_ESM.zip › 1Aii/rce849 mu 1_4 percent saccharin002c1t13.tif]

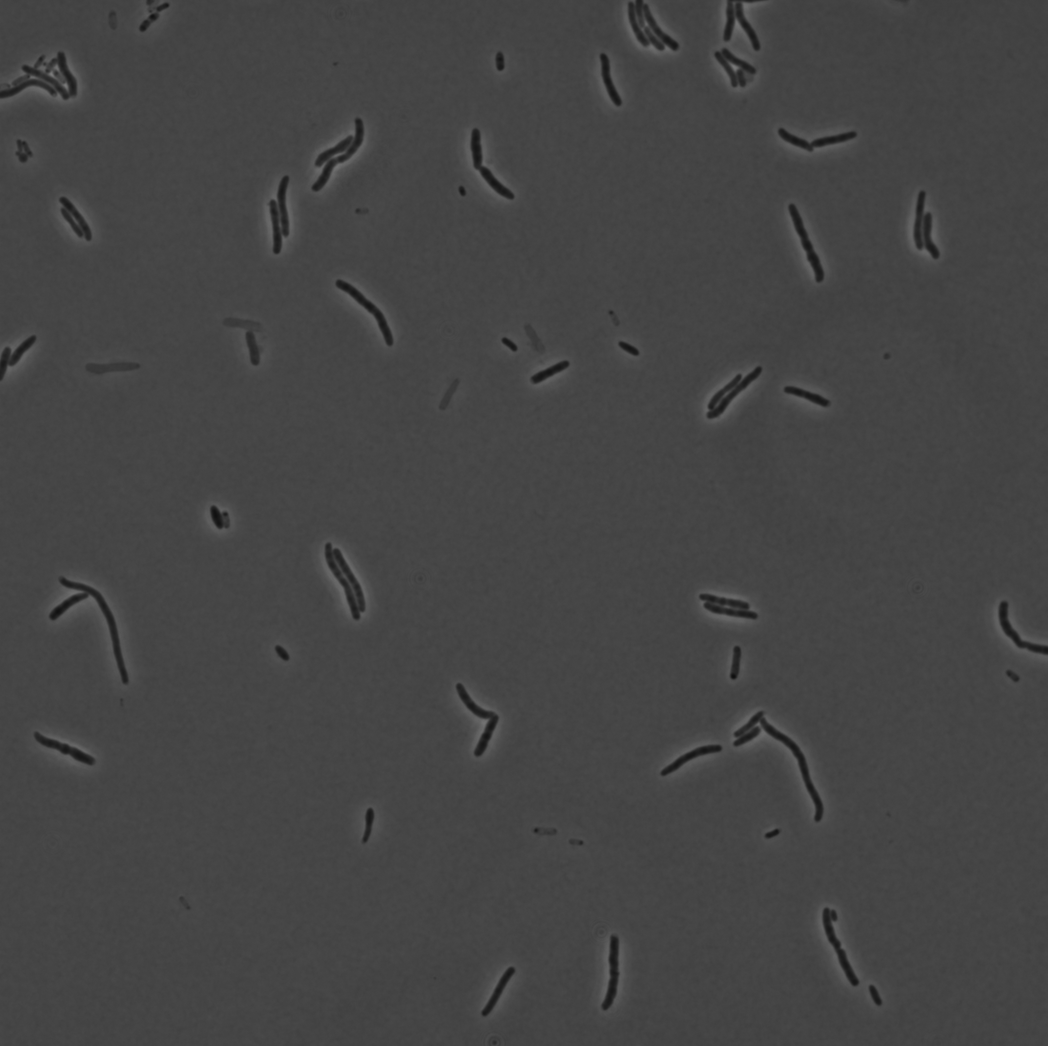

Supplement: Supplementary file 13 — Source data Fig. 1 [file 44321_2025_219_MOESM13_ESM.zip › 1Aii/rce849 mu 1_4 percent saccharin002c1t07.tif]

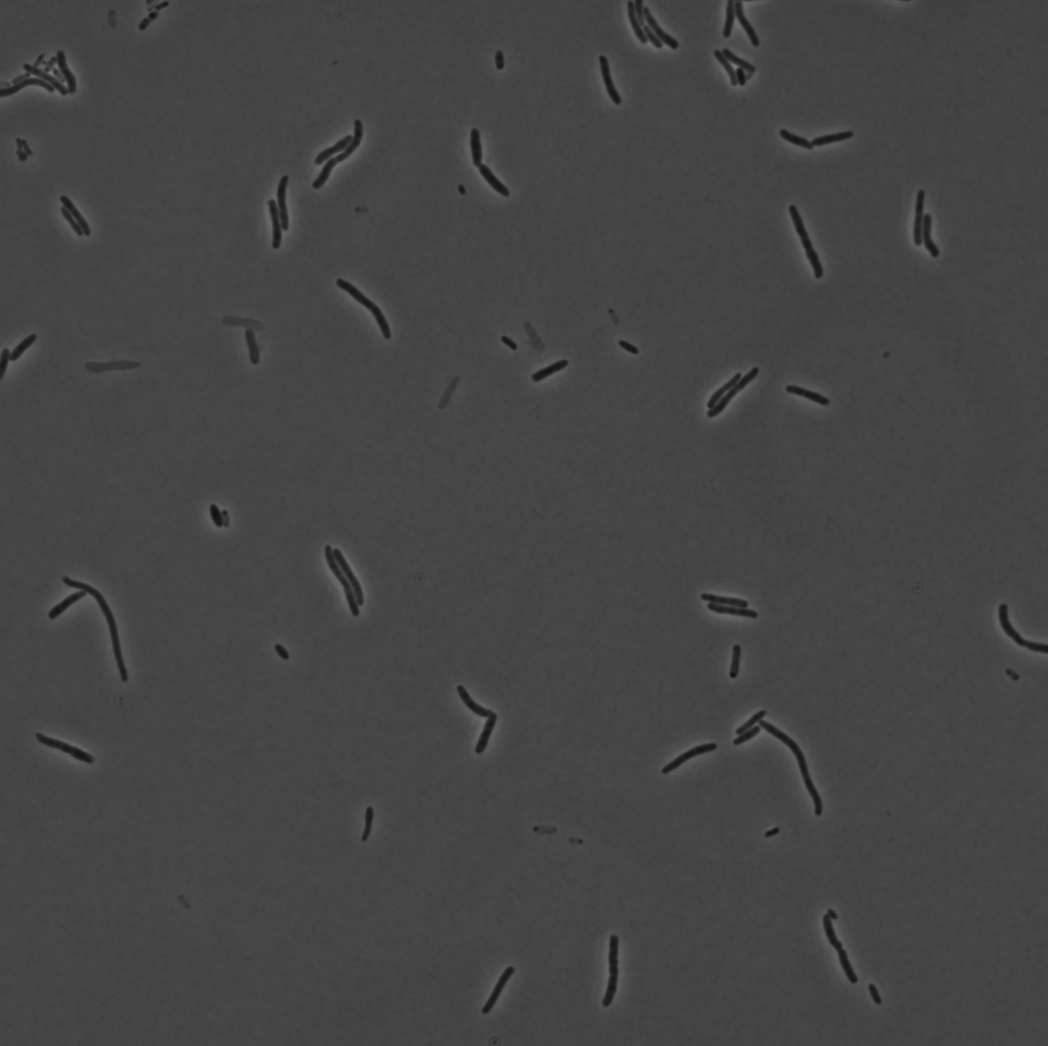

Supplement: Supplementary file 13 — Source data Fig. 1 [file 44321_2025_219_MOESM13_ESM.zip › 1Aii/rce849 mu 1_4 percent saccharin002c1t06.tif]

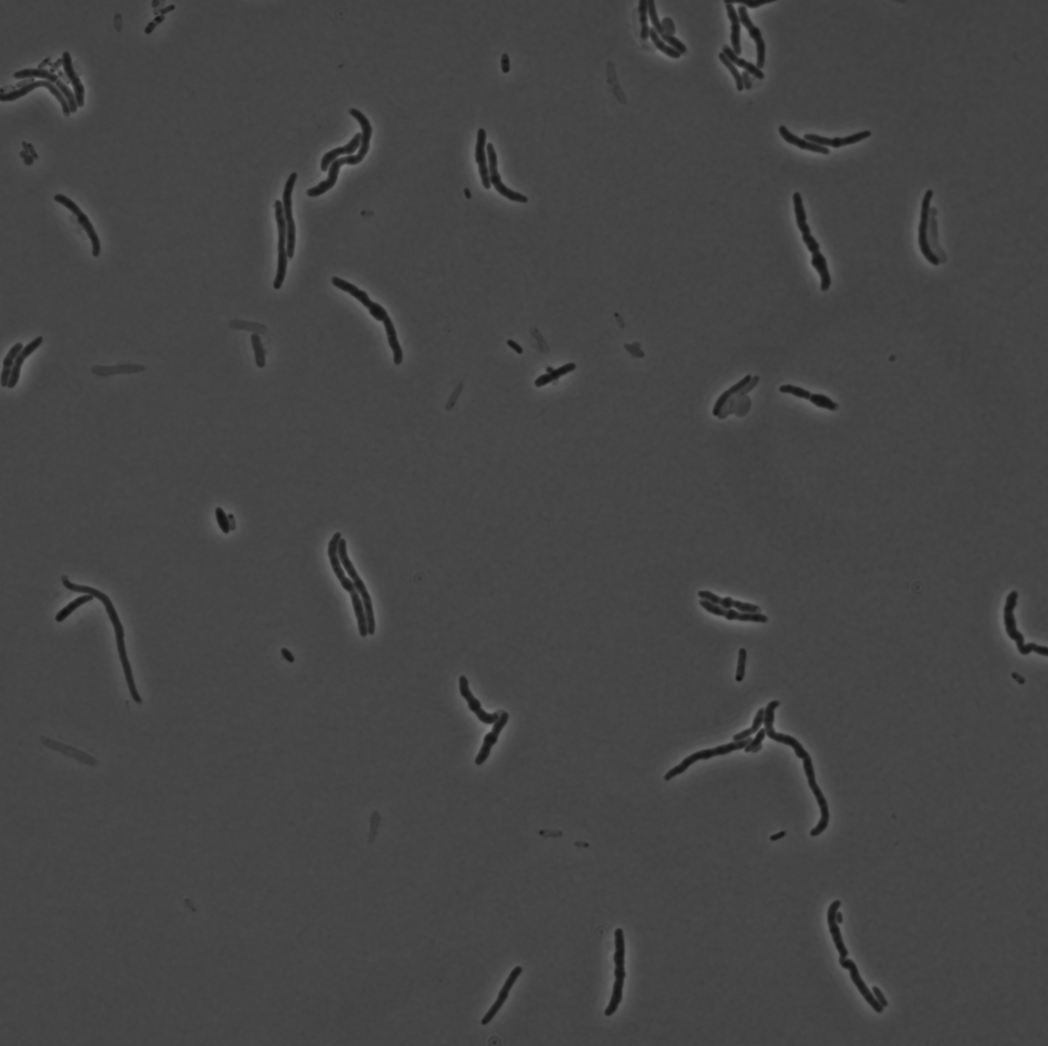

Supplement: Supplementary file 13 — Source data Fig. 1 [file 44321_2025_219_MOESM13_ESM.zip › 1Aii/rce849 mu 1_4 percent saccharin002c1t12.tif]

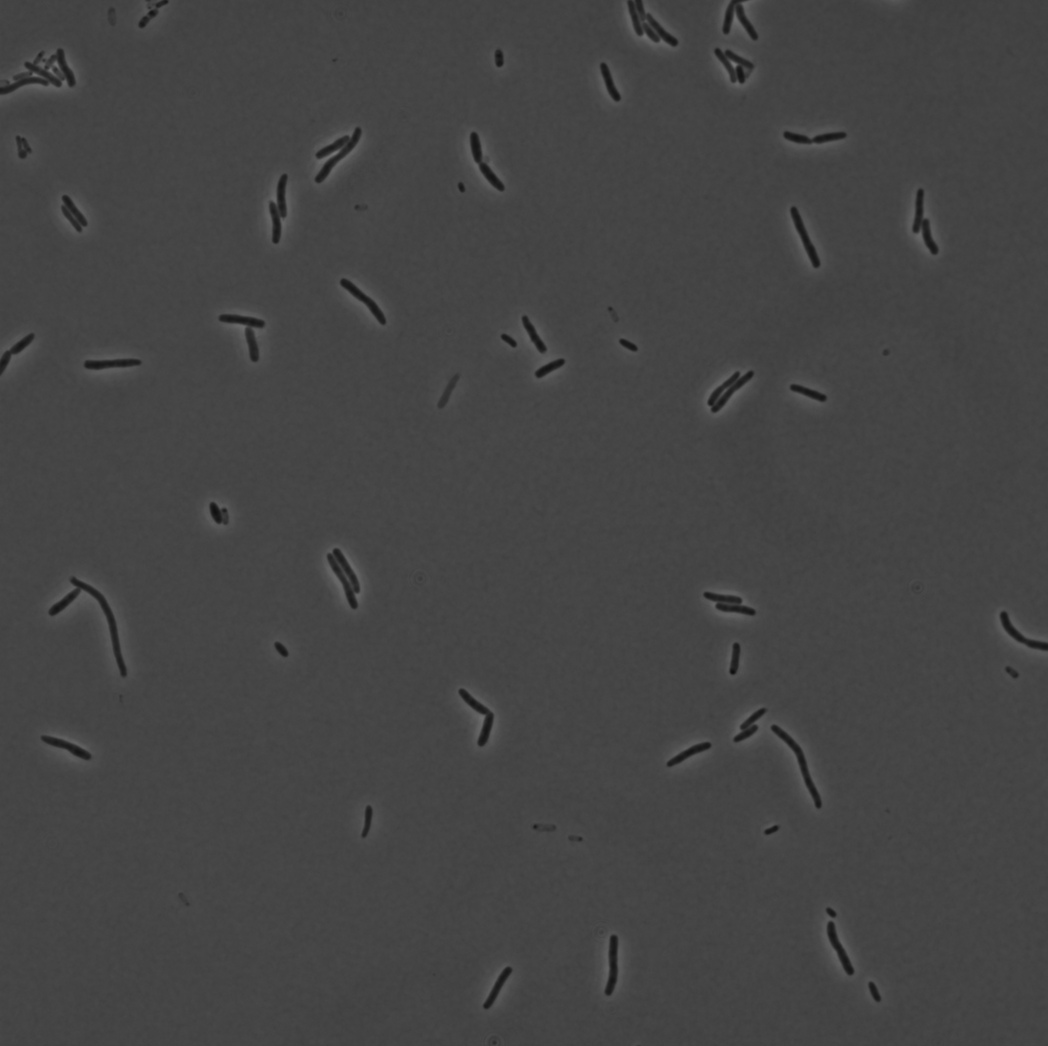

Supplement: Supplementary file 13 — Source data Fig. 1 [file 44321_2025_219_MOESM13_ESM.zip › 1Aii/rce849 mu 1_4 percent saccharin002c1t04.tif]

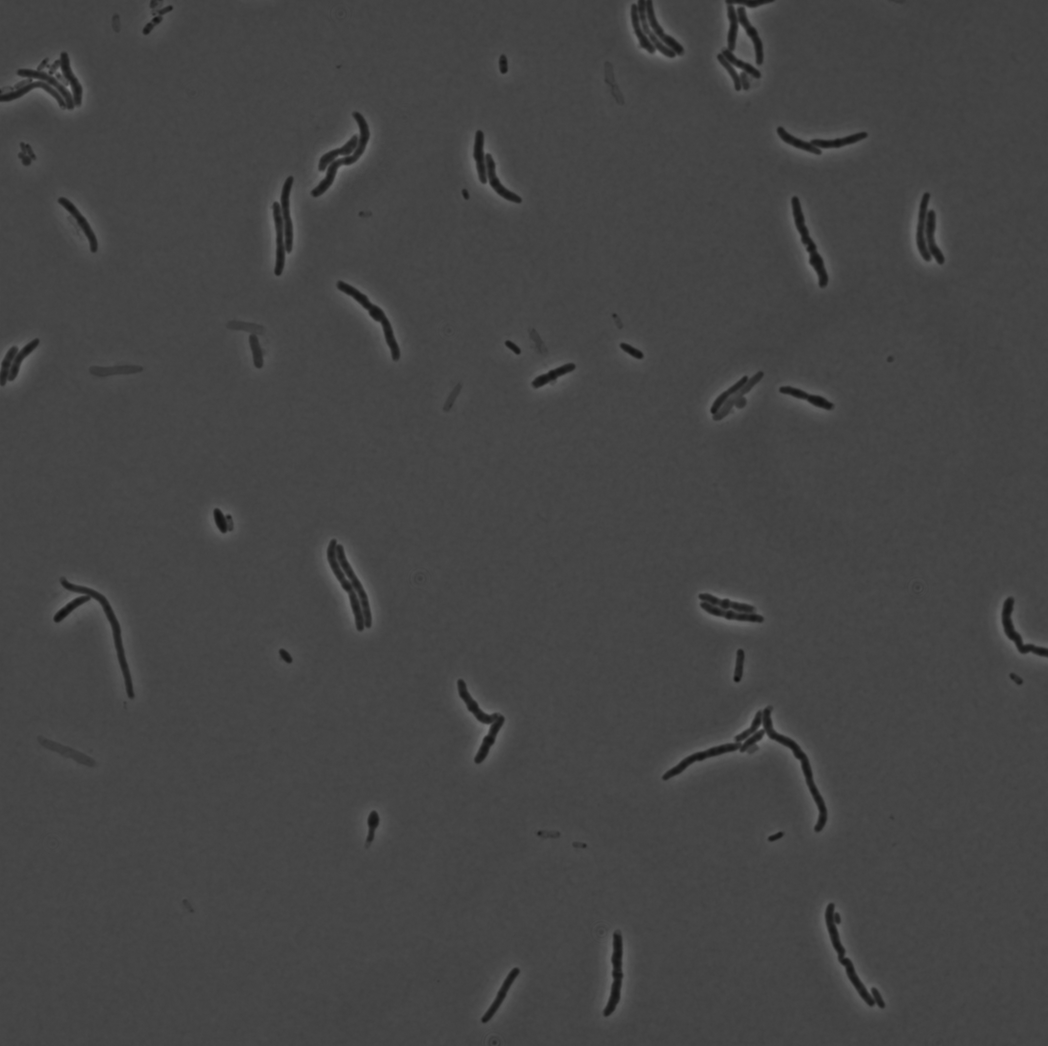

Supplement: Supplementary file 13 — Source data Fig. 1 [file 44321_2025_219_MOESM13_ESM.zip › 1Aii/rce849 mu 1_4 percent saccharin002c1t10.tif]

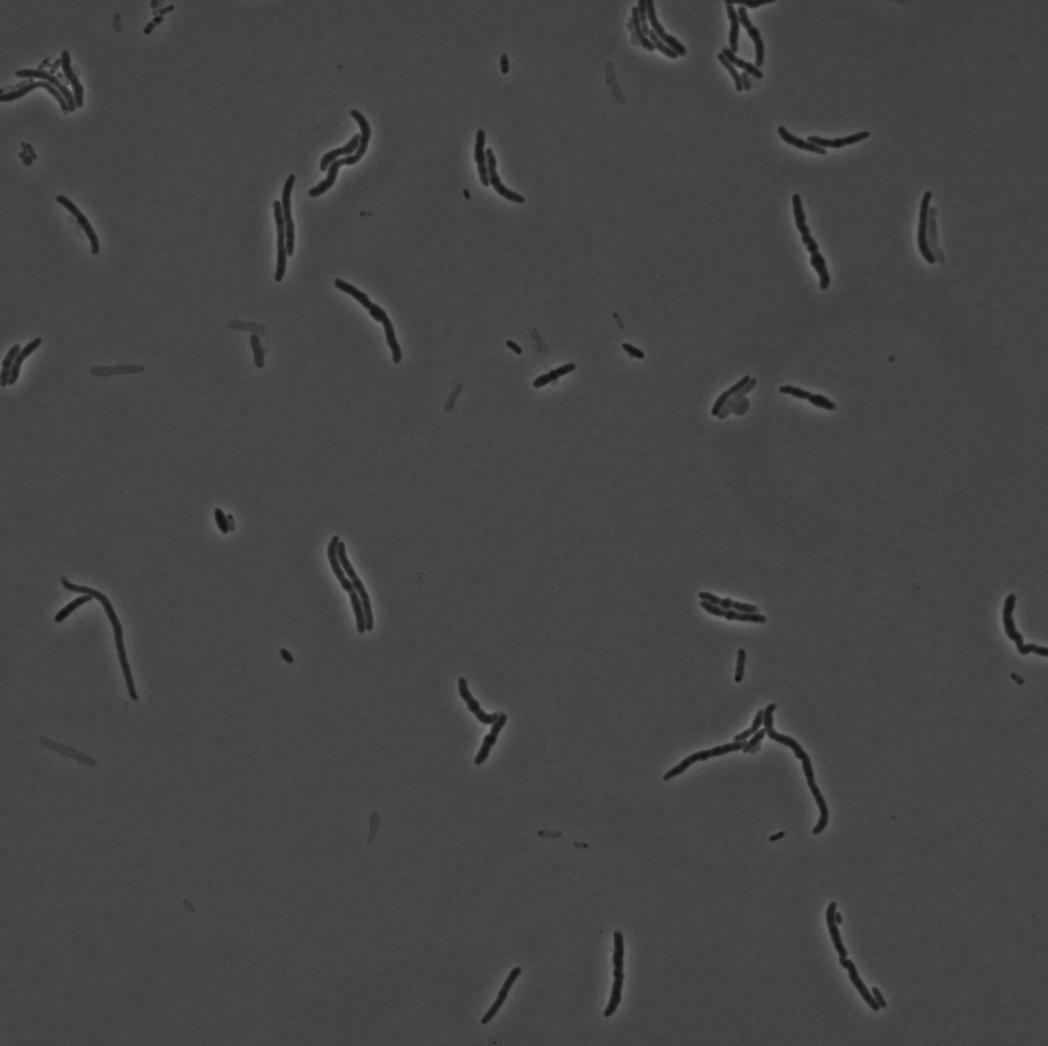

Supplement: Supplementary file 13 — Source data Fig. 1 [file 44321_2025_219_MOESM13_ESM.zip › 1Aii/rce849 mu 1_4 percent saccharin002c1t11.tif]

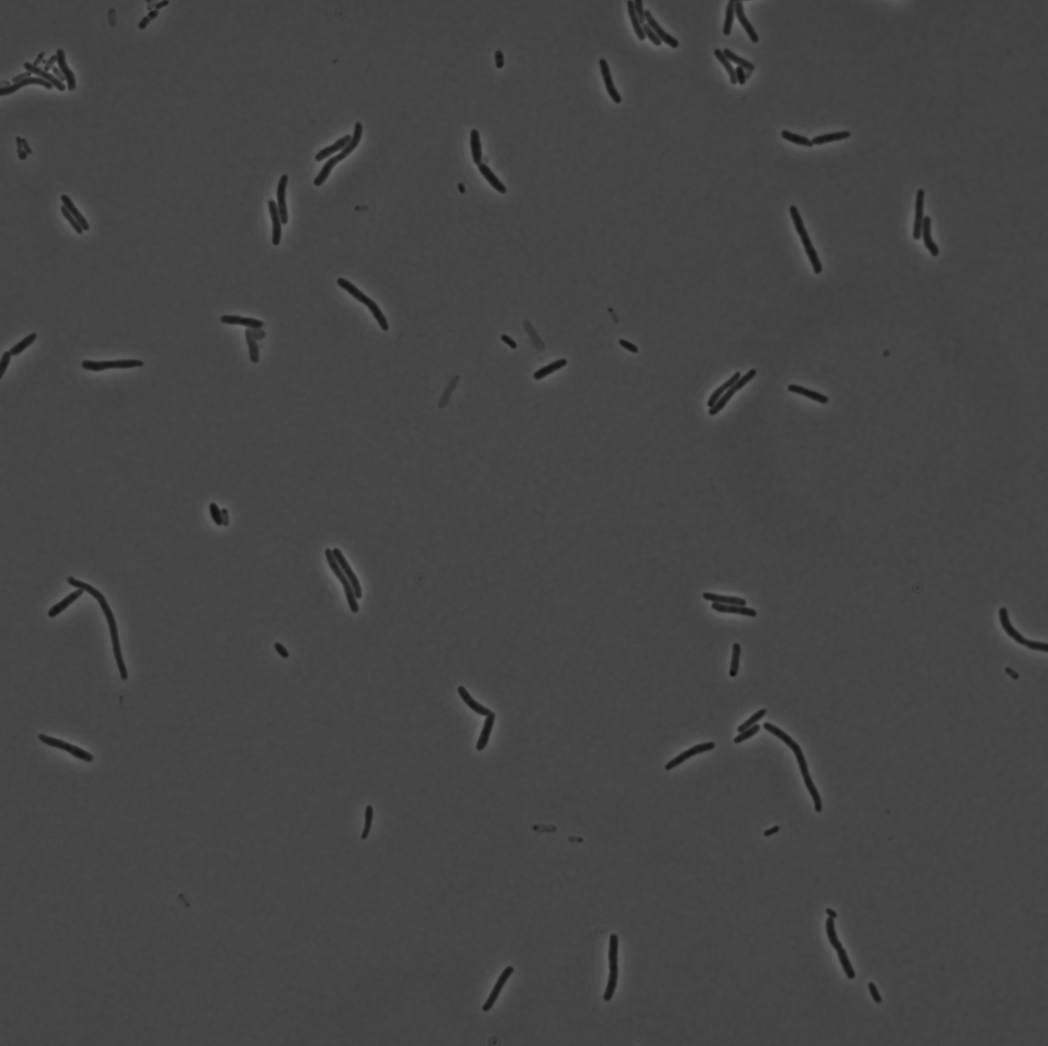

Supplement: Supplementary file 13 — Source data Fig. 1 [file 44321_2025_219_MOESM13_ESM.zip › 1Aii/rce849 mu 1_4 percent saccharin002c1t05.tif]

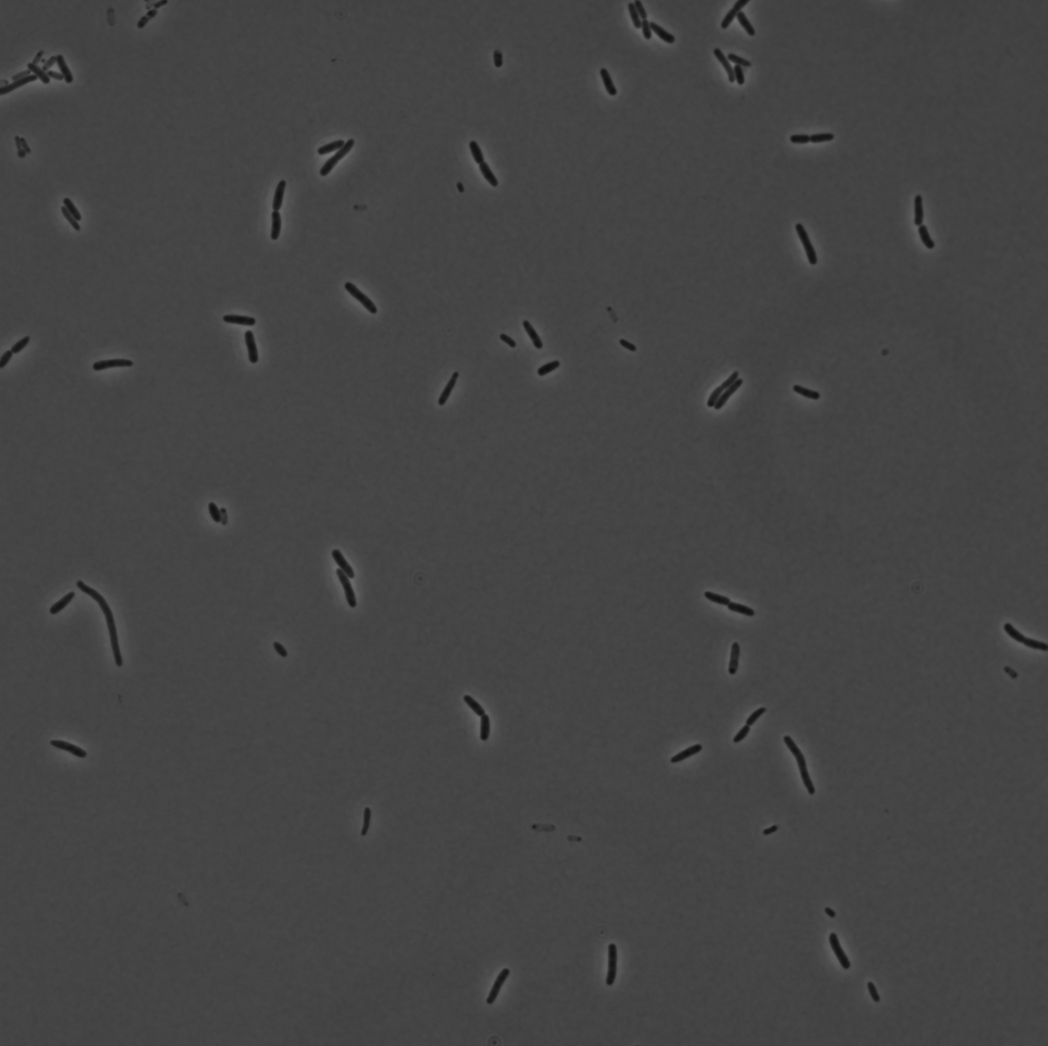

Supplement: Supplementary file 13 — Source data Fig. 1 [file 44321_2025_219_MOESM13_ESM.zip › 1Aii/rce849 mu 1_4 percent saccharin002c1t01.tif]

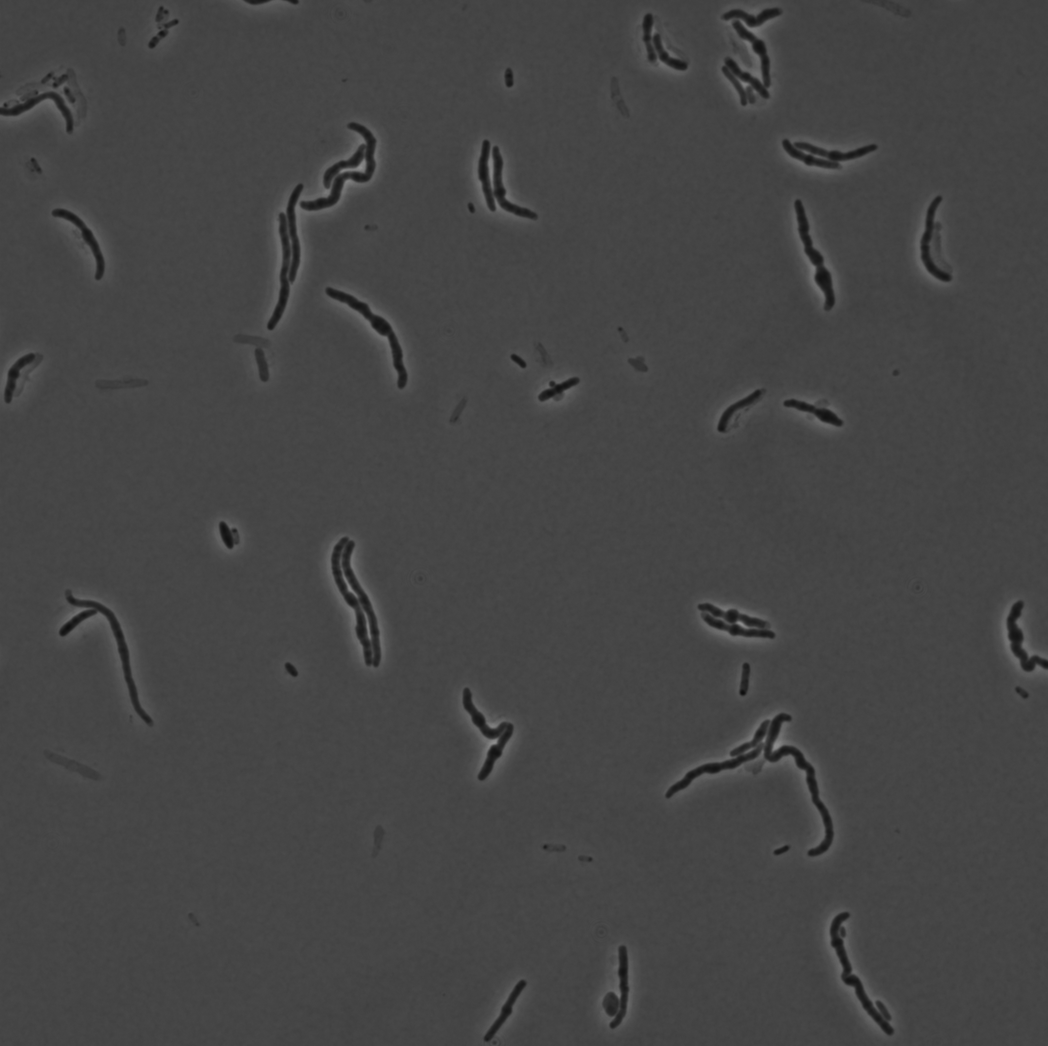

Supplement: Supplementary file 13 — Source data Fig. 1 [file 44321_2025_219_MOESM13_ESM.zip › 1Aii/rce849 mu 1_4 percent saccharin002c1t15.tif]

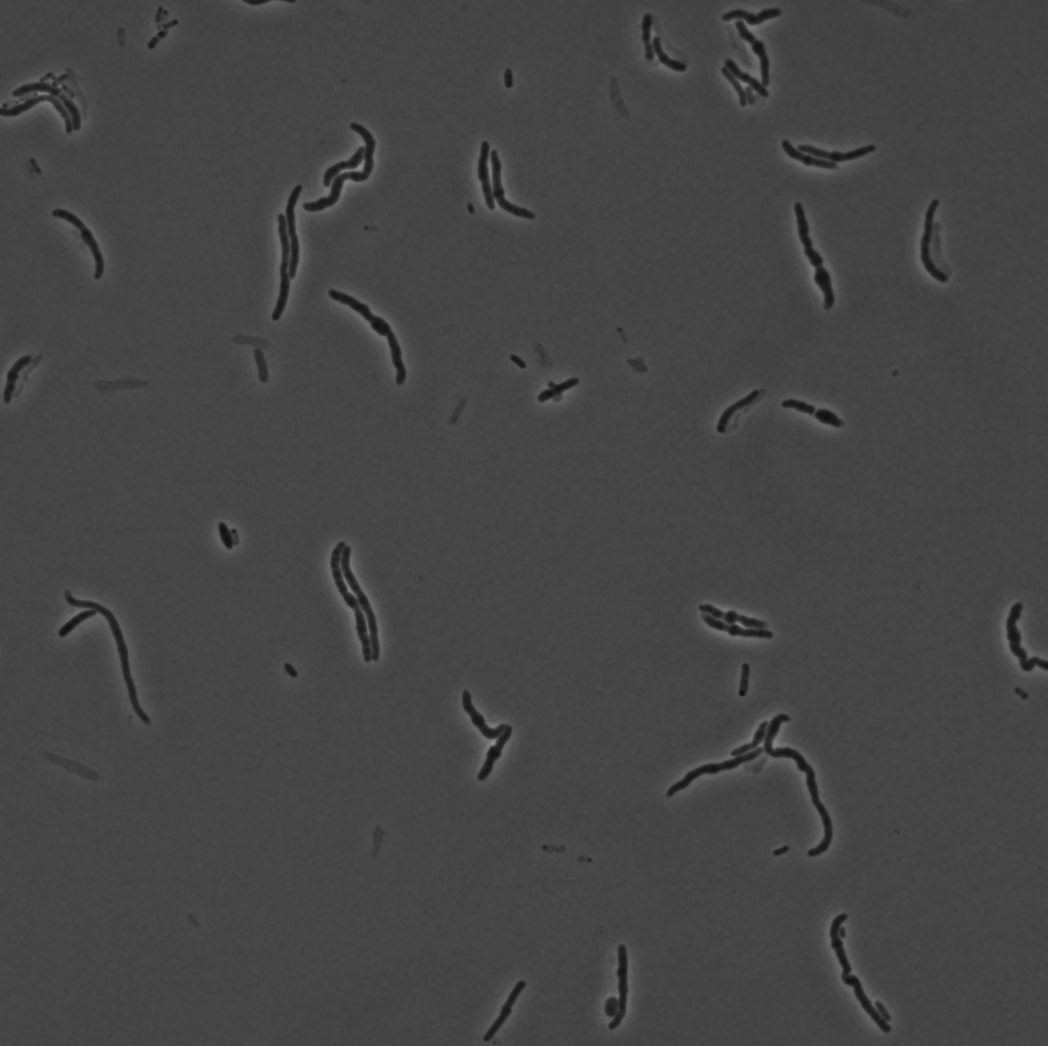

Supplement: Supplementary file 13 — Source data Fig. 1 [file 44321_2025_219_MOESM13_ESM.zip › 1Aii/rce849 mu 1_4 percent saccharin002c1t14.tif]

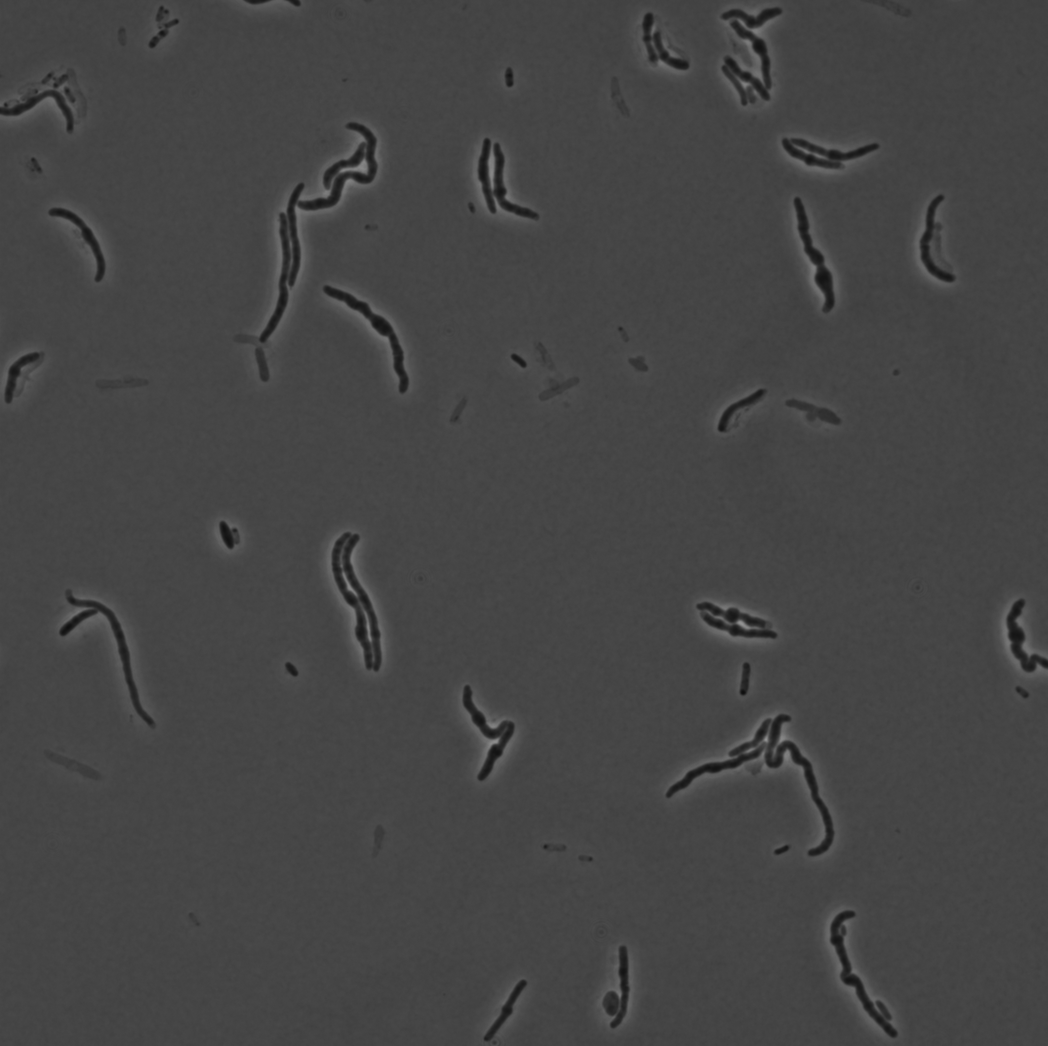

Supplement: Supplementary file 13 — Source data Fig. 1 [file 44321_2025_219_MOESM13_ESM.zip › 1Aii/rce849 mu 1_4 percent saccharin002c1t16.tif]

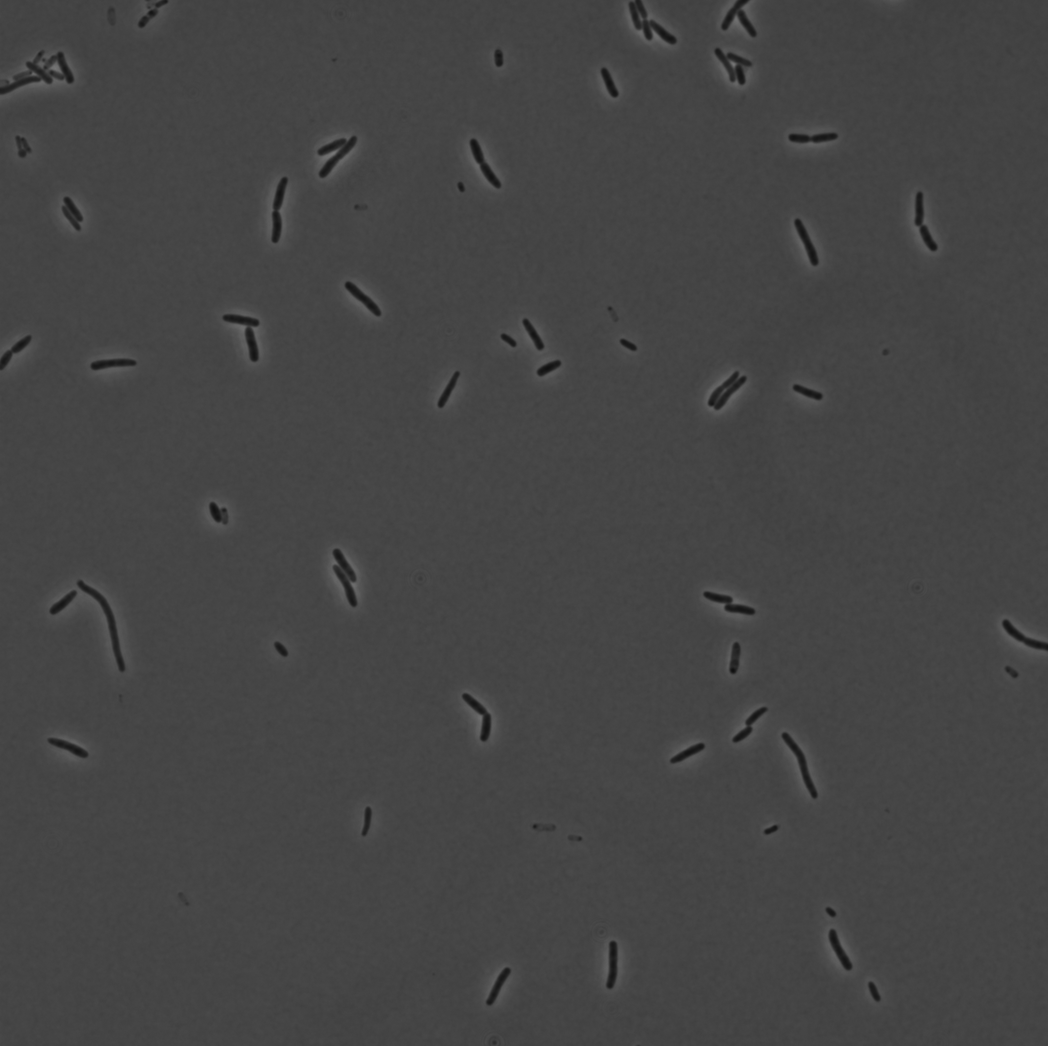

Supplement: Supplementary file 13 — Source data Fig. 1 [file 44321_2025_219_MOESM13_ESM.zip › 1Aii/rce849 mu 1_4 percent saccharin002c1t02.tif]

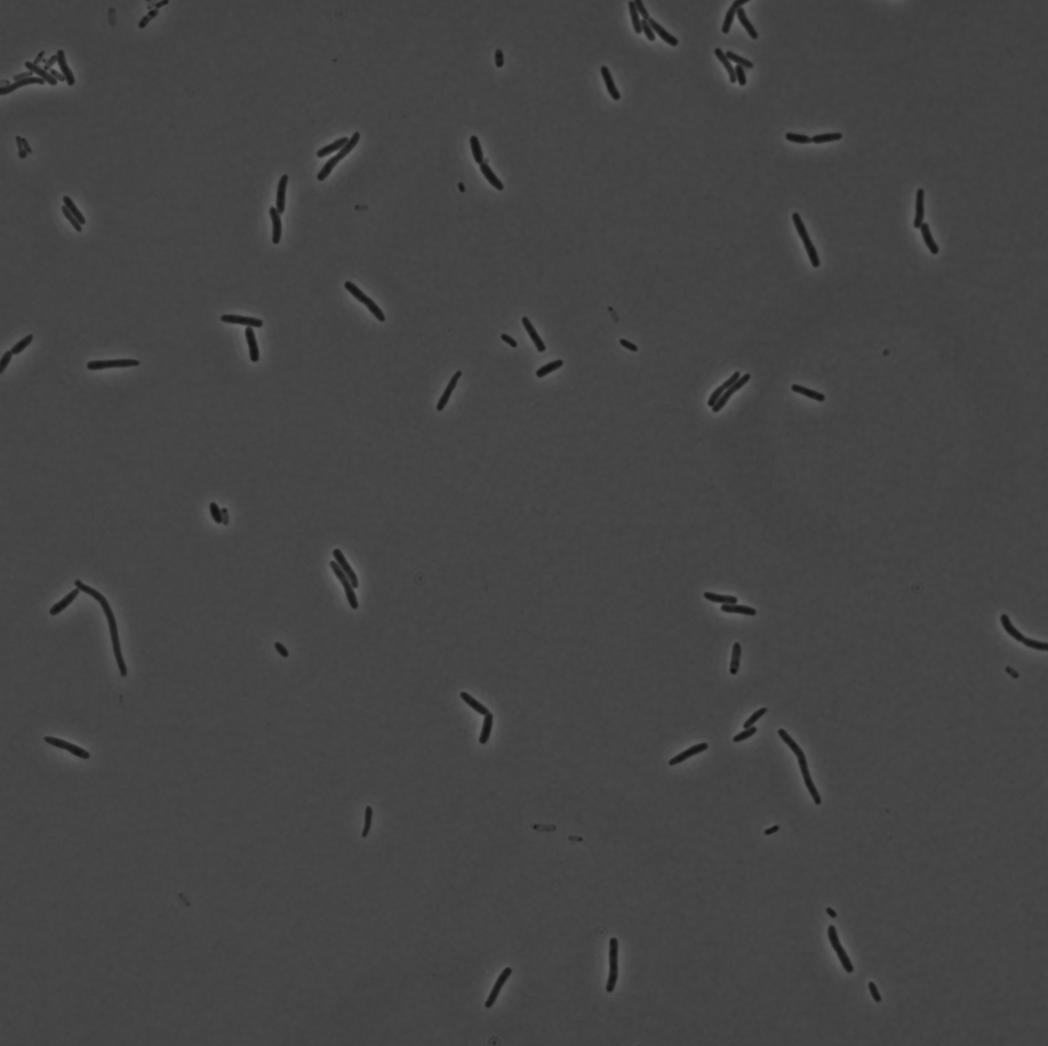

Supplement: Supplementary file 13 — Source data Fig. 1 [file 44321_2025_219_MOESM13_ESM.zip › 1Aii/rce849 mu 1_4 percent saccharin002c1t03.tif]

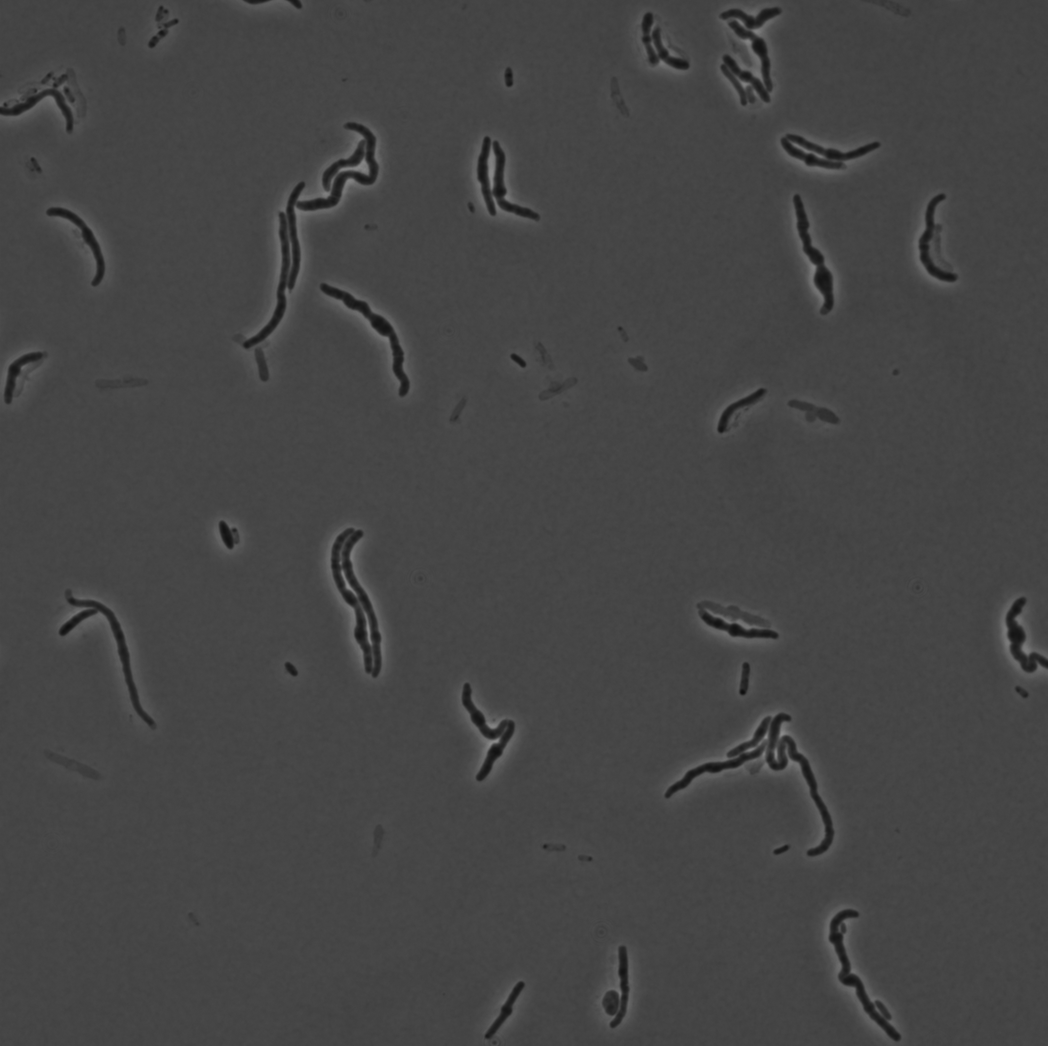

Supplement: Supplementary file 13 — Source data Fig. 1 [file 44321_2025_219_MOESM13_ESM.zip › 1Aii/rce849 mu 1_4 percent saccharin002c1t17.tif]

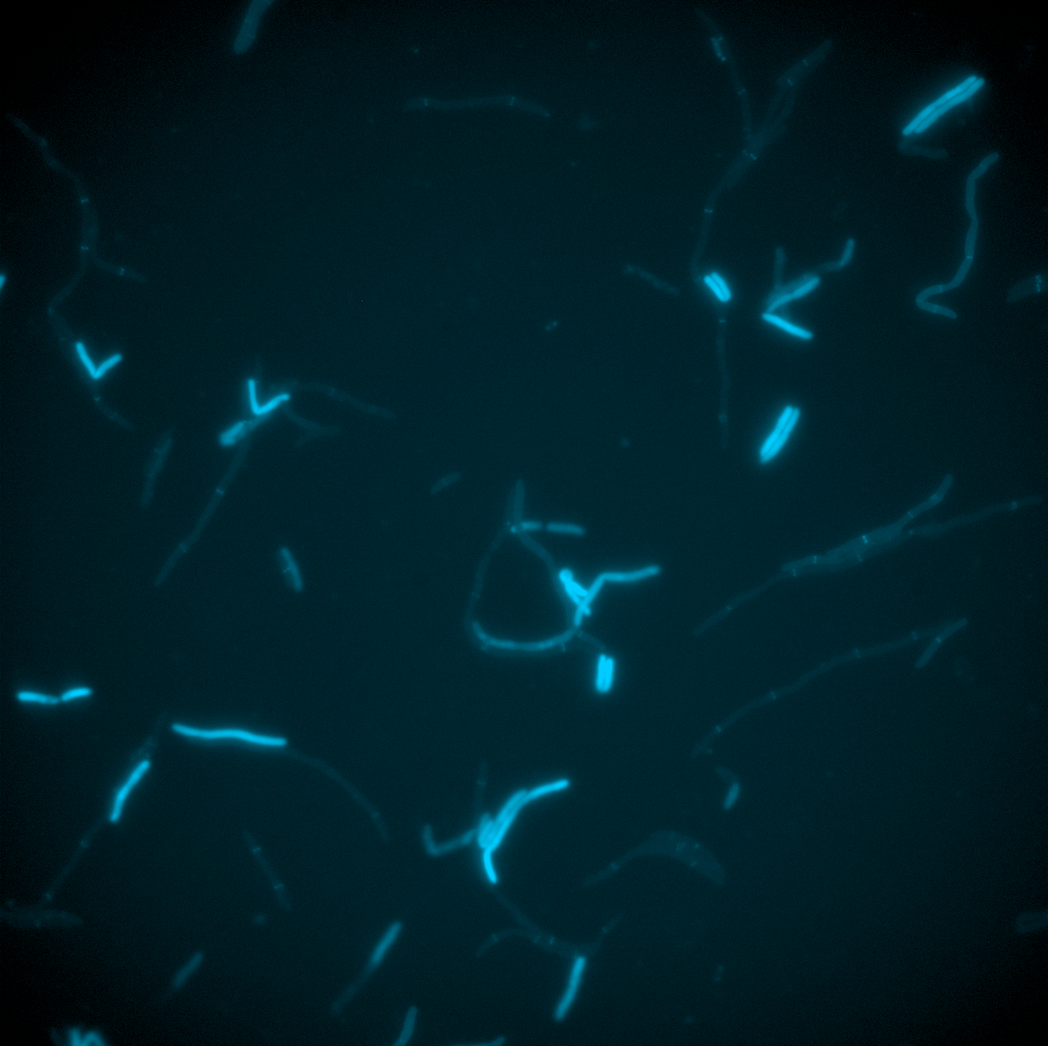

Supplement: Supplementary file 13 — Source data Fig. 1 [file 44321_2025_219_MOESM13_ESM.zip › 1B/RCe849 1_4 saccharin 90 min019_RGB_eCFP.tif]

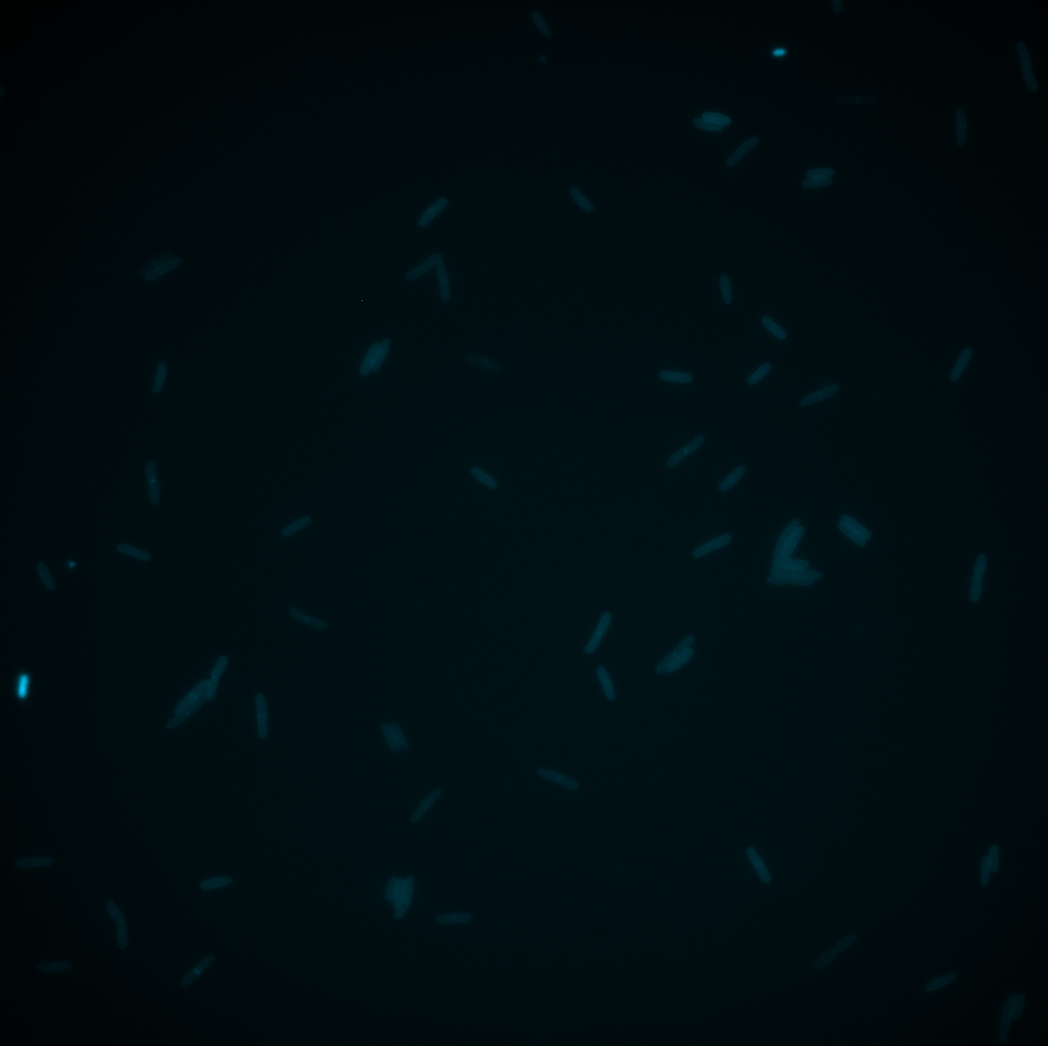

Supplement: Supplementary file 13 — Source data Fig. 1 [file 44321_2025_219_MOESM13_ESM.zip › 1B/RCe849 1_4 saccharin 0 min002_RGB_eCFP.tif]

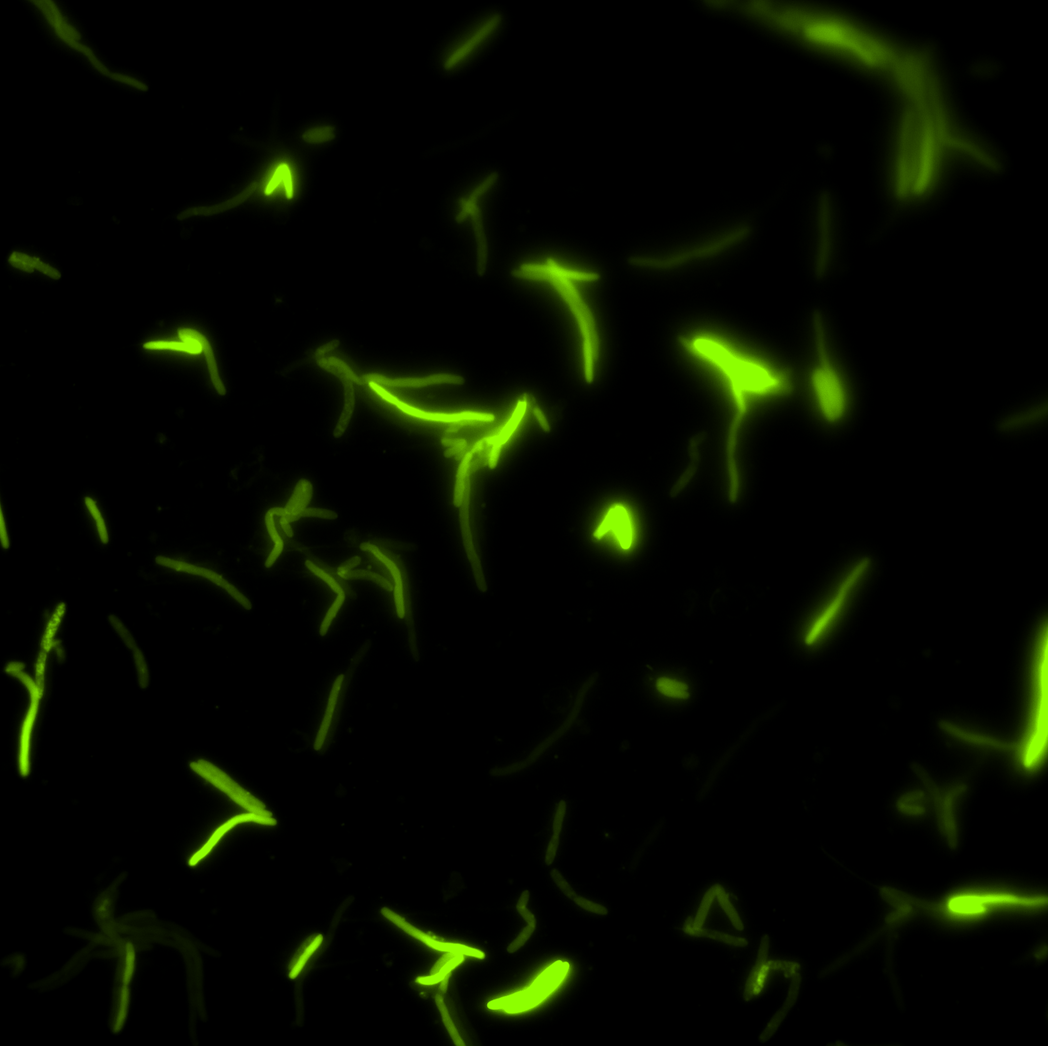

Supplement: Supplementary file 13 — Source data Fig. 1 [file 44321_2025_219_MOESM13_ESM.zip › 1B/RCe849 1_4 saccharin 60 min012_RGB_eYFP.tif]

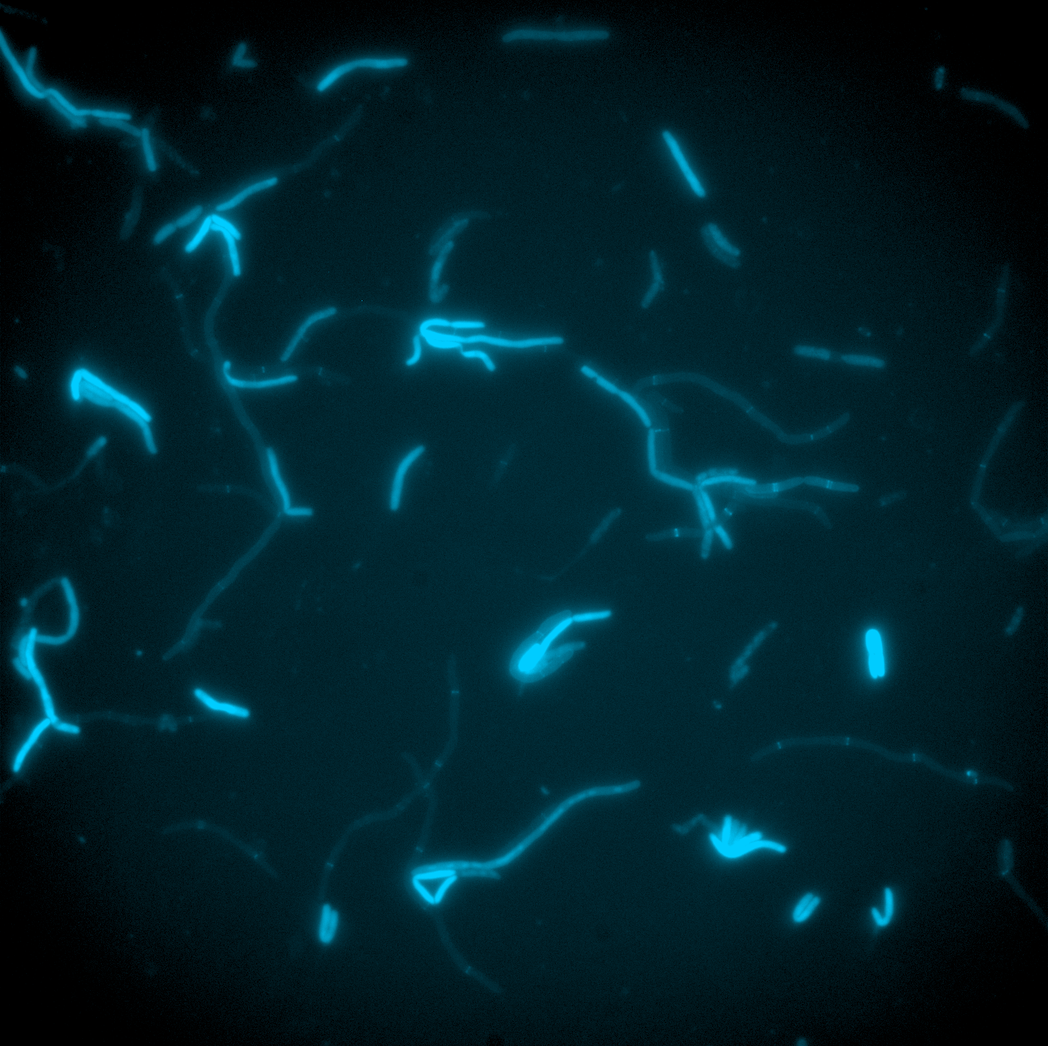

Supplement: Supplementary file 13 — Source data Fig. 1 [file 44321_2025_219_MOESM13_ESM.zip › 1B/RCe849 1_4 saccharin 90 min018_RGB_eCFP.tif]

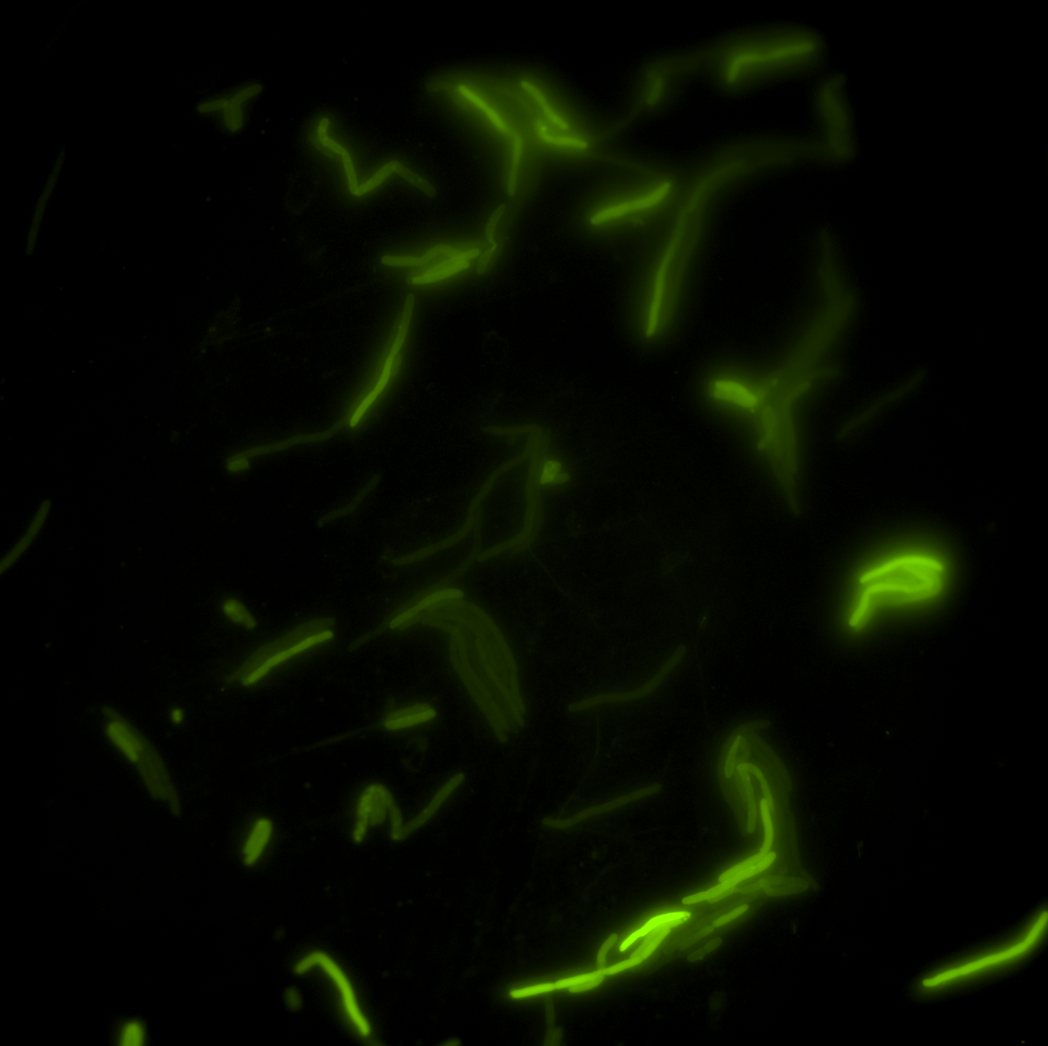

Supplement: Supplementary file 13 — Source data Fig. 1 [file 44321_2025_219_MOESM13_ESM.zip › 1B/RCe849 1_4 saccharin 60 min013_RGB_eYFP.tif]

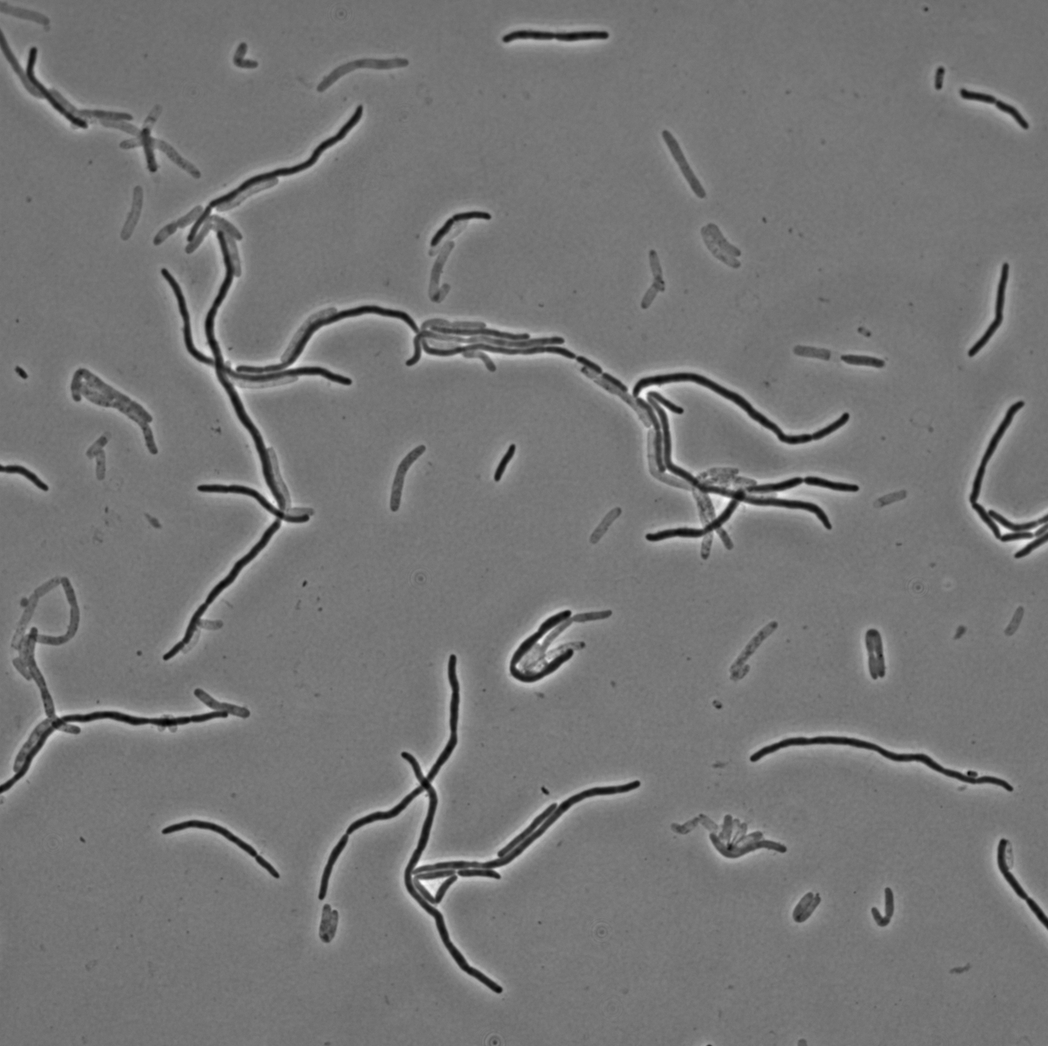

Supplement: Supplementary file 13 — Source data Fig. 1 [file 44321_2025_219_MOESM13_ESM.zip › 1B/RCe849 1_4 saccharin 90 min018_RGB_Brightfield.tif]

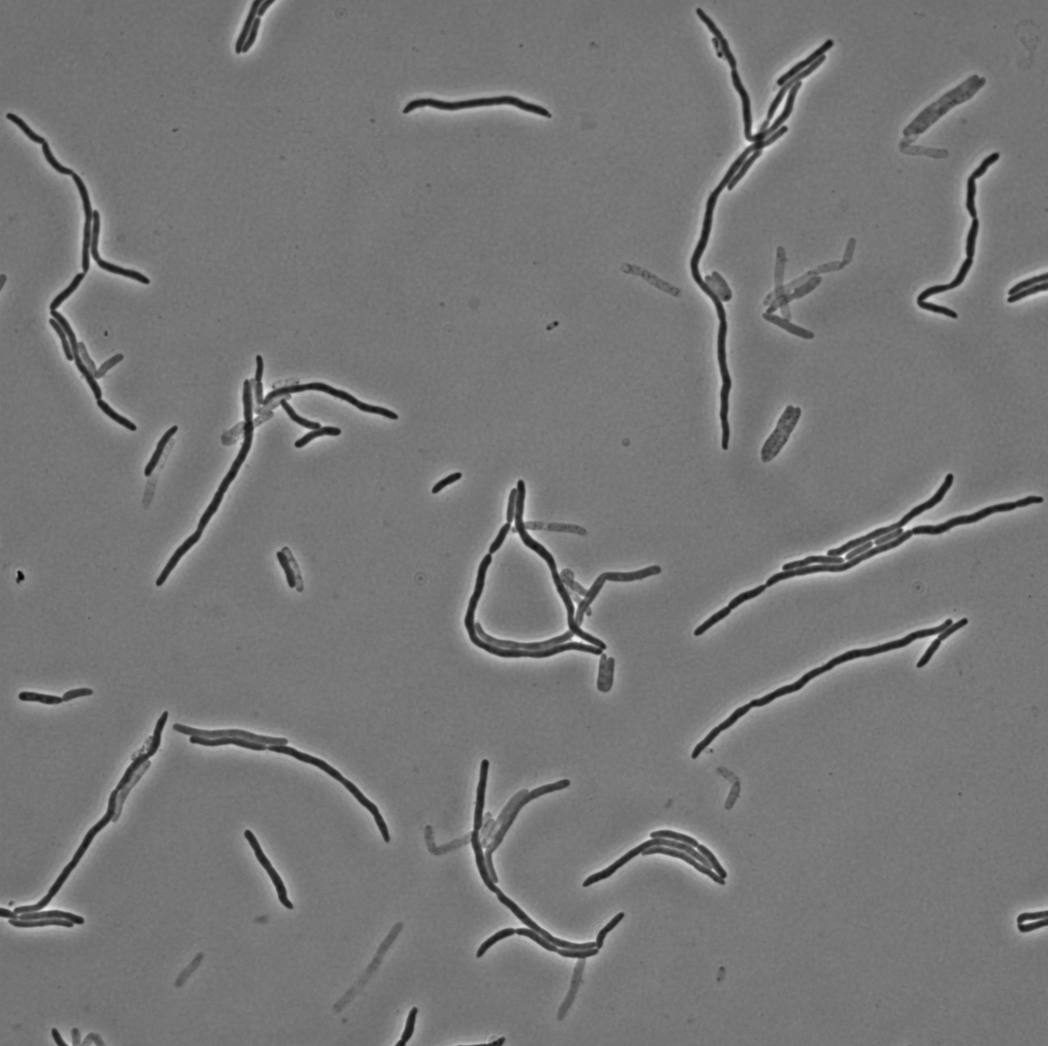

Supplement: Supplementary file 13 — Source data Fig. 1 [file 44321_2025_219_MOESM13_ESM.zip › 1B/RCe849 1_4 saccharin 90 min019_RGB_Brightfield.tif]

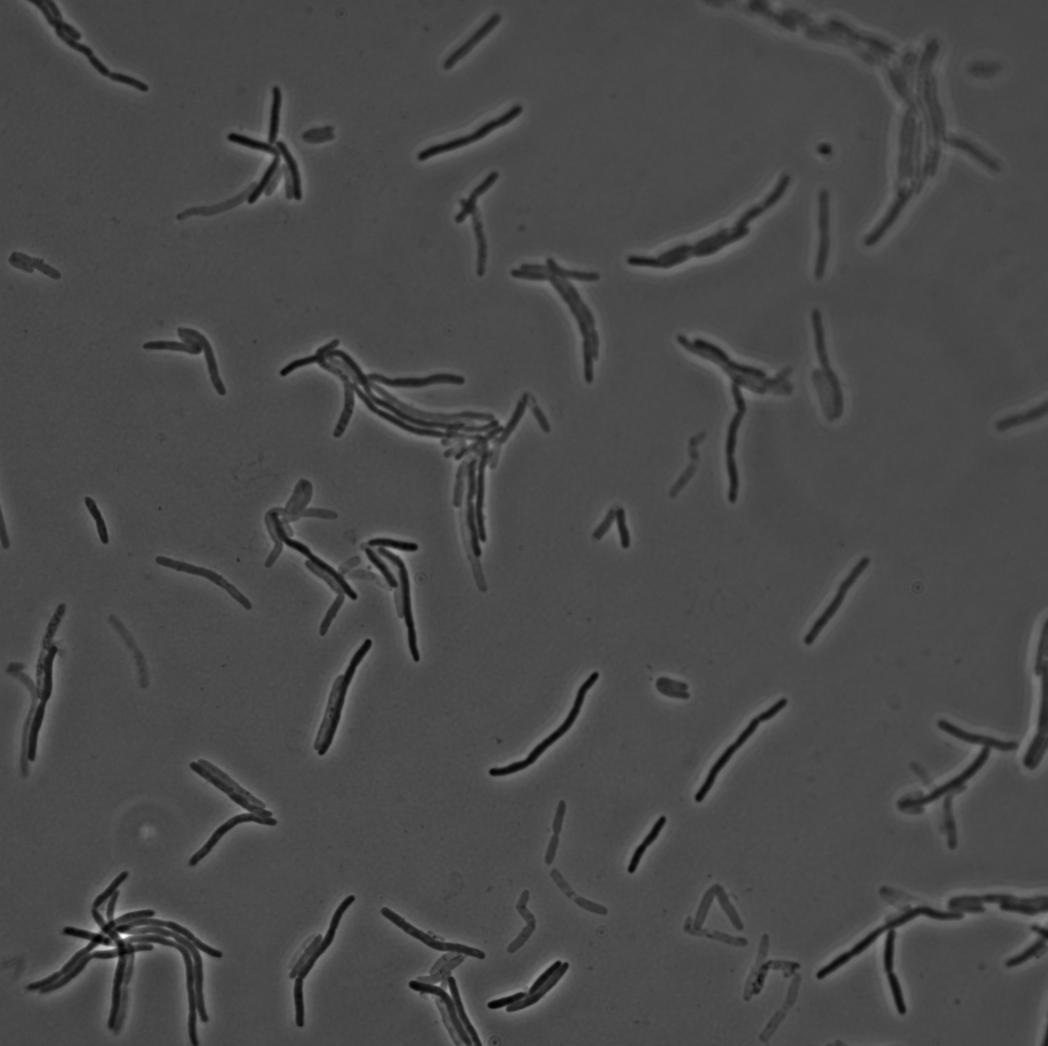

Supplement: Supplementary file 13 — Source data Fig. 1 [file 44321_2025_219_MOESM13_ESM.zip › 1B/RCe849 1_4 saccharin 60 min012_RGB_Brightfield.tif]

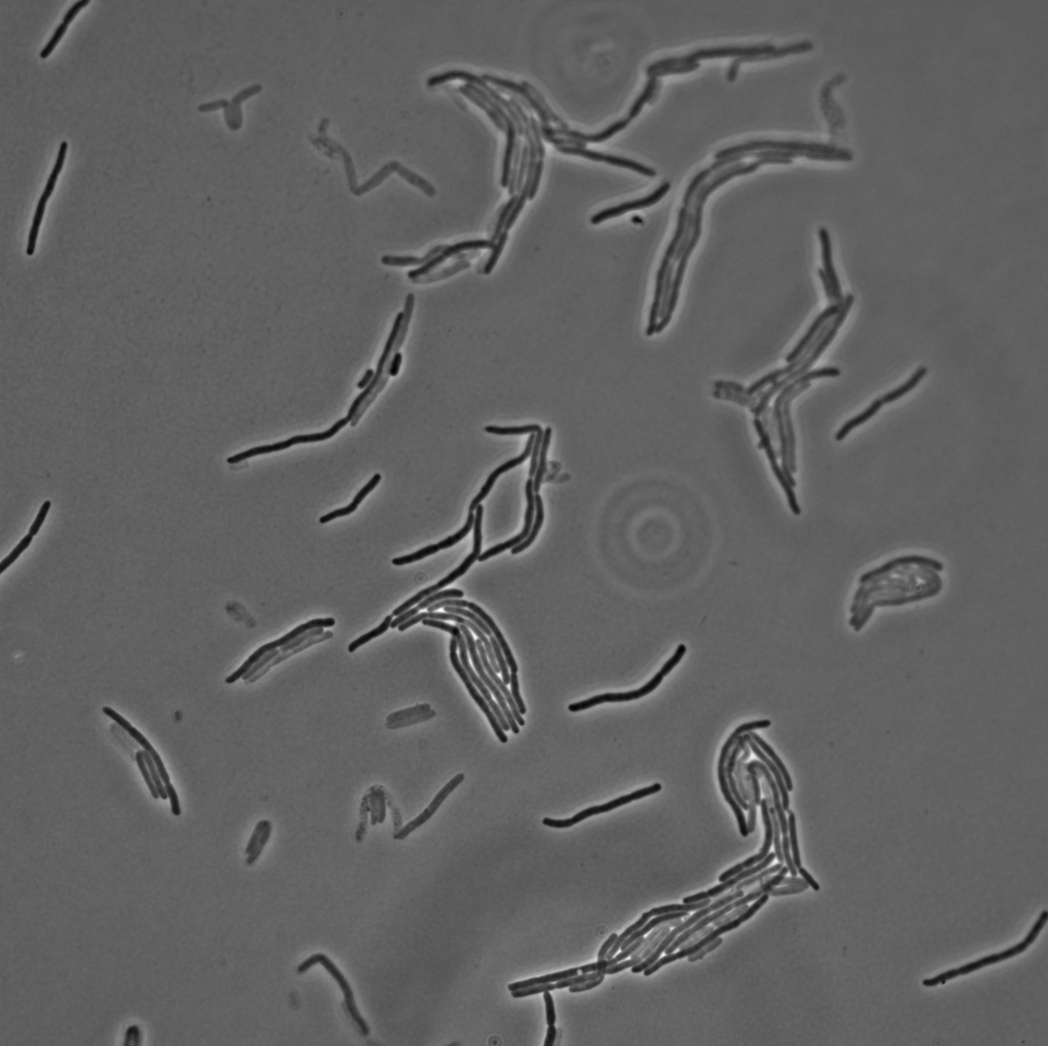

Supplement: Supplementary file 13 — Source data Fig. 1 [file 44321_2025_219_MOESM13_ESM.zip › 1B/RCe849 1_4 saccharin 60 min013_RGB_Brightfield.tif]

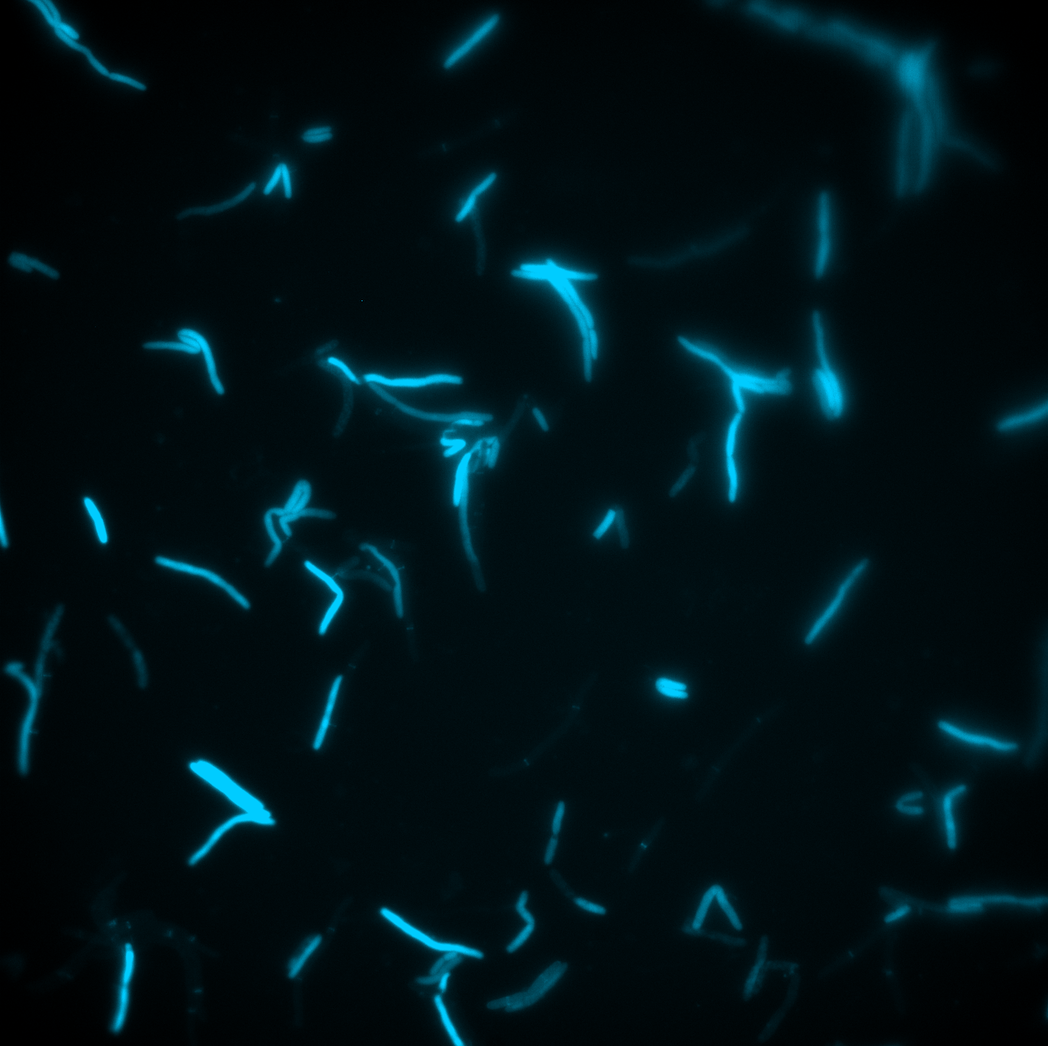

Supplement: Supplementary file 13 — Source data Fig. 1 [file 44321_2025_219_MOESM13_ESM.zip › 1B/RCe849 1_4 saccharin 60 min012_RGB_eCFP.tif]

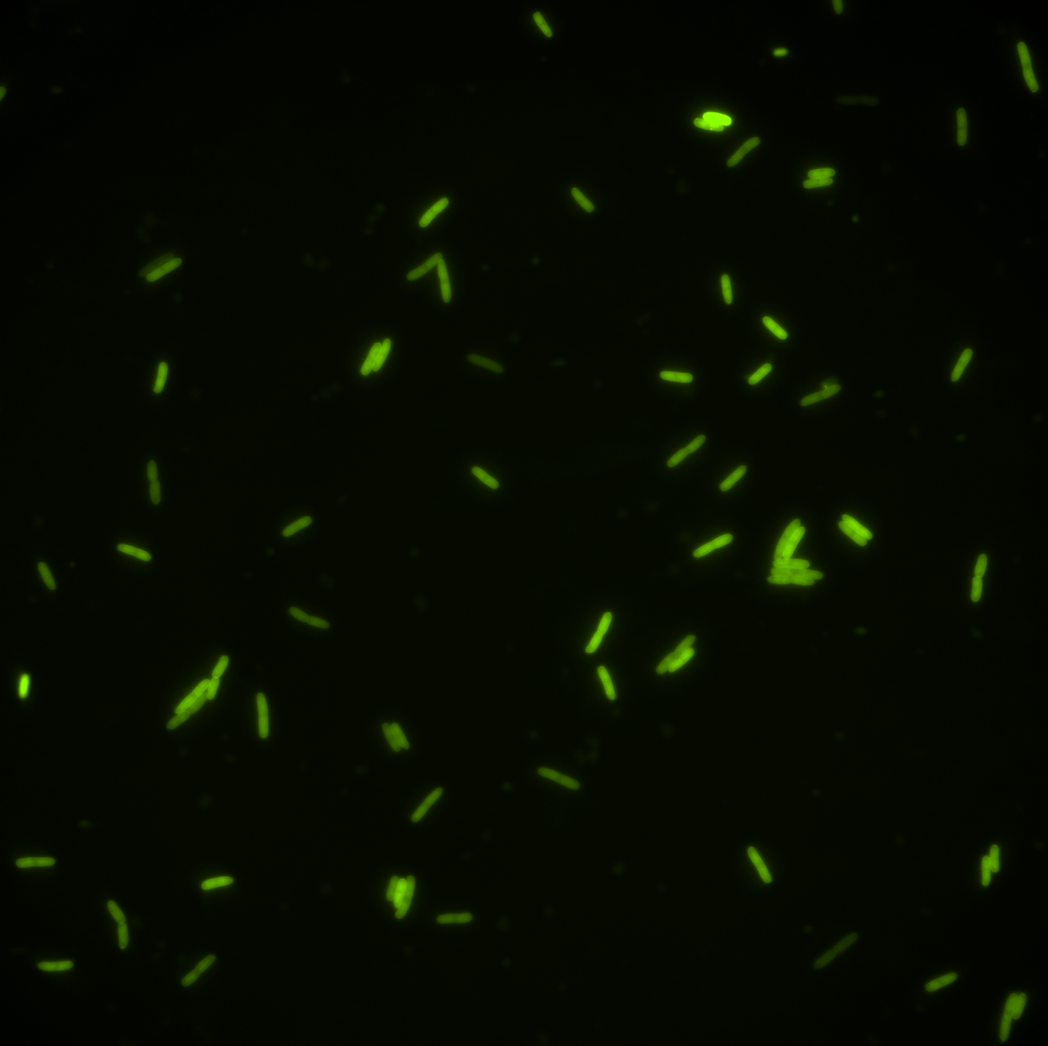

Supplement: Supplementary file 13 — Source data Fig. 1 [file 44321_2025_219_MOESM13_ESM.zip › 1B/RCe849 1_4 saccharin 0 min002_RGB_eYFP.tif]

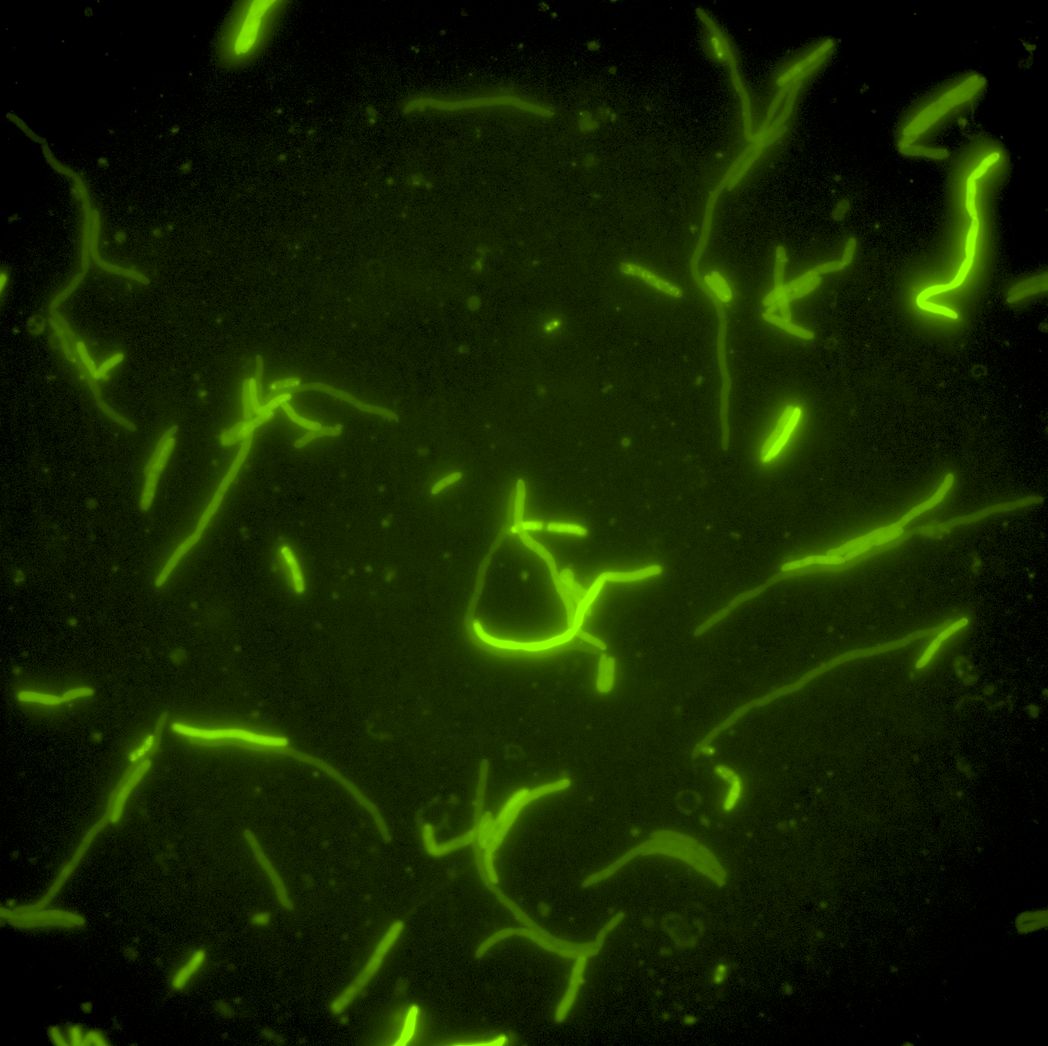

Supplement: Supplementary file 13 — Source data Fig. 1 [file 44321_2025_219_MOESM13_ESM.zip › 1B/RCe849 1_4 saccharin 90 min019_RGB_eYFP.tif]

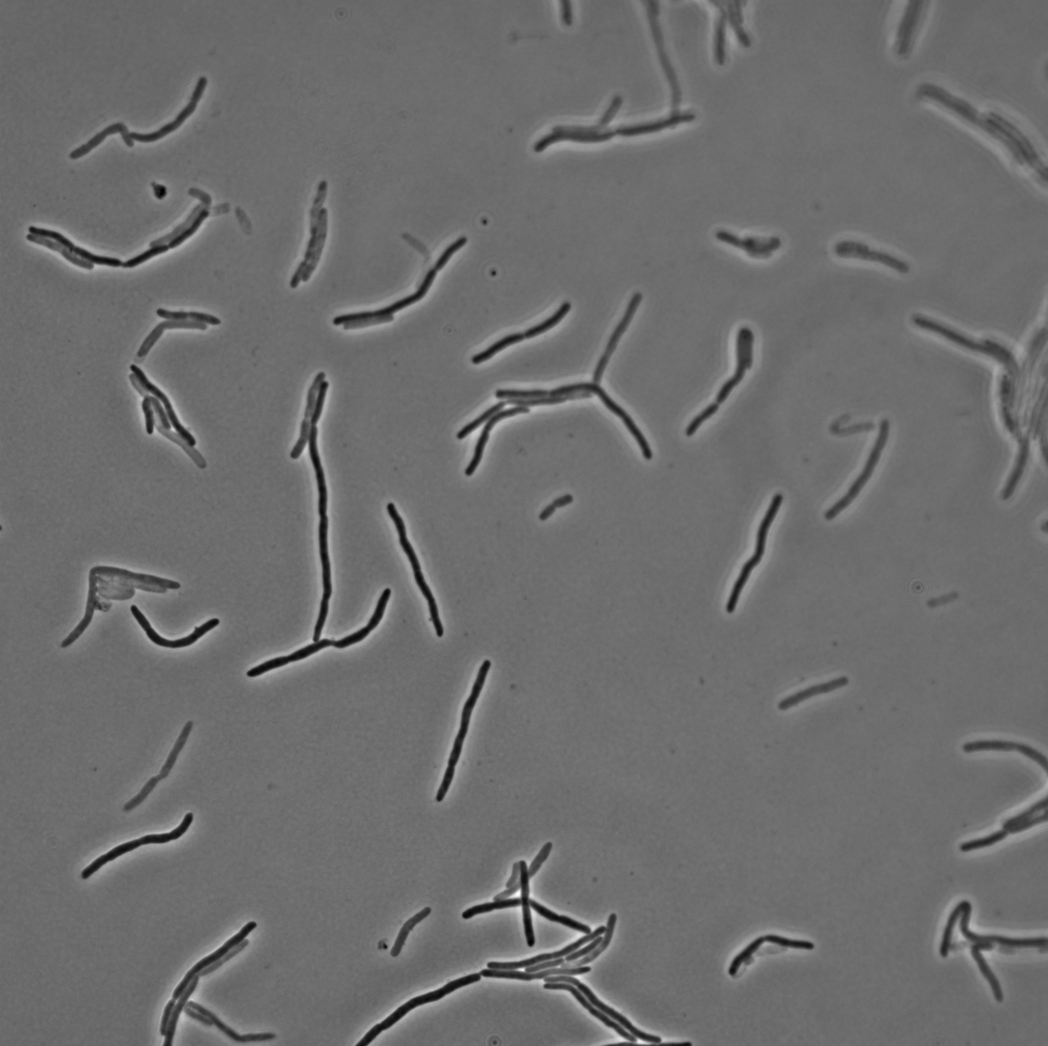

Supplement: Supplementary file 13 — Source data Fig. 1 [file 44321_2025_219_MOESM13_ESM.zip › 1B/RCe849 1_4 saccharin 60 min011_RGB_Brightfield.tif]

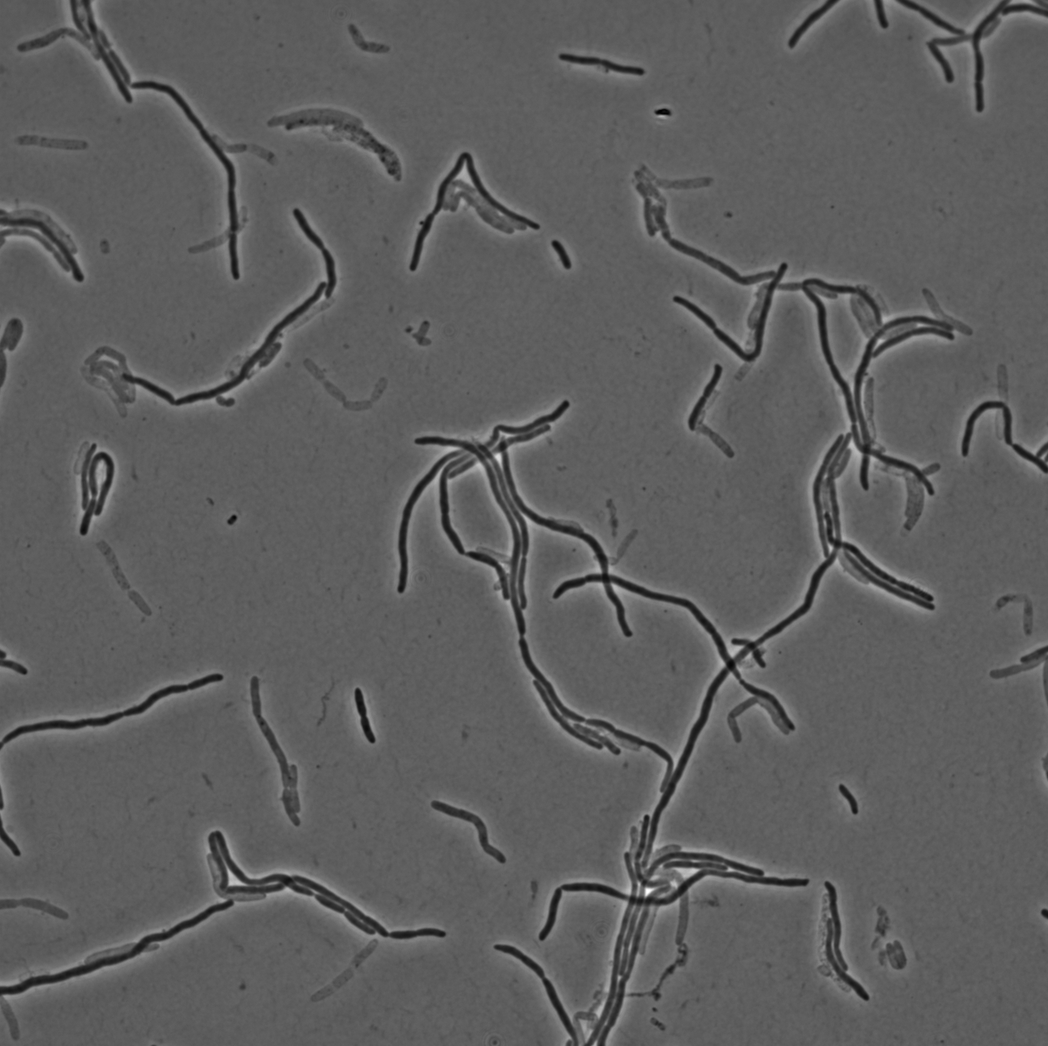

Supplement: Supplementary file 13 — Source data Fig. 1 [file 44321_2025_219_MOESM13_ESM.zip › 1B/RCe849 1_4 saccharin 90 min017_RGB_Brightfield.tif]

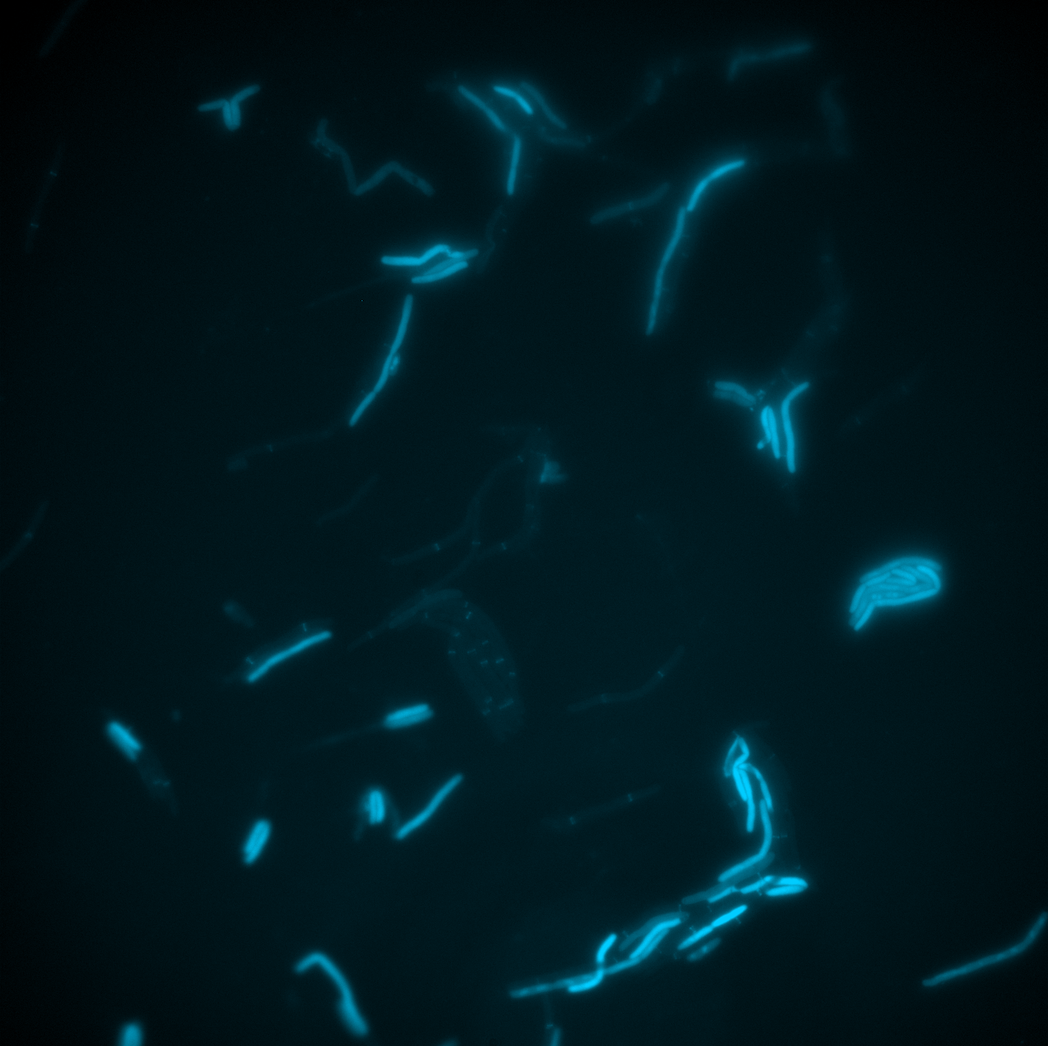

Supplement: Supplementary file 13 — Source data Fig. 1 [file 44321_2025_219_MOESM13_ESM.zip › 1B/RCe849 1_4 saccharin 60 min013_RGB_eCFP.tif]

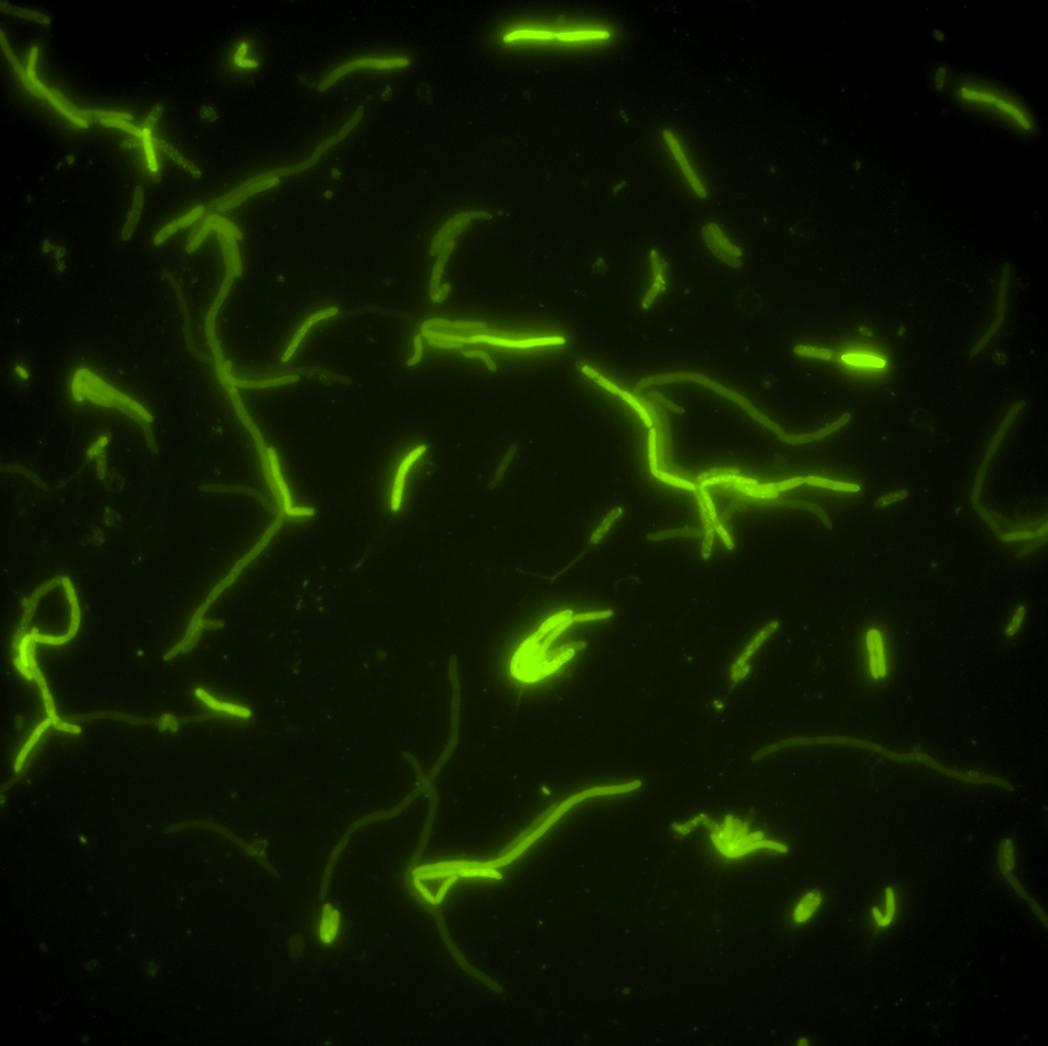

Supplement: Supplementary file 13 — Source data Fig. 1 [file 44321_2025_219_MOESM13_ESM.zip › 1B/RCe849 1_4 saccharin 90 min018_RGB_eYFP.tif]

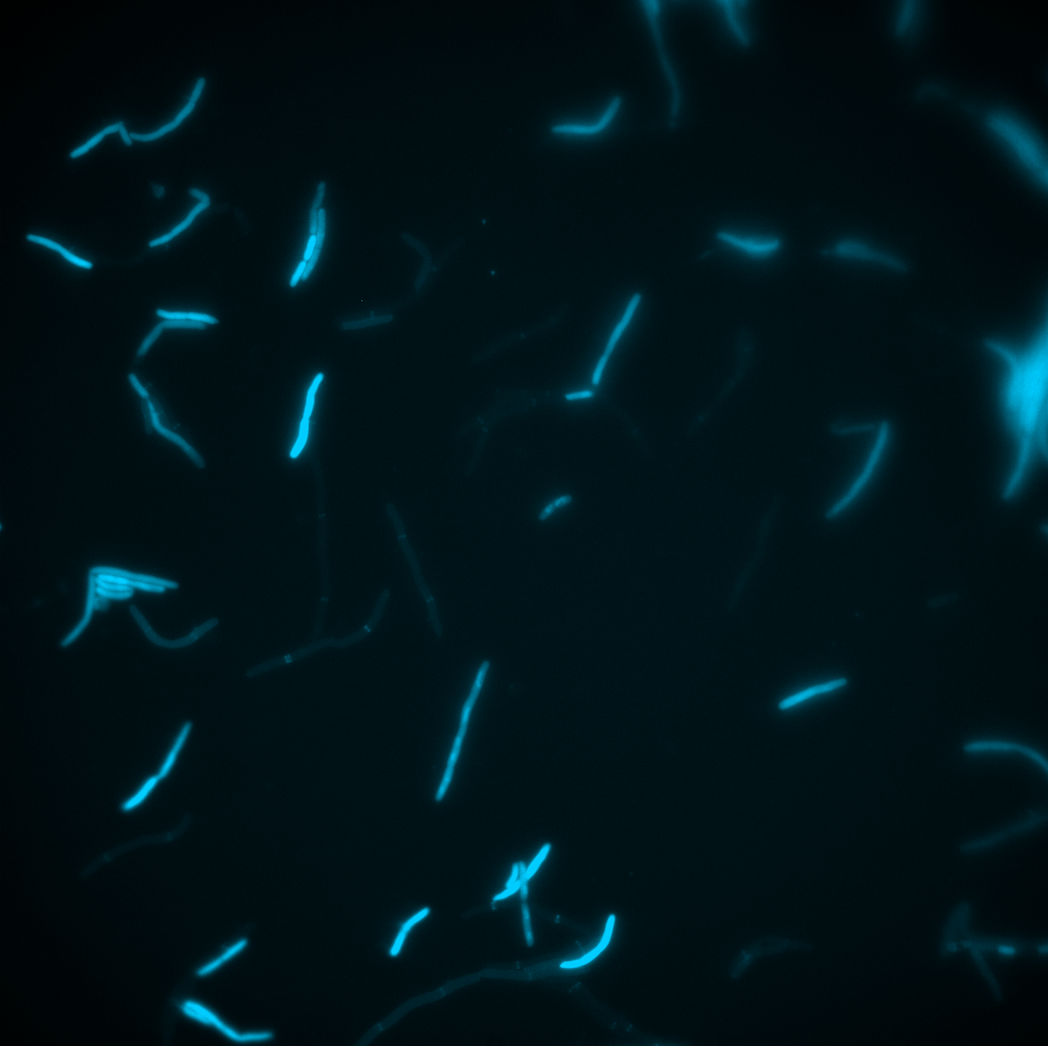

Supplement: Supplementary file 13 — Source data Fig. 1 [file 44321_2025_219_MOESM13_ESM.zip › 1B/RCe849 1_4 saccharin 60 min011_RGB_eCFP.tif]

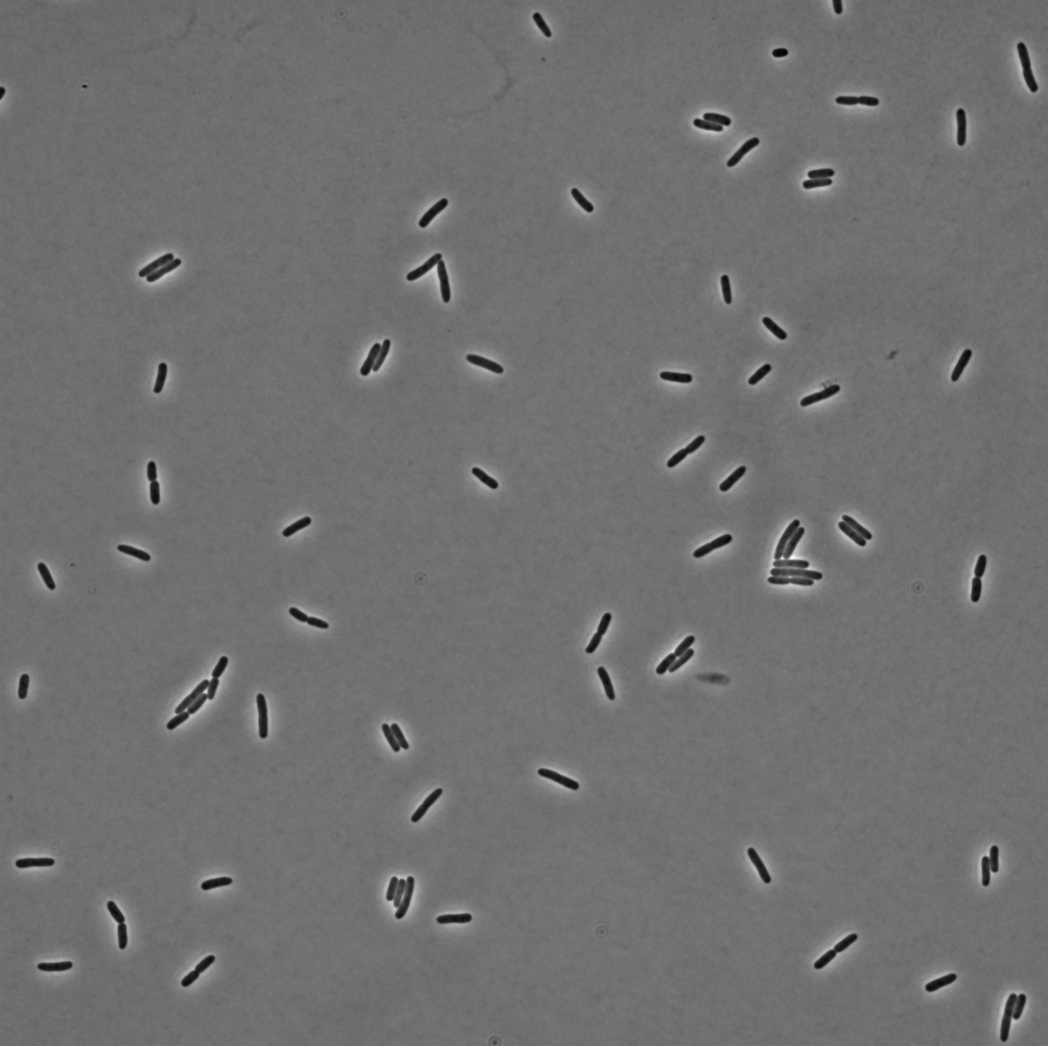

Supplement: Supplementary file 13 — Source data Fig. 1 [file 44321_2025_219_MOESM13_ESM.zip › 1B/RCe849 1_4 saccharin 0 min002_RGB_Brightfield.tif]

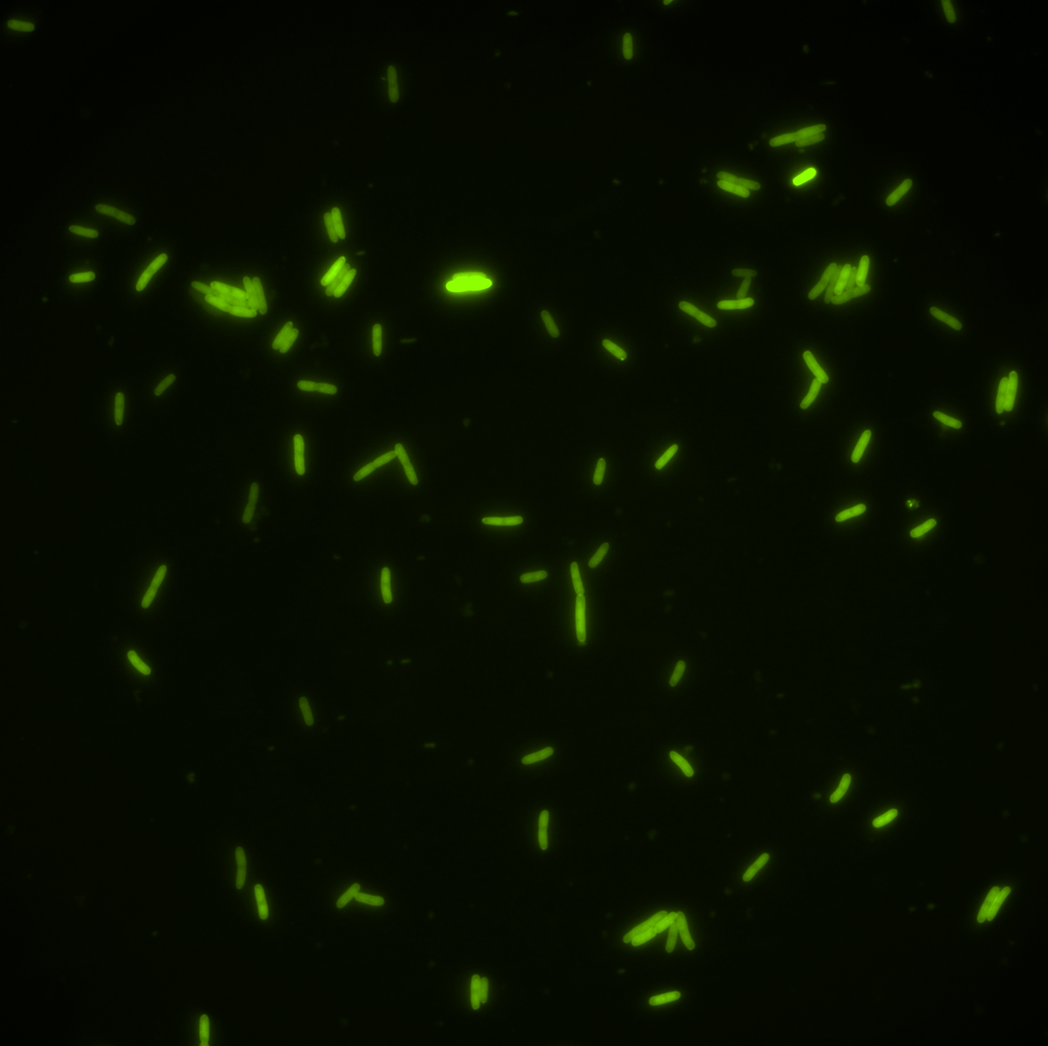

Supplement: Supplementary file 13 — Source data Fig. 1 [file 44321_2025_219_MOESM13_ESM.zip › 1B/RCe849 1_4 saccharin 0 min001_RGB_eYFP.tif]

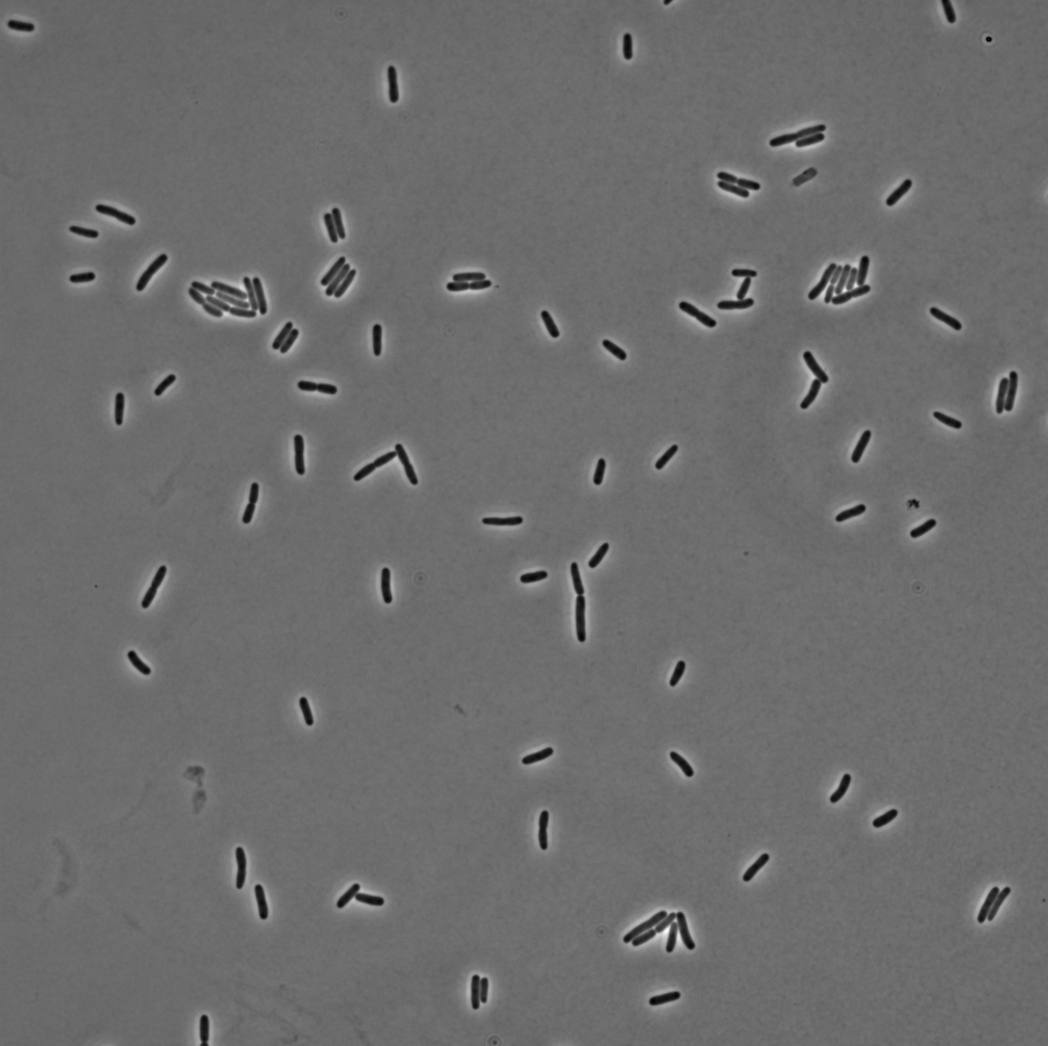

Supplement: Supplementary file 13 — Source data Fig. 1 [file 44321_2025_219_MOESM13_ESM.zip › 1B/RCe849 1_4 saccharin 0 min001_RGB_Brightfield.tif]

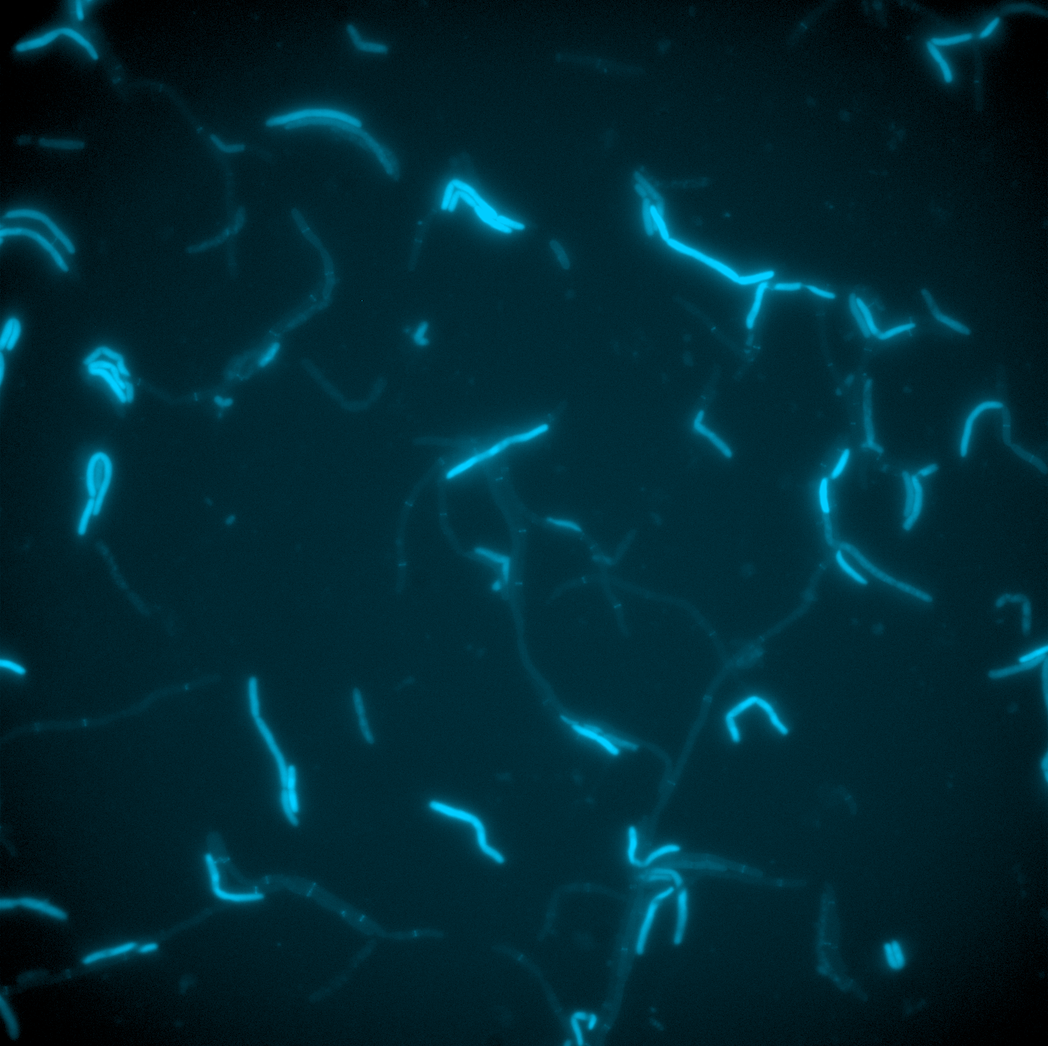

Supplement: Supplementary file 13 — Source data Fig. 1 [file 44321_2025_219_MOESM13_ESM.zip › 1B/RCe849 1_4 saccharin 90 min017_RGB_eCFP.tif]

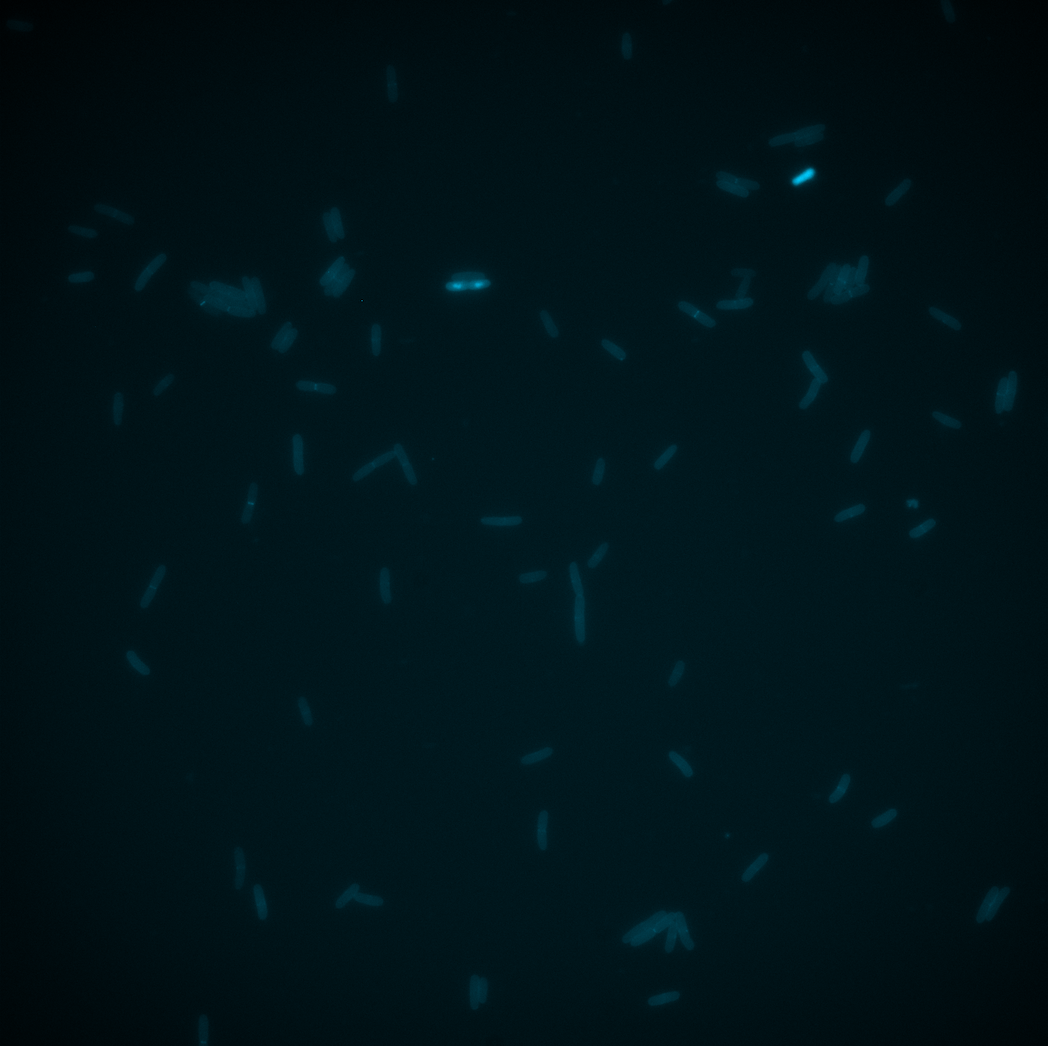

Supplement: Supplementary file 13 — Source data Fig. 1 [file 44321_2025_219_MOESM13_ESM.zip › 1B/RCe849 1_4 saccharin 0 min001_RGB_eCFP.tif]

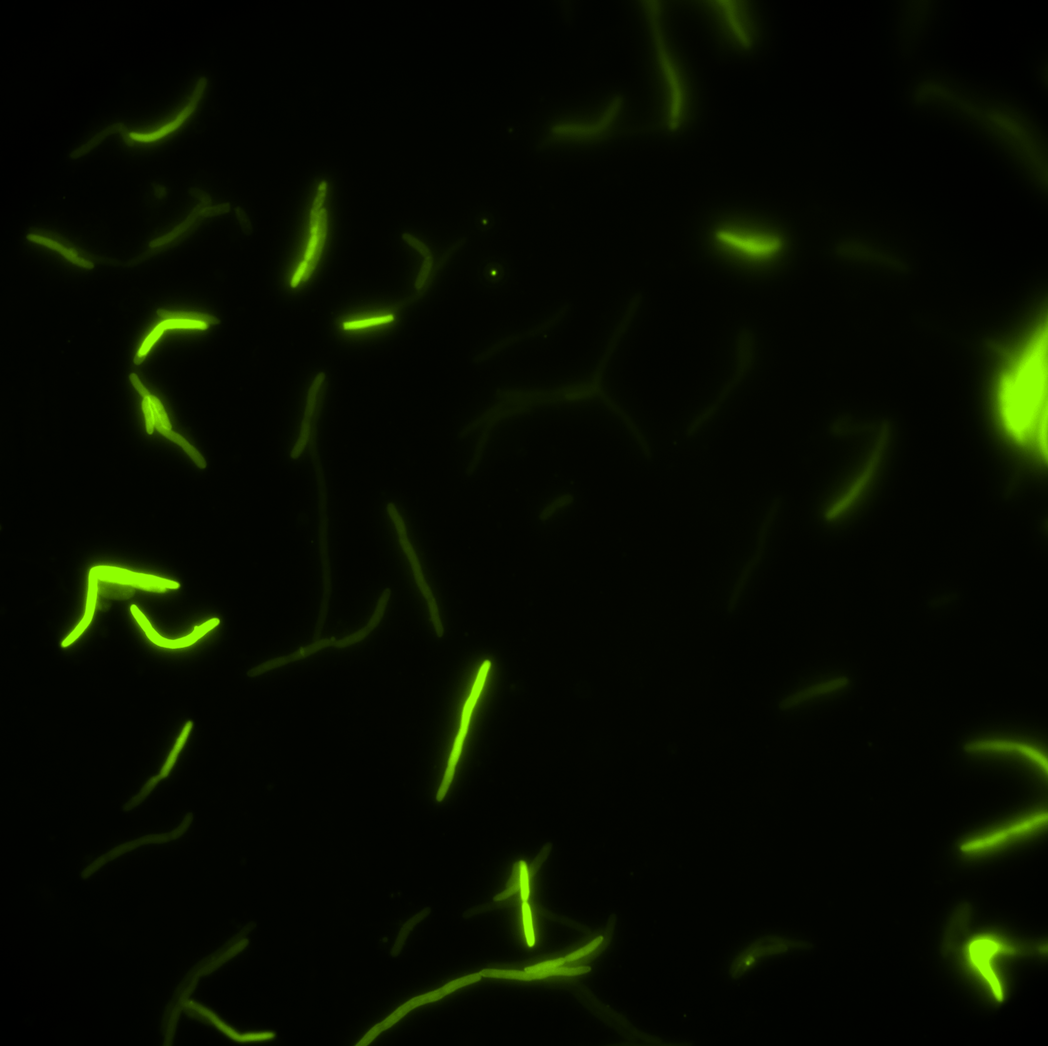

Supplement: Supplementary file 13 — Source data Fig. 1 [file 44321_2025_219_MOESM13_ESM.zip › 1B/RCe849 1_4 saccharin 60 min011_RGB_eYFP.tif]

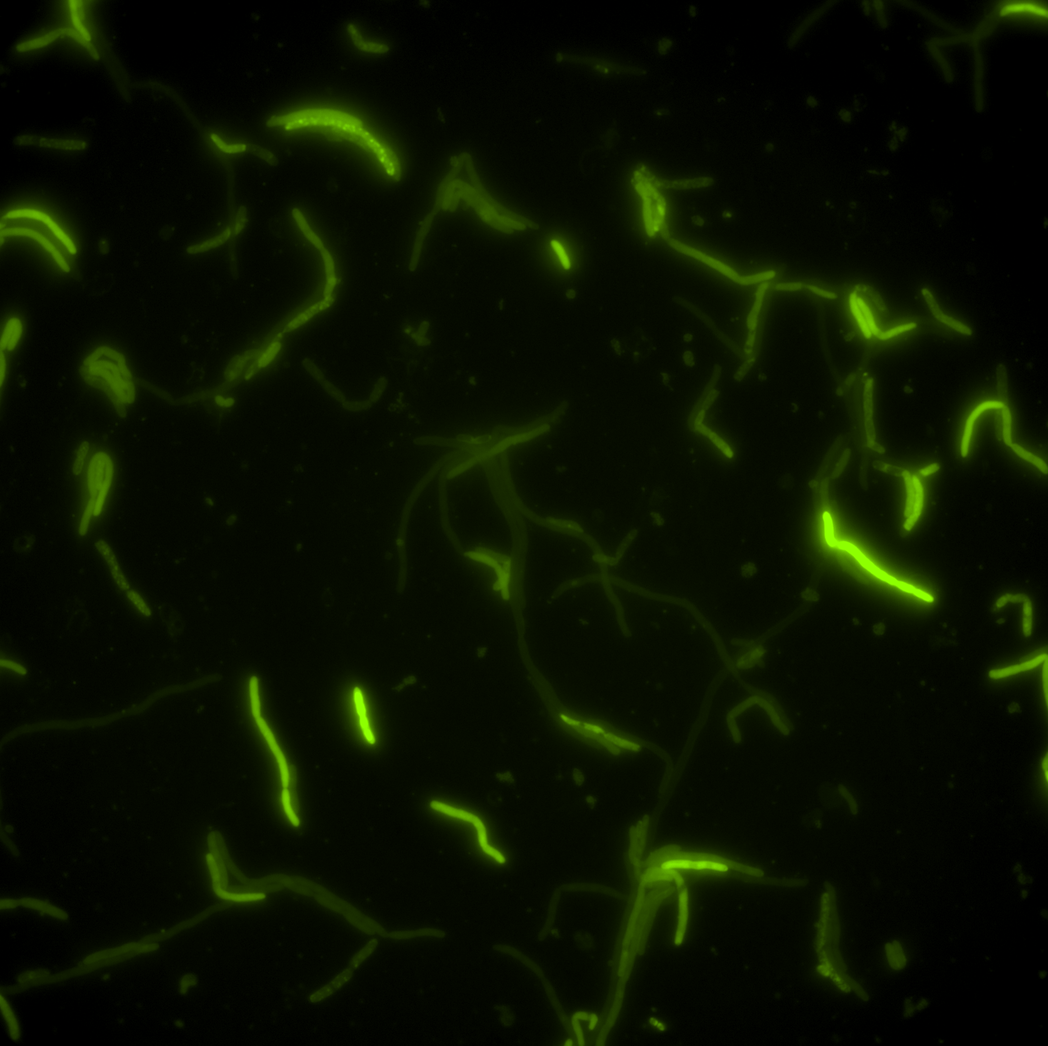

Supplement: Supplementary file 13 — Source data Fig. 1 [file 44321_2025_219_MOESM13_ESM.zip › 1B/RCe849 1_4 saccharin 90 min017_RGB_eYFP.tif]

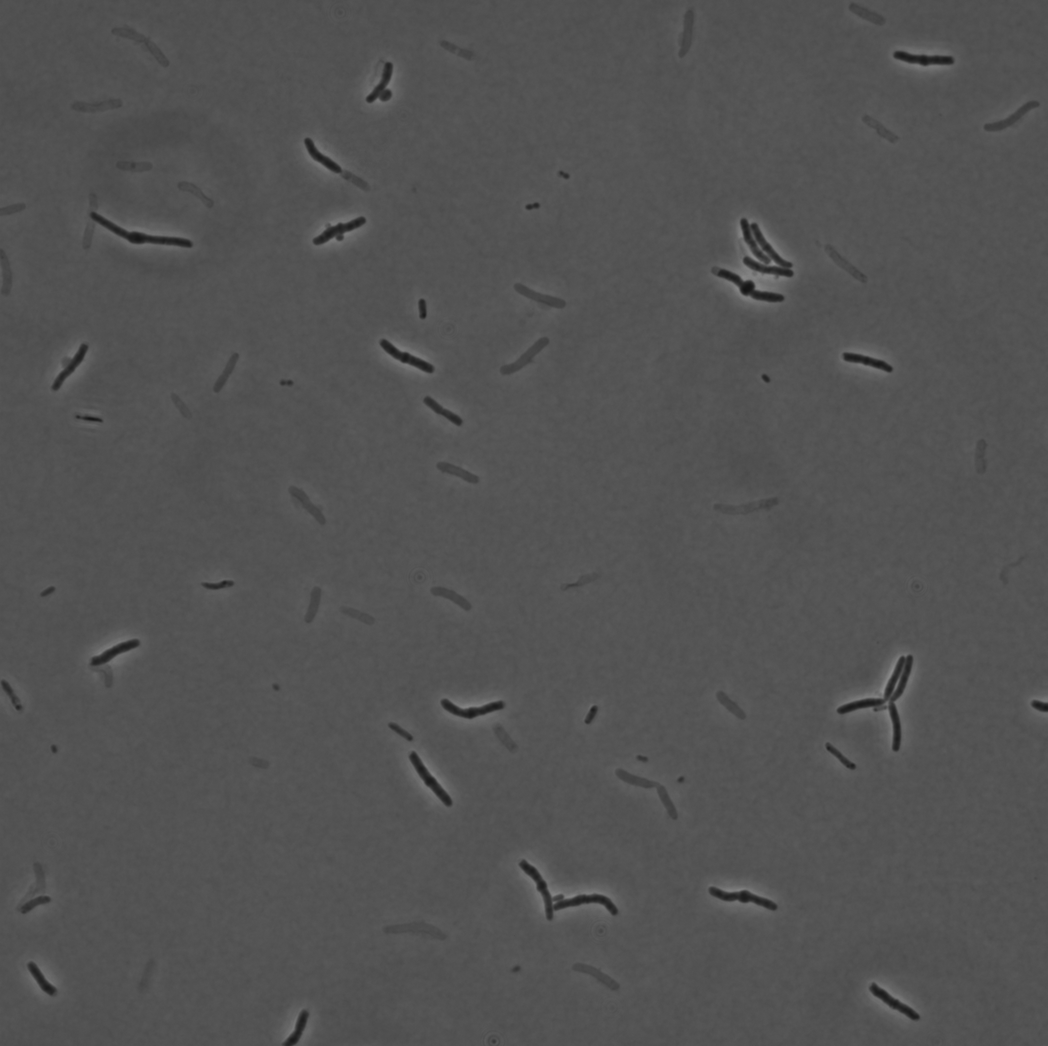

Supplement: Supplementary file 13 — Source data Fig. 1 [file 44321_2025_219_MOESM13_ESM.zip › 1C/MG1655 1_4 saccharin 10 NAO membrane stain008.tif]

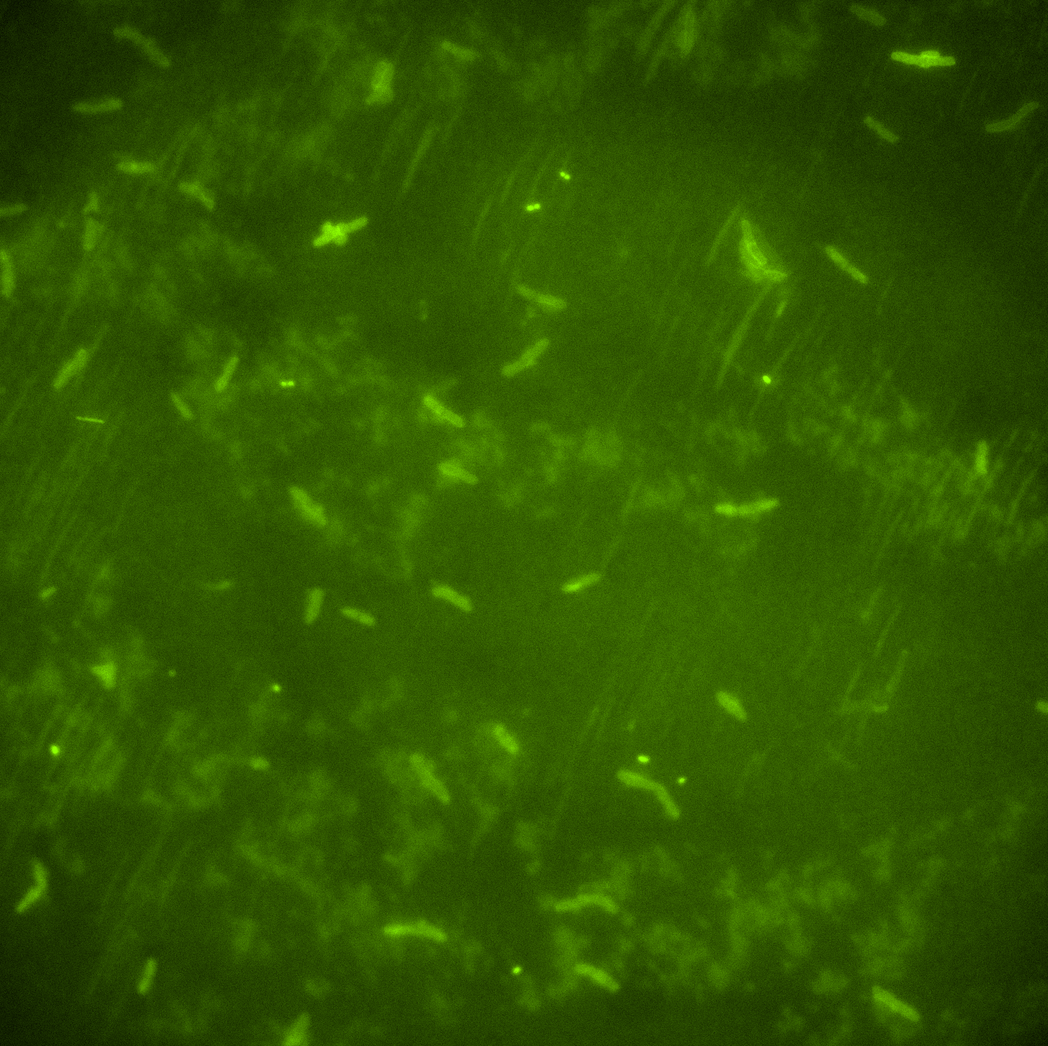

Supplement: Supplementary file 13 — Source data Fig. 1 [file 44321_2025_219_MOESM13_ESM.zip › 1C/MG1655 1_4 saccharin 10 NAO membrane stain015_RGB_eYFP.tif]

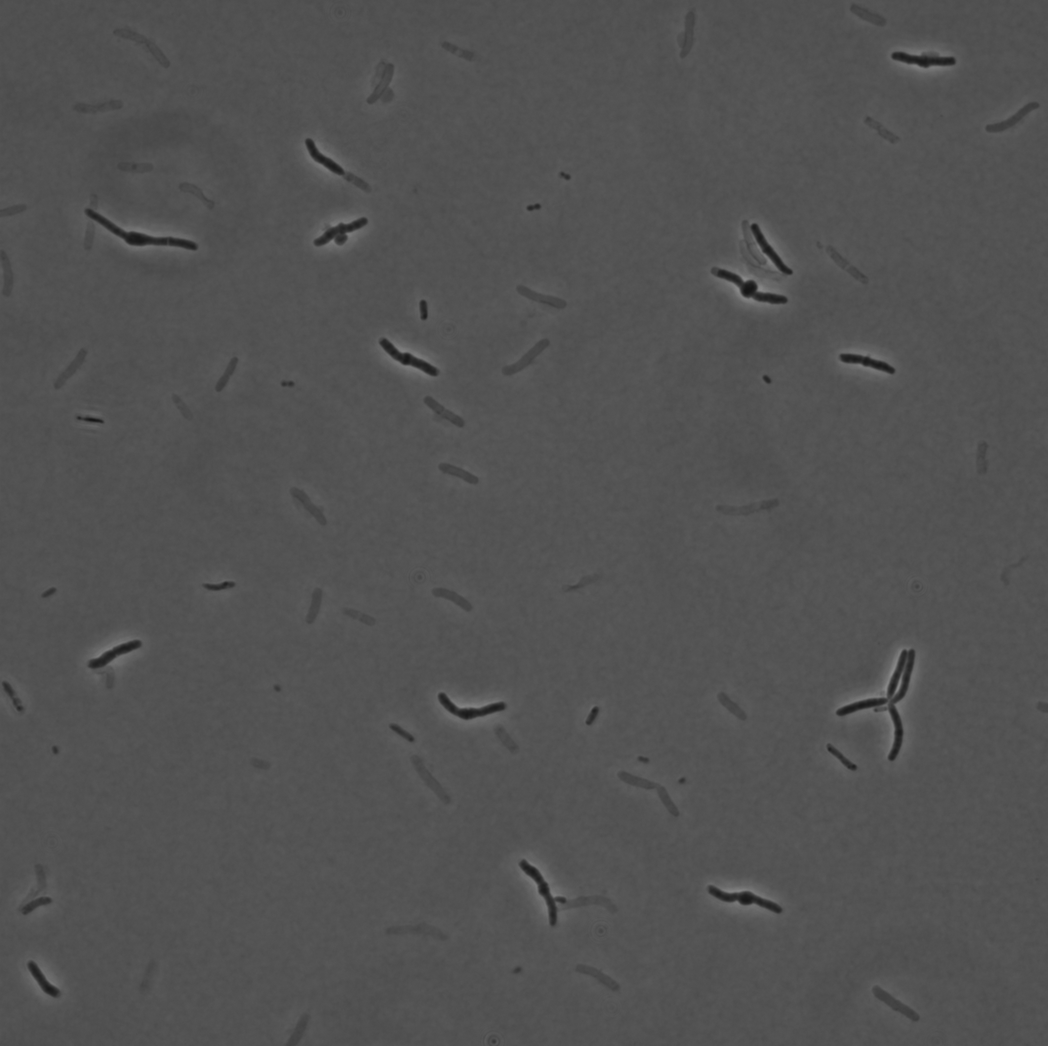

Supplement: Supplementary file 13 — Source data Fig. 1 [file 44321_2025_219_MOESM13_ESM.zip › 1C/MG1655 1_4 saccharin 10 NAO membrane stain015_RGB_Brightfield.tif]

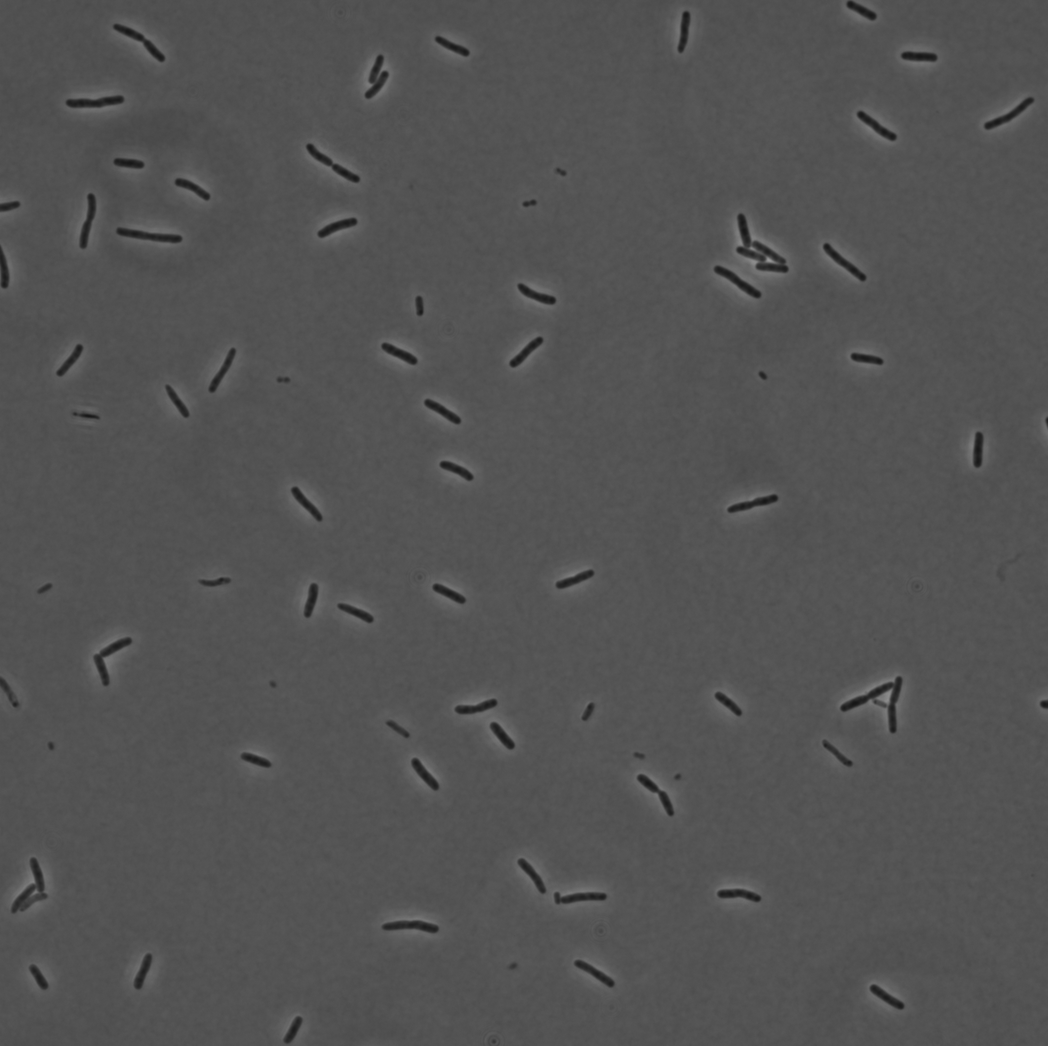

Supplement: Supplementary file 13 — Source data Fig. 1 [file 44321_2025_219_MOESM13_ESM.zip › 1C/MG1655 1_4 saccharin 10 NAO membrane stain005_T2.tif]

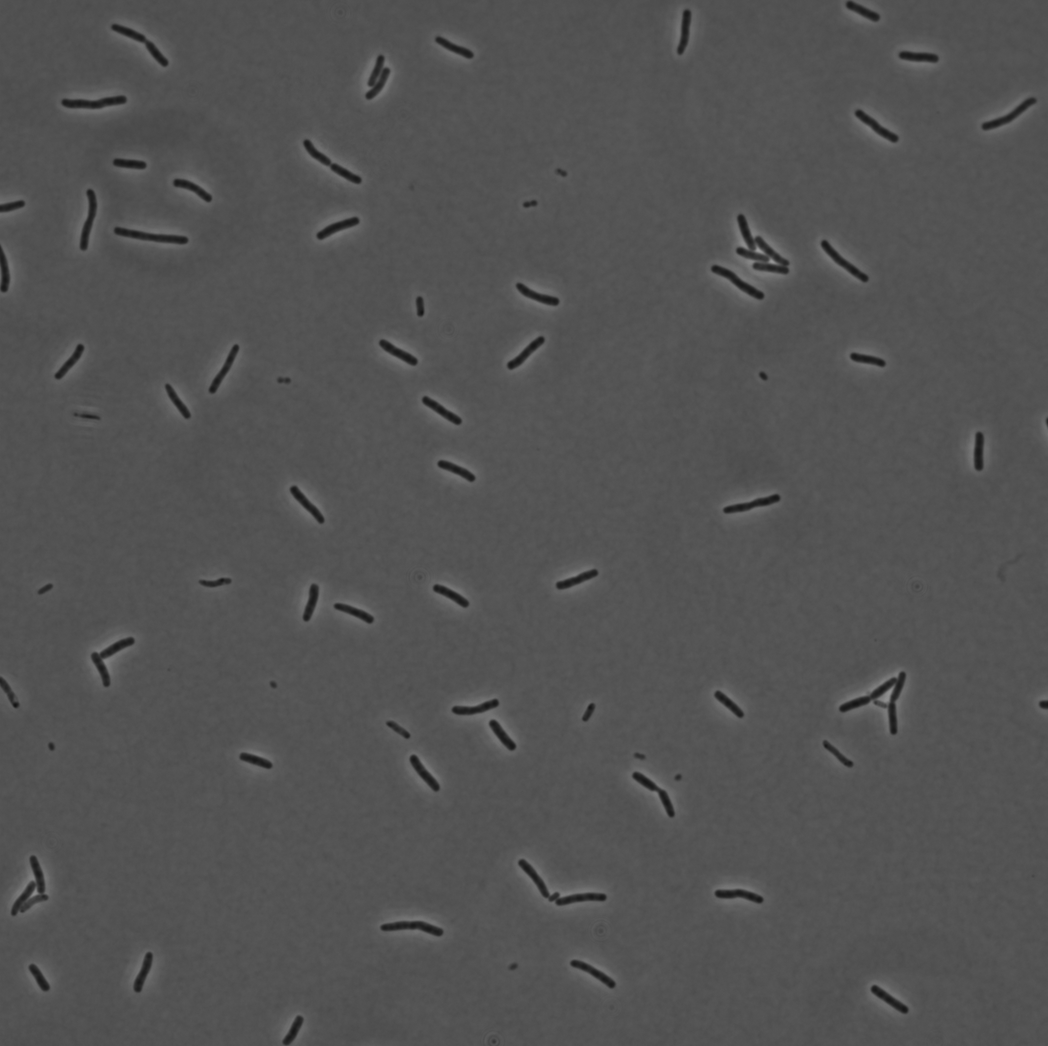

Supplement: Supplementary file 13 — Source data Fig. 1 [file 44321_2025_219_MOESM13_ESM.zip › 1C/MG1655 1_4 saccharin 10 NAO membrane stain005_T3.tif]

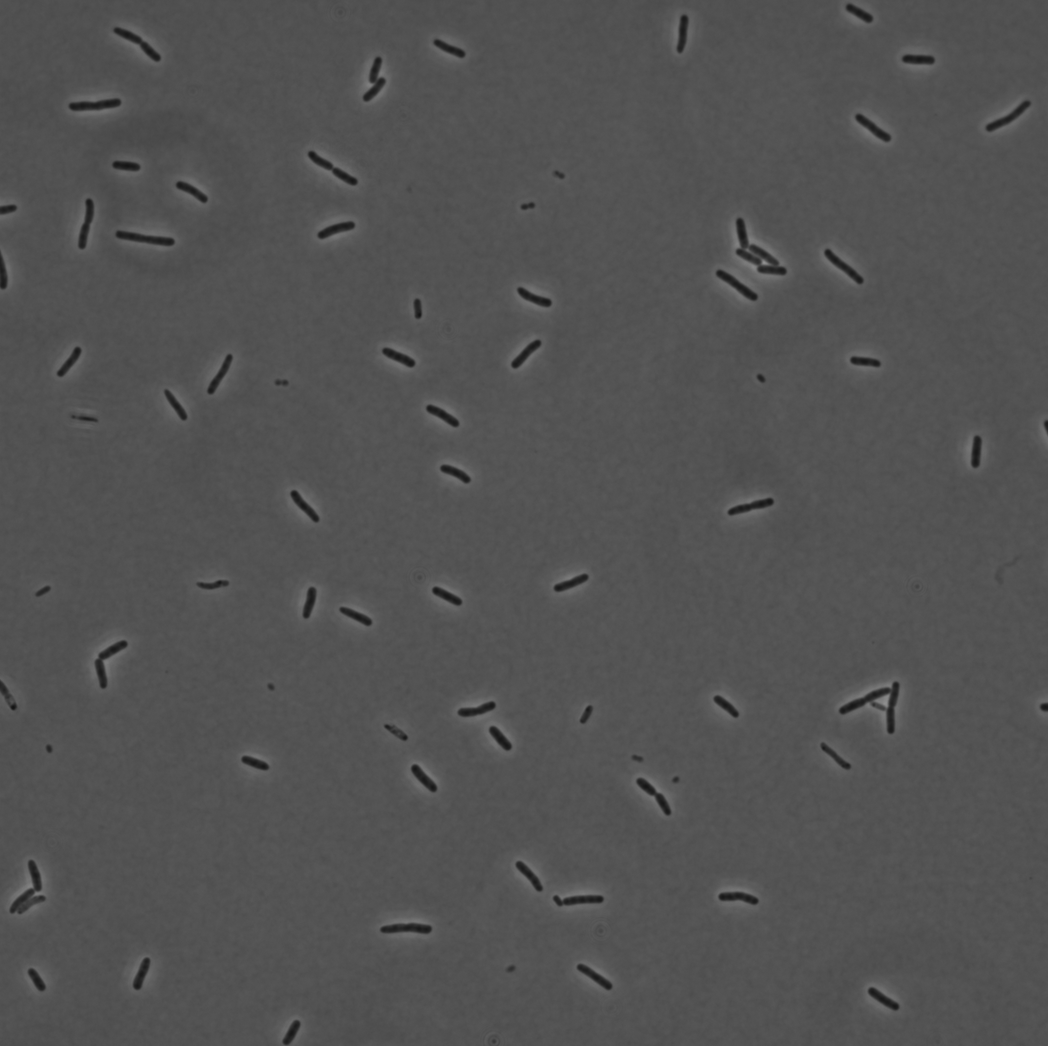

Supplement: Supplementary file 13 — Source data Fig. 1 [file 44321_2025_219_MOESM13_ESM.zip › 1C/MG1655 1_4 saccharin 10 NAO membrane stain005_T1.tif]

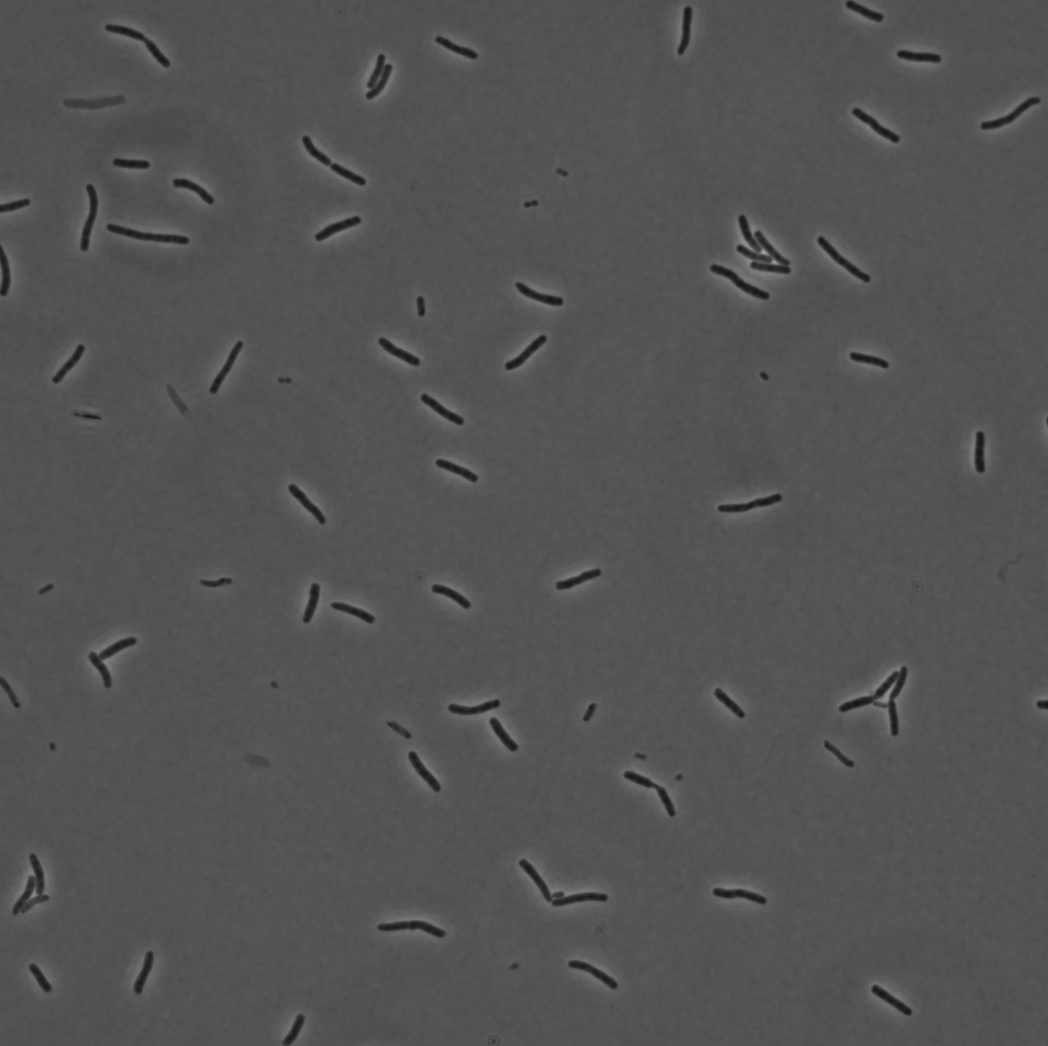

Supplement: Supplementary file 13 — Source data Fig. 1 [file 44321_2025_219_MOESM13_ESM.zip › 1C/MG1655 1_4 saccharin 10 NAO membrane stain005_T4.tif]

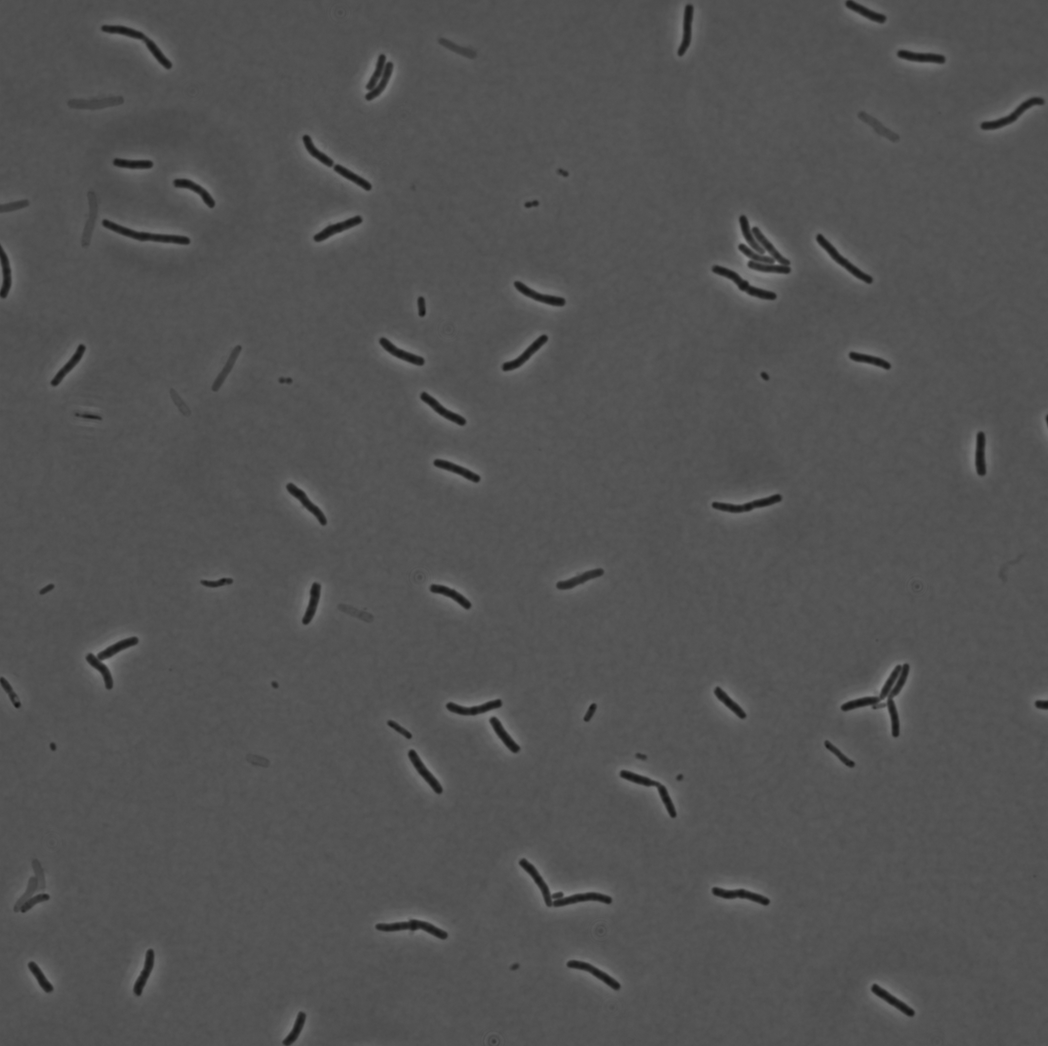

Supplement: Supplementary file 13 — Source data Fig. 1 [file 44321_2025_219_MOESM13_ESM.zip › 1C/MG1655 1_4 saccharin 10 NAO membrane stain005_T5.tif]

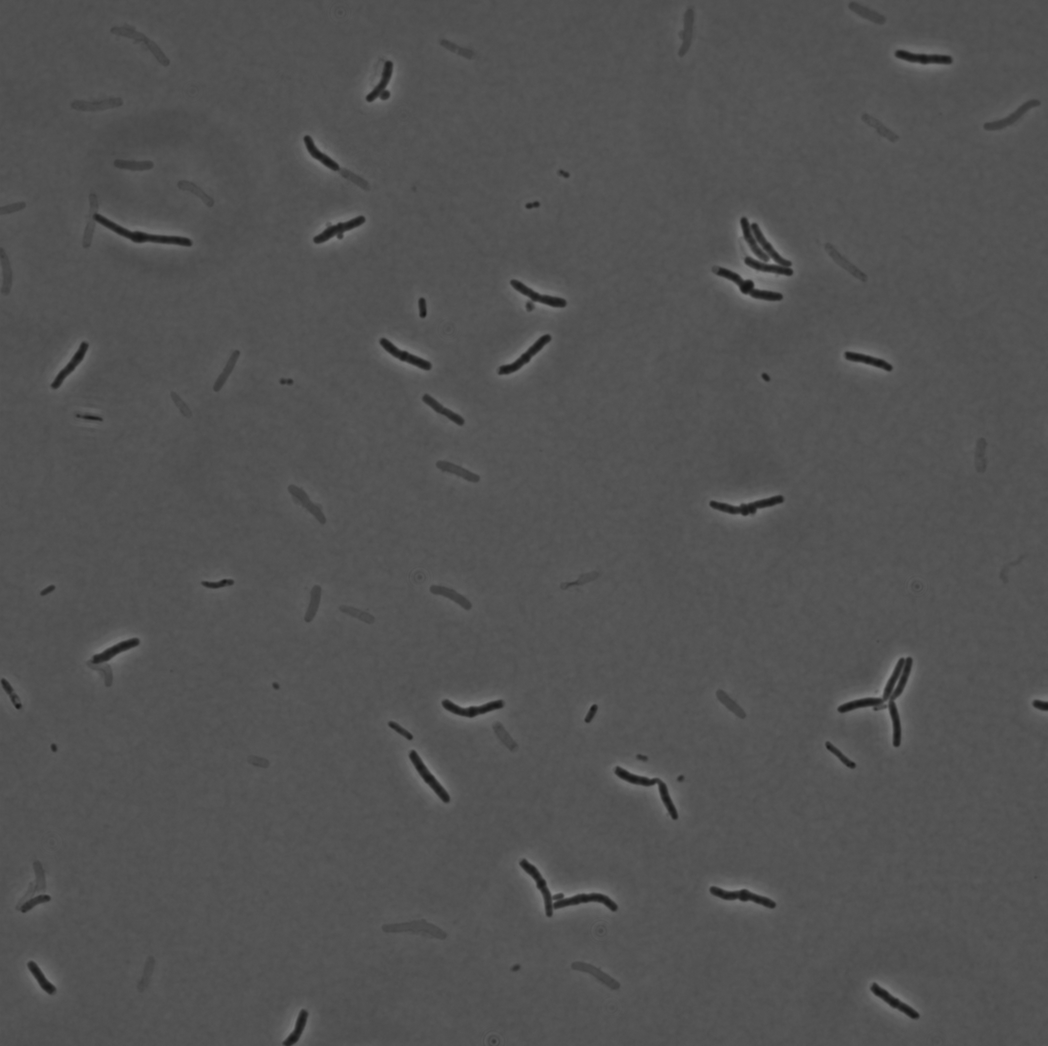

Supplement: Supplementary file 13 — Source data Fig. 1 [file 44321_2025_219_MOESM13_ESM.zip › 1C/MG1655 1_4 saccharin 10 NAO membrane stain005_T7.tif]

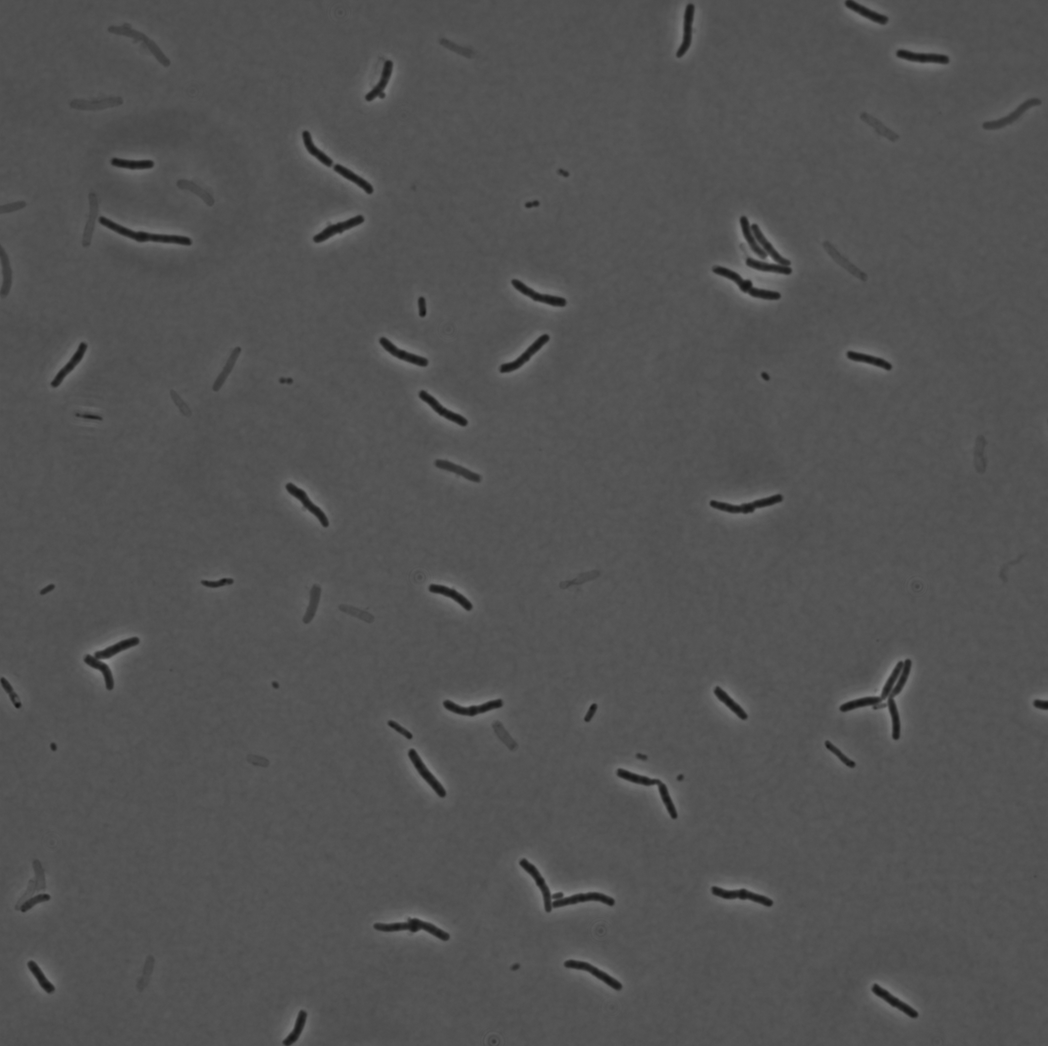

Supplement: Supplementary file 13 — Source data Fig. 1 [file 44321_2025_219_MOESM13_ESM.zip › 1C/MG1655 1_4 saccharin 10 NAO membrane stain005_T6.tif]

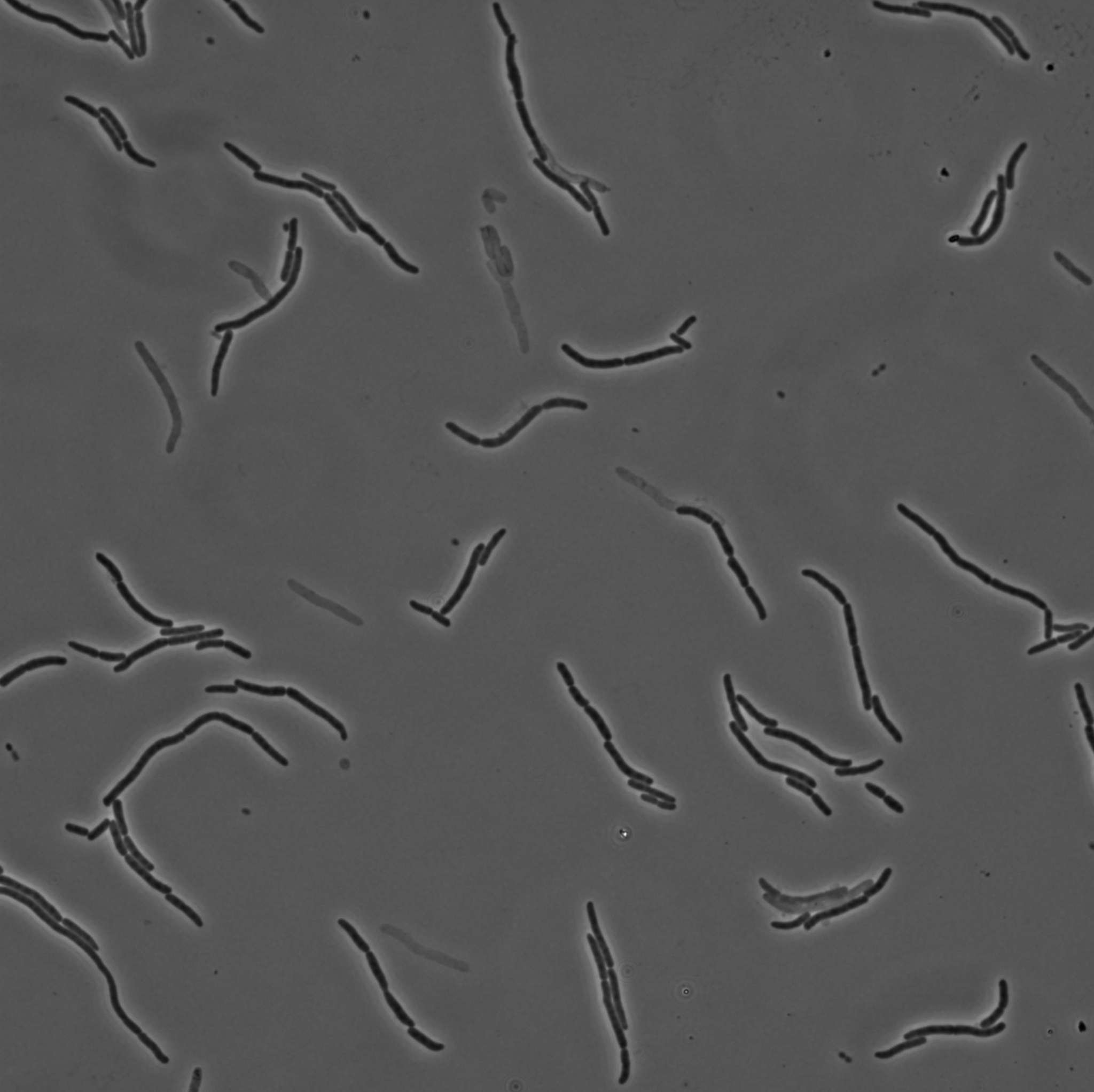

Supplement: Supplementary file 14 — Source data Fig. 2 [file 44321_2025_219_MOESM14_ESM.zip › Figure 2/2A/RCe853 1_4 saccharin TL1021_RGB_Brightfield.tif]

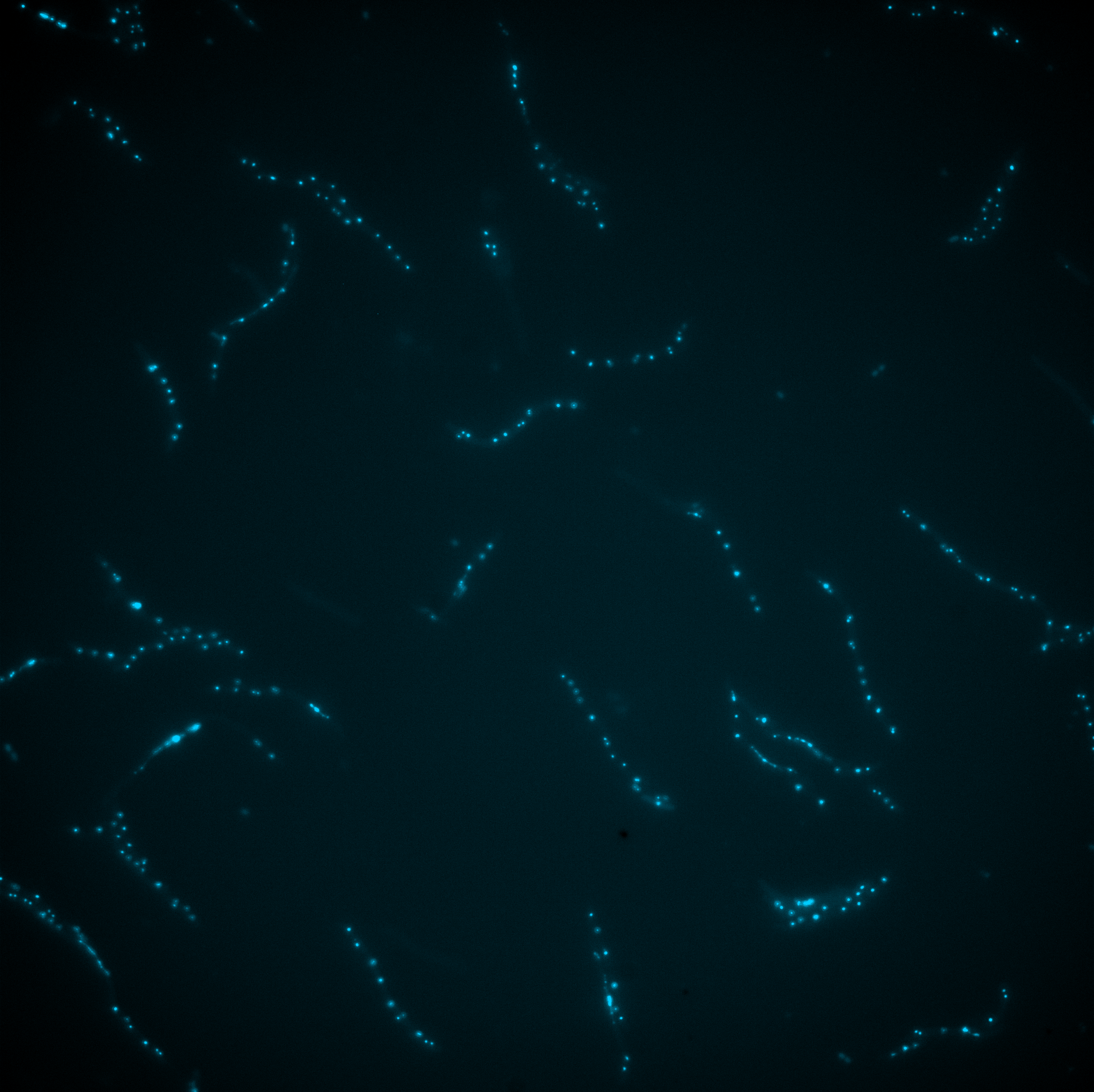

Supplement: Supplementary file 14 — Source data Fig. 2 [file 44321_2025_219_MOESM14_ESM.zip › Figure 2/2A/RCe853 1_4 saccharin TL1021_RGB_eCFP.tif]

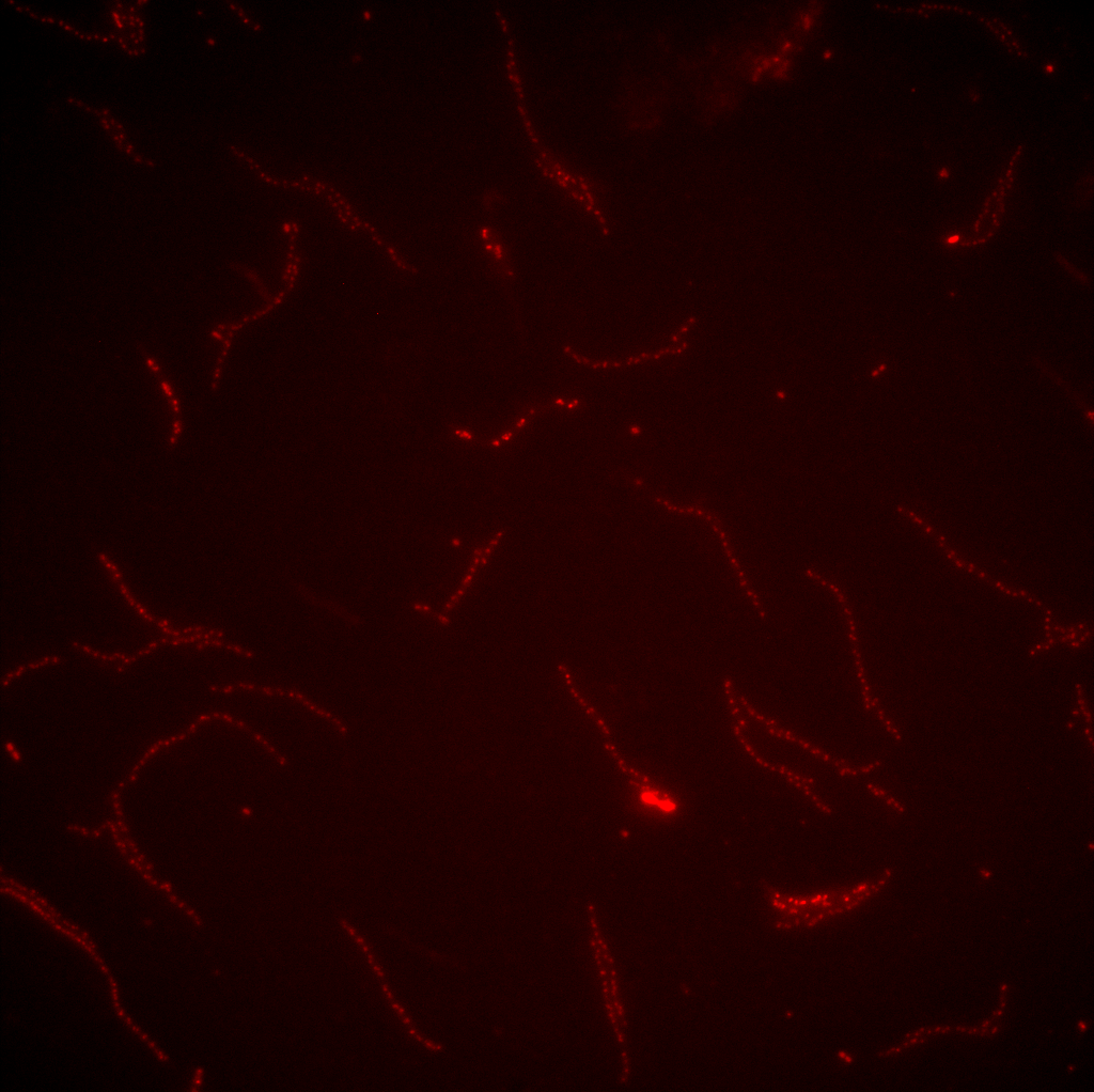

Supplement: Supplementary file 14 — Source data Fig. 2 [file 44321_2025_219_MOESM14_ESM.zip › Figure 2/2A/RCe853 1_4 saccharin TL1021_RGB_mCherry.tif]

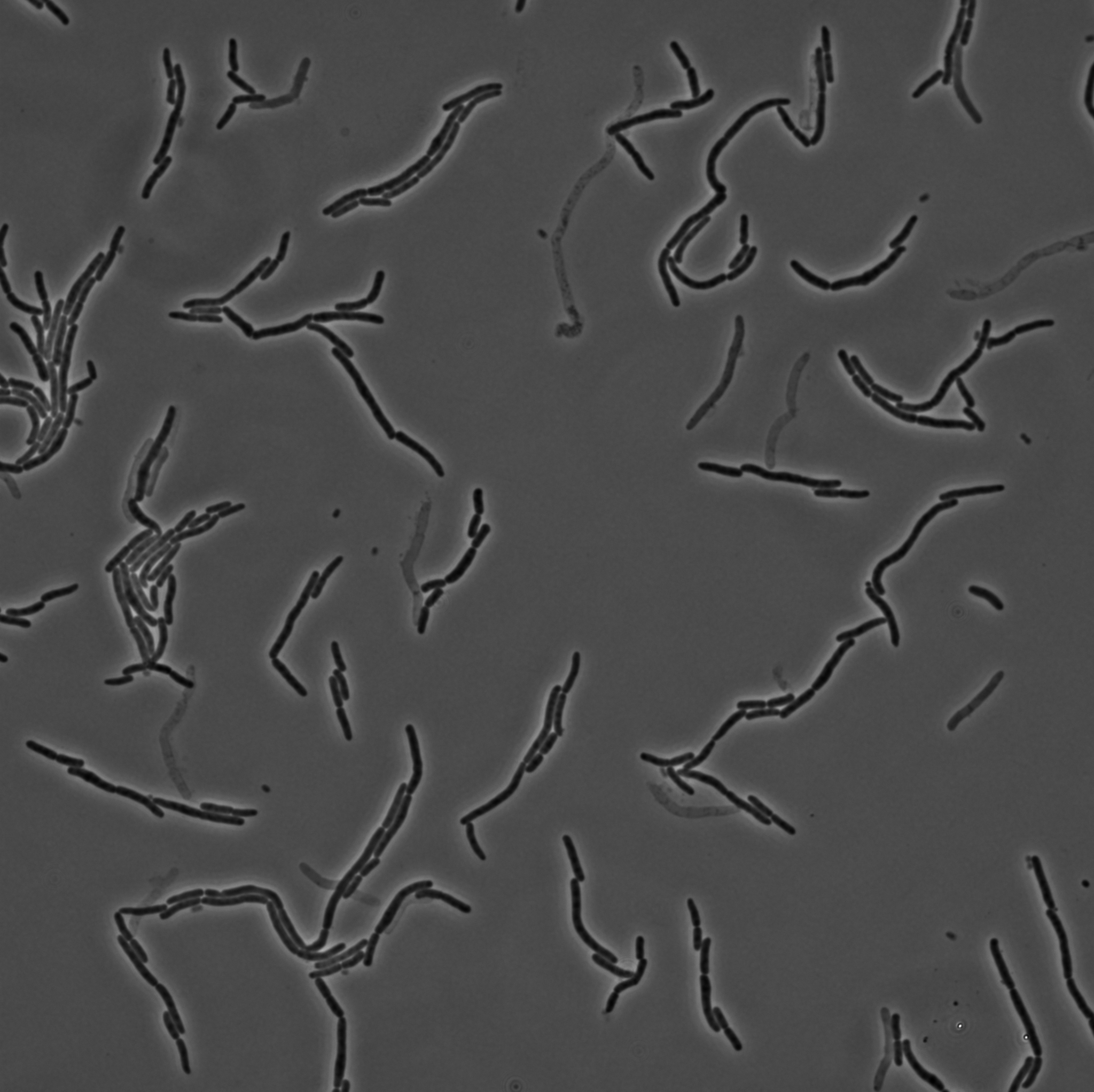

Supplement: Supplementary file 14 — Source data Fig. 2 [file 44321_2025_219_MOESM14_ESM.zip › Figure 2/2A/RCe853 1_4 saccharin TL1022_RGB_Brightfield.tif]

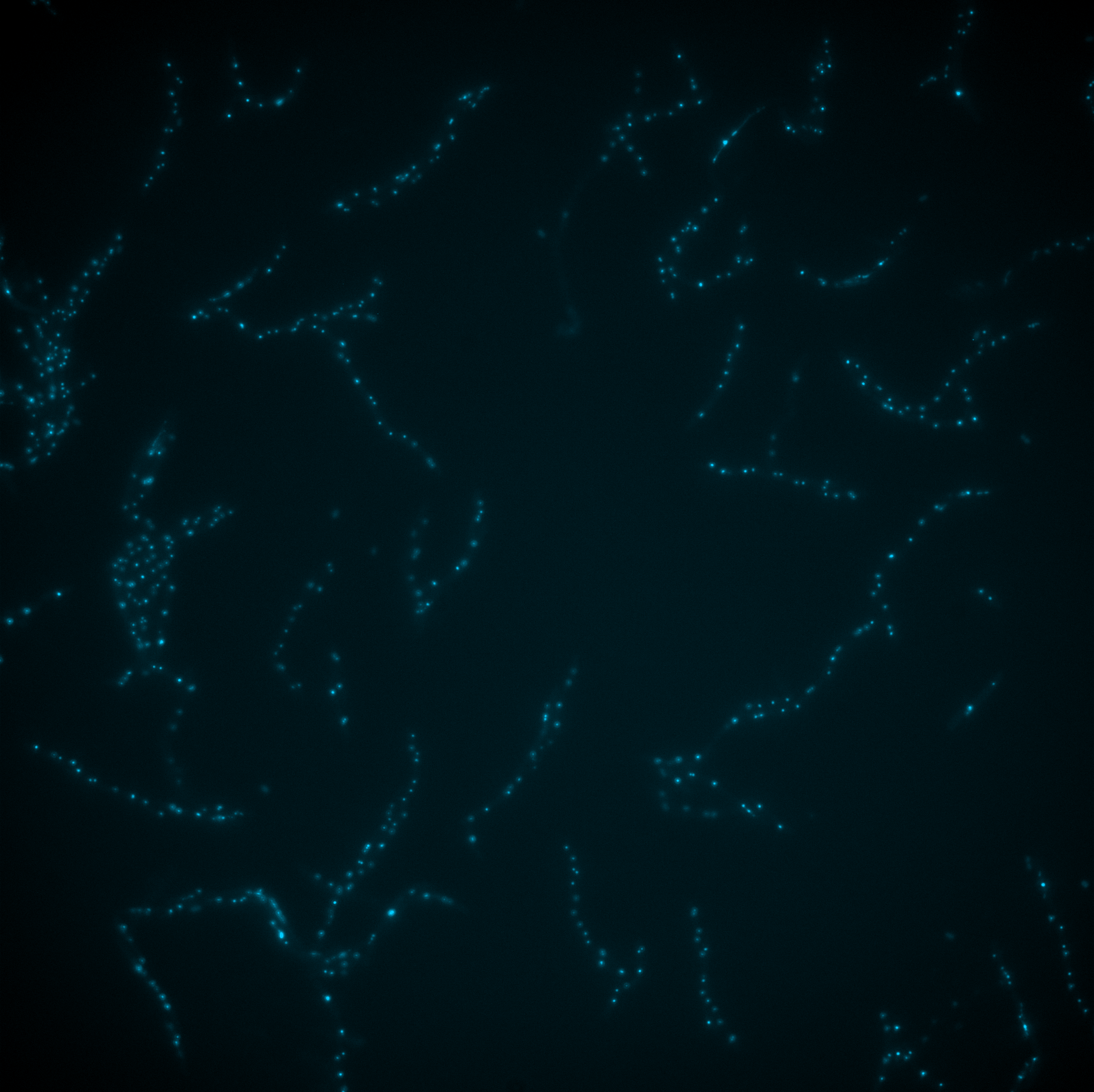

Supplement: Supplementary file 14 — Source data Fig. 2 [file 44321_2025_219_MOESM14_ESM.zip › Figure 2/2A/RCe853 1_4 saccharin TL1022_RGB_eCFP.tif]

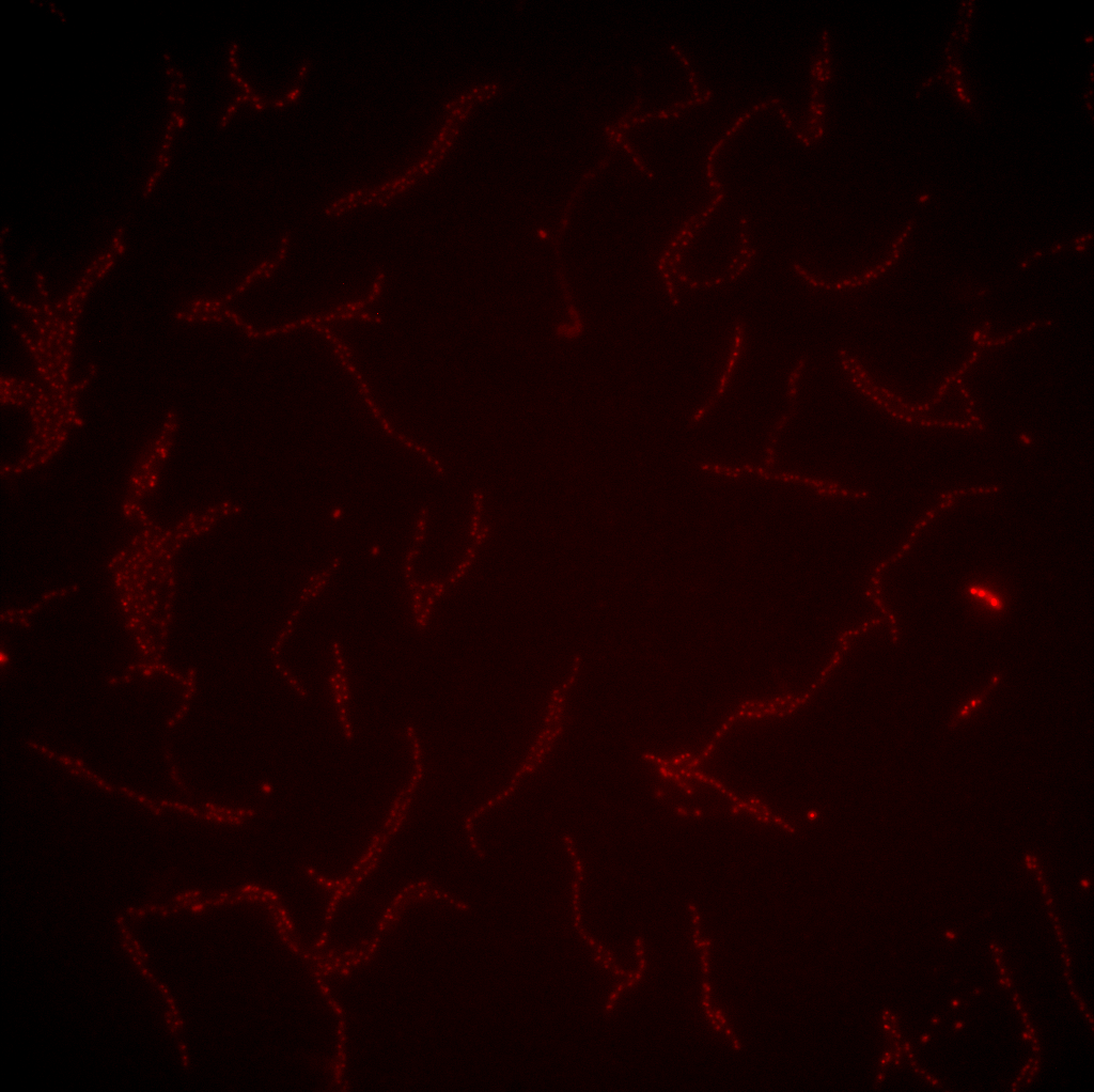

Supplement: Supplementary file 14 — Source data Fig. 2 [file 44321_2025_219_MOESM14_ESM.zip › Figure 2/2A/RCe853 1_4 saccharin TL1022_RGB_mCherry.tif]

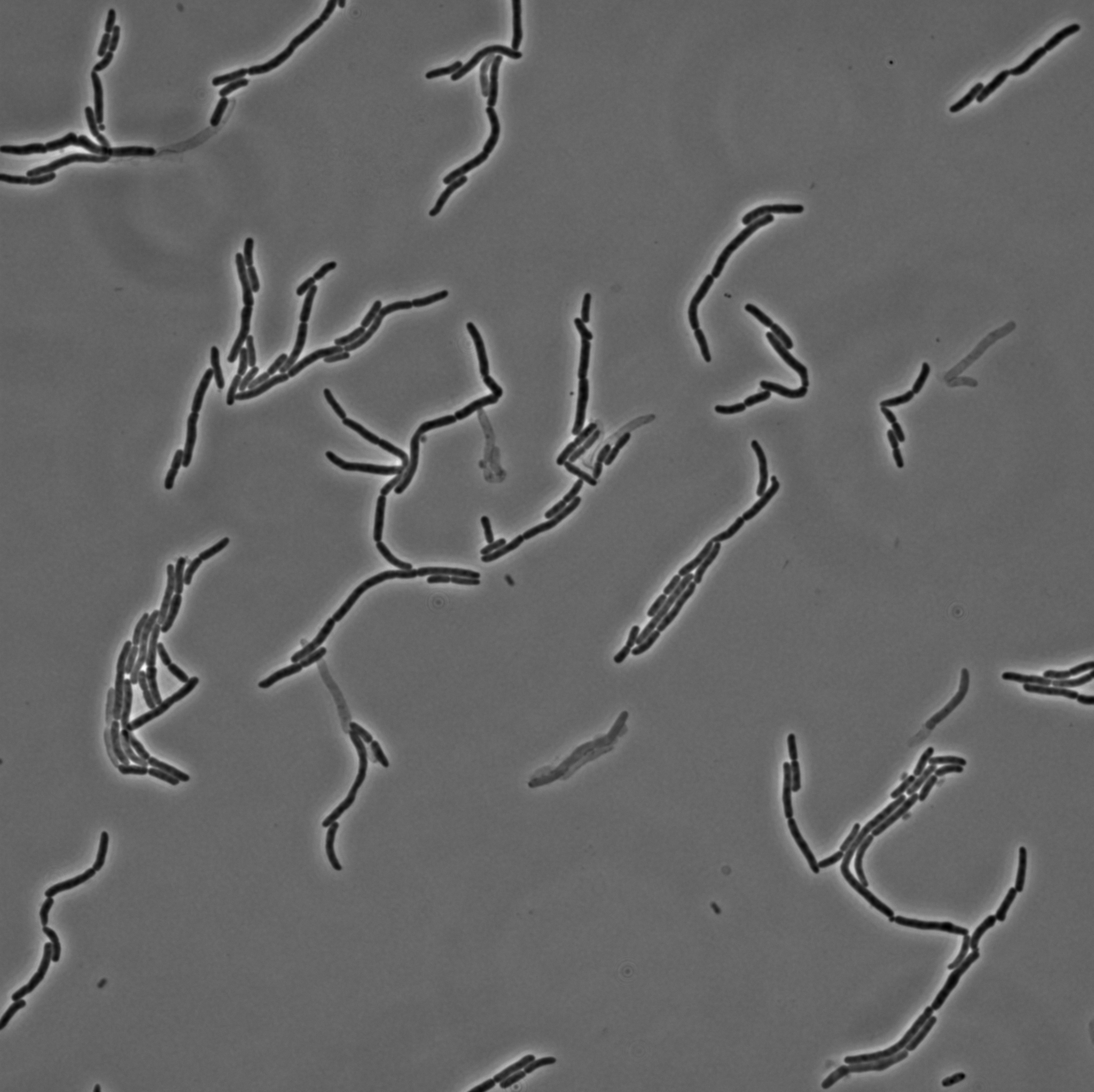

Supplement: Supplementary file 14 — Source data Fig. 2 [file 44321_2025_219_MOESM14_ESM.zip › Figure 2/2A/RCe853 1_4 saccharin TL1023_RGB_Brightfield.tif]

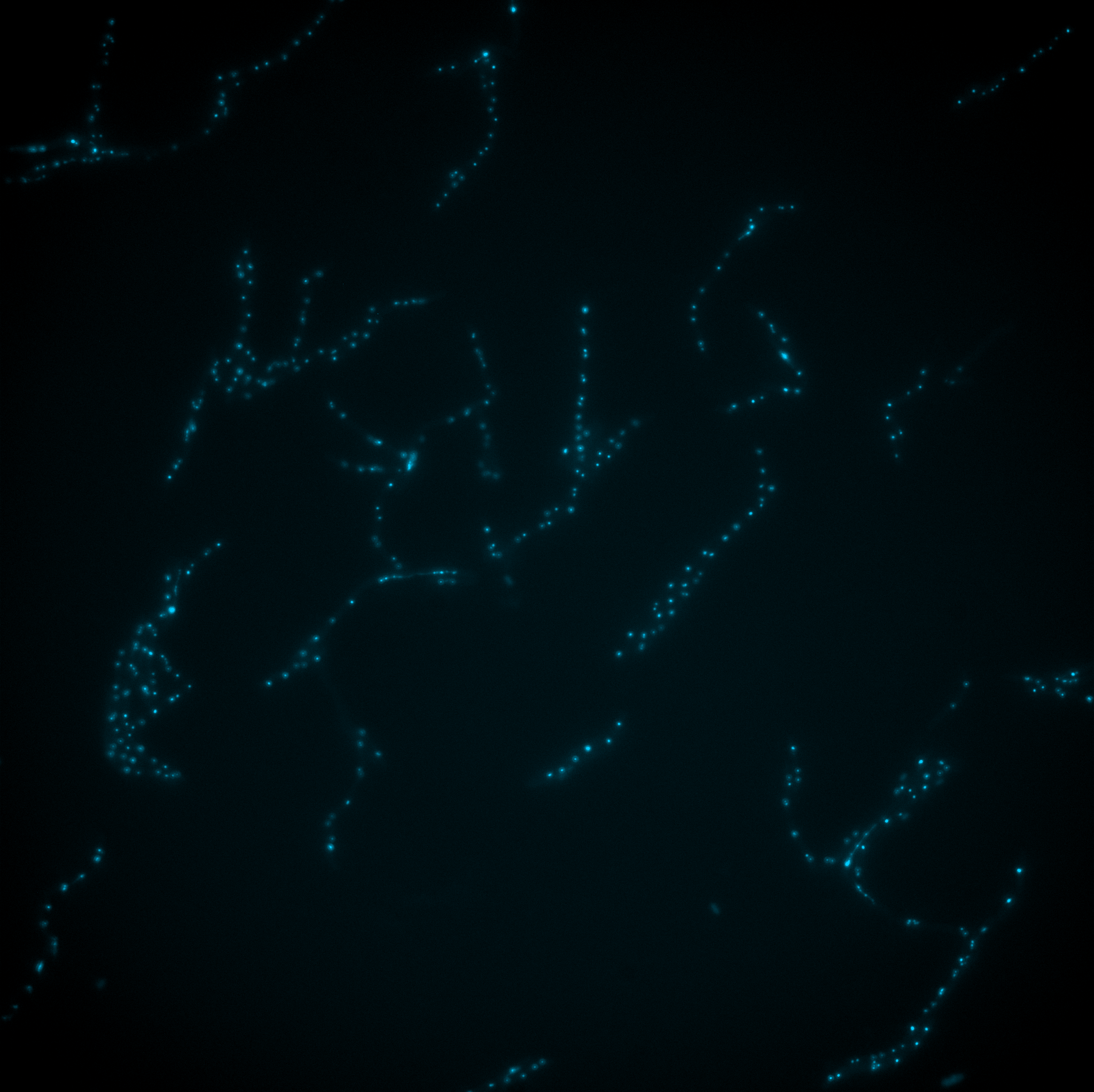

Supplement: Supplementary file 14 — Source data Fig. 2 [file 44321_2025_219_MOESM14_ESM.zip › Figure 2/2A/RCe853 1_4 saccharin TL1023_RGB_eCFP.tif]

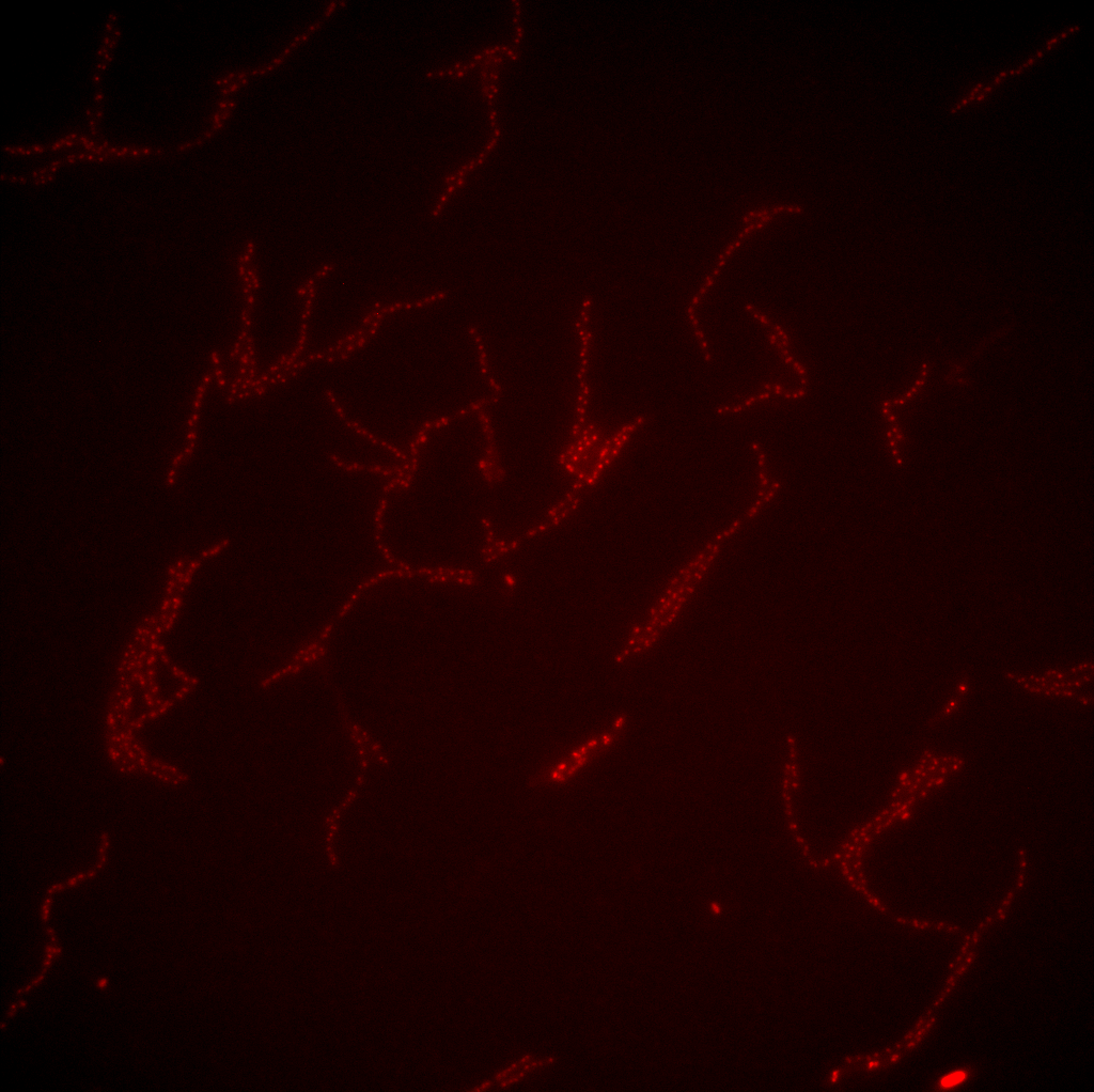

Supplement: Supplementary file 14 — Source data Fig. 2 [file 44321_2025_219_MOESM14_ESM.zip › Figure 2/2A/RCe853 1_4 saccharin TL1023_RGB_mCherry.tif]

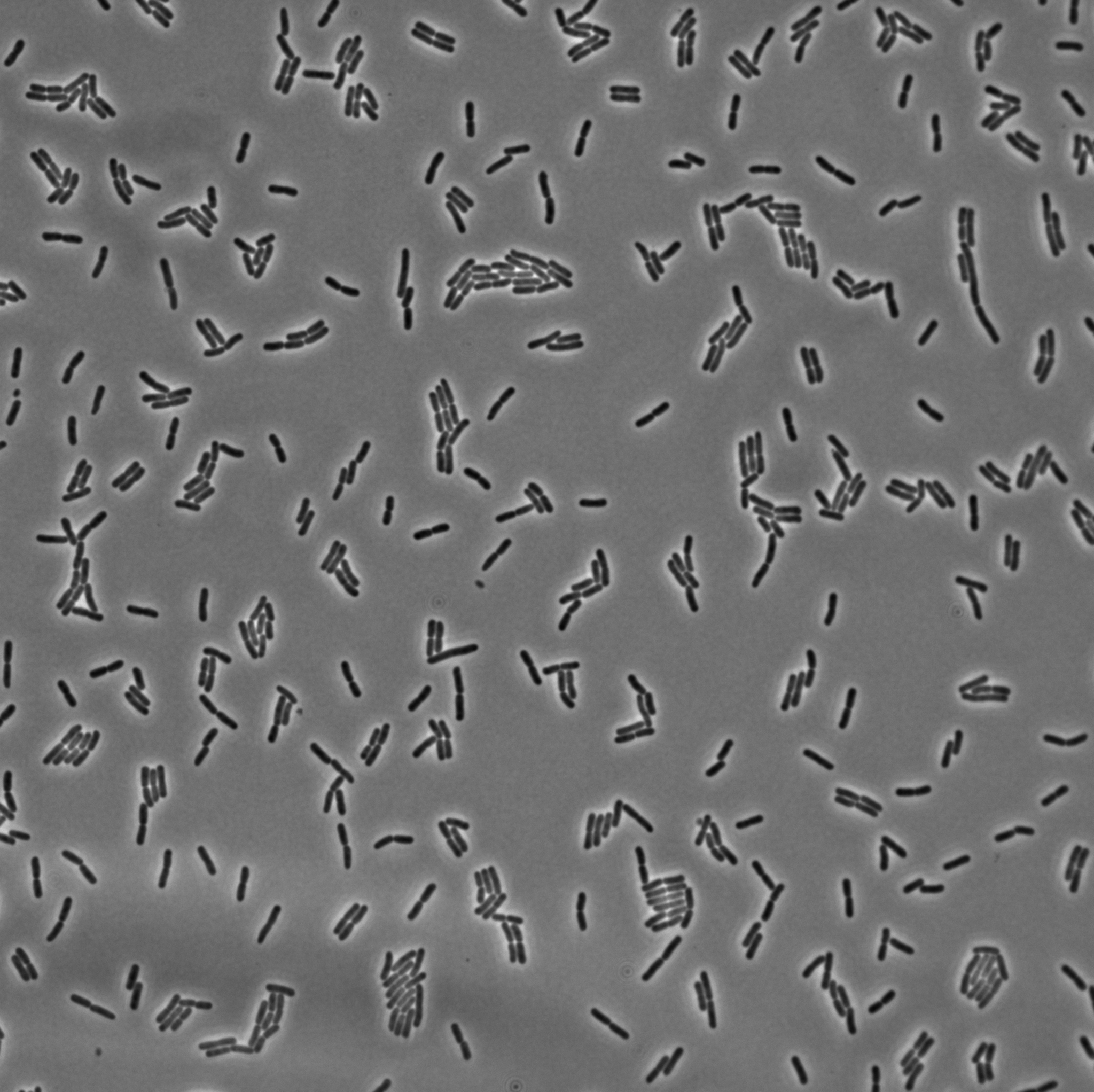

Supplement: Supplementary file 14 — Source data Fig. 2 [file 44321_2025_219_MOESM14_ESM.zip › Figure 2/2A/RCe853 no saccharin TL1017_RGB_Brightfield.tif]

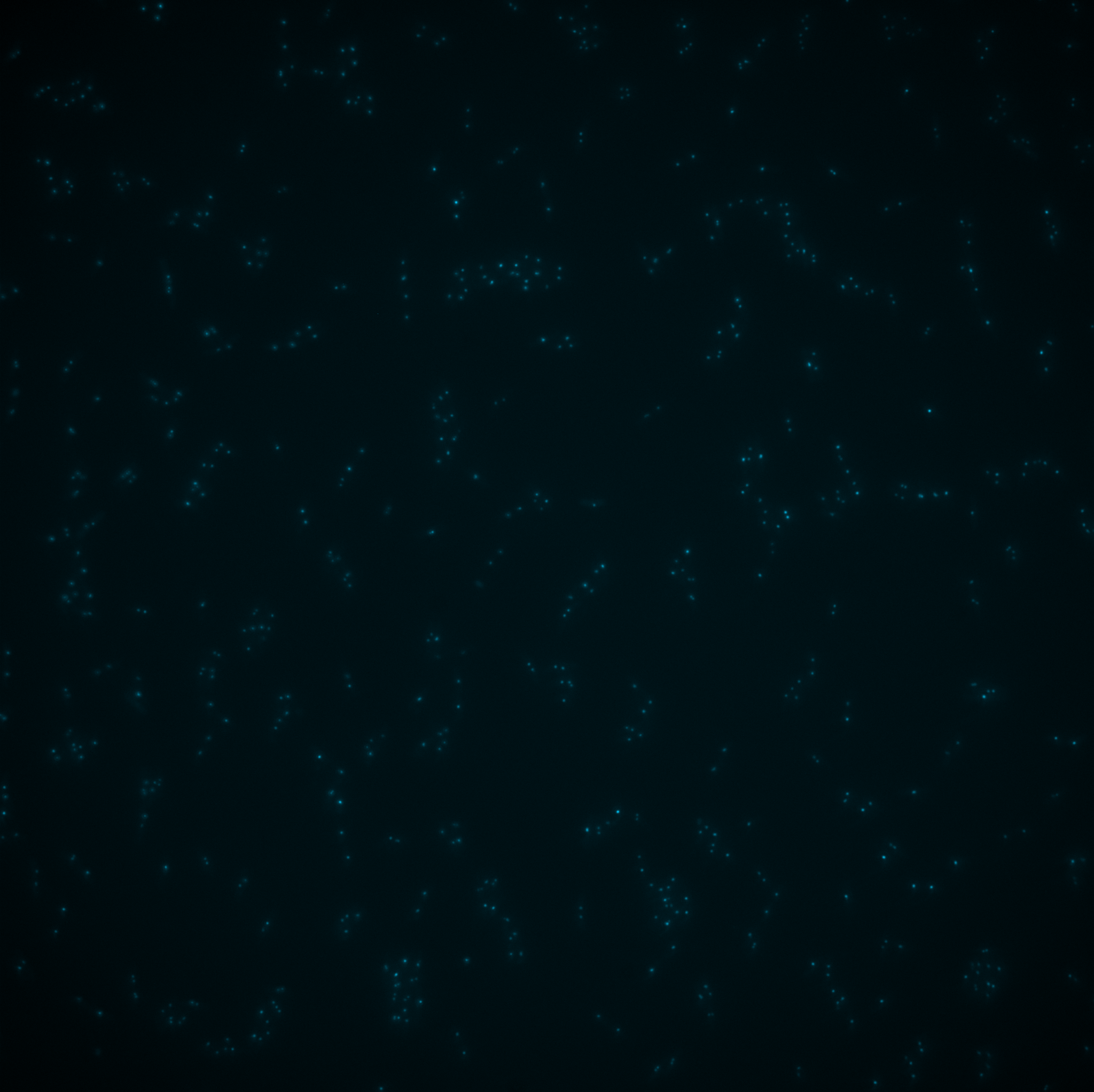

Supplement: Supplementary file 14 — Source data Fig. 2 [file 44321_2025_219_MOESM14_ESM.zip › Figure 2/2A/RCe853 no saccharin TL1017_RGB_eCFP.tif]

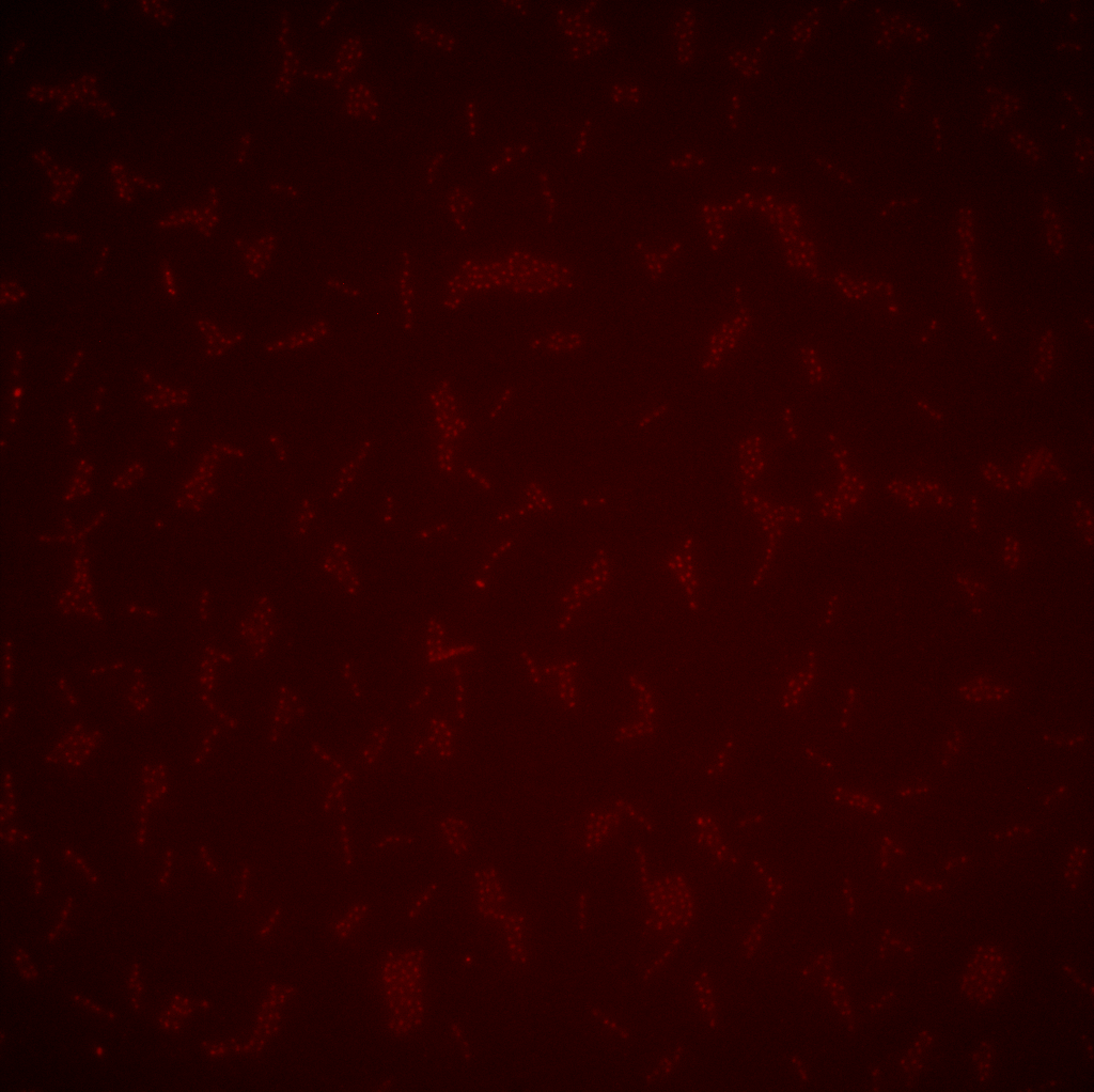

Supplement: Supplementary file 14 — Source data Fig. 2 [file 44321_2025_219_MOESM14_ESM.zip › Figure 2/2A/RCe853 no saccharin TL1017_RGB_mCherry.tif]

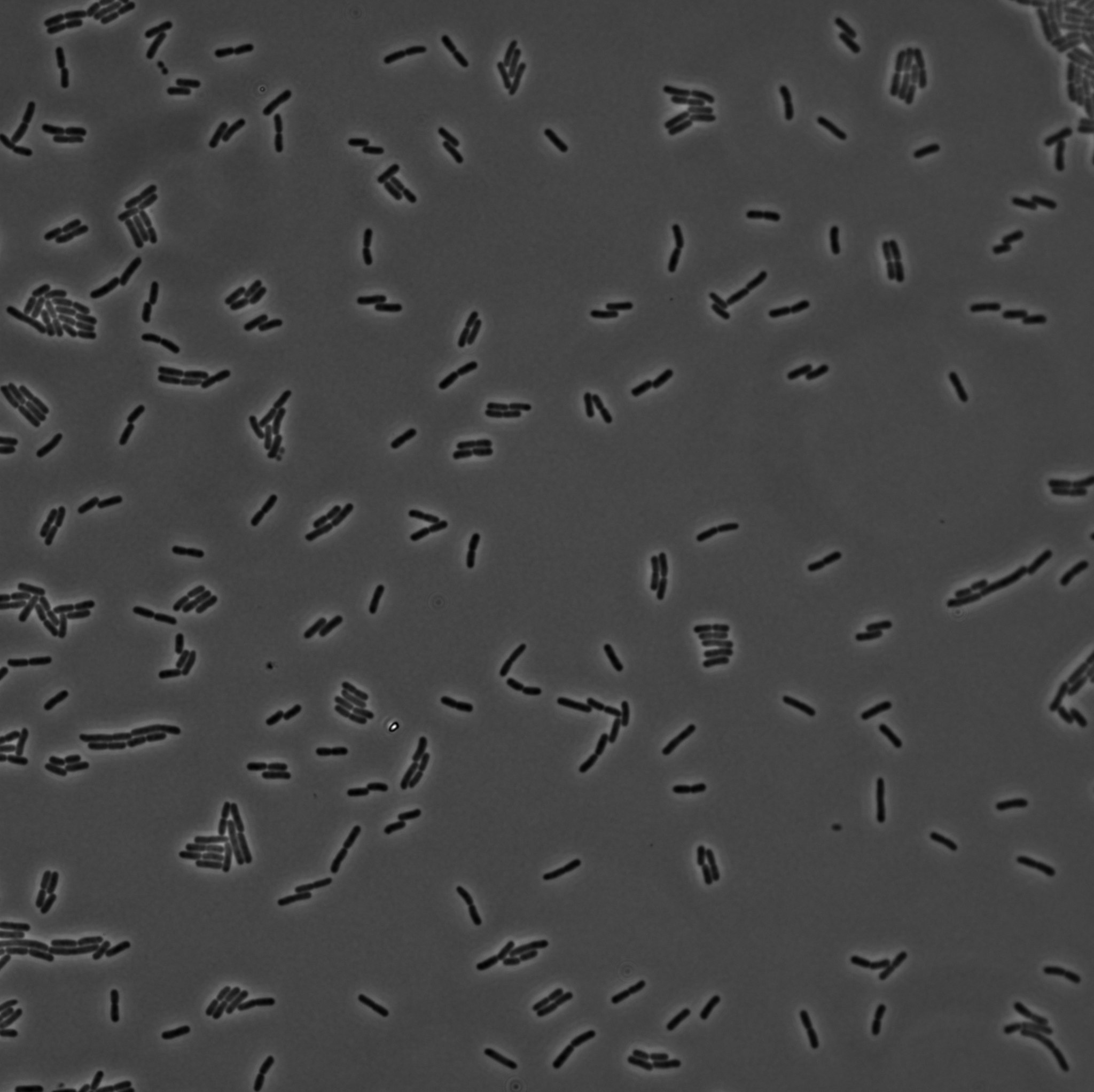

Supplement: Supplementary file 14 — Source data Fig. 2 [file 44321_2025_219_MOESM14_ESM.zip › Figure 2/2A/RCe853 no saccharin TL1018_RGB_Brightfield.tif]

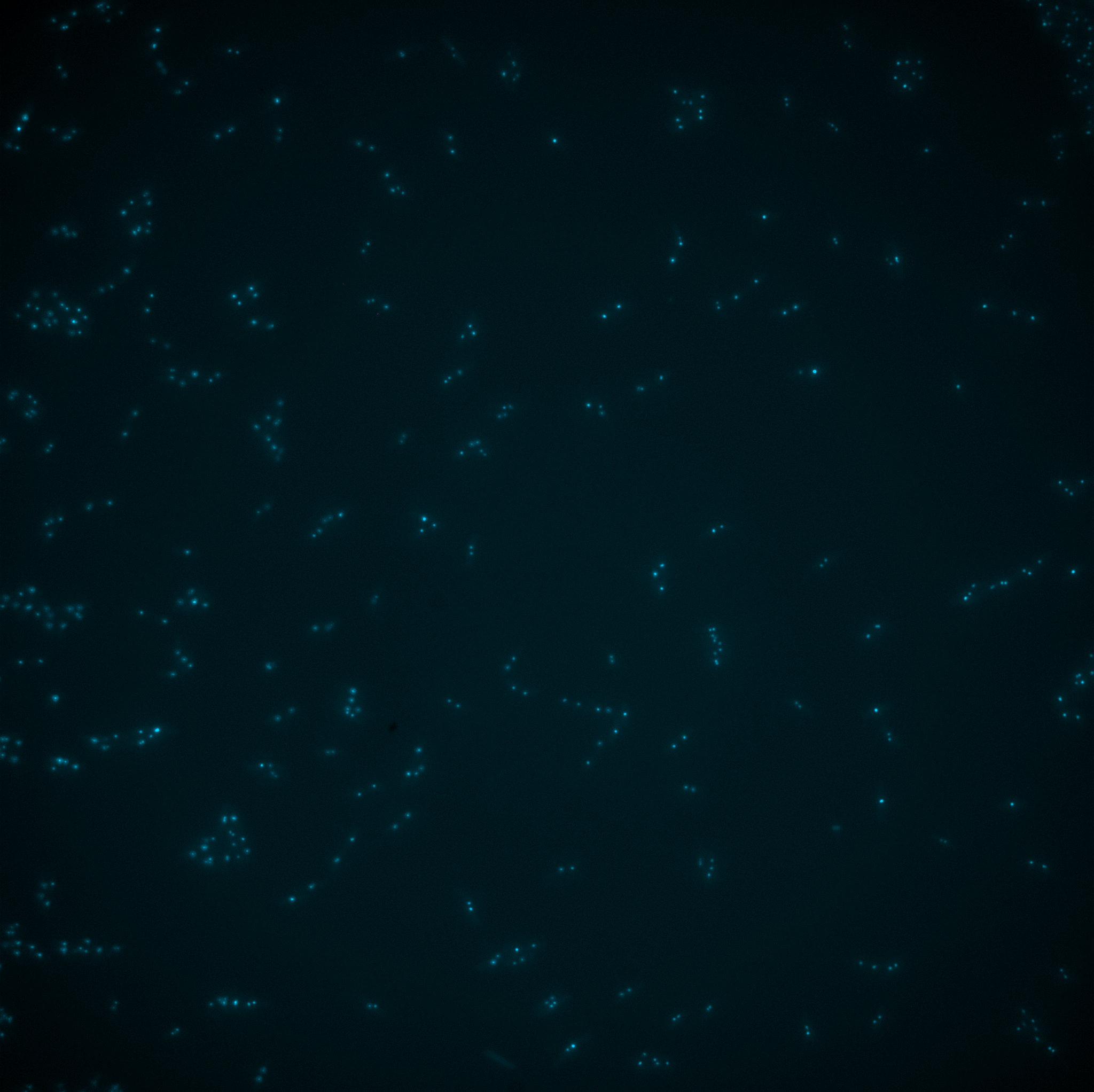

Supplement: Supplementary file 14 — Source data Fig. 2 [file 44321_2025_219_MOESM14_ESM.zip › Figure 2/2A/RCe853 no saccharin TL1018_RGB_eCFP.tif]

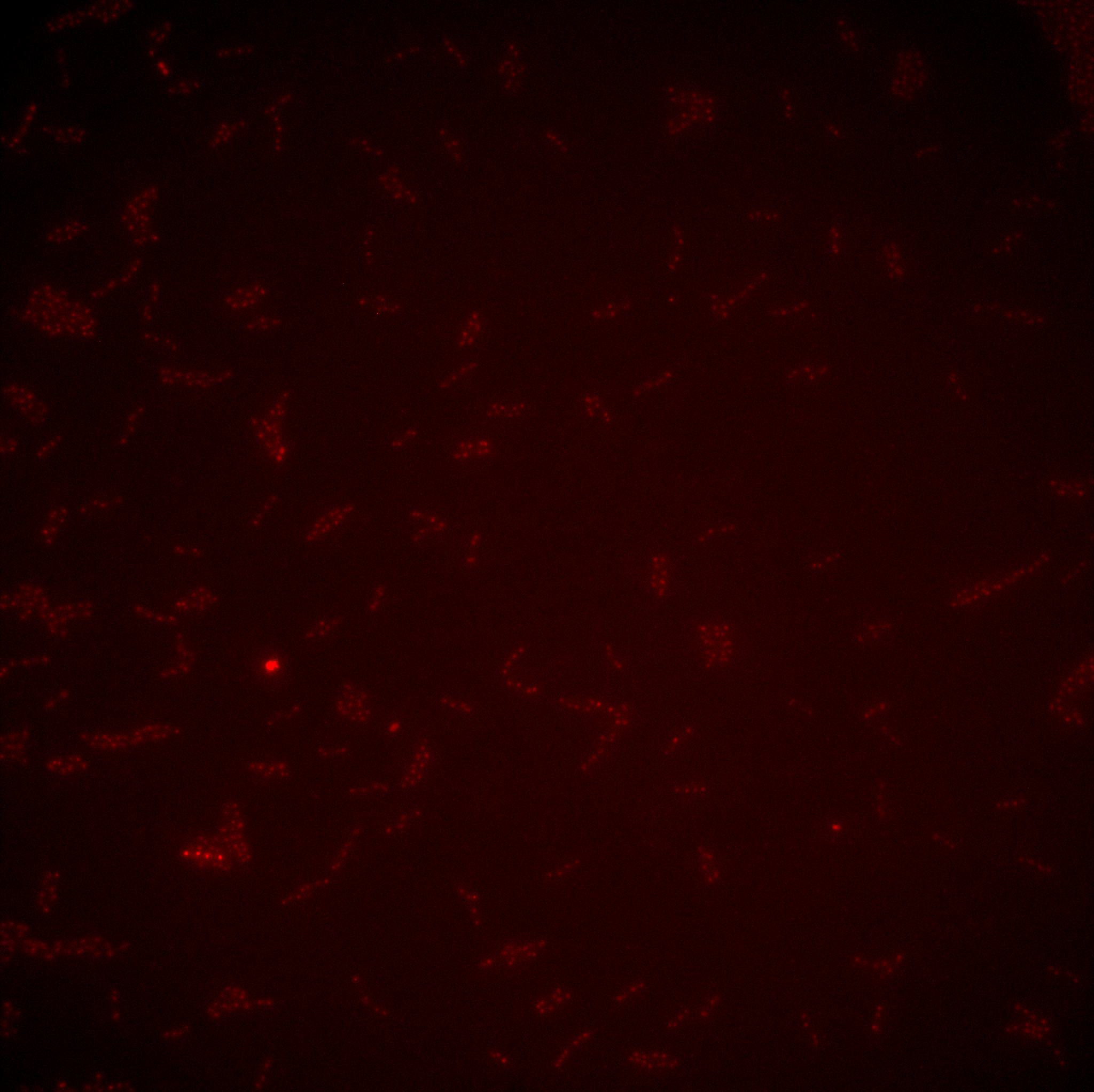

Supplement: Supplementary file 14 — Source data Fig. 2 [file 44321_2025_219_MOESM14_ESM.zip › Figure 2/2A/RCe853 no saccharin TL1018_RGB_mCherry.tif]

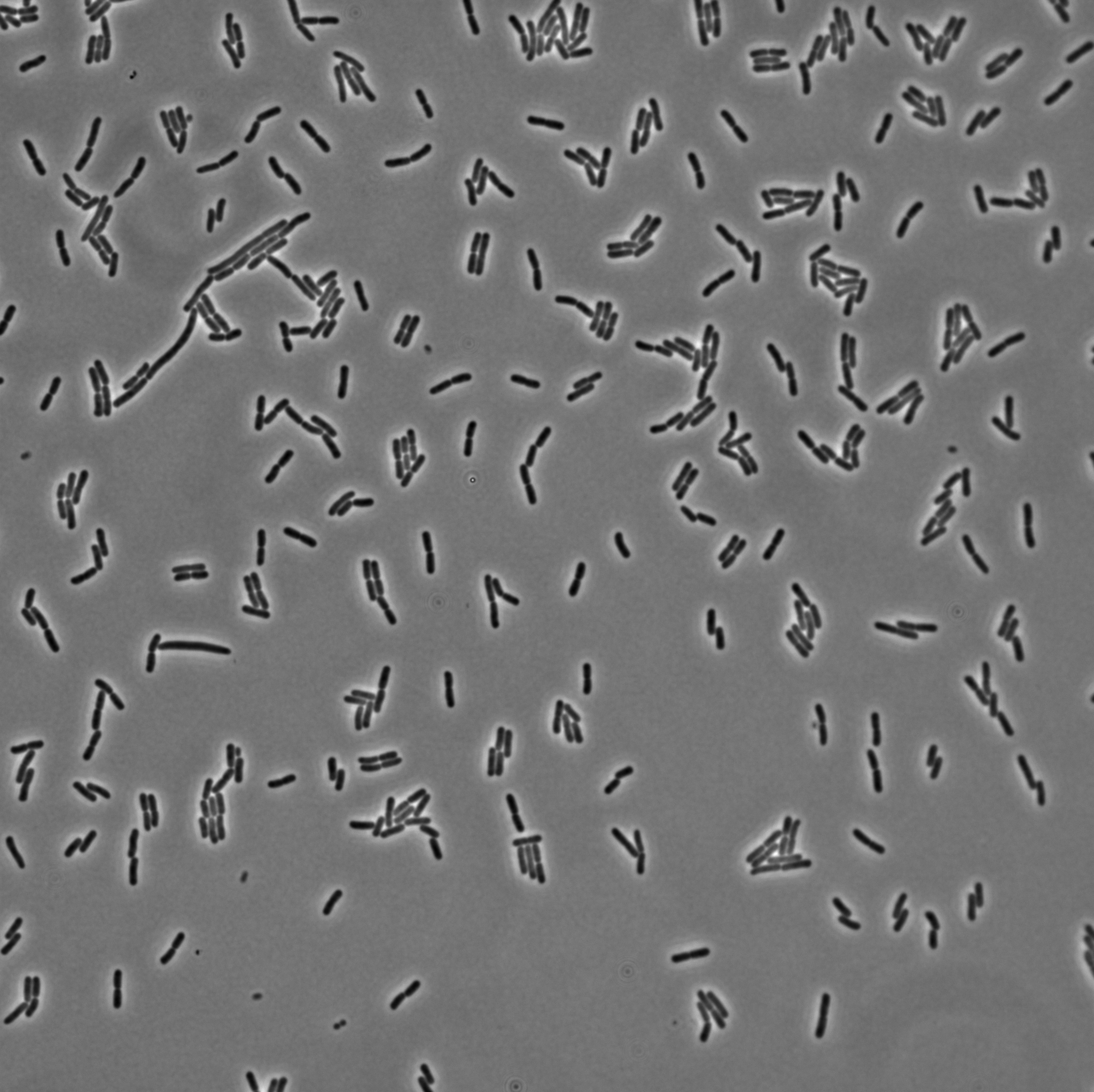

Supplement: Supplementary file 14 — Source data Fig. 2 [file 44321_2025_219_MOESM14_ESM.zip › Figure 2/2A/RCe853 no saccharin TL1019_RGB_Brightfield.tif]
