# Supplementary figures and images for: Saccharin disrupts bacterial cell envelope stability and interferes with DNA replication dynamics (part 2 of 2)
Source: EMBO Mol Med. 2025 Apr 1;17(5):993–1017. doi: 10.1038/s44321-025-00219-1 (PMC12081710; doi:10.1038/s44321-025-00219-1)

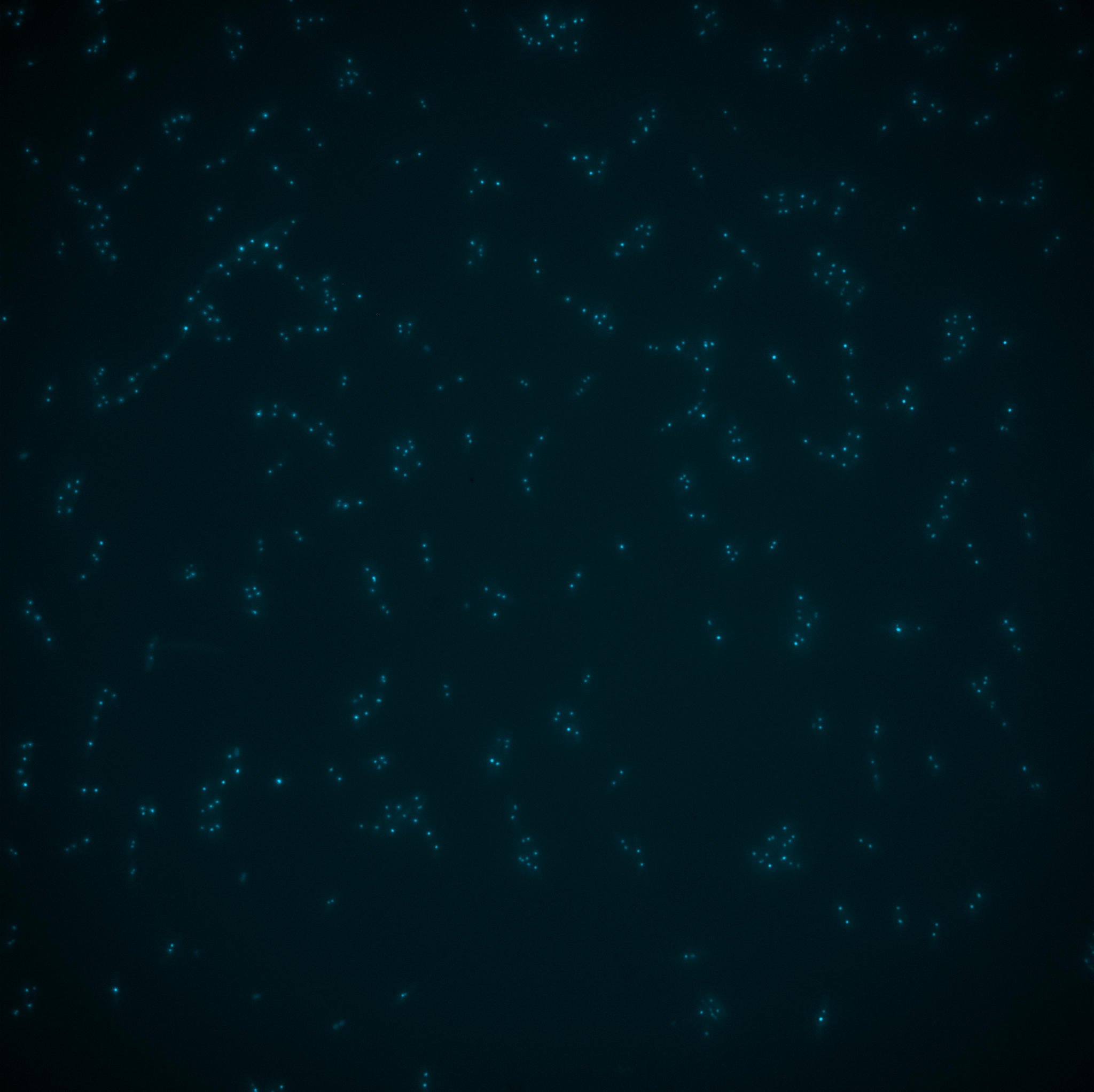

Supplement: Supplementary file 14 — Source data Fig. 2 [file 44321_2025_219_MOESM14_ESM.zip › Figure 2/2A/RCe853 no saccharin TL1019_RGB_eCFP.tif]

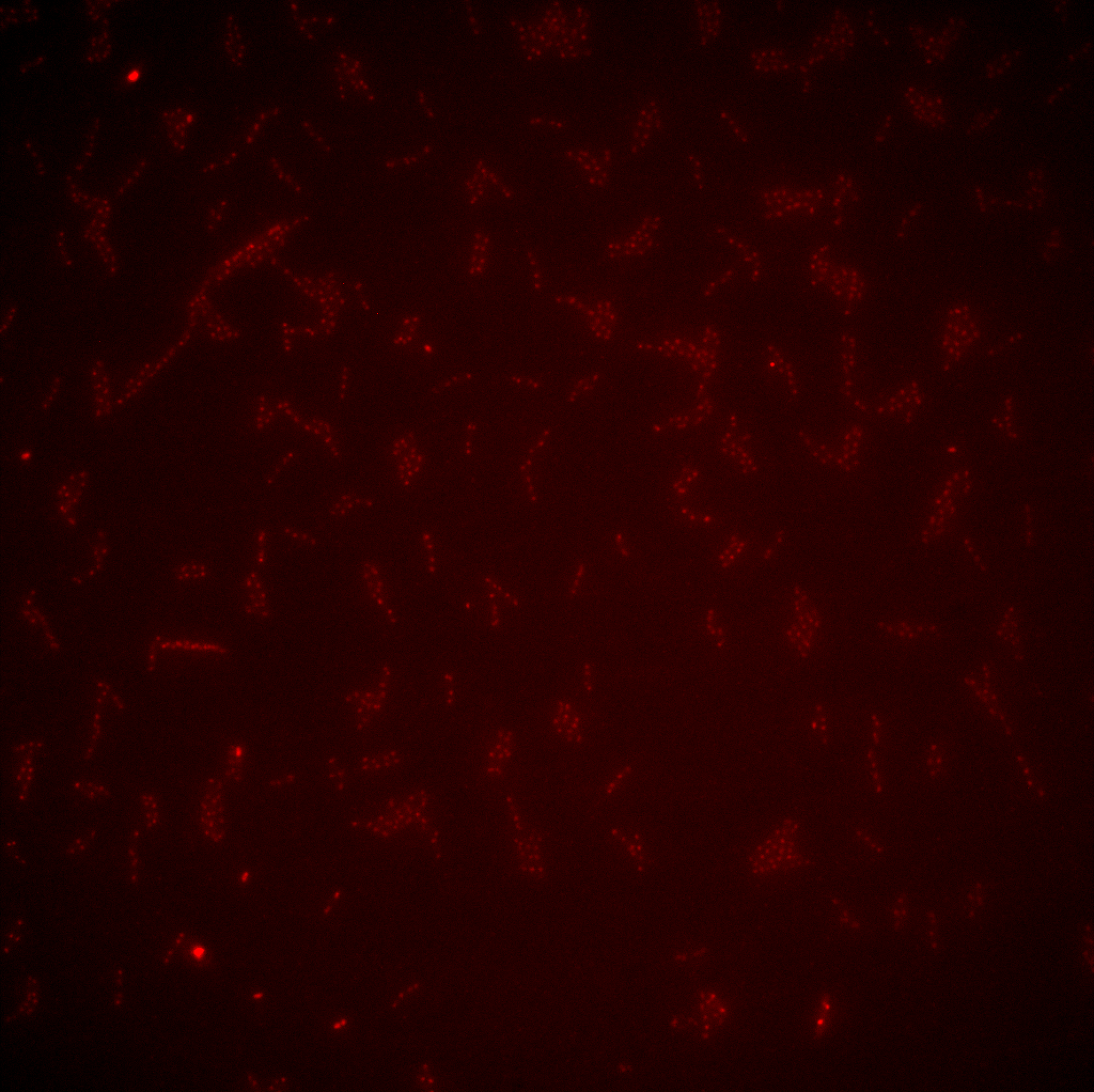

Supplement: Supplementary file 14 — Source data Fig. 2 [file 44321_2025_219_MOESM14_ESM.zip › Figure 2/2A/RCe853 no saccharin TL1019_RGB_mCherry.tif]

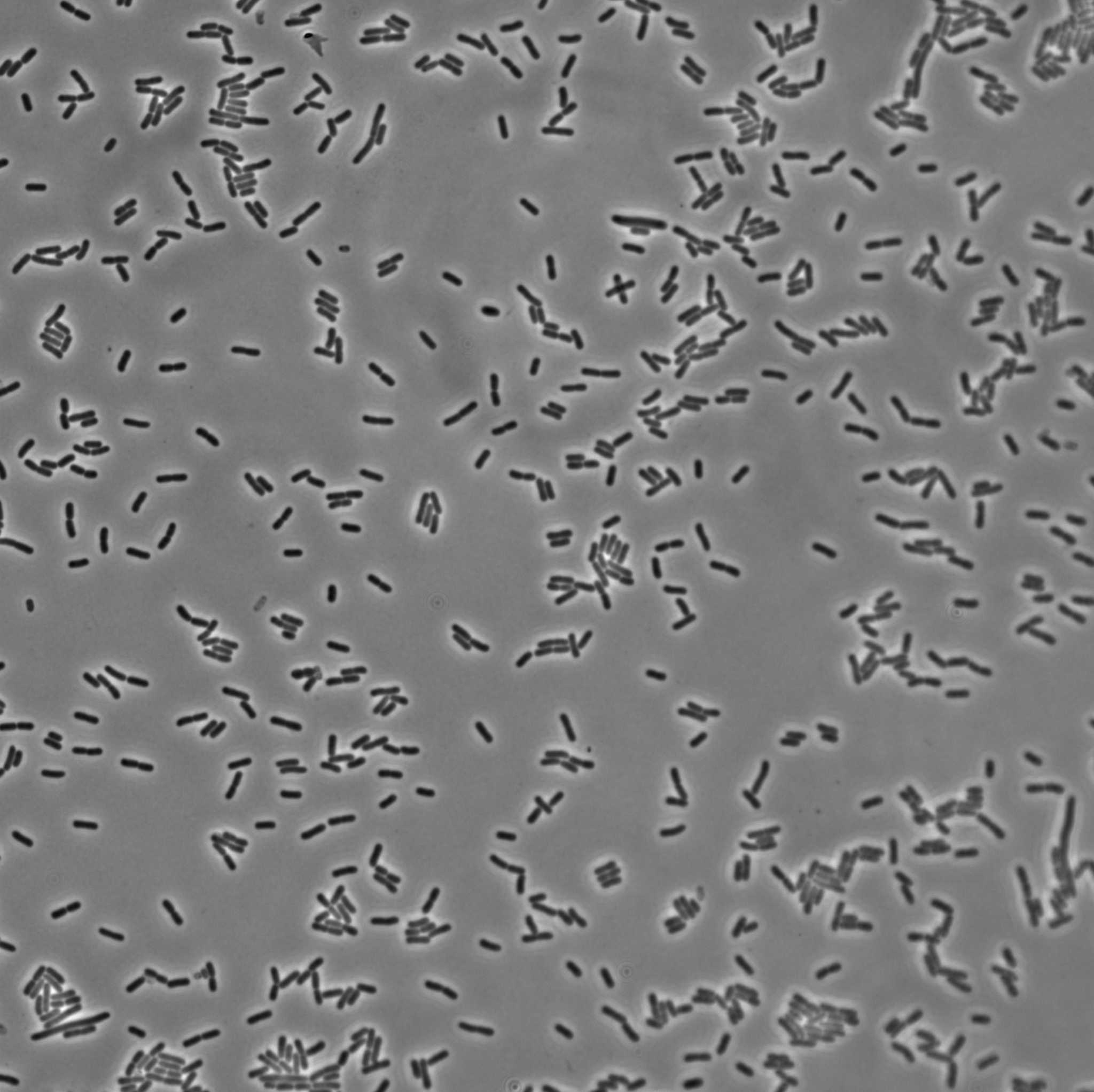

Supplement: Supplementary file 14 — Source data Fig. 2 [file 44321_2025_219_MOESM14_ESM.zip › Figure 2/2C/JD 1473 053_RGB_Brightfield.tif]

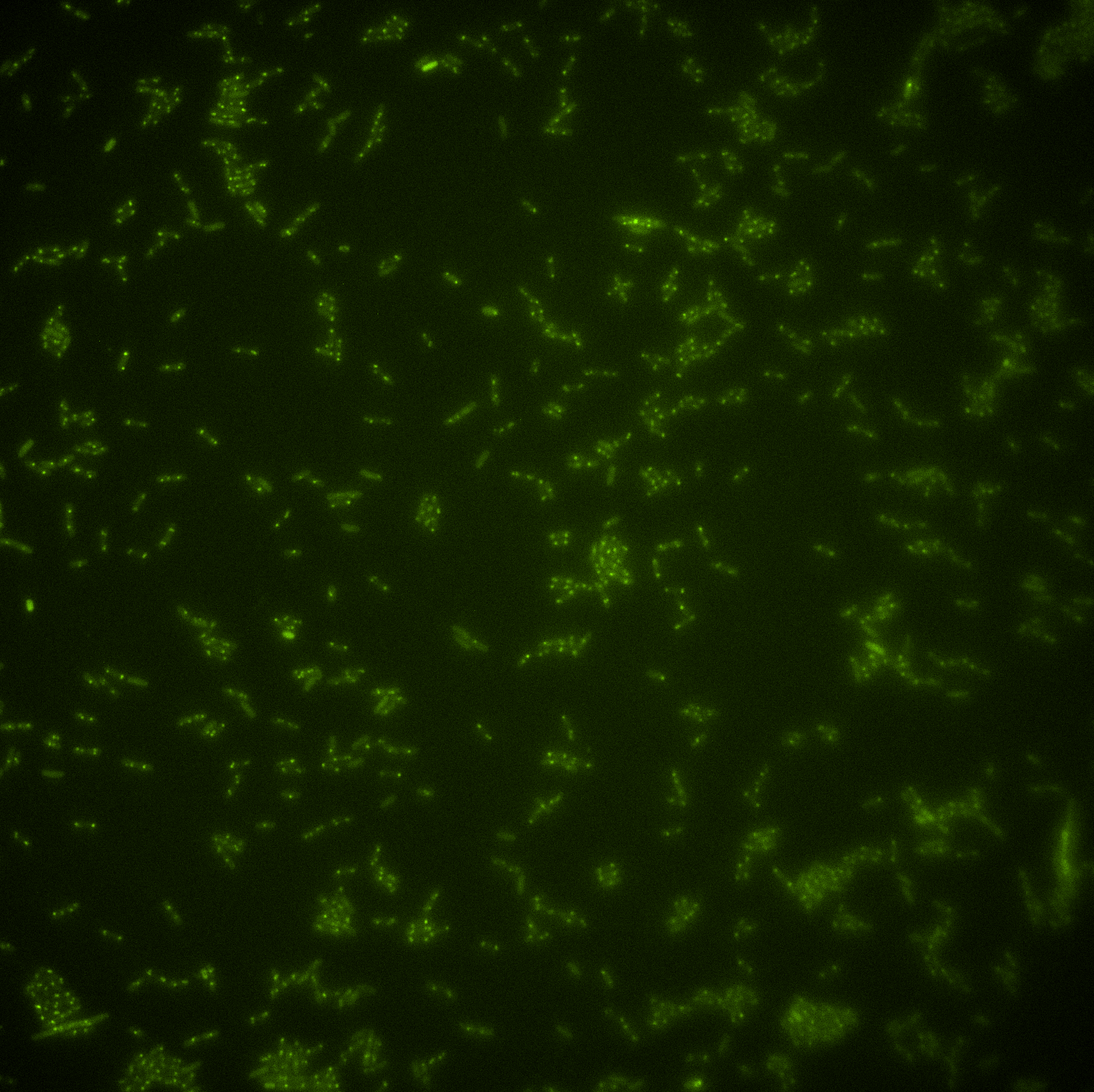

Supplement: Supplementary file 14 — Source data Fig. 2 [file 44321_2025_219_MOESM14_ESM.zip › Figure 2/2C/JD 1473 053_RGB_eYFP.tif]

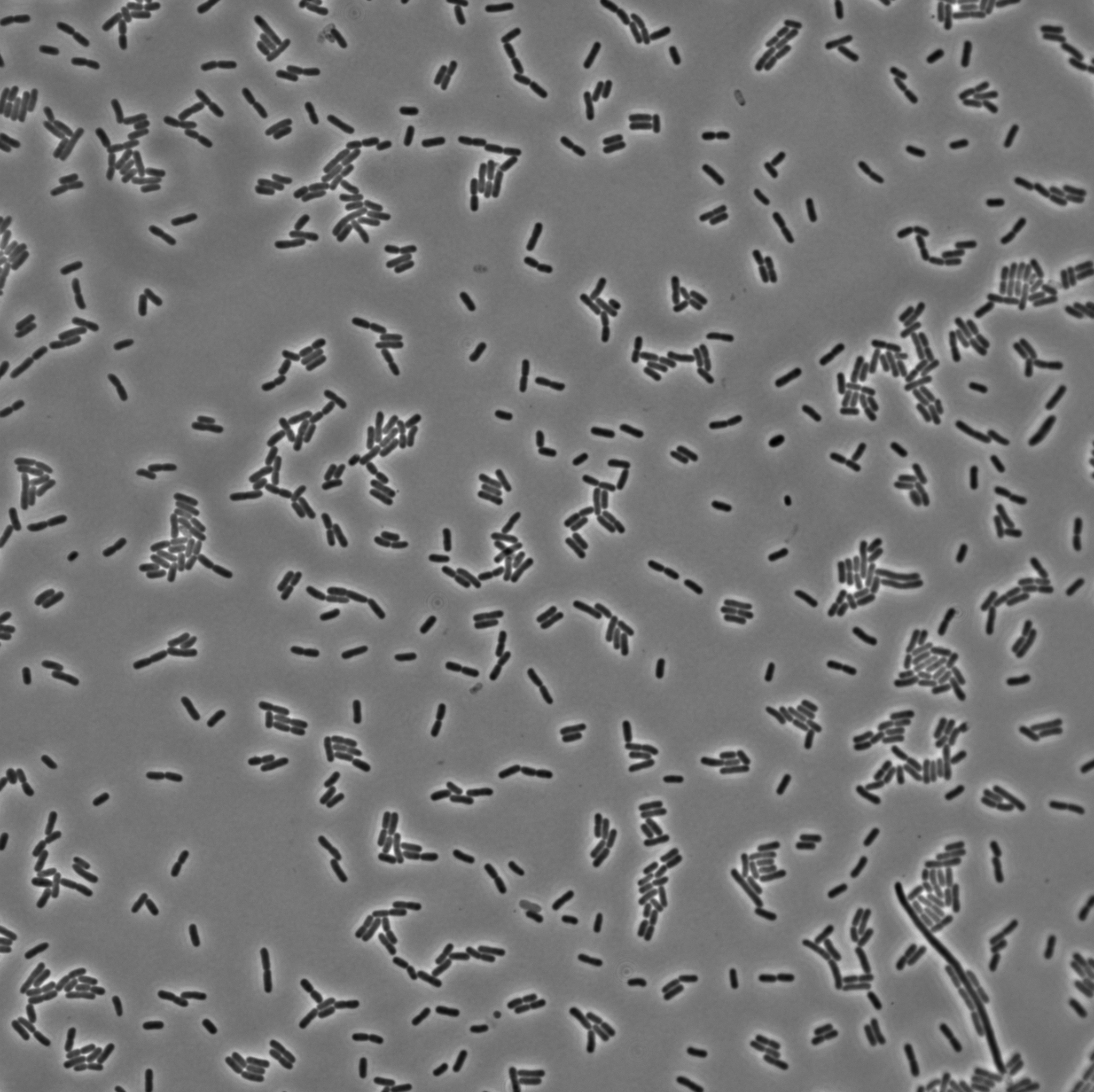

Supplement: Supplementary file 14 — Source data Fig. 2 [file 44321_2025_219_MOESM14_ESM.zip › Figure 2/2C/JD 1473 054_RGB_Brightfield.tif]

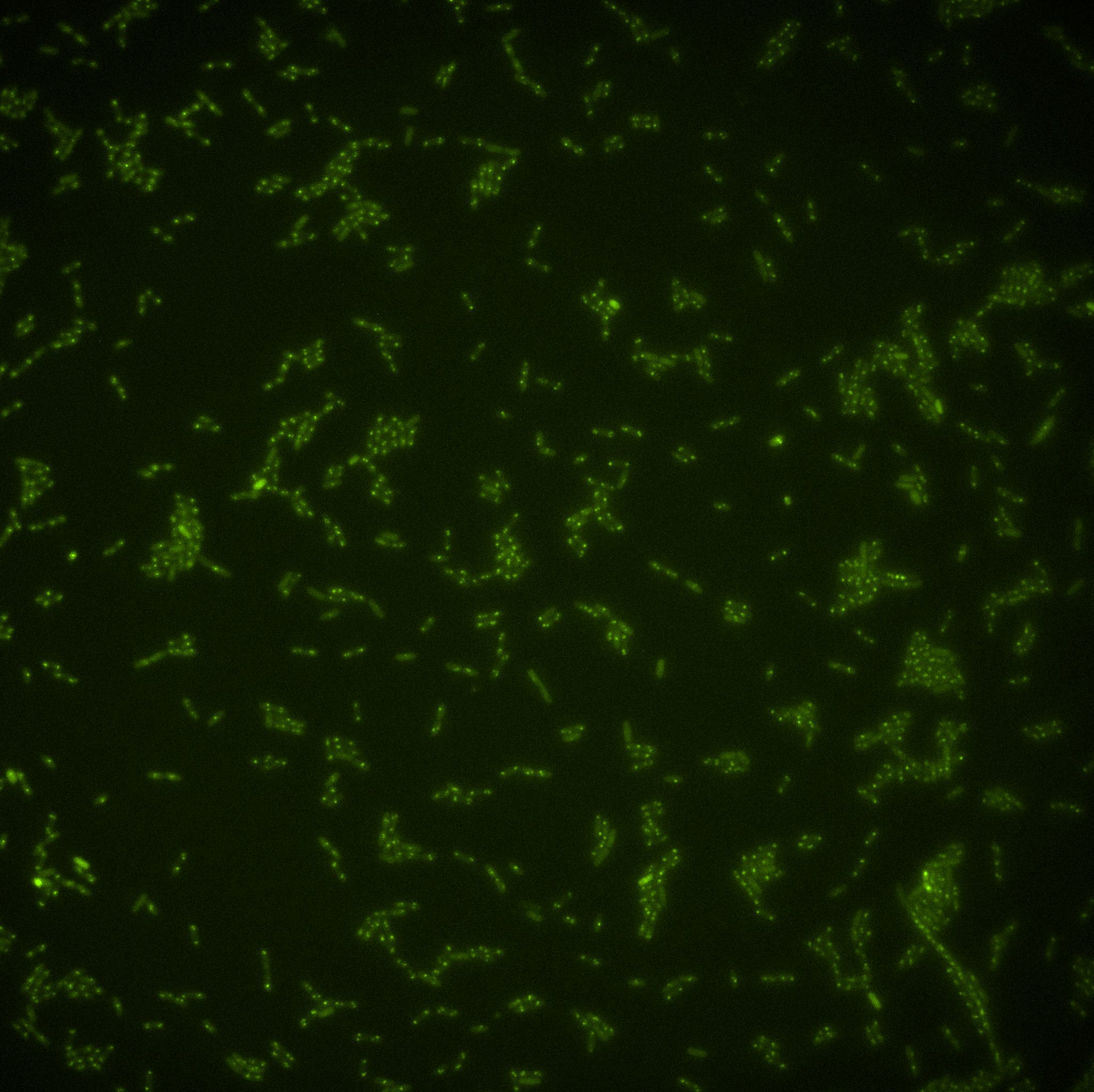

Supplement: Supplementary file 14 — Source data Fig. 2 [file 44321_2025_219_MOESM14_ESM.zip › Figure 2/2C/JD 1473 054_RGB_eYFP.tif]

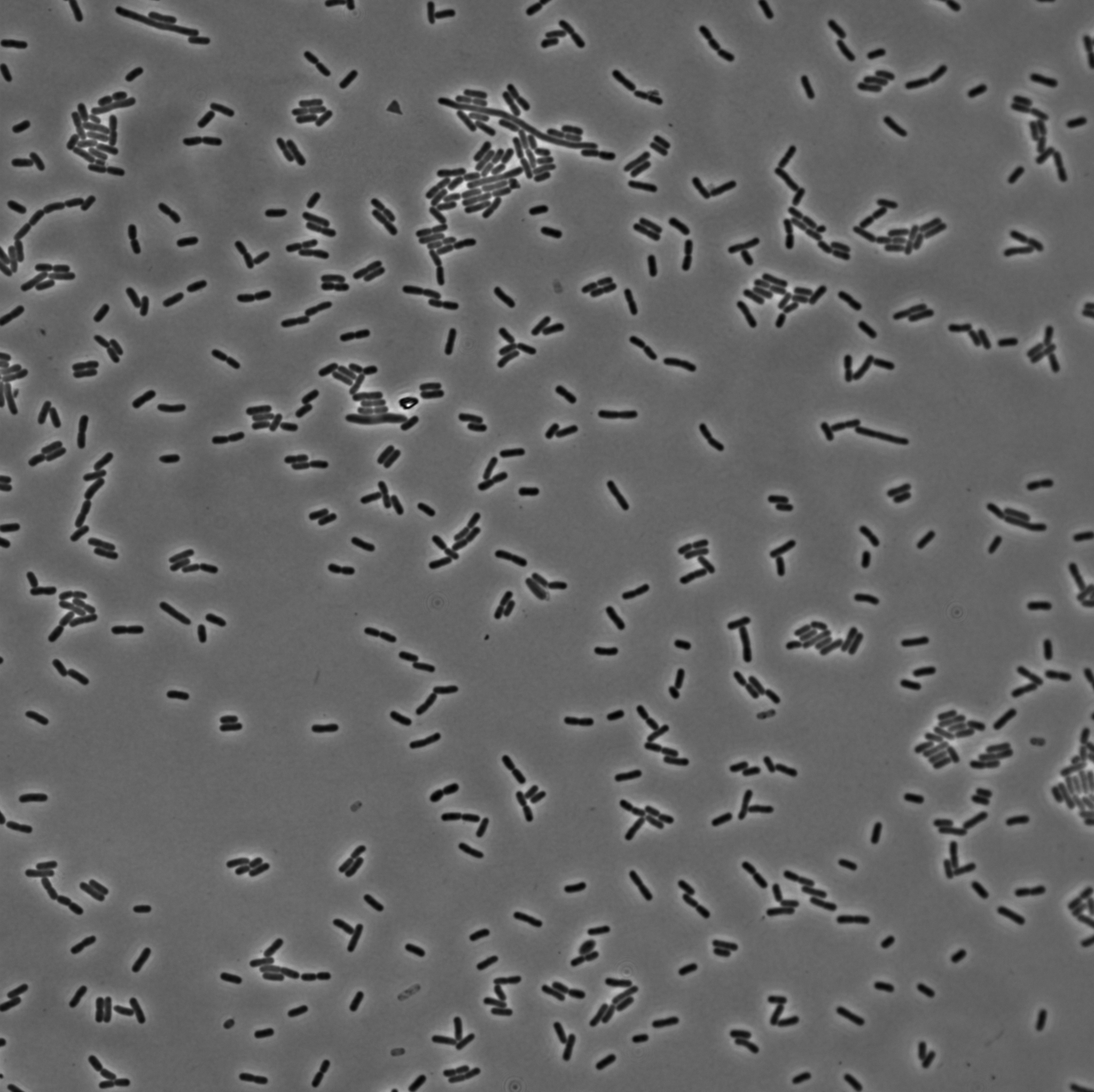

Supplement: Supplementary file 14 — Source data Fig. 2 [file 44321_2025_219_MOESM14_ESM.zip › Figure 2/2C/JD 1473 055_RGB_Brightfield.tif]

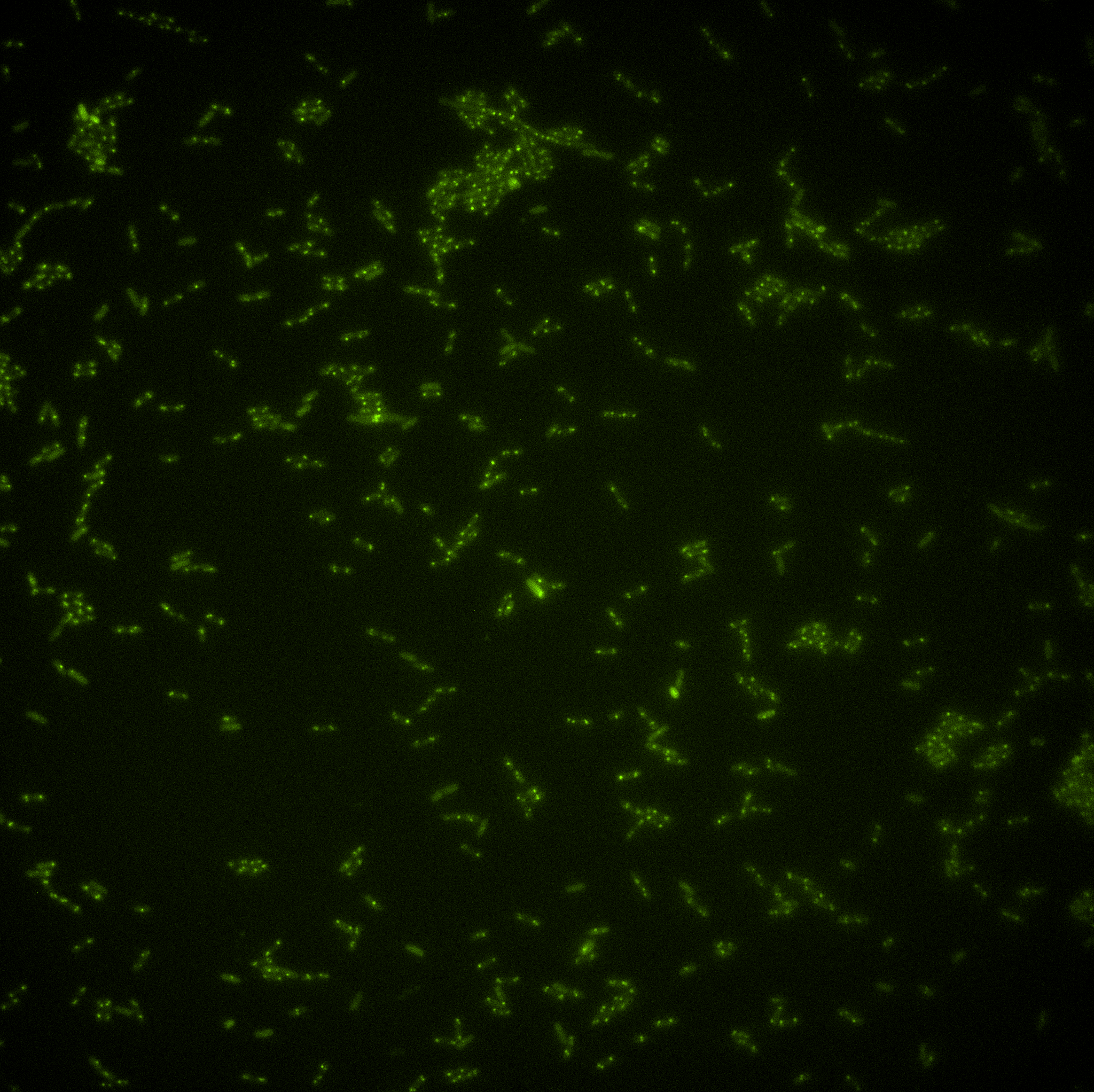

Supplement: Supplementary file 14 — Source data Fig. 2 [file 44321_2025_219_MOESM14_ESM.zip › Figure 2/2C/JD 1473 055_RGB_eYFP.tif]

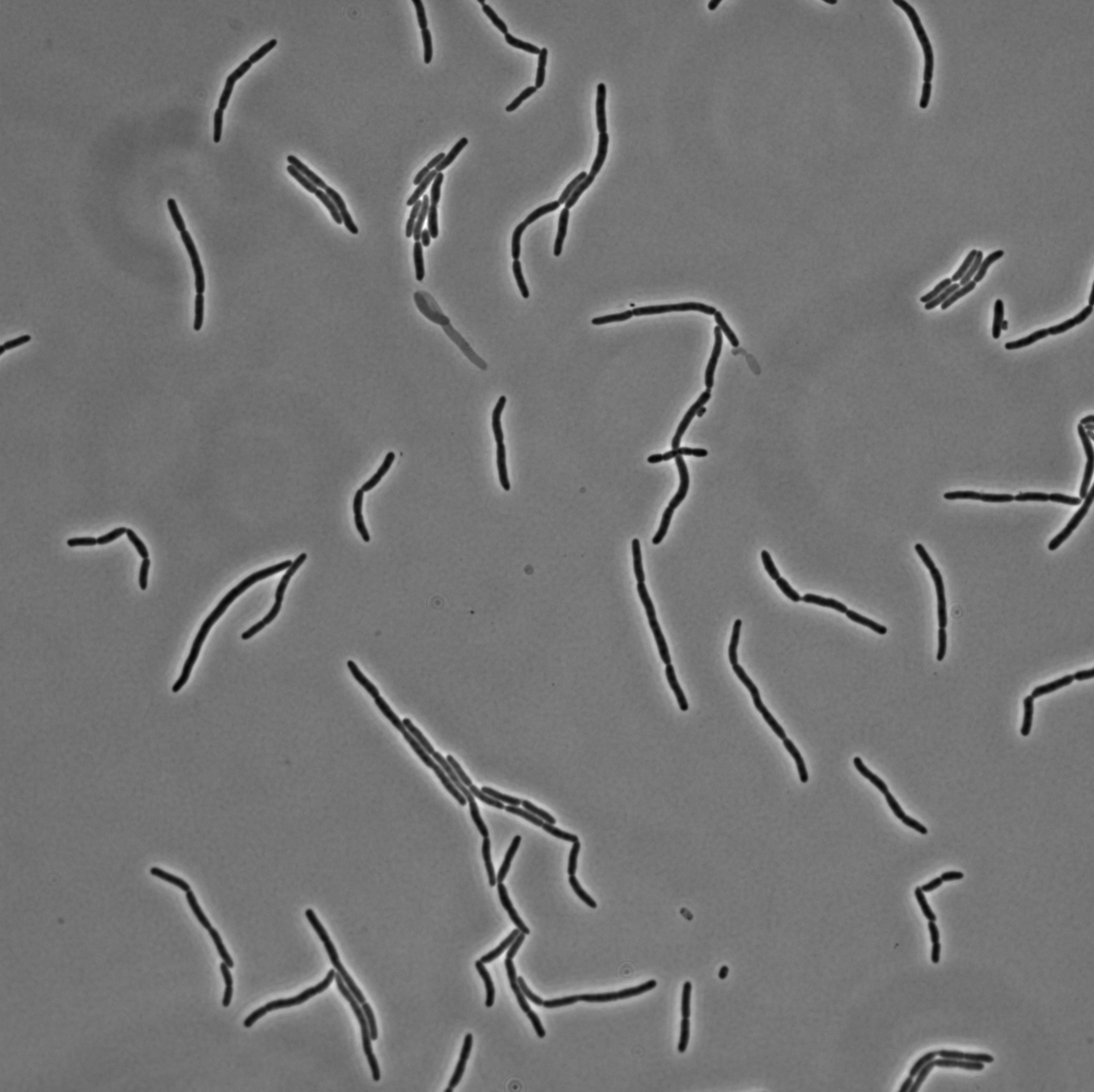

Supplement: Supplementary file 14 — Source data Fig. 2 [file 44321_2025_219_MOESM14_ESM.zip › Figure 2/2C/JD 1473 1.4%1hr 059_RGB_Brightfield.tif]

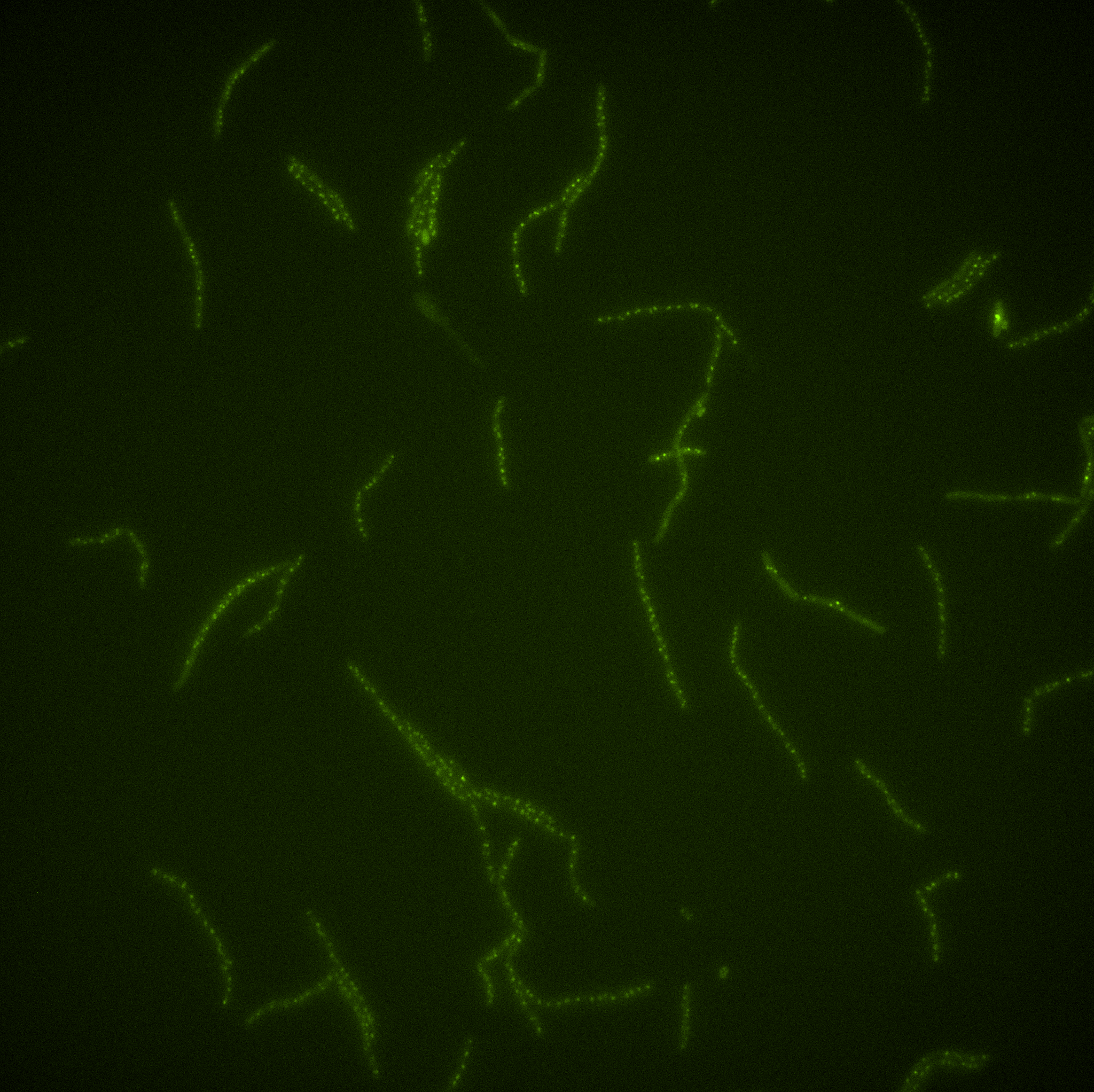

Supplement: Supplementary file 14 — Source data Fig. 2 [file 44321_2025_219_MOESM14_ESM.zip › Figure 2/2C/JD 1473 1.4%1hr 059_RGB_eYFP.tif]

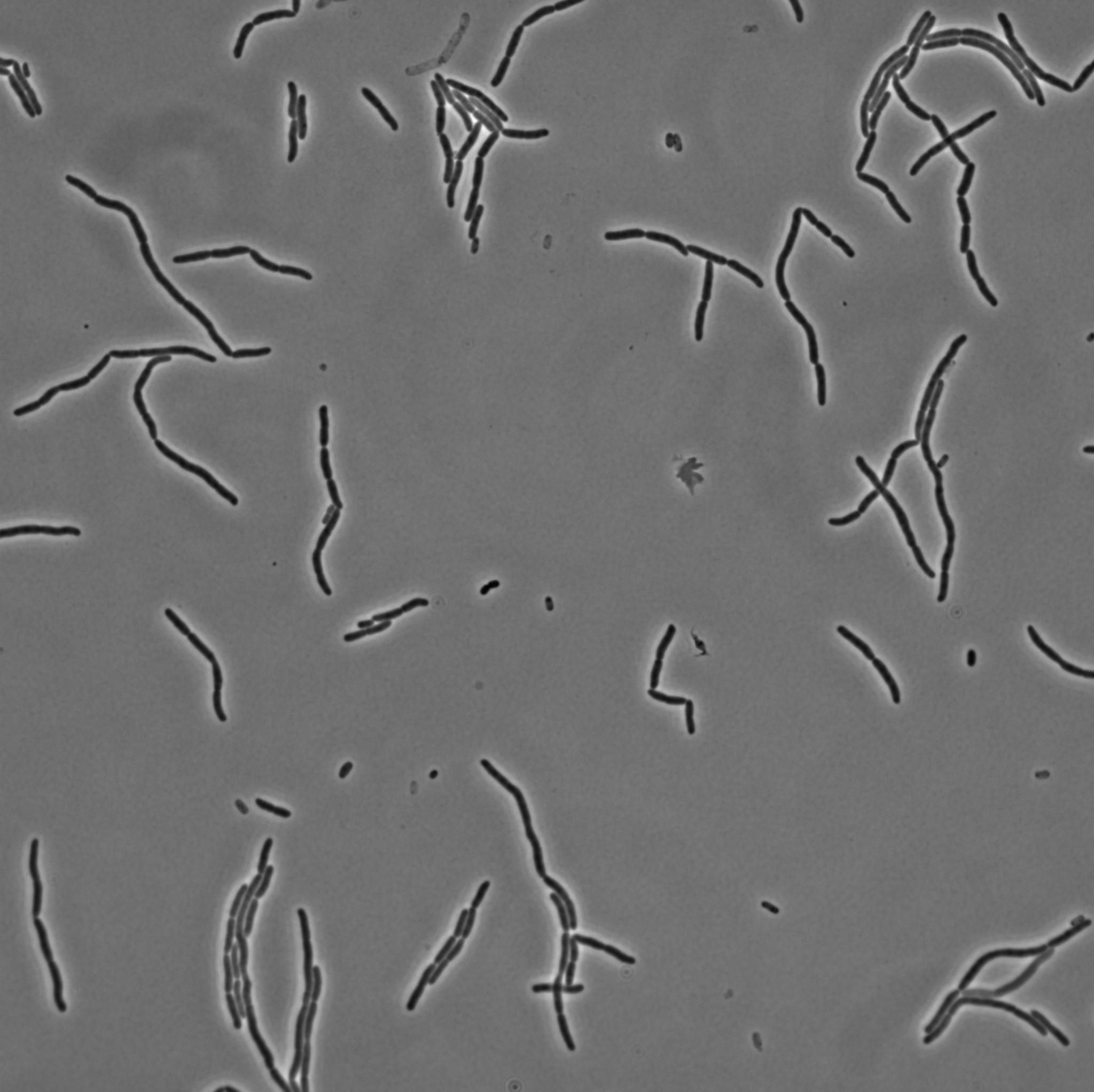

Supplement: Supplementary file 14 — Source data Fig. 2 [file 44321_2025_219_MOESM14_ESM.zip › Figure 2/2C/JD 1473 1.4%1hr 060_RGB_Brightfield.tif]

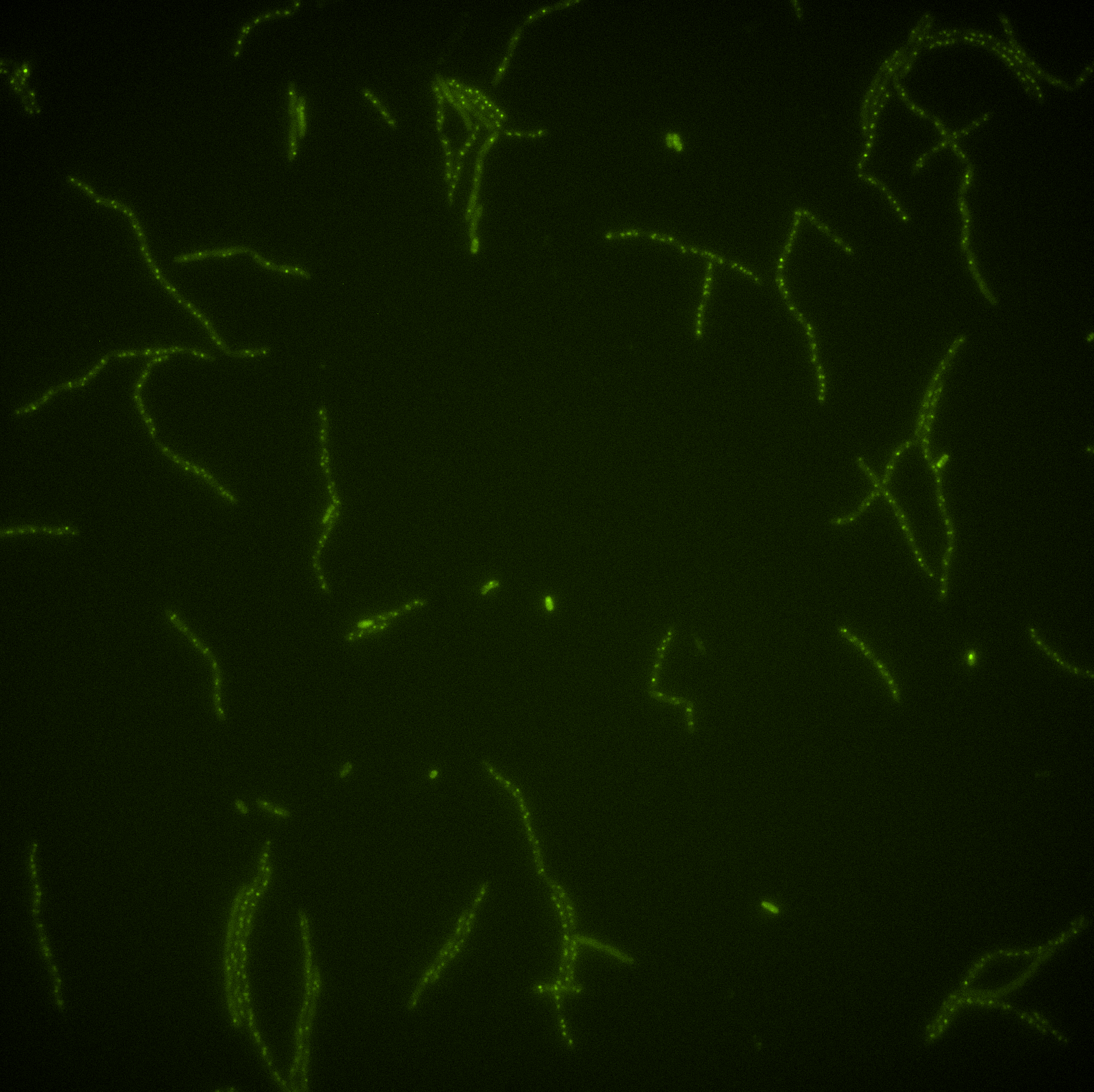

Supplement: Supplementary file 14 — Source data Fig. 2 [file 44321_2025_219_MOESM14_ESM.zip › Figure 2/2C/JD 1473 1.4%1hr 060_RGB_eYFP.tif]

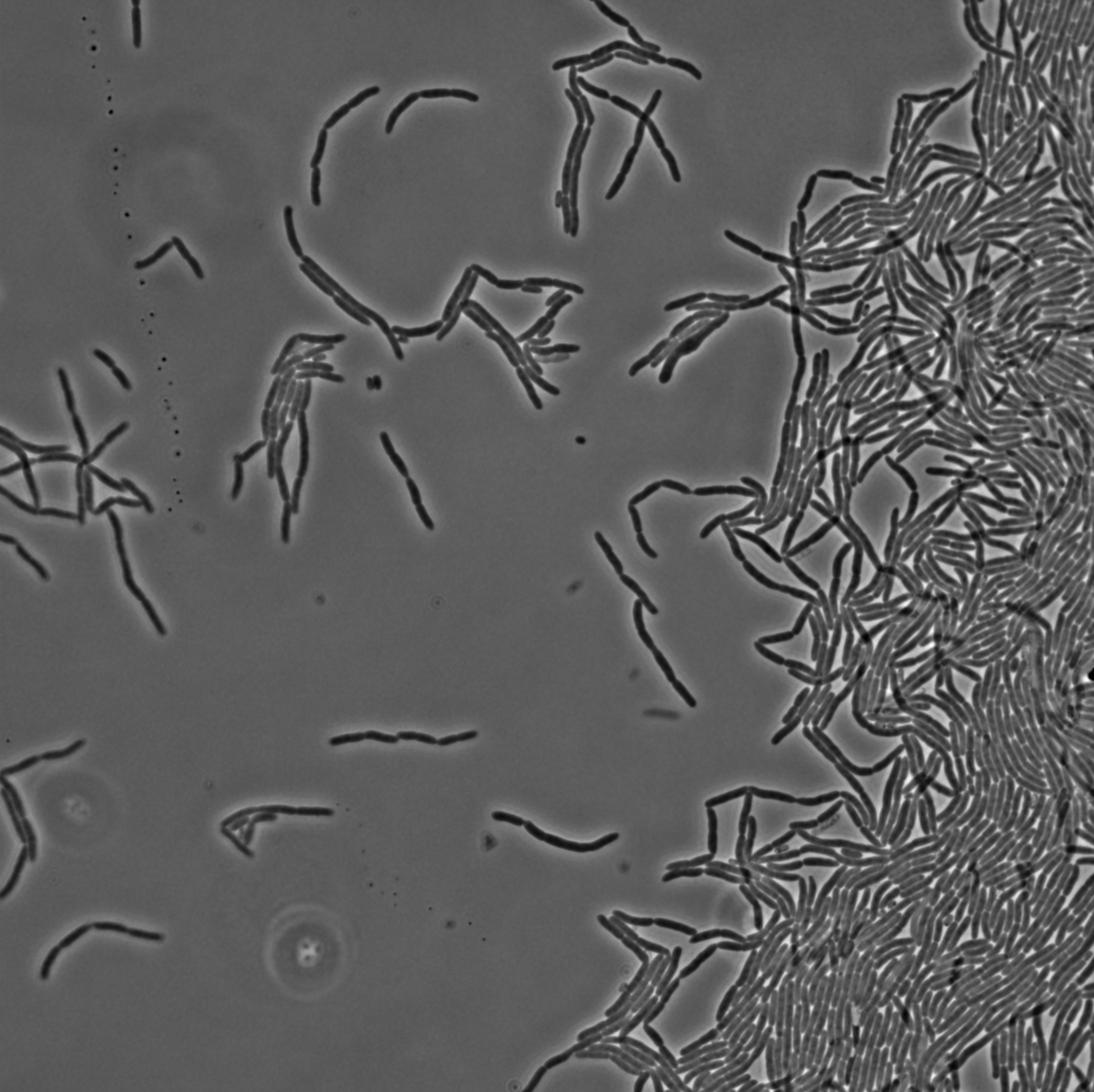

Supplement: Supplementary file 14 — Source data Fig. 2 [file 44321_2025_219_MOESM14_ESM.zip › Figure 2/2C/JD 1473 1.4%1hr 061_RGB_Brightfield.tif]

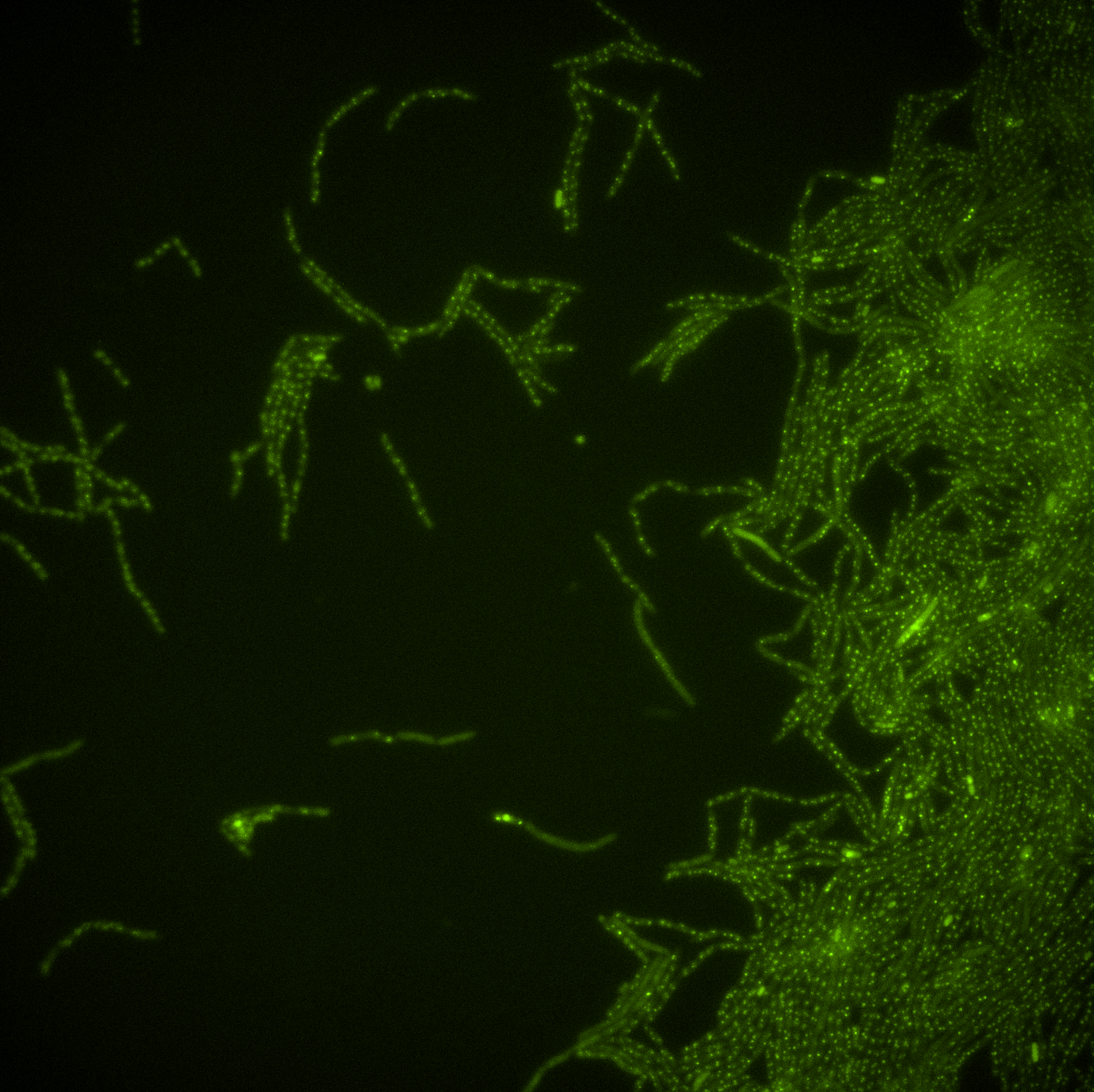

Supplement: Supplementary file 14 — Source data Fig. 2 [file 44321_2025_219_MOESM14_ESM.zip › Figure 2/2C/JD 1473 1.4%1hr 061_RGB_eYFP.tif]

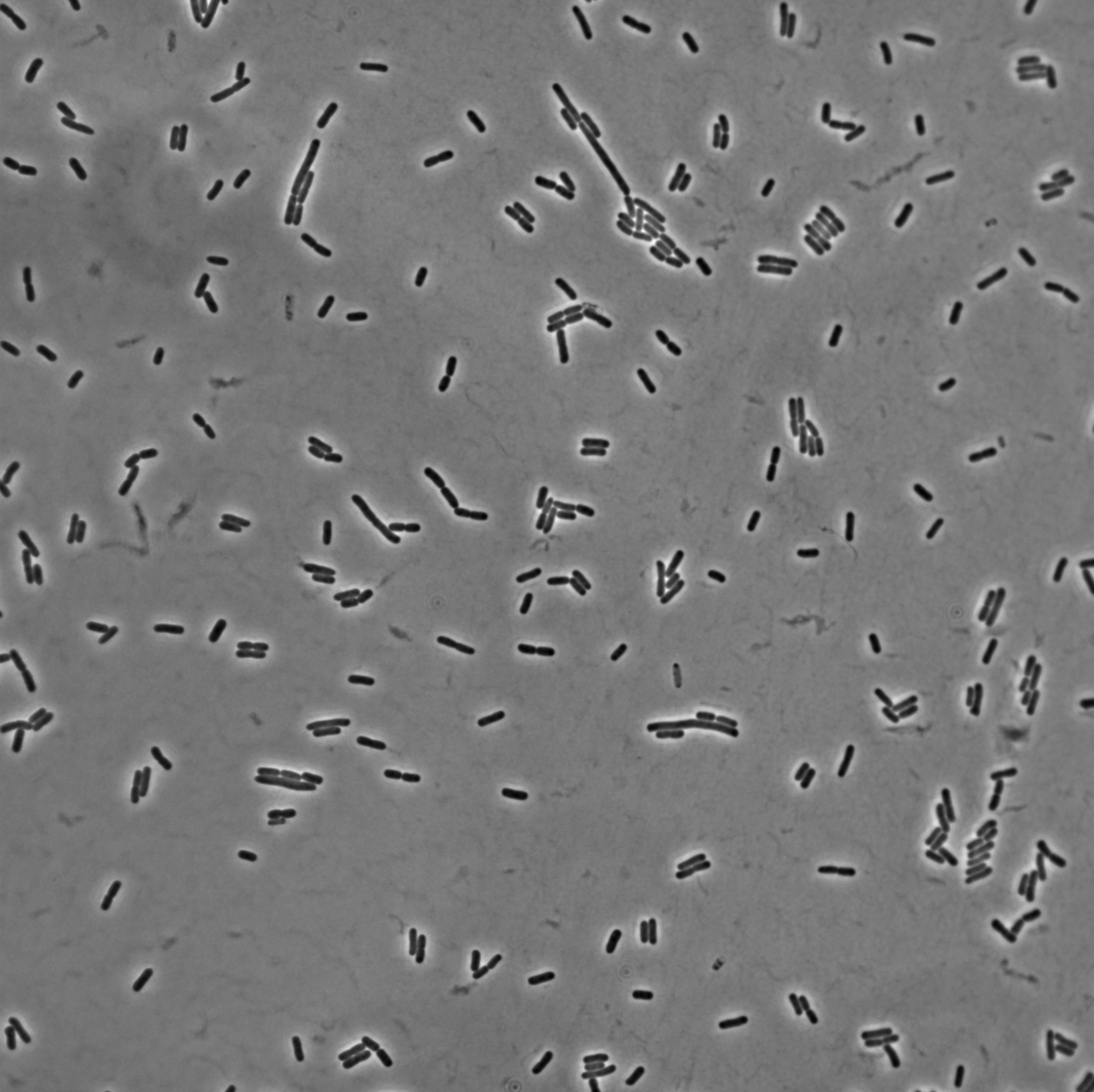

Supplement: Supplementary file 14 — Source data Fig. 2 [file 44321_2025_219_MOESM14_ESM.zip › Figure 2/2E/JD 1708 066_RGB_Brightfield.tif]

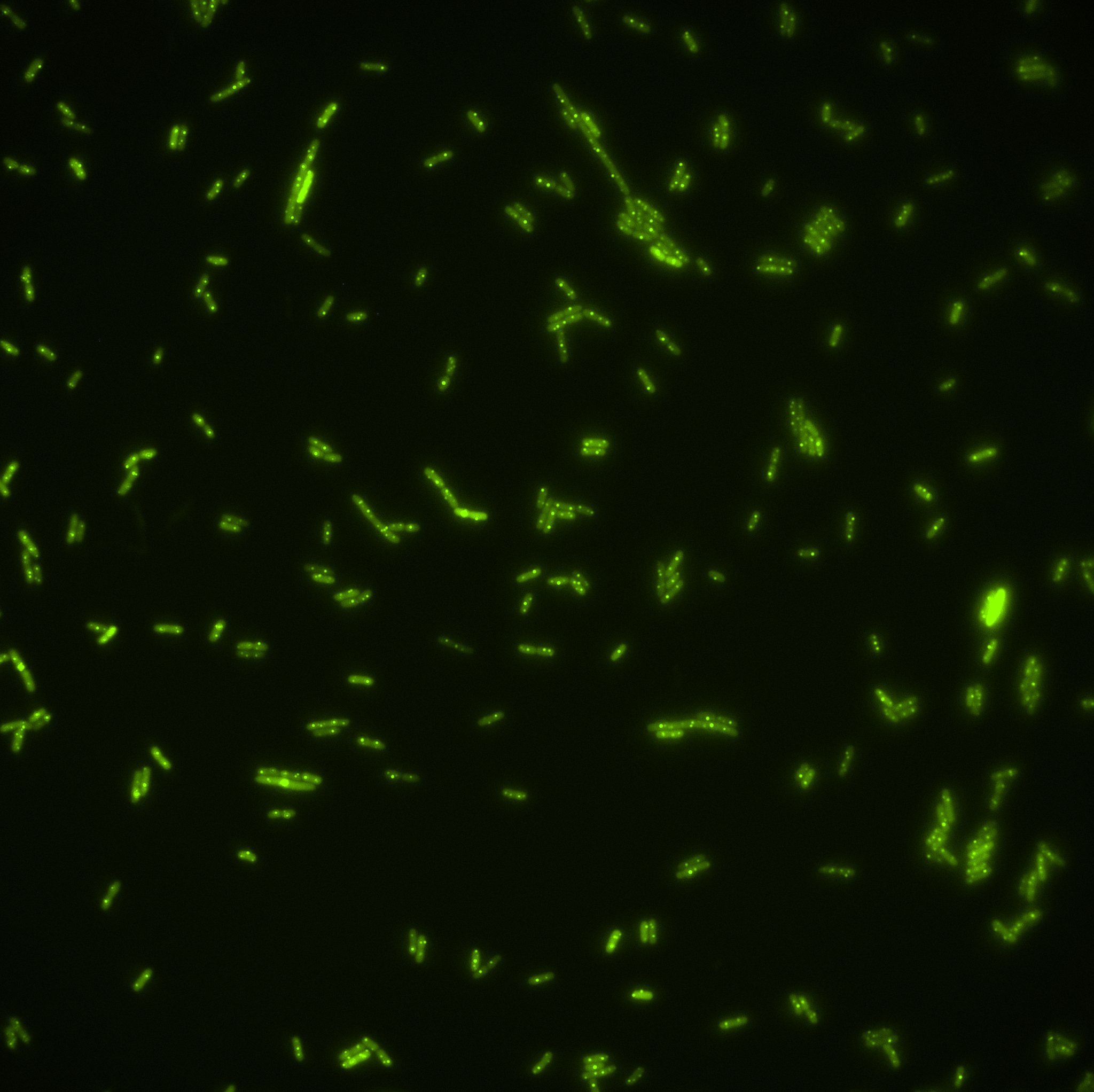

Supplement: Supplementary file 14 — Source data Fig. 2 [file 44321_2025_219_MOESM14_ESM.zip › Figure 2/2E/JD 1708 066_RGB_eYFP.tif]

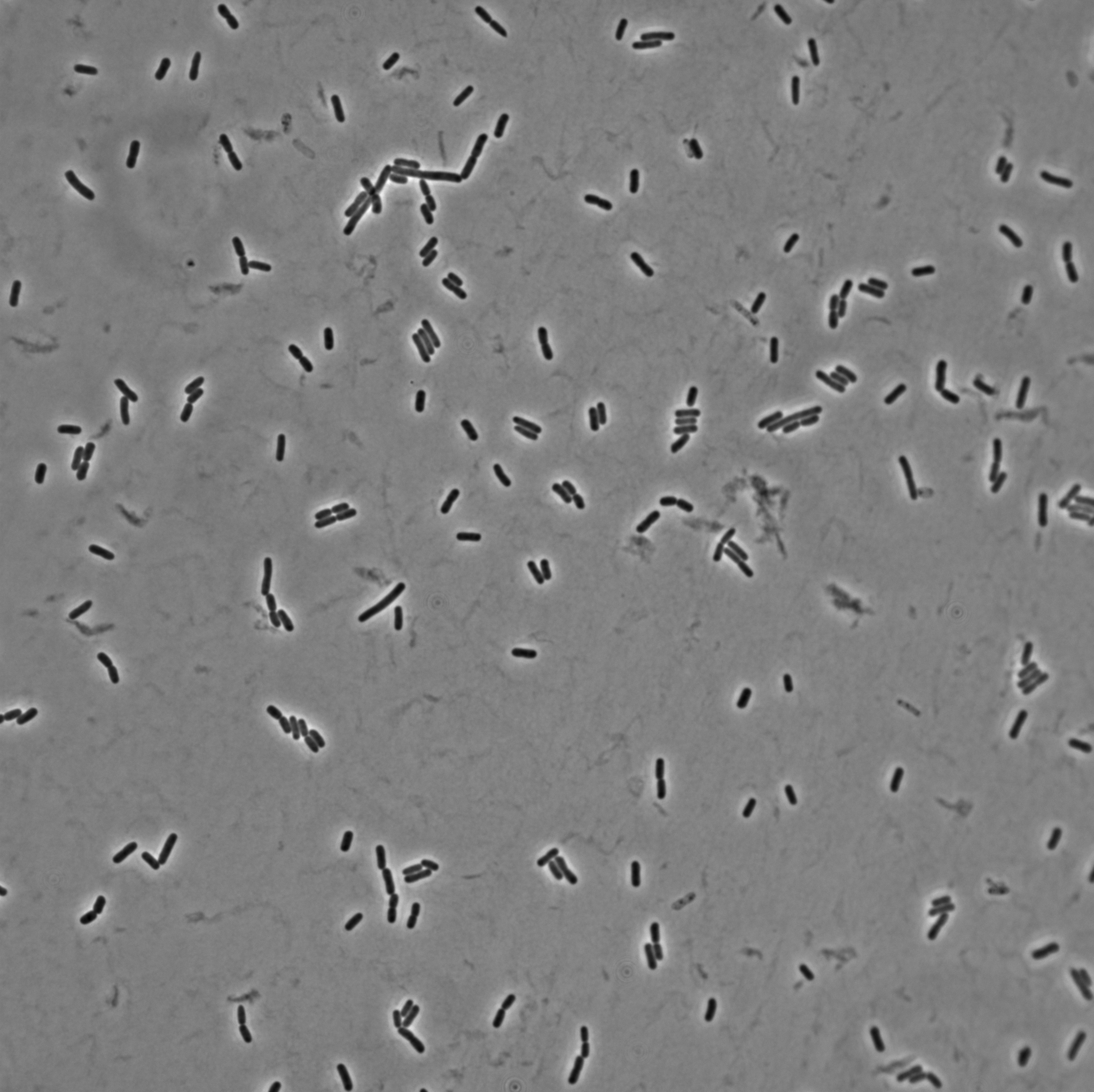

Supplement: Supplementary file 14 — Source data Fig. 2 [file 44321_2025_219_MOESM14_ESM.zip › Figure 2/2E/JD 1708 067_RGB_Brightfield.tif]

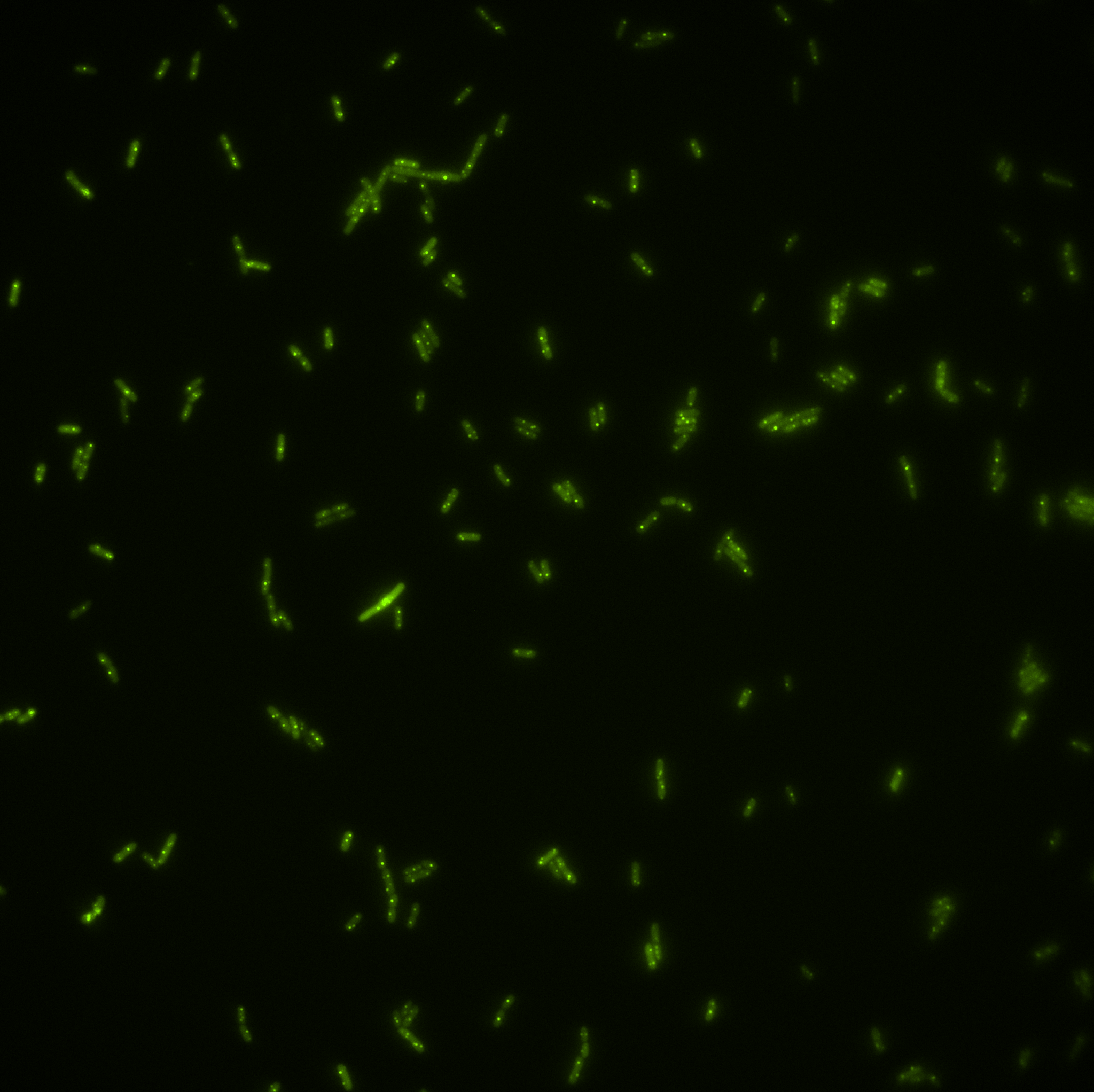

Supplement: Supplementary file 14 — Source data Fig. 2 [file 44321_2025_219_MOESM14_ESM.zip › Figure 2/2E/JD 1708 067_RGB_eYFP.tif]

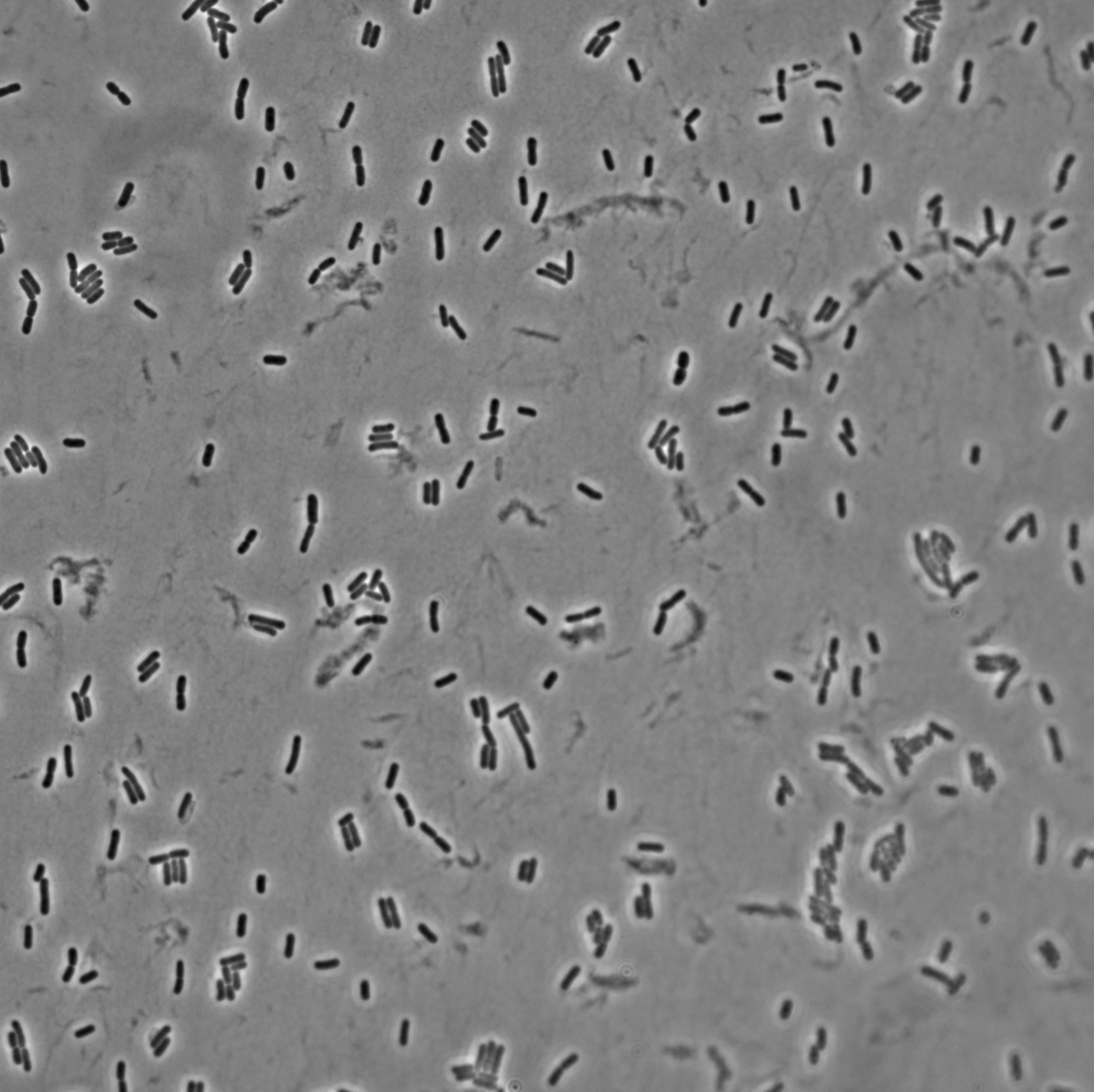

Supplement: Supplementary file 14 — Source data Fig. 2 [file 44321_2025_219_MOESM14_ESM.zip › Figure 2/2E/JD 1708 068_RGB_Brightfield.tif]

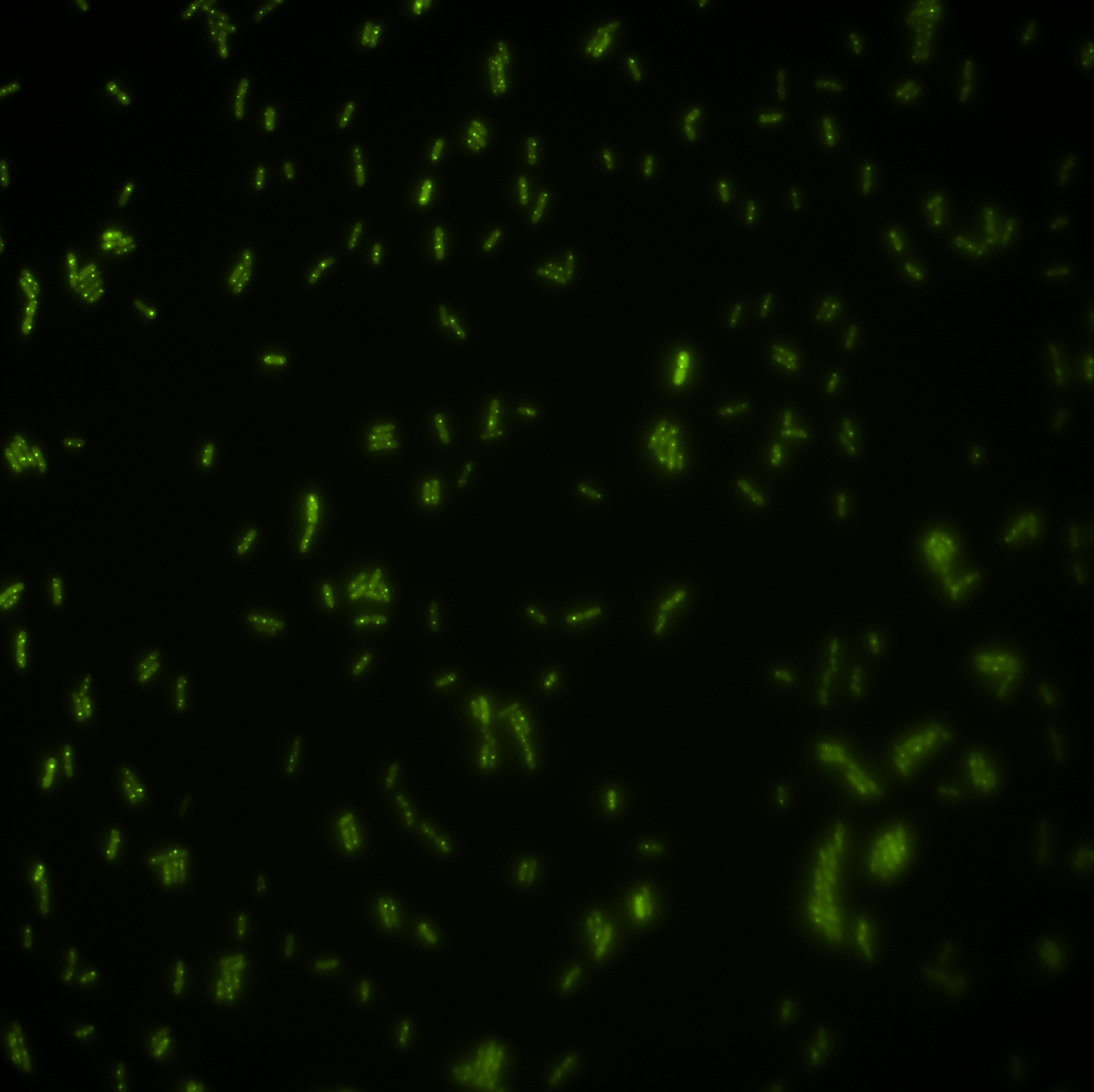

Supplement: Supplementary file 14 — Source data Fig. 2 [file 44321_2025_219_MOESM14_ESM.zip › Figure 2/2E/JD 1708 068_RGB_eYFP.tif]

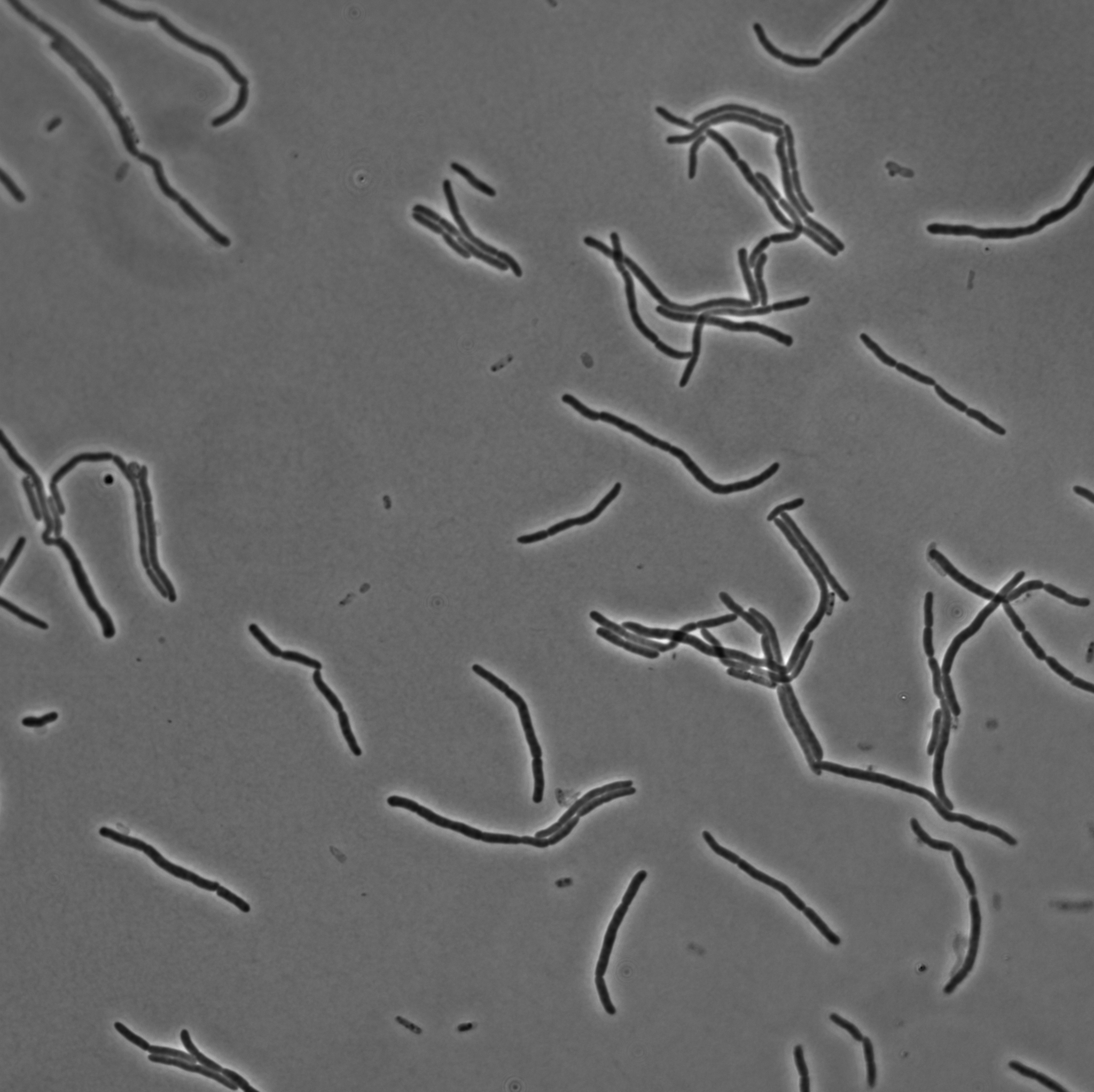

Supplement: Supplementary file 14 — Source data Fig. 2 [file 44321_2025_219_MOESM14_ESM.zip › Figure 2/2E/JD 1708 1.4% 071_RGB_Brightfield.tif]

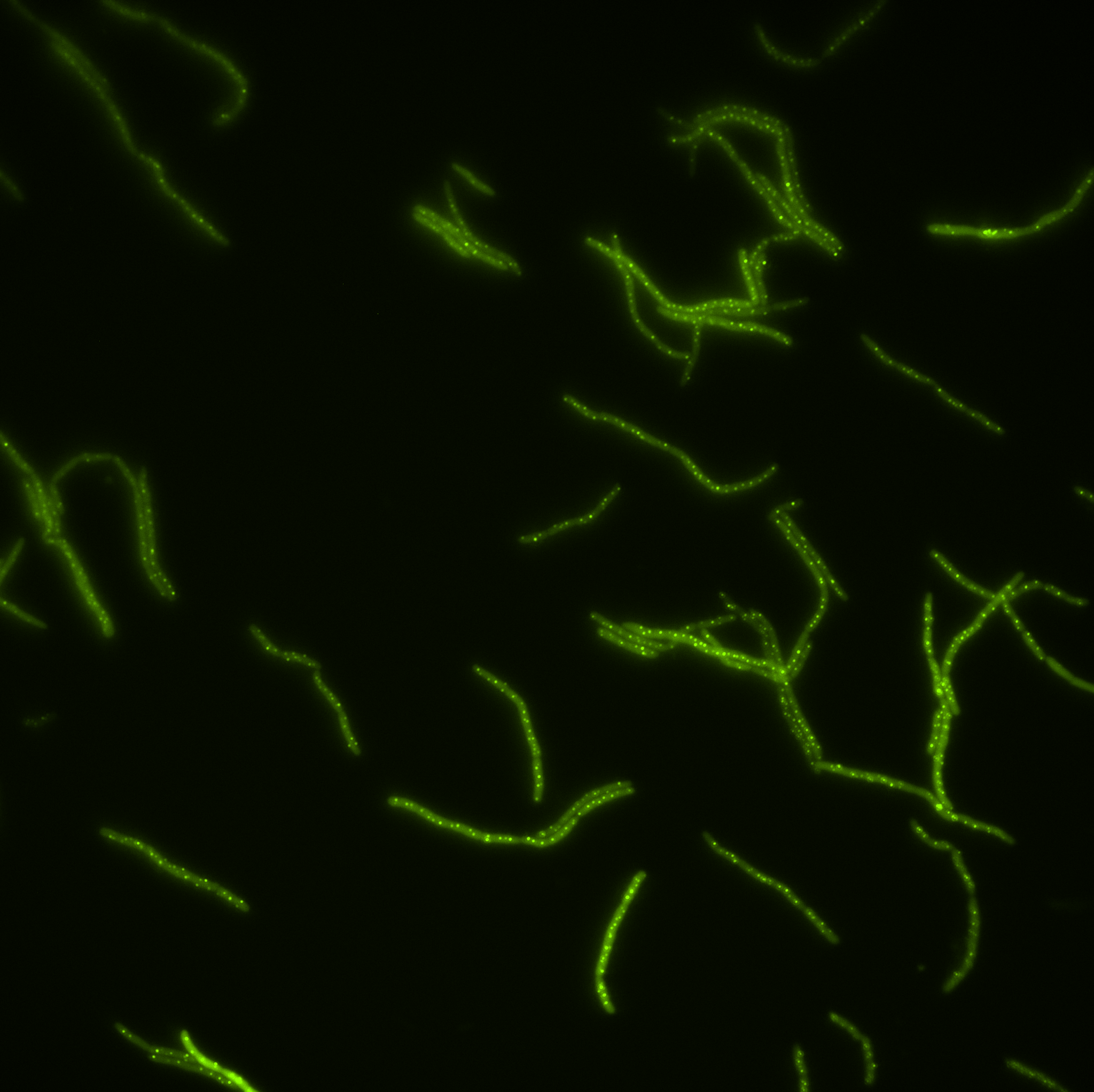

Supplement: Supplementary file 14 — Source data Fig. 2 [file 44321_2025_219_MOESM14_ESM.zip › Figure 2/2E/JD 1708 1.4% 071_RGB_eYFP.tif]

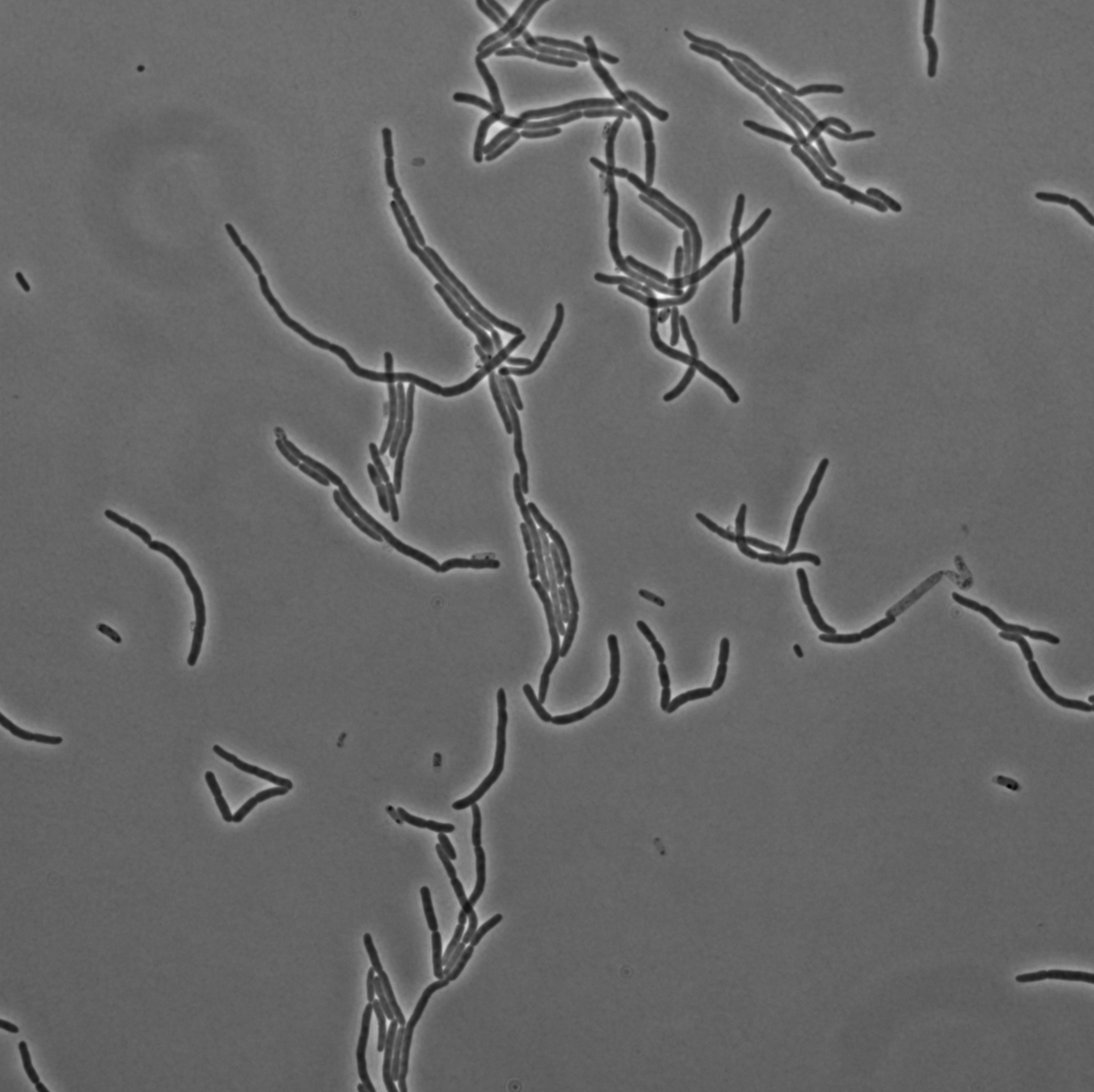

Supplement: Supplementary file 14 — Source data Fig. 2 [file 44321_2025_219_MOESM14_ESM.zip › Figure 2/2E/JD 1708 1.4% 076_RGB_Brightfield.tif]

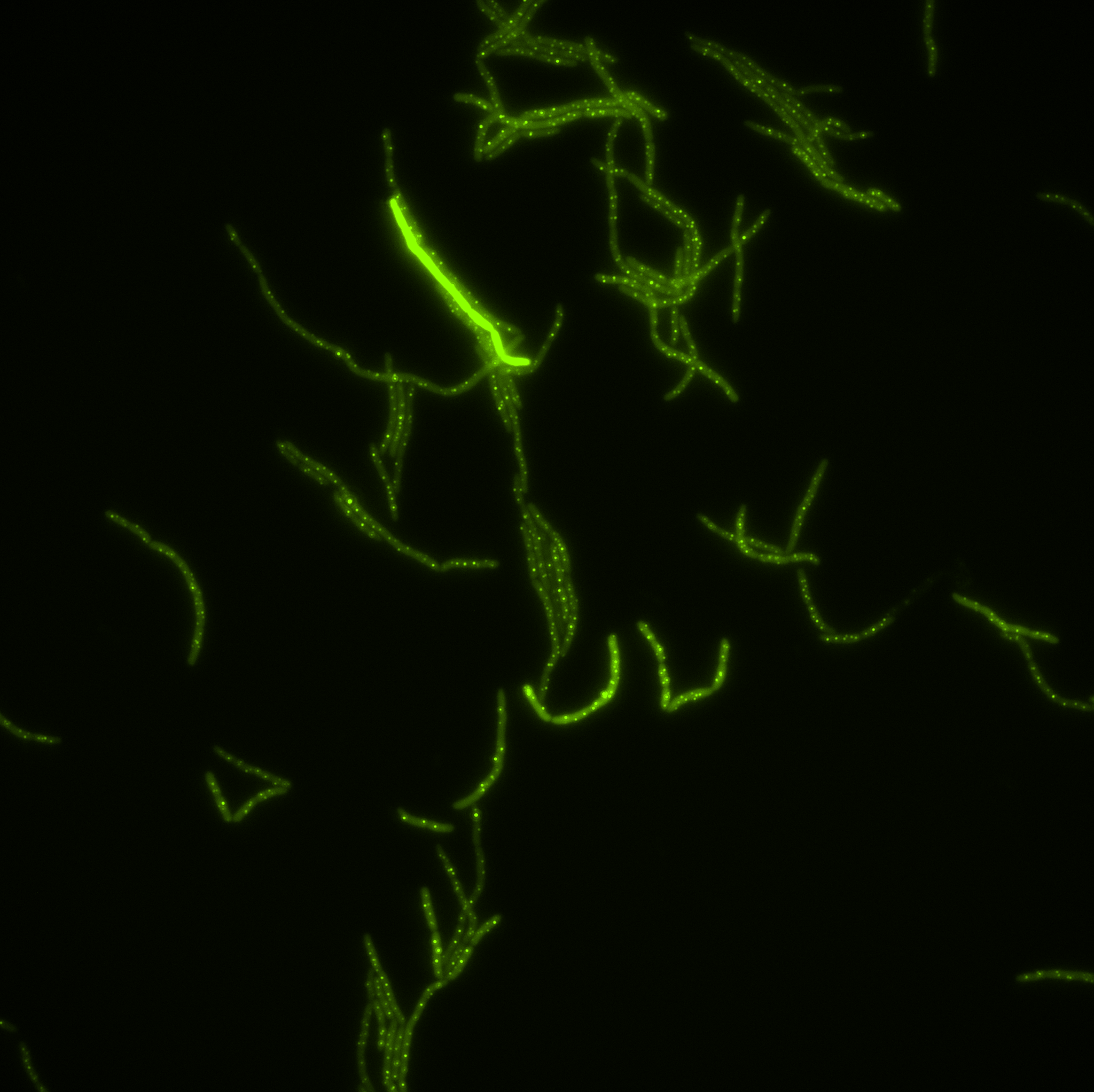

Supplement: Supplementary file 14 — Source data Fig. 2 [file 44321_2025_219_MOESM14_ESM.zip › Figure 2/2E/JD 1708 1.4% 076_RGB_eYFP.tif]

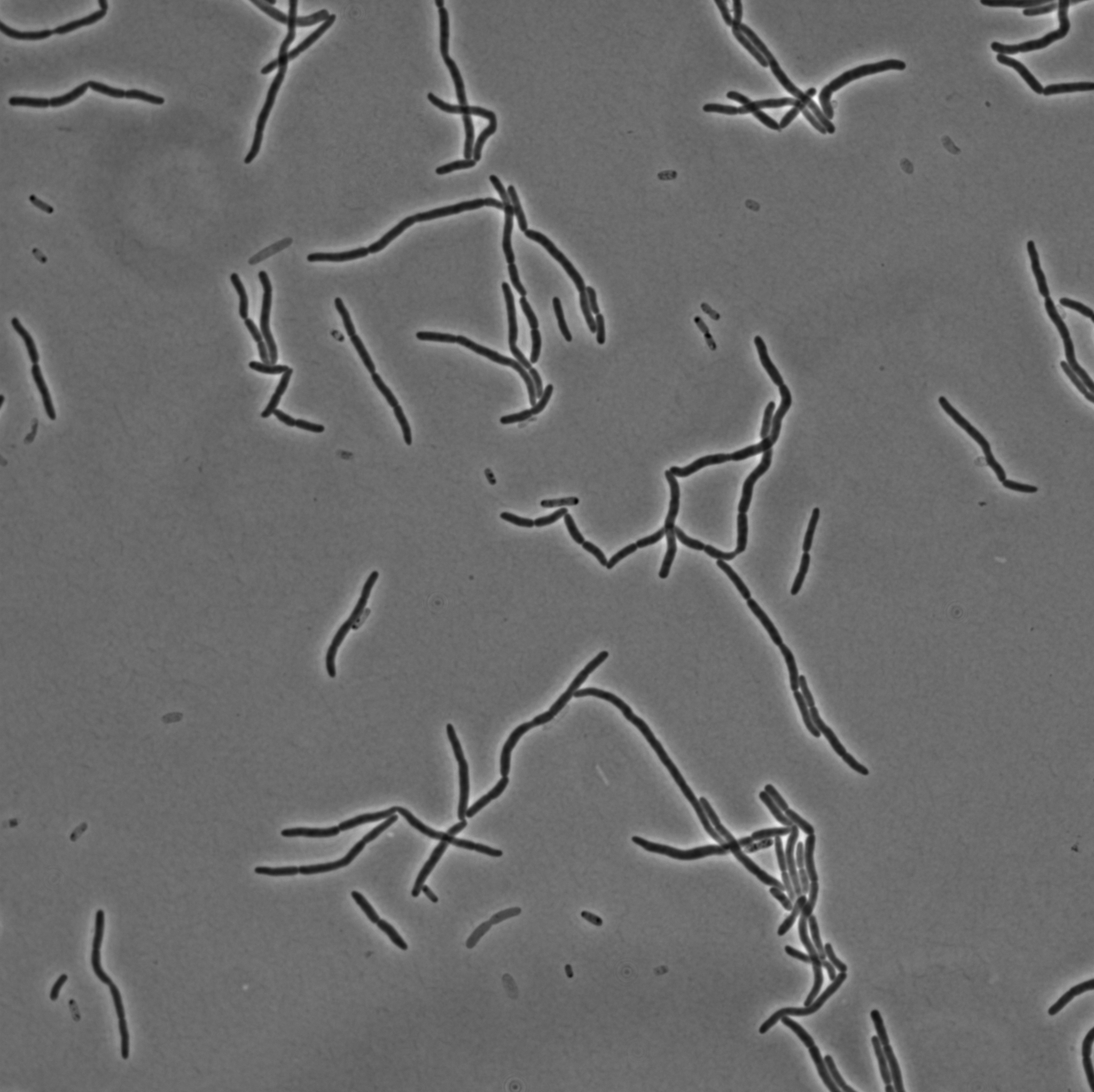

Supplement: Supplementary file 14 — Source data Fig. 2 [file 44321_2025_219_MOESM14_ESM.zip › Figure 2/2E/JD 1708 1.4% 077_RGB_Brightfield.tif]

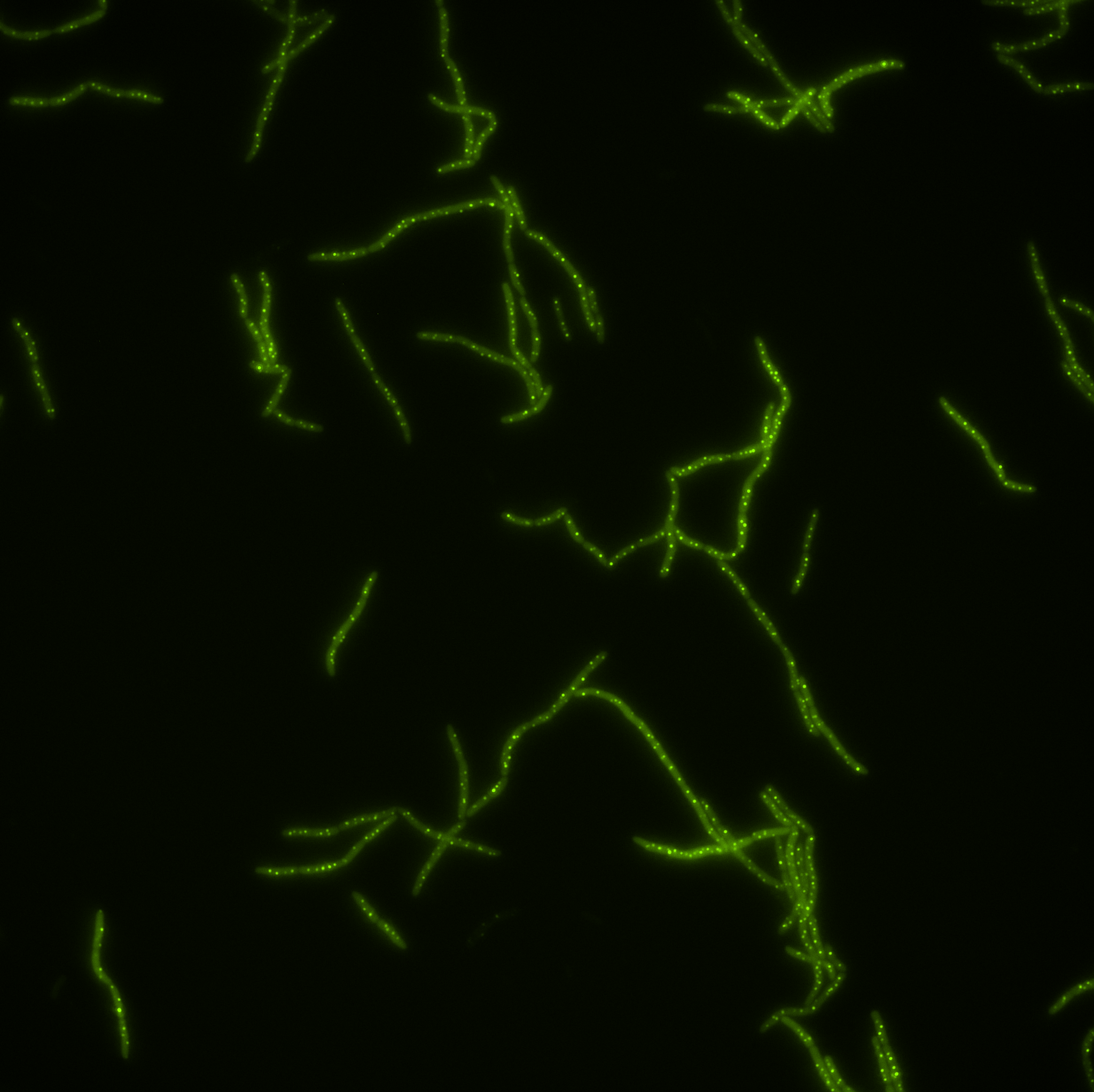

Supplement: Supplementary file 14 — Source data Fig. 2 [file 44321_2025_219_MOESM14_ESM.zip › Figure 2/2E/JD 1708 1.4% 077_RGB_eYFP.tif]

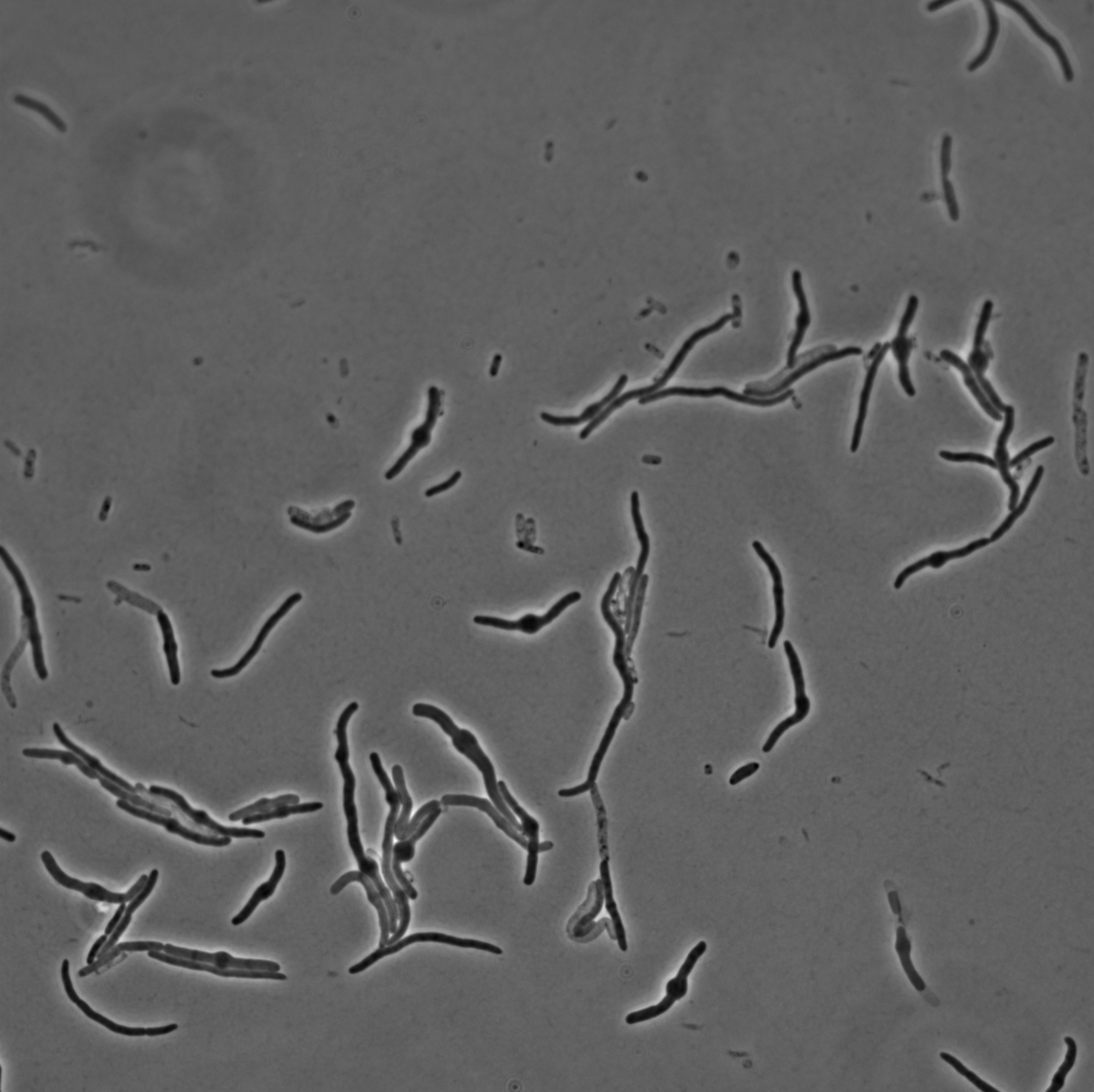

Supplement: Supplementary file 14 — Source data Fig. 2 [file 44321_2025_219_MOESM14_ESM.zip › Figure 2/2E/JD 1708 2% 073_RGB_Brightfield.tif]

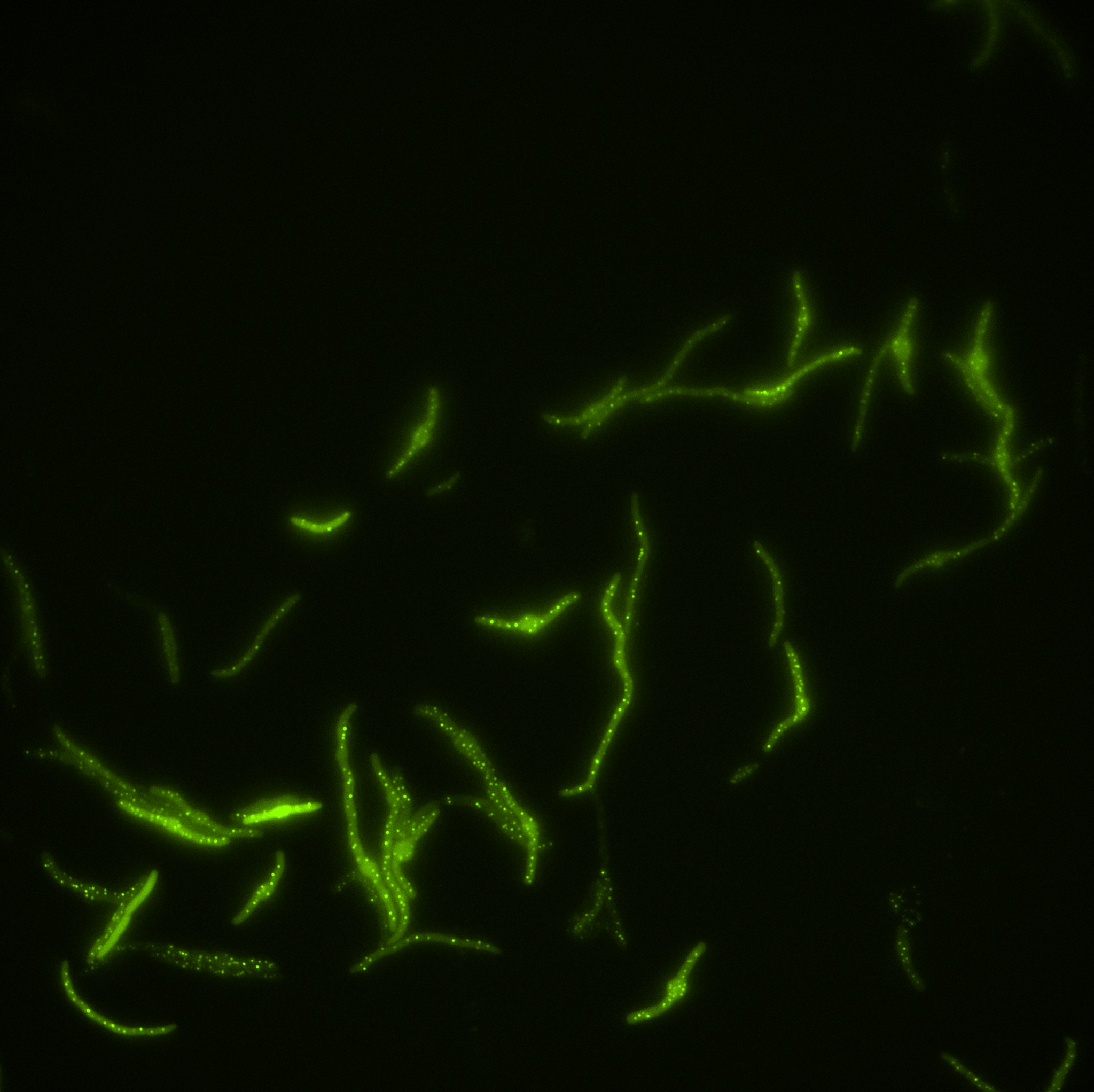

Supplement: Supplementary file 14 — Source data Fig. 2 [file 44321_2025_219_MOESM14_ESM.zip › Figure 2/2E/JD 1708 2% 073_RGB_eYFP.tif]

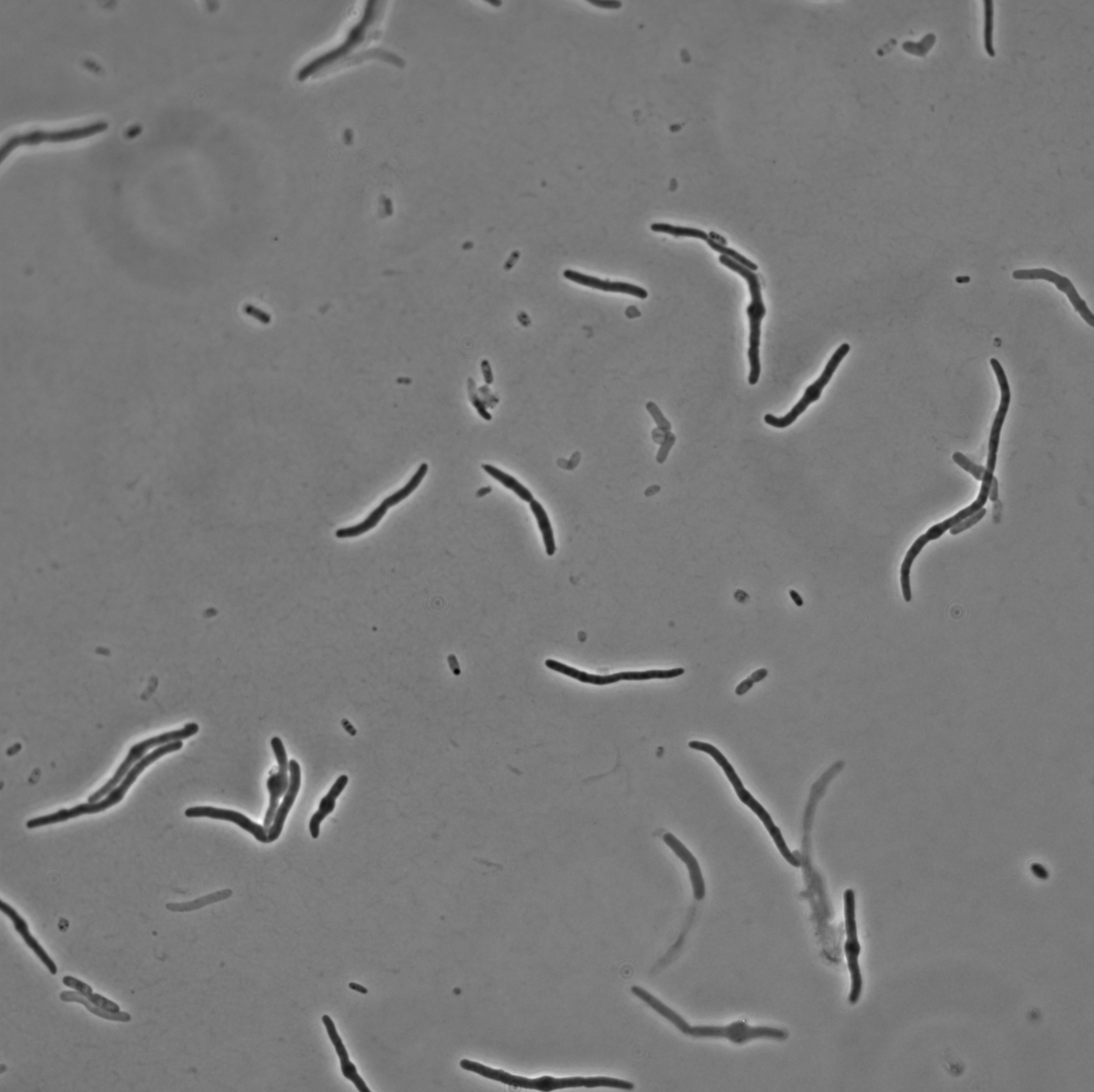

Supplement: Supplementary file 14 — Source data Fig. 2 [file 44321_2025_219_MOESM14_ESM.zip › Figure 2/2E/JD 1708 2% 074_RGB_Brightfield.tif]

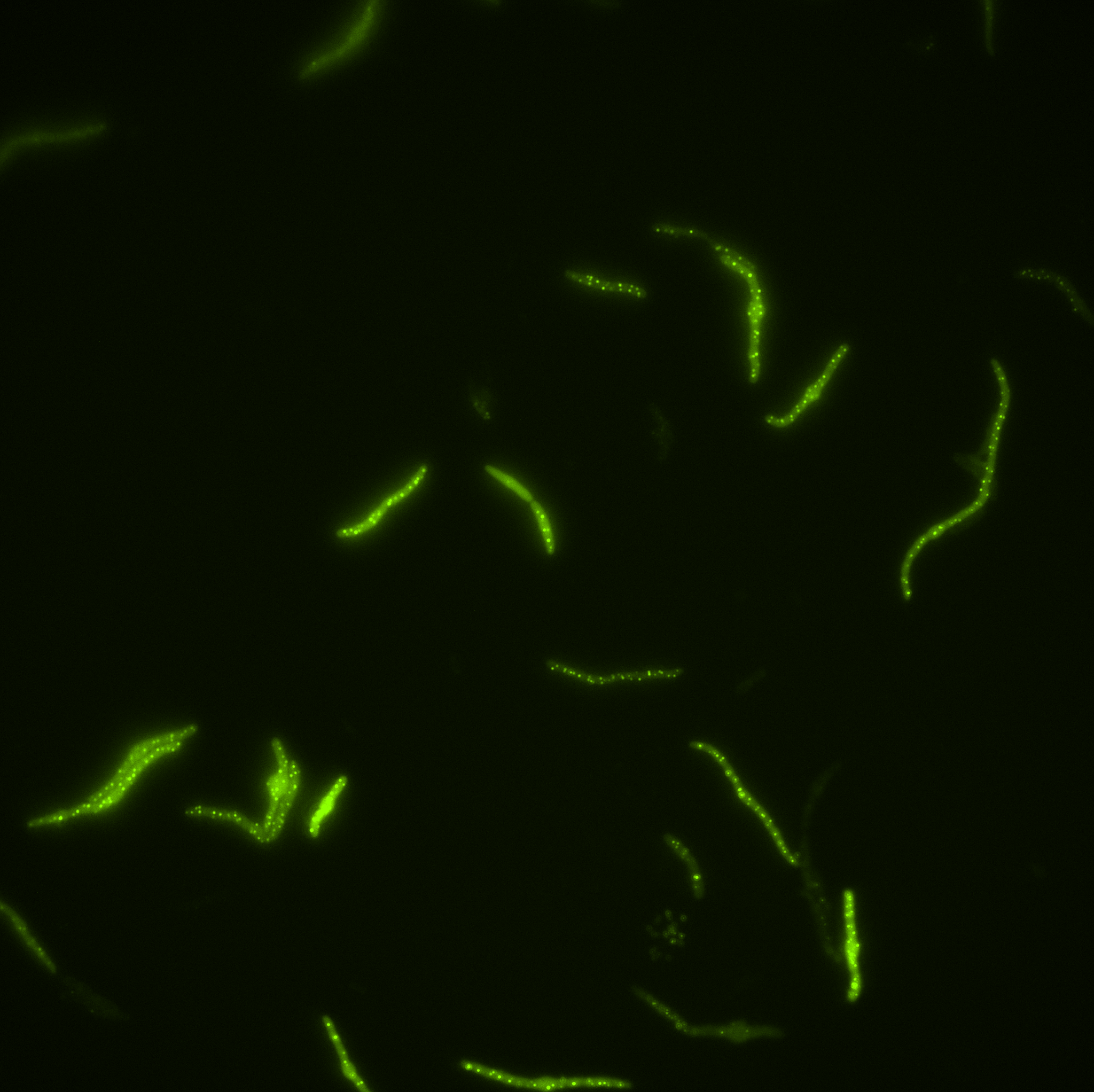

Supplement: Supplementary file 14 — Source data Fig. 2 [file 44321_2025_219_MOESM14_ESM.zip › Figure 2/2E/JD 1708 2% 074_RGB_eYFP.tif]

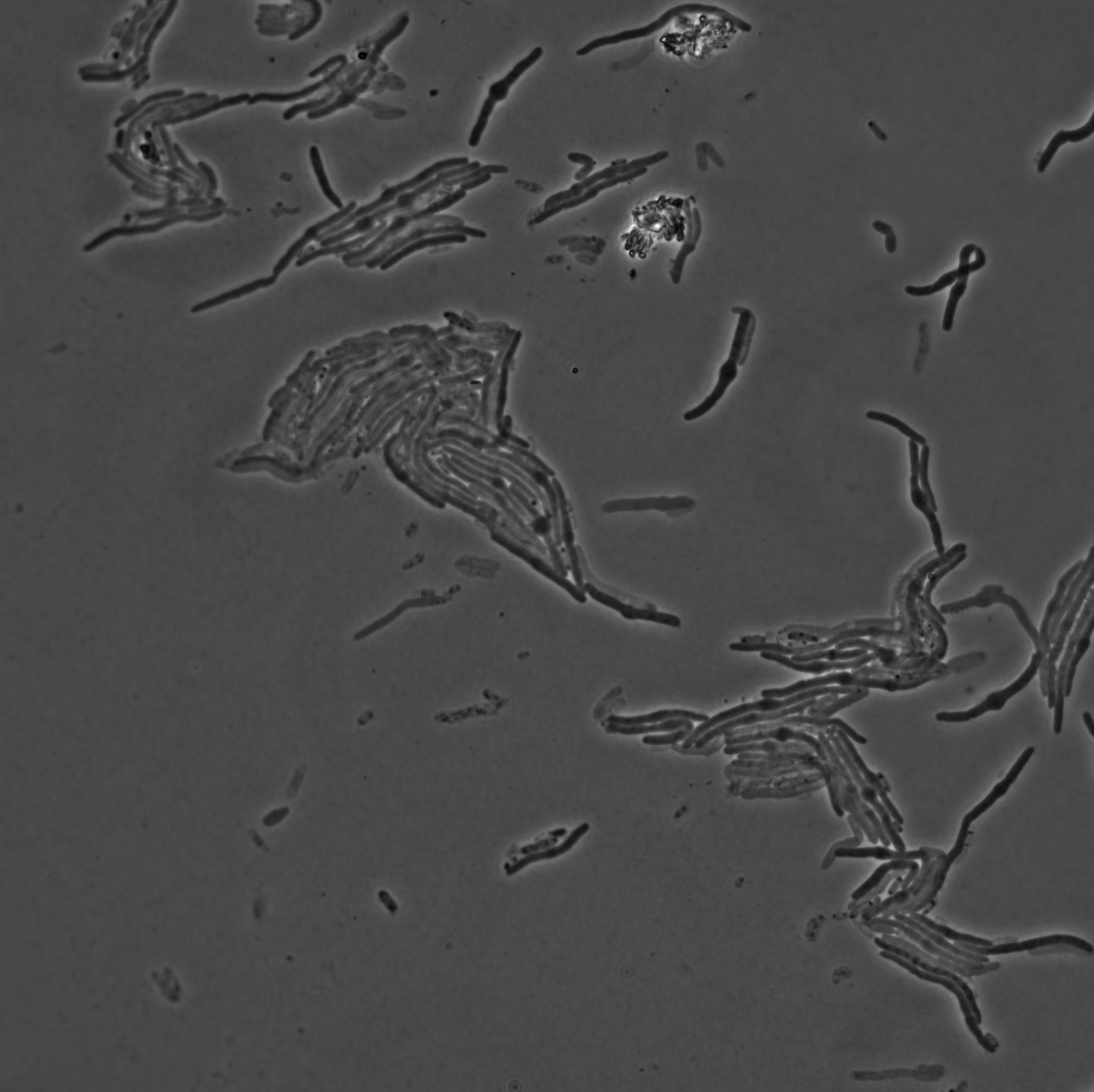

Supplement: Supplementary file 14 — Source data Fig. 2 [file 44321_2025_219_MOESM14_ESM.zip › Figure 2/2E/JD 1708 2% 075_RGB_Brightfield.tif]

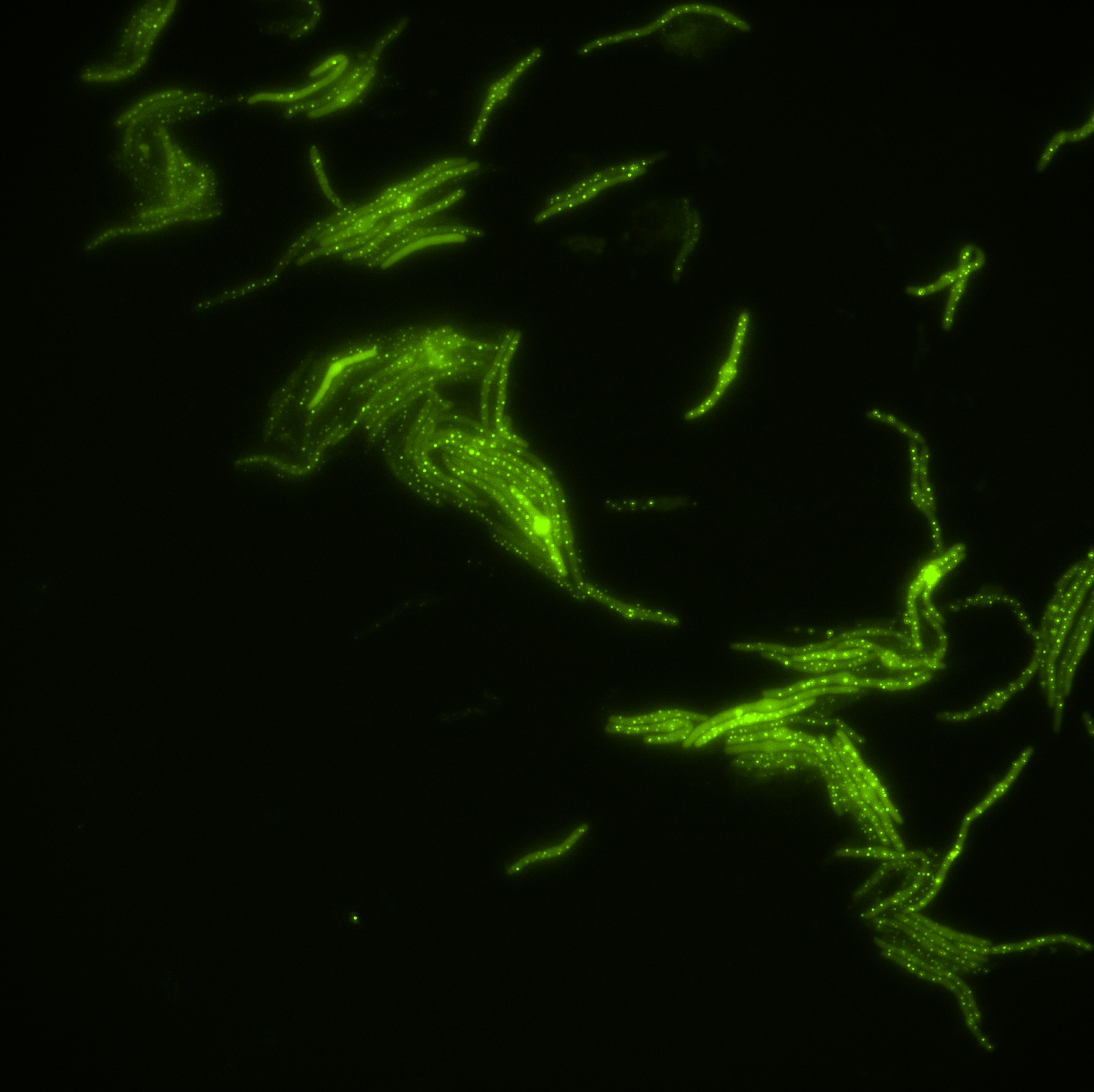

Supplement: Supplementary file 14 — Source data Fig. 2 [file 44321_2025_219_MOESM14_ESM.zip › Figure 2/2E/JD 1708 2% 075_RGB_eYFP.tif]

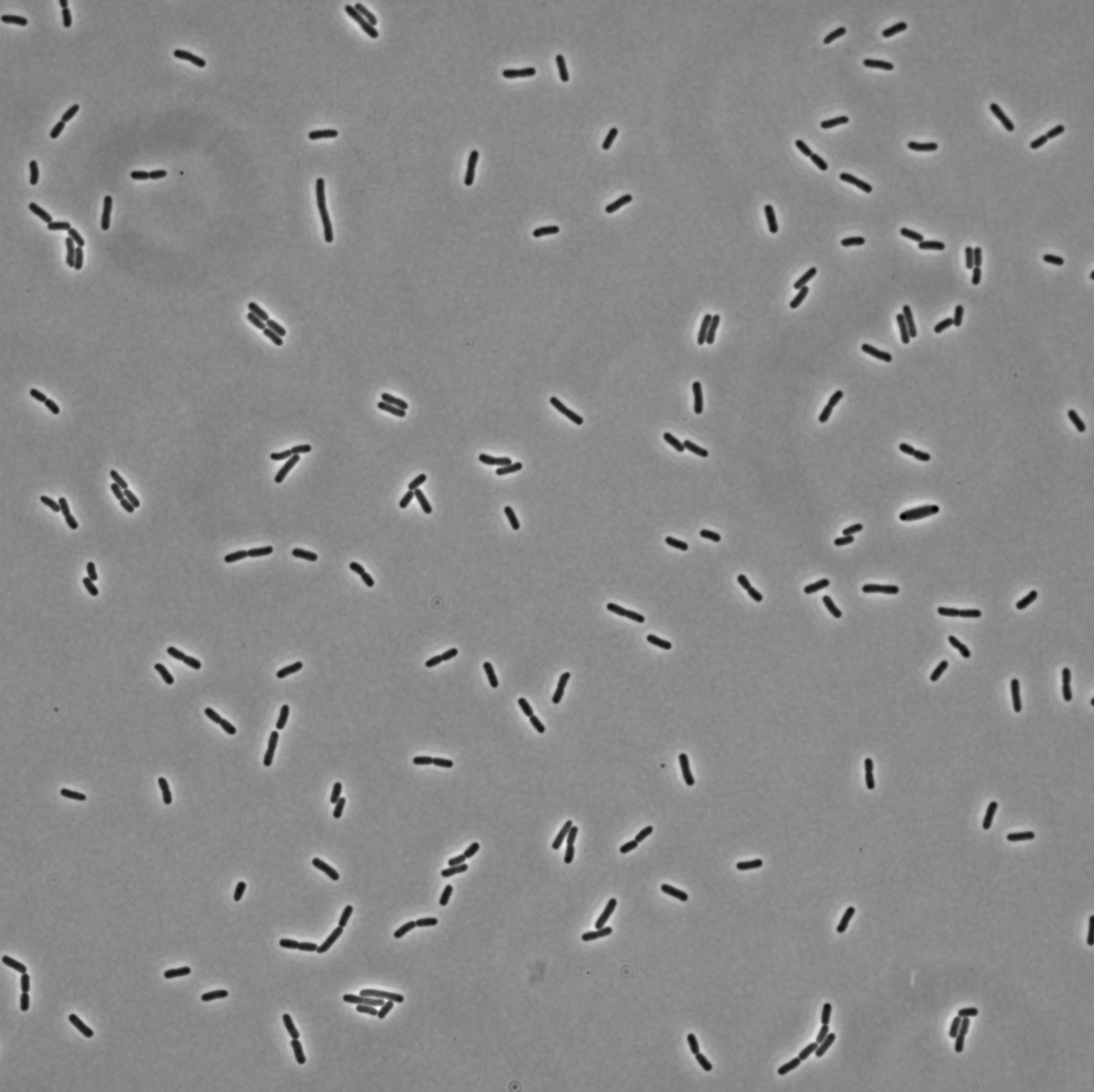

Supplement: Supplementary file 15 — Source data Fig. 3 [file 44321_2025_219_MOESM15_ESM.zip › Figure 3/3A/JD1708 0_1 ara 60 min 0 sac045_RGB_Brightfield.tif]

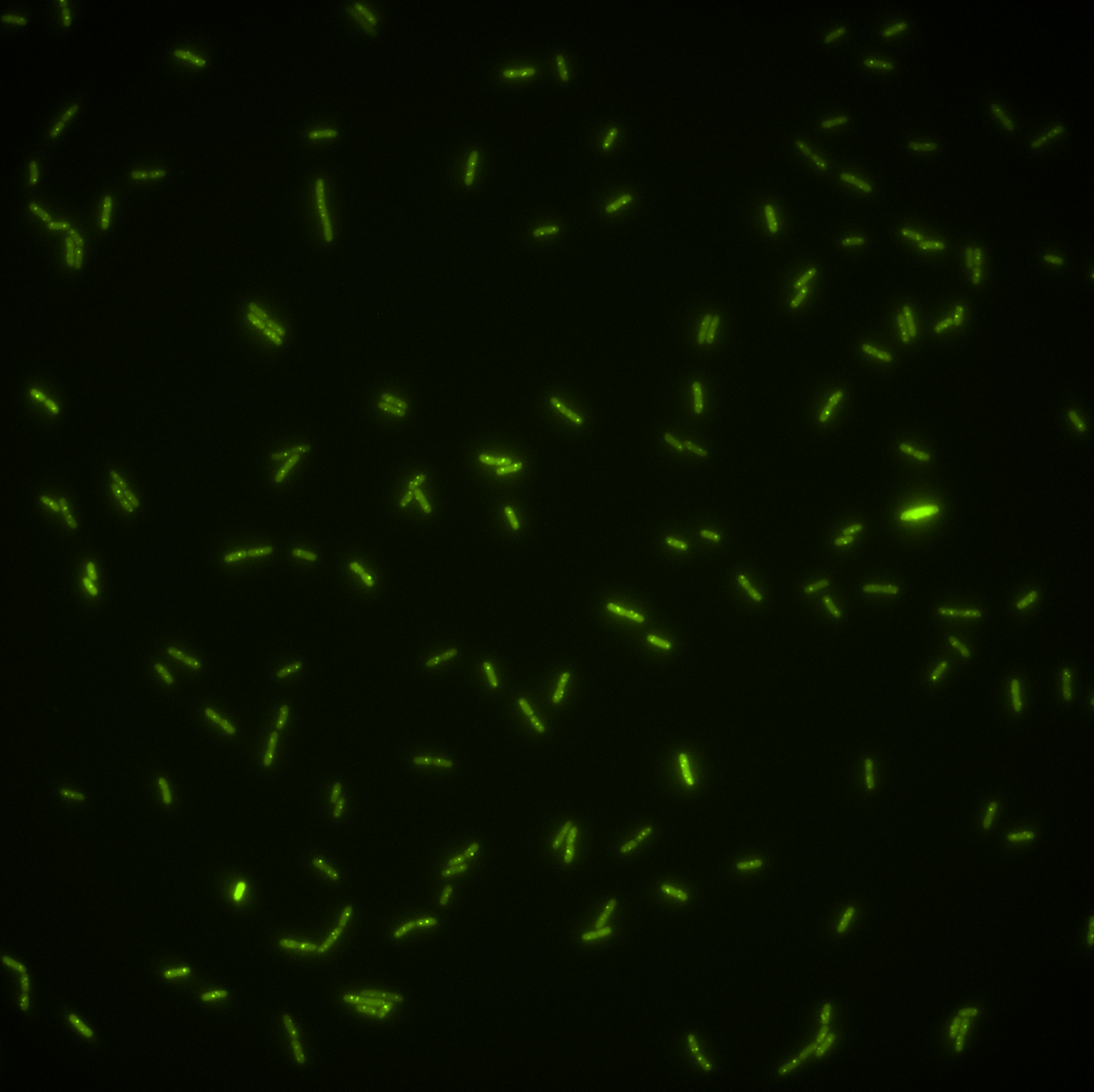

Supplement: Supplementary file 15 — Source data Fig. 3 [file 44321_2025_219_MOESM15_ESM.zip › Figure 3/3A/JD1708 0_1 ara 60 min 0 sac045_RGB_eYFP.tif]

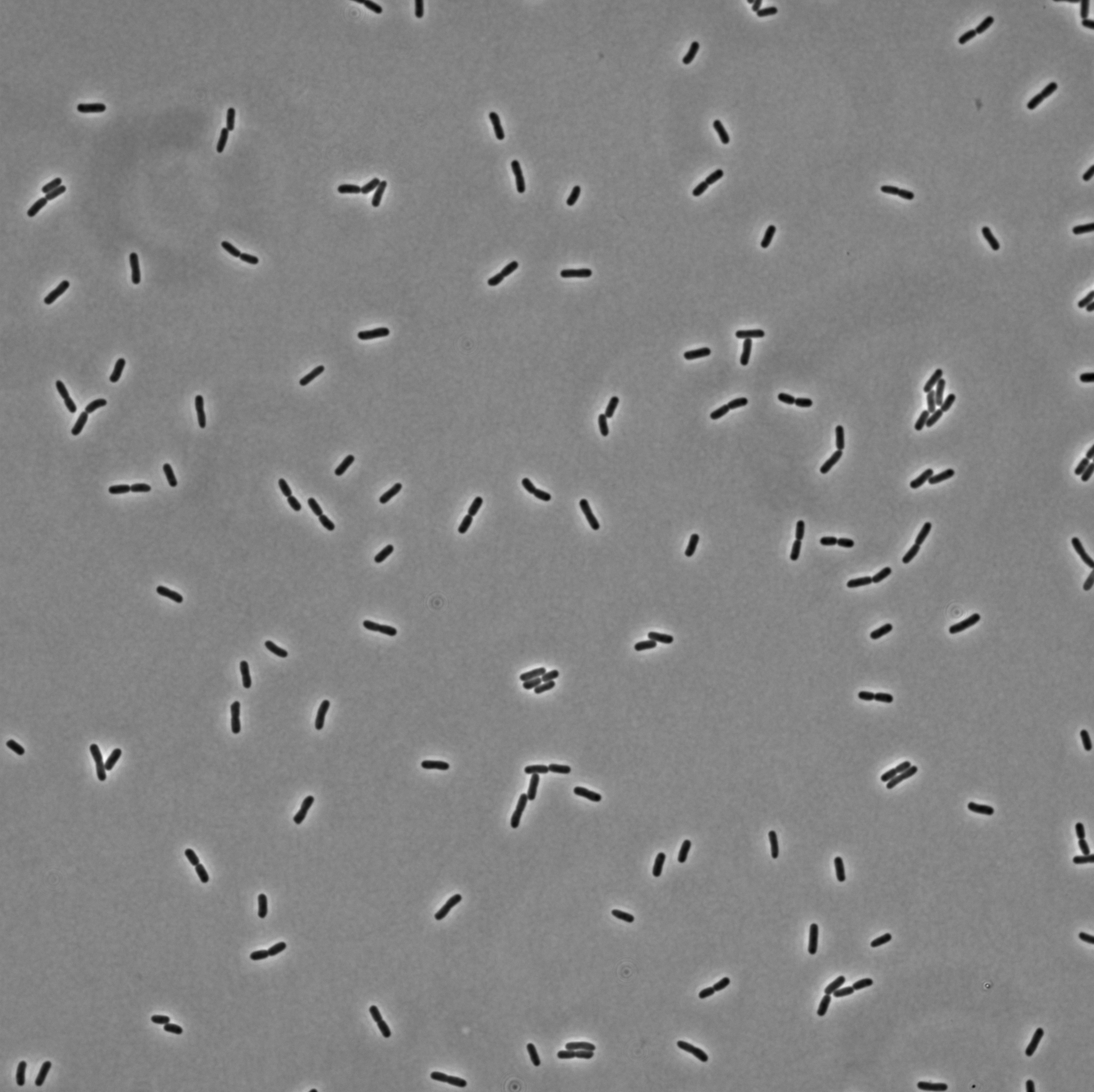

Supplement: Supplementary file 15 — Source data Fig. 3 [file 44321_2025_219_MOESM15_ESM.zip › Figure 3/3A/JD1708 0_1 ara 60 min 0 sac046_RGB_Brightfield.tif]

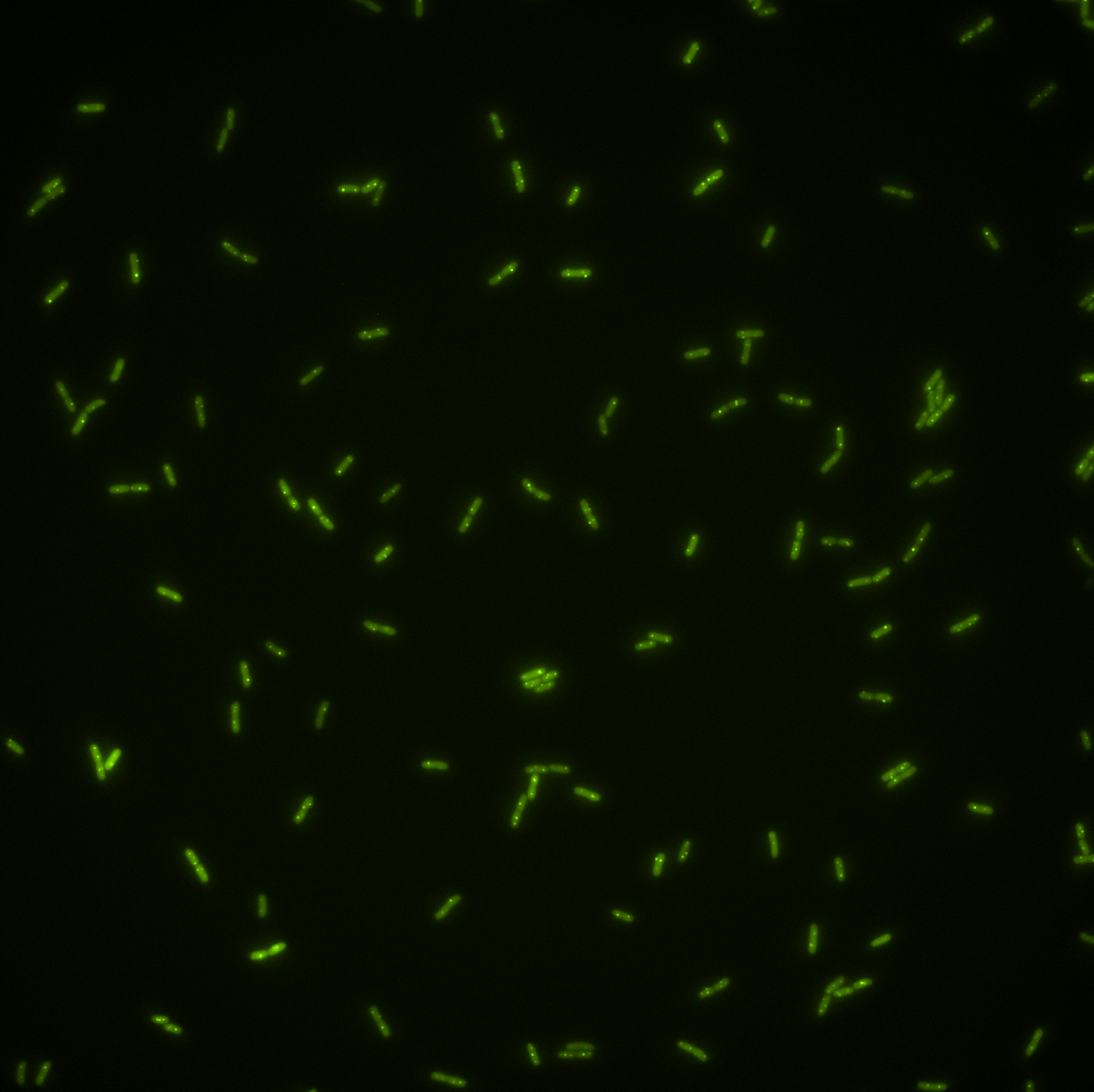

Supplement: Supplementary file 15 — Source data Fig. 3 [file 44321_2025_219_MOESM15_ESM.zip › Figure 3/3A/JD1708 0_1 ara 60 min 0 sac046_RGB_eYFP.tif]

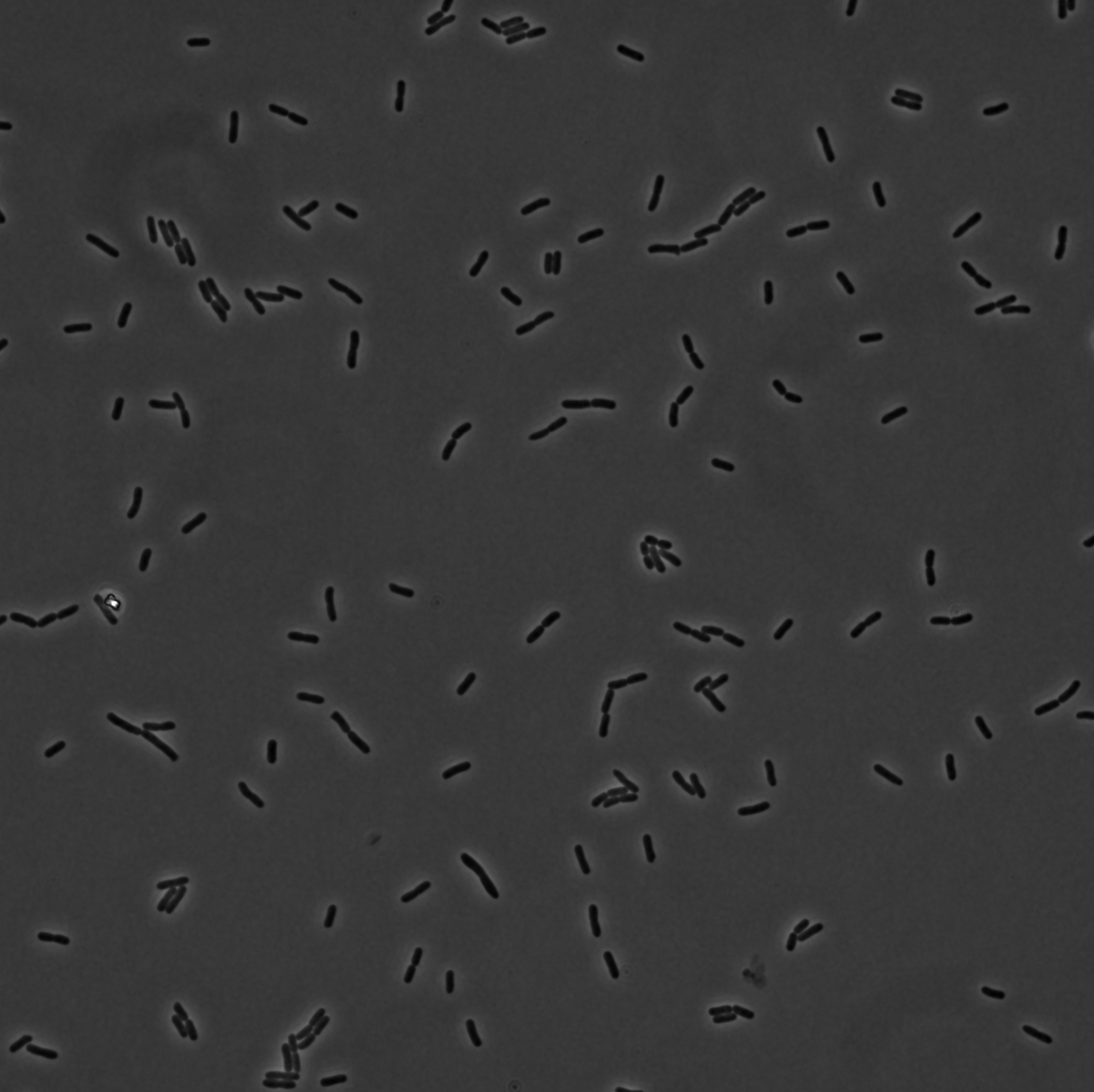

Supplement: Supplementary file 15 — Source data Fig. 3 [file 44321_2025_219_MOESM15_ESM.zip › Figure 3/3A/JD1708 0_1 ara 60 min 0 sac047_RGB_Brightfield.tif]

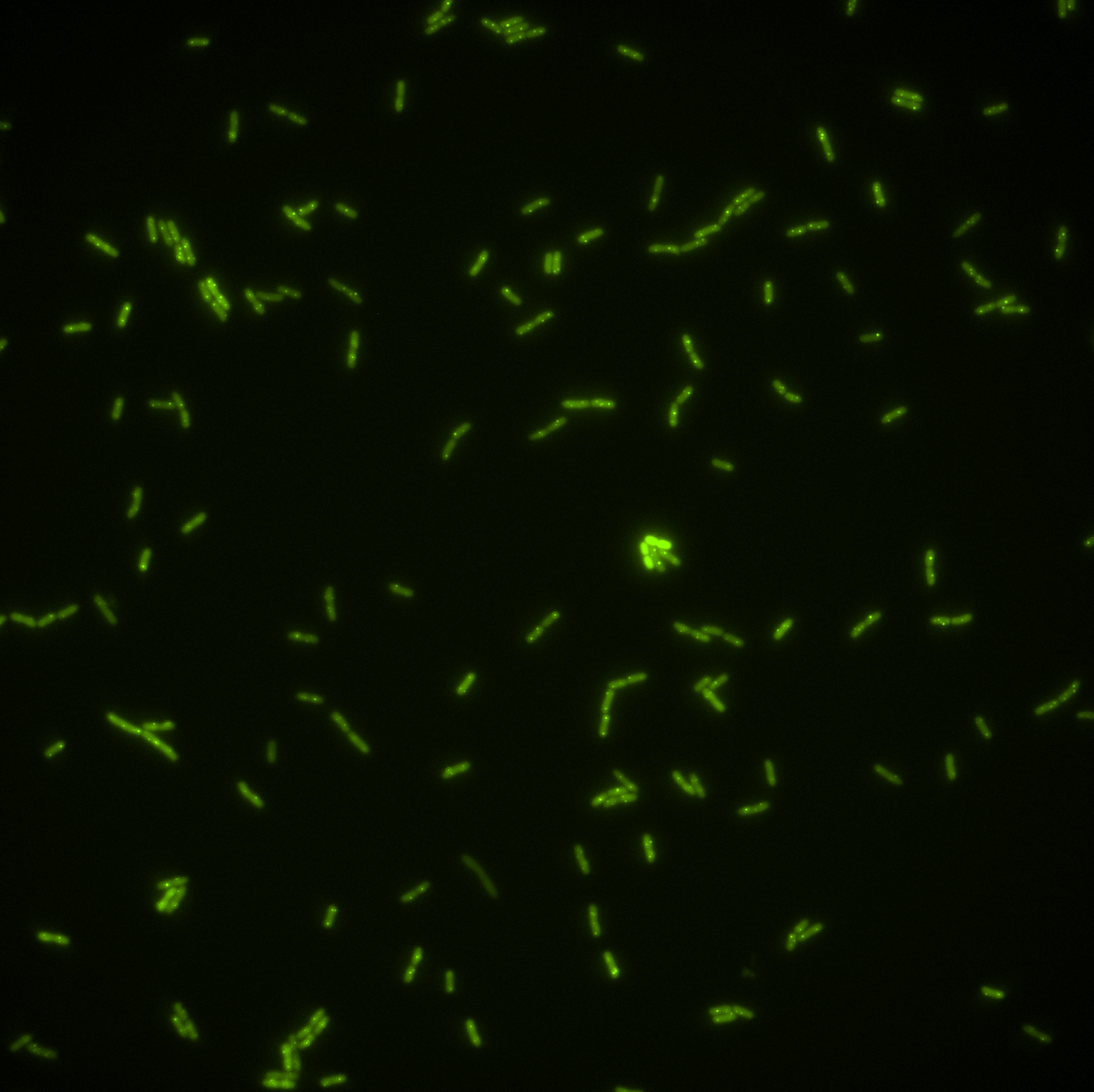

Supplement: Supplementary file 15 — Source data Fig. 3 [file 44321_2025_219_MOESM15_ESM.zip › Figure 3/3A/JD1708 0_1 ara 60 min 0 sac047_RGB_eYFP.tif]

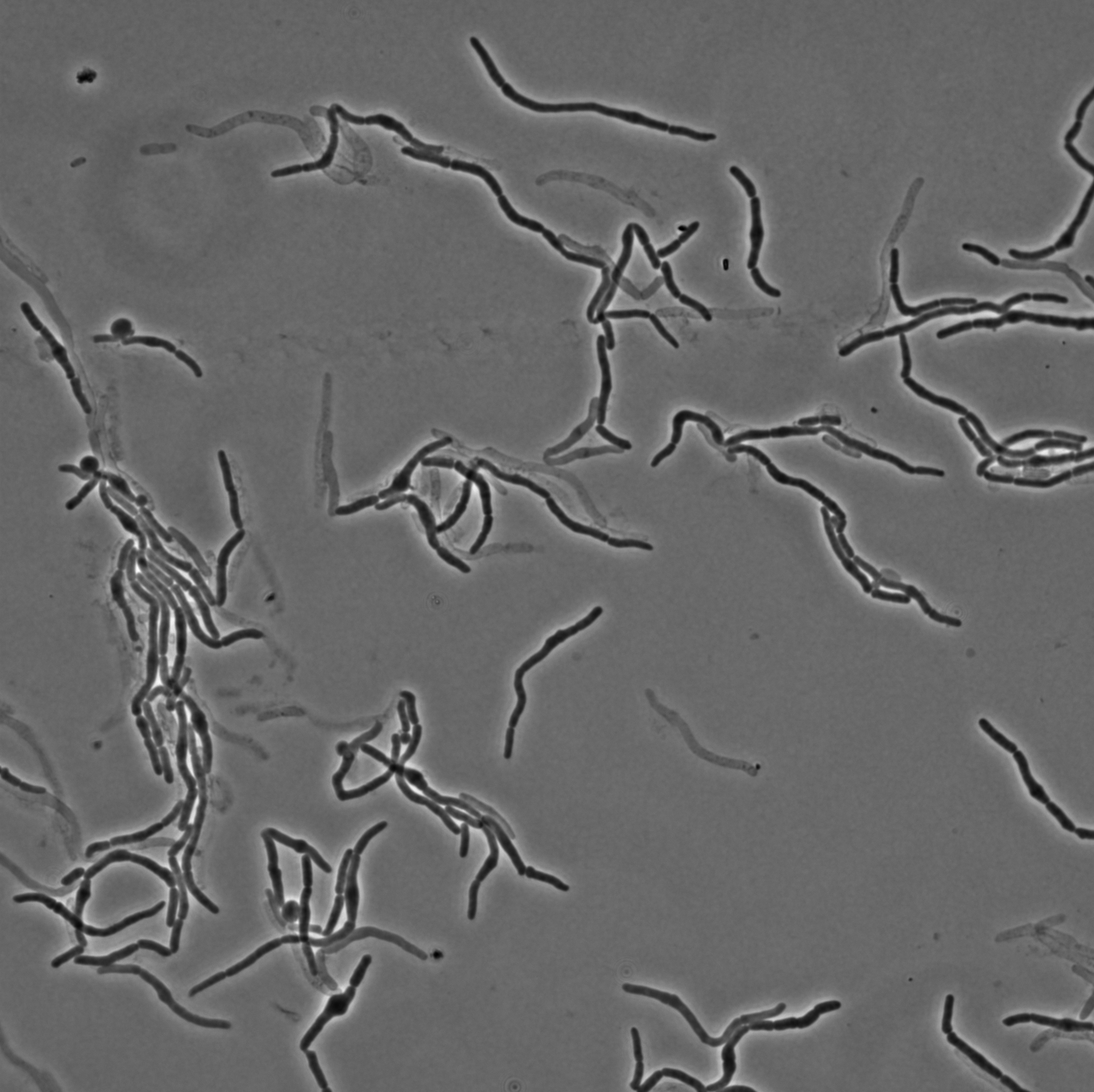

Supplement: Supplementary file 15 — Source data Fig. 3 [file 44321_2025_219_MOESM15_ESM.zip › Figure 3/3A/JD1708 0_1 ara 60 min 1_4 sac049_RGB_Brightfield.tif]

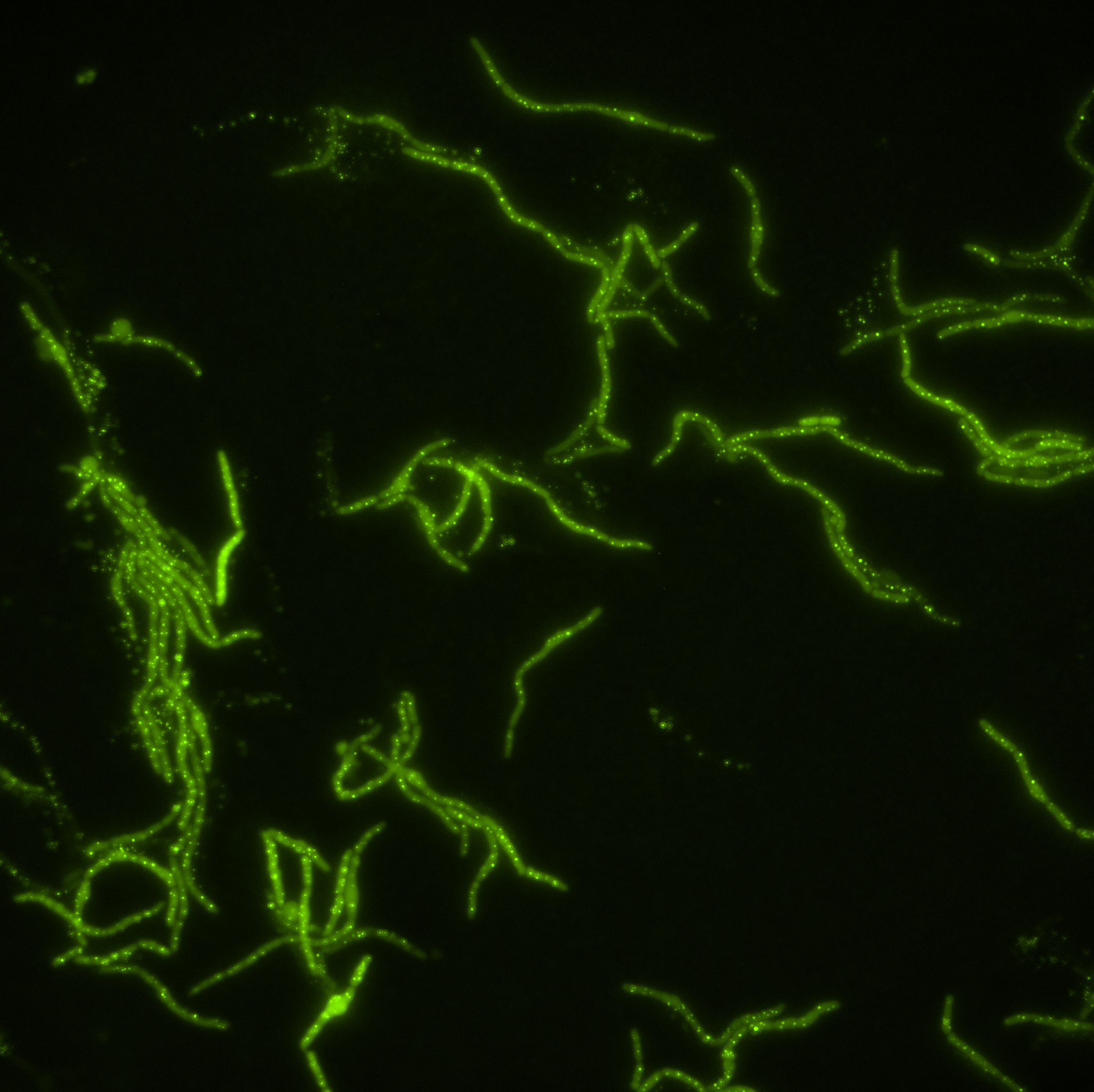

Supplement: Supplementary file 15 — Source data Fig. 3 [file 44321_2025_219_MOESM15_ESM.zip › Figure 3/3A/JD1708 0_1 ara 60 min 1_4 sac049_RGB_eYFP.tif]

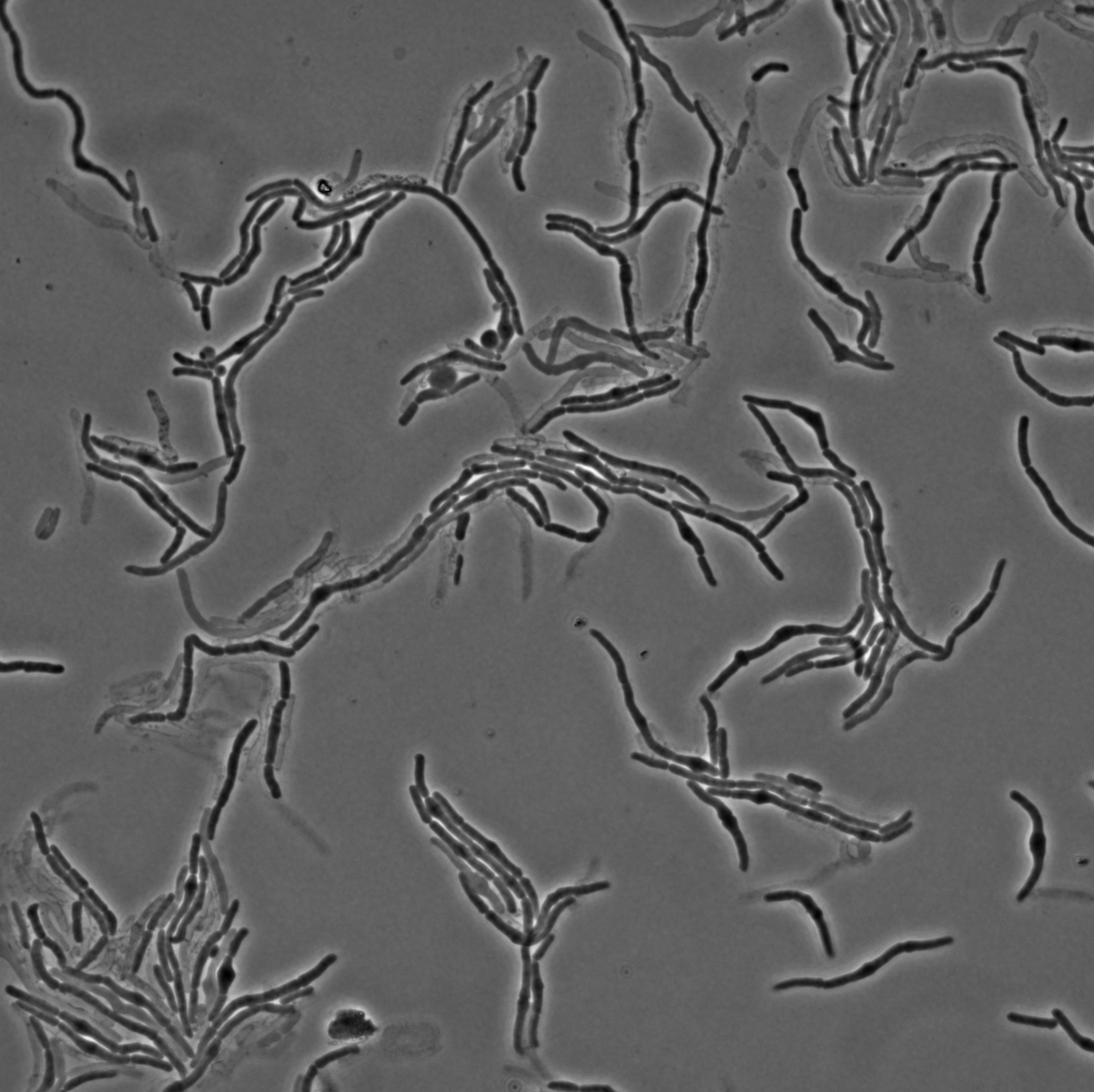

Supplement: Supplementary file 15 — Source data Fig. 3 [file 44321_2025_219_MOESM15_ESM.zip › Figure 3/3A/JD1708 0_1 ara 60 min 1_4 sac050_RGB_Brightfield.tif]

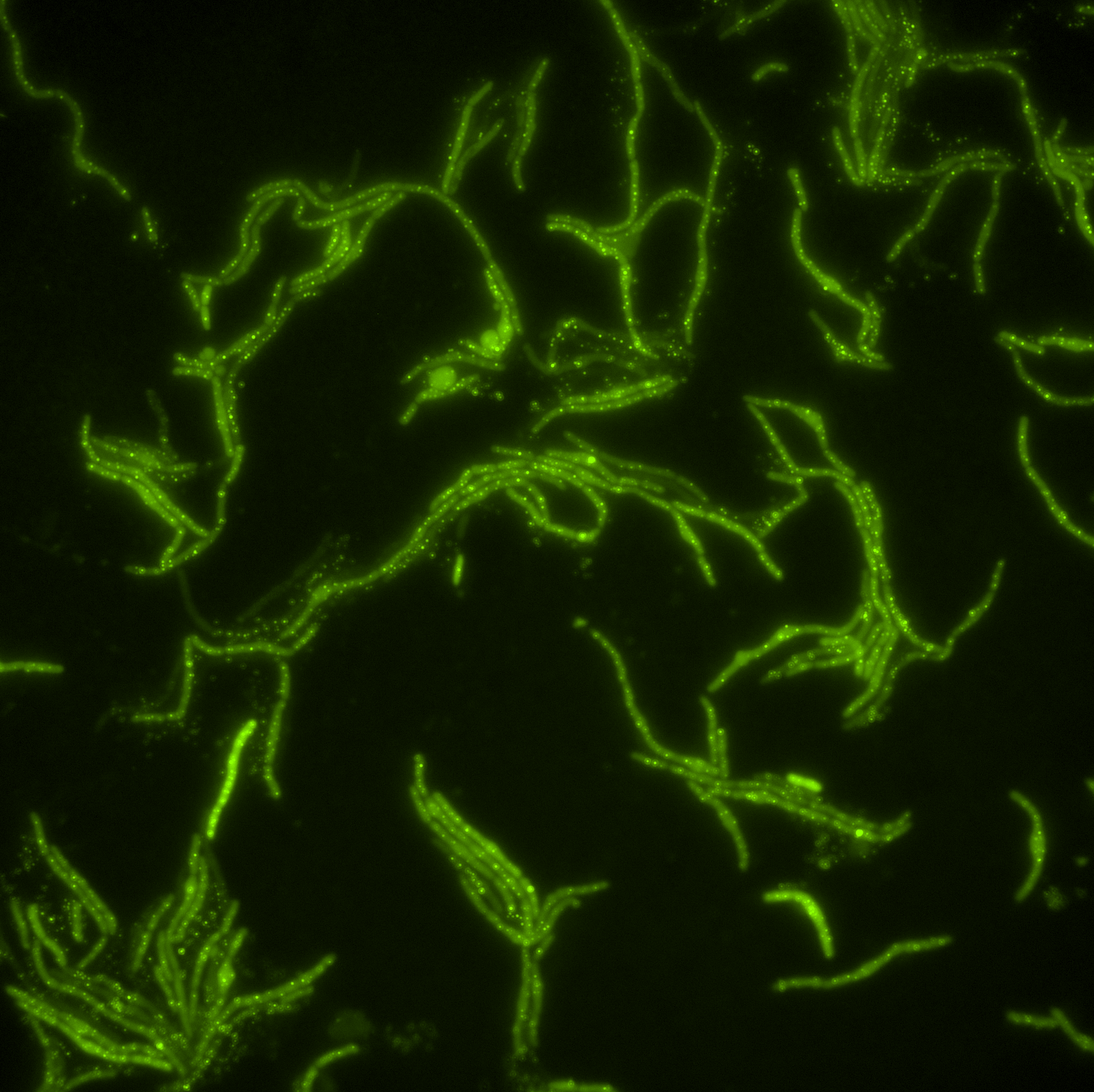

Supplement: Supplementary file 15 — Source data Fig. 3 [file 44321_2025_219_MOESM15_ESM.zip › Figure 3/3A/JD1708 0_1 ara 60 min 1_4 sac050_RGB_eYFP.tif]

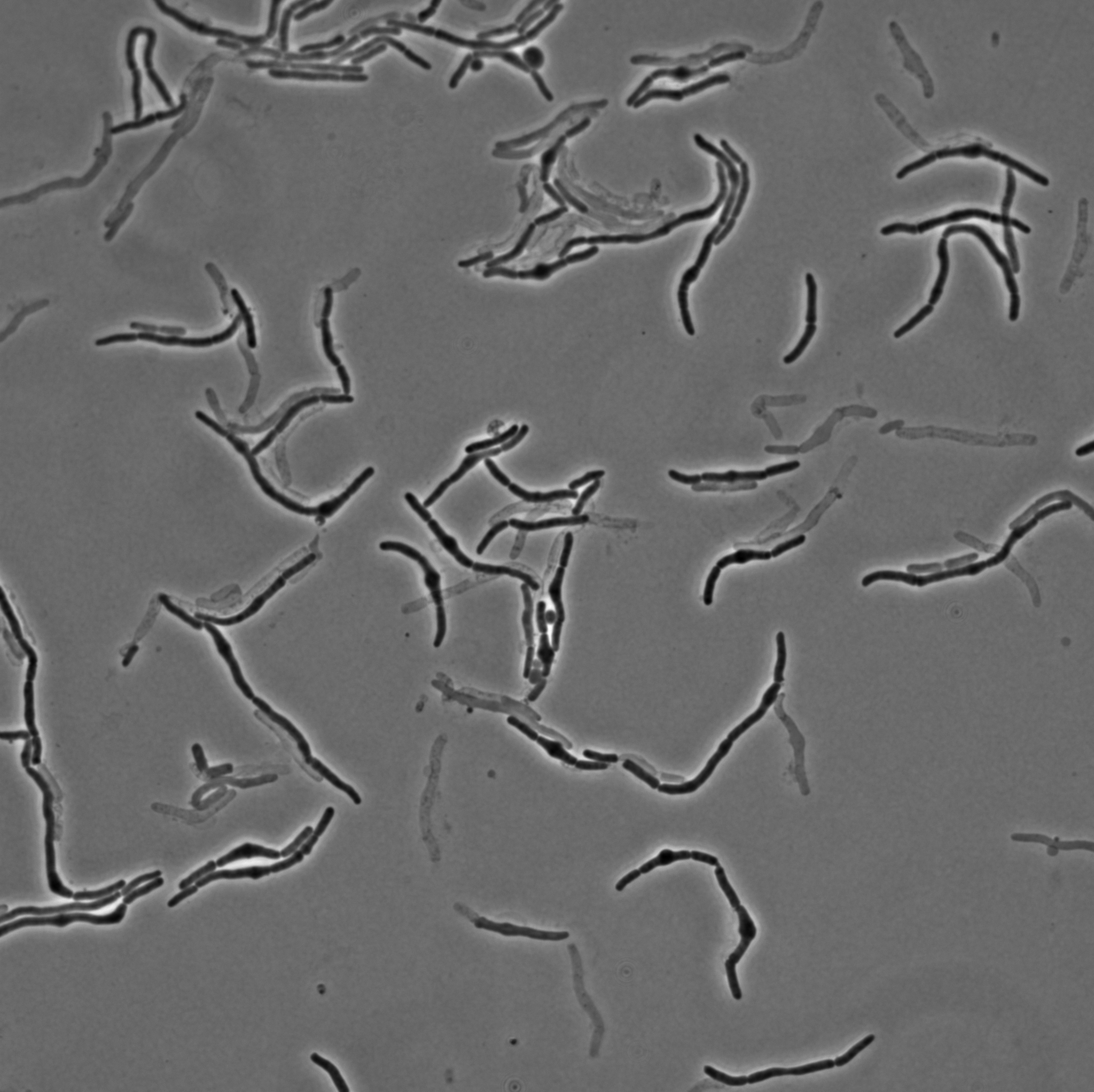

Supplement: Supplementary file 15 — Source data Fig. 3 [file 44321_2025_219_MOESM15_ESM.zip › Figure 3/3A/JD1708 0_1 ara 60 min 1_4 sac051_RGB_Brightfield.tif]

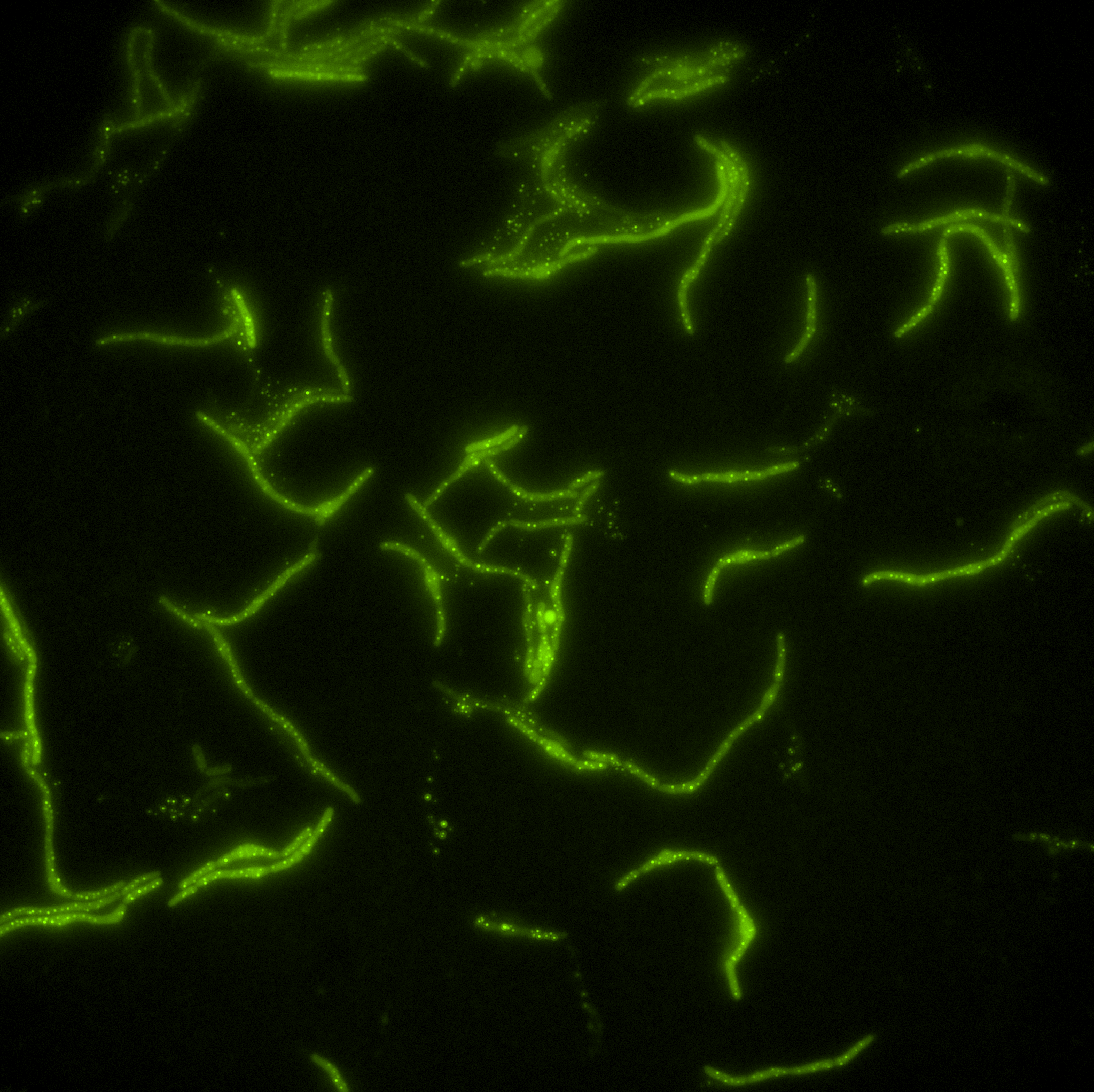

Supplement: Supplementary file 15 — Source data Fig. 3 [file 44321_2025_219_MOESM15_ESM.zip › Figure 3/3A/JD1708 0_1 ara 60 min 1_4 sac051_RGB_eYFP.tif]

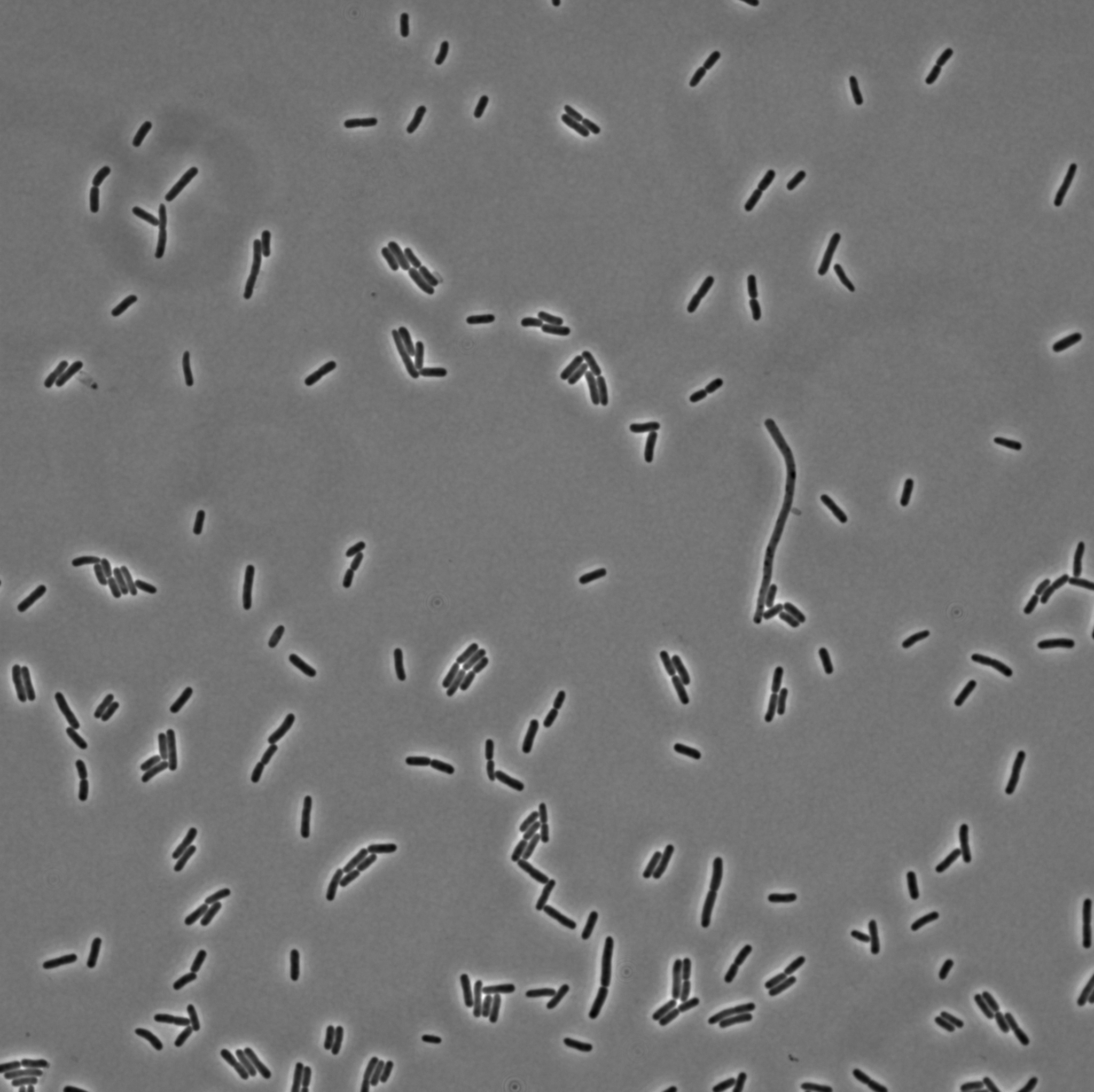

Supplement: Supplementary file 15 — Source data Fig. 3 [file 44321_2025_219_MOESM15_ESM.zip › Figure 3/3A/SP078 0_1 ara 60 min 0 sac039_RGB_Brightfield.tif]

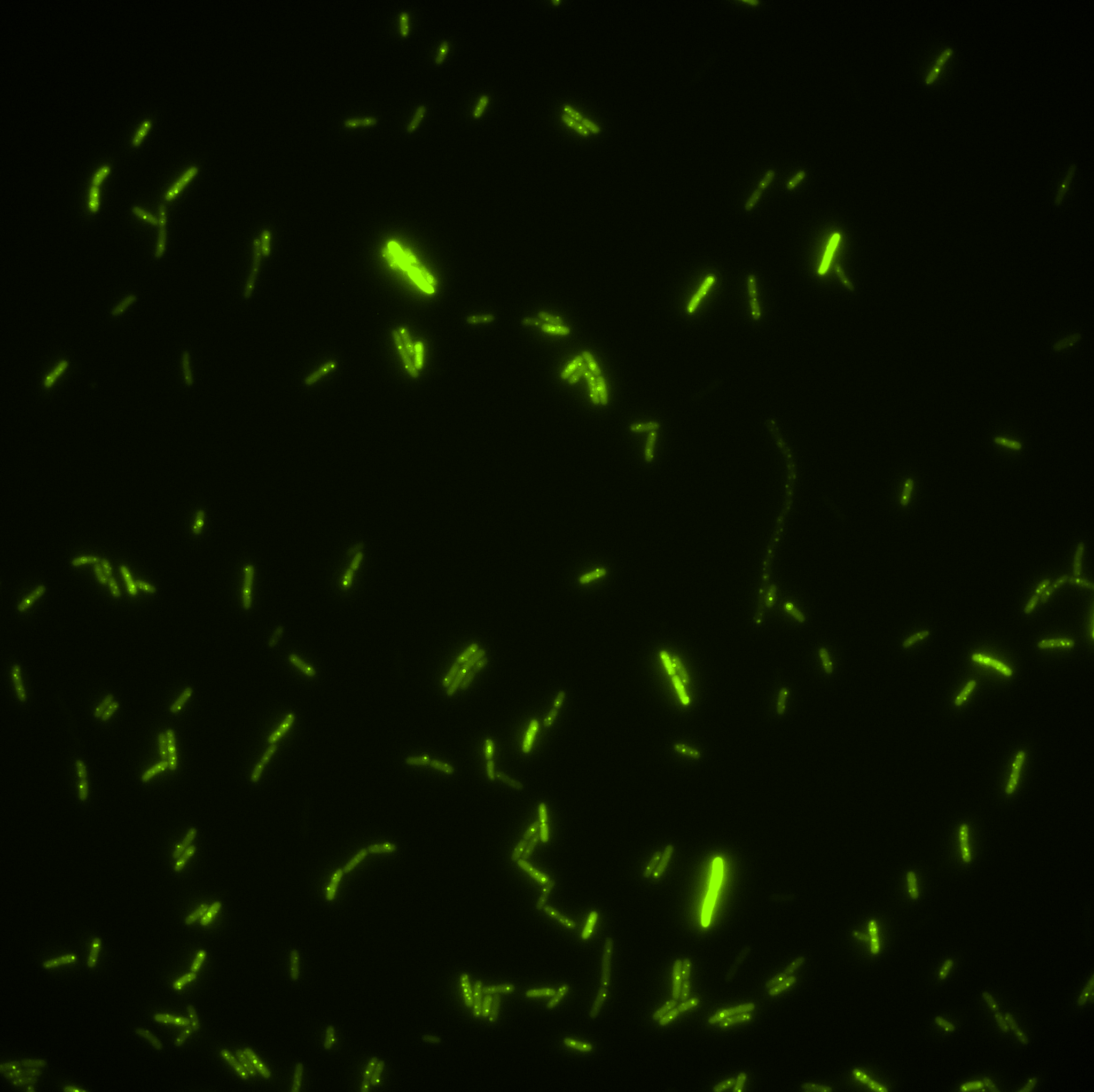

Supplement: Supplementary file 15 — Source data Fig. 3 [file 44321_2025_219_MOESM15_ESM.zip › Figure 3/3A/SP078 0_1 ara 60 min 0 sac039_RGB_eYFP.tif]

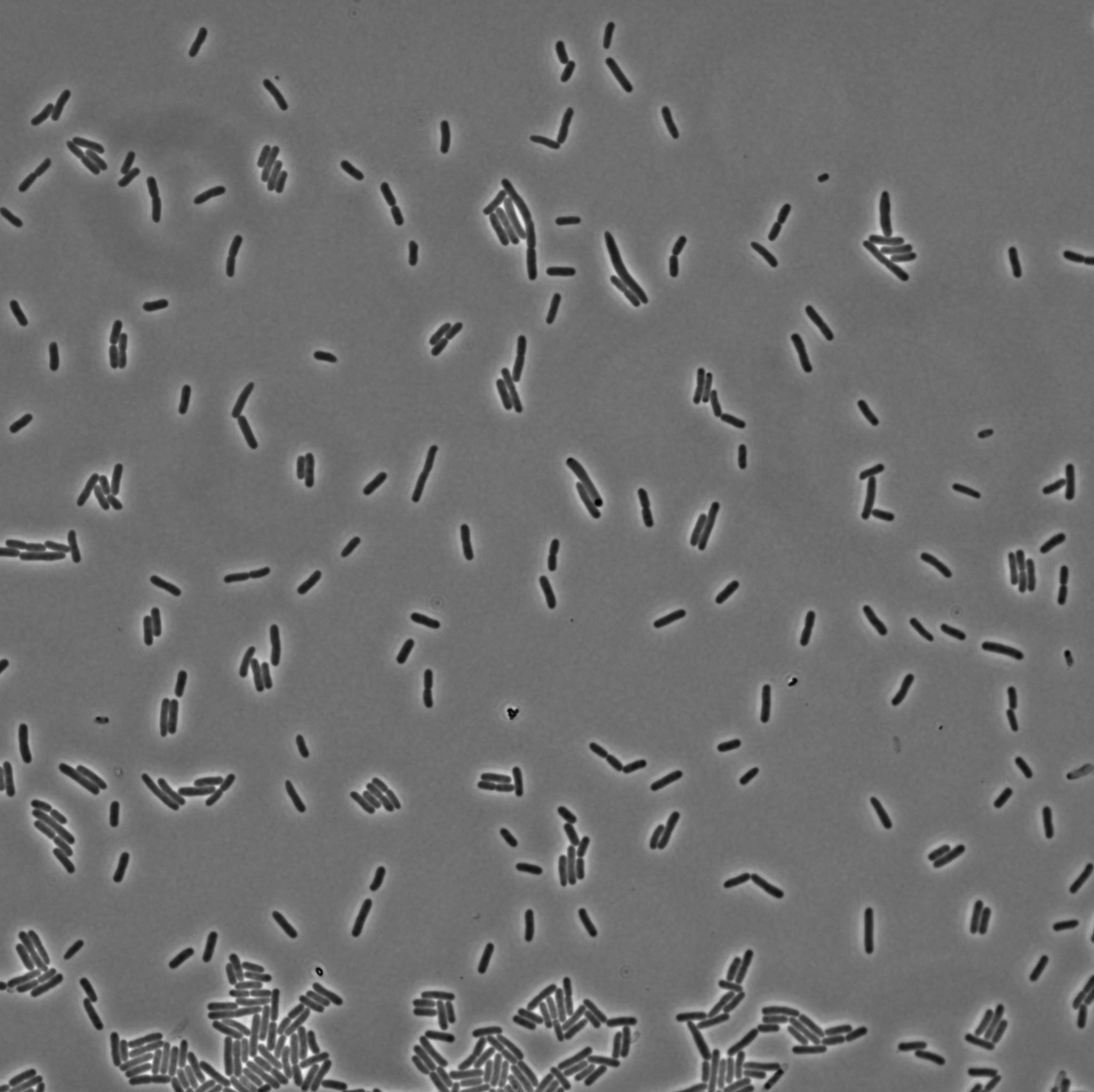

Supplement: Supplementary file 15 — Source data Fig. 3 [file 44321_2025_219_MOESM15_ESM.zip › Figure 3/3A/SP078 0_1 ara 60 min 0 sac040_RGB_Brightfield.tif]

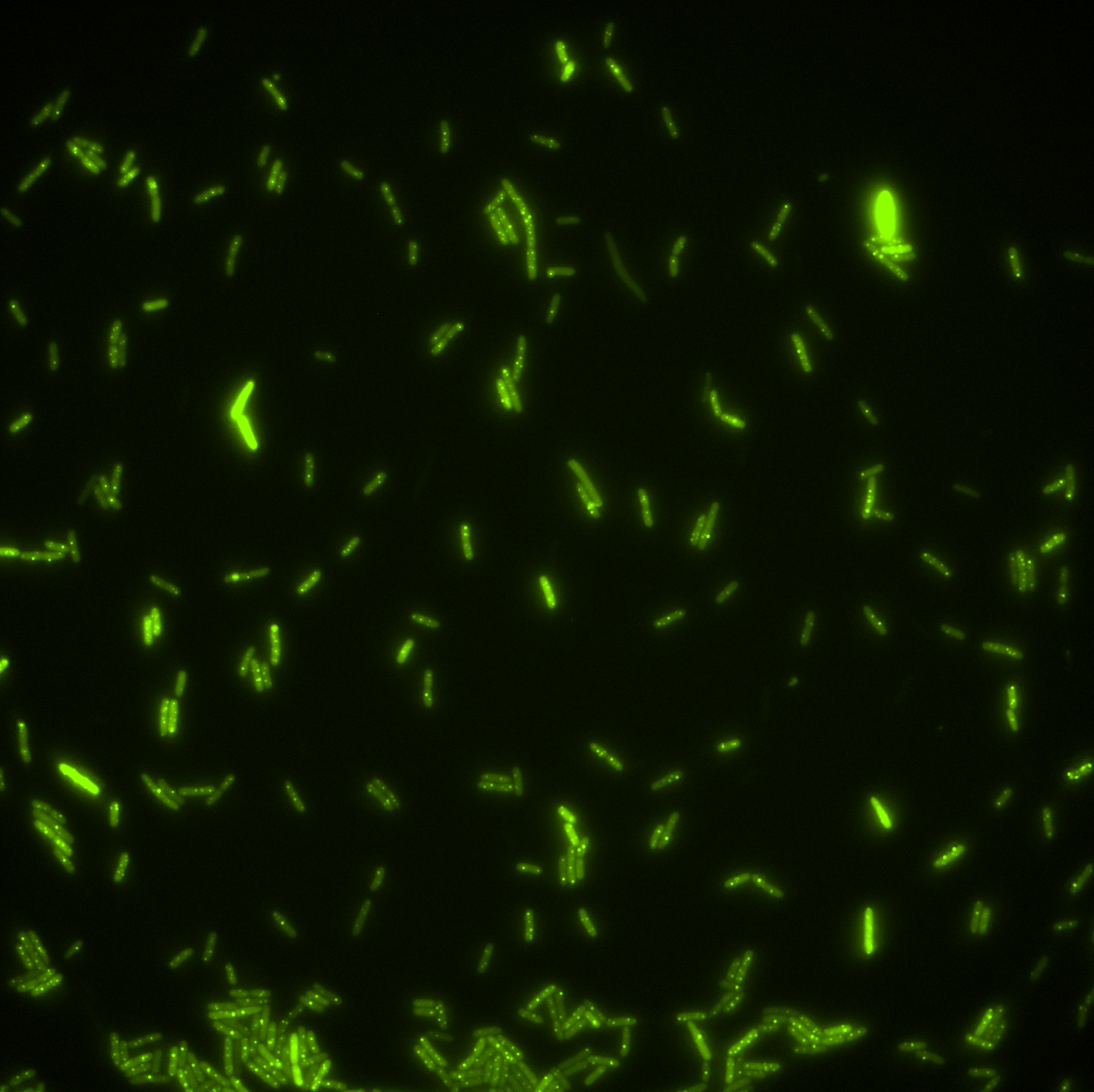

Supplement: Supplementary file 15 — Source data Fig. 3 [file 44321_2025_219_MOESM15_ESM.zip › Figure 3/3A/SP078 0_1 ara 60 min 0 sac040_RGB_eYFP.tif]

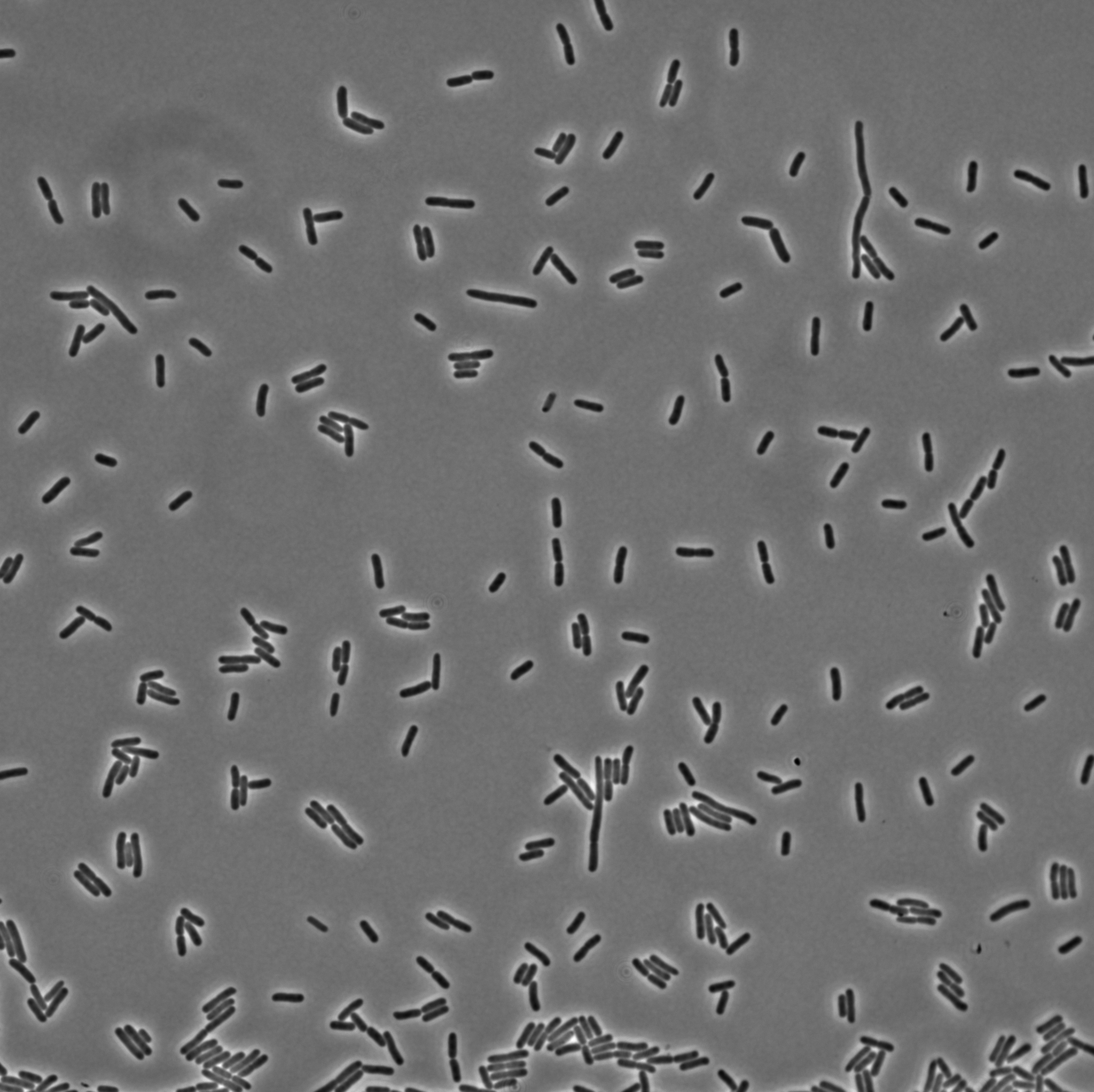

Supplement: Supplementary file 15 — Source data Fig. 3 [file 44321_2025_219_MOESM15_ESM.zip › Figure 3/3A/SP078 0_1 ara 60 min 0 sac041_RGB_Brightfield.tif]

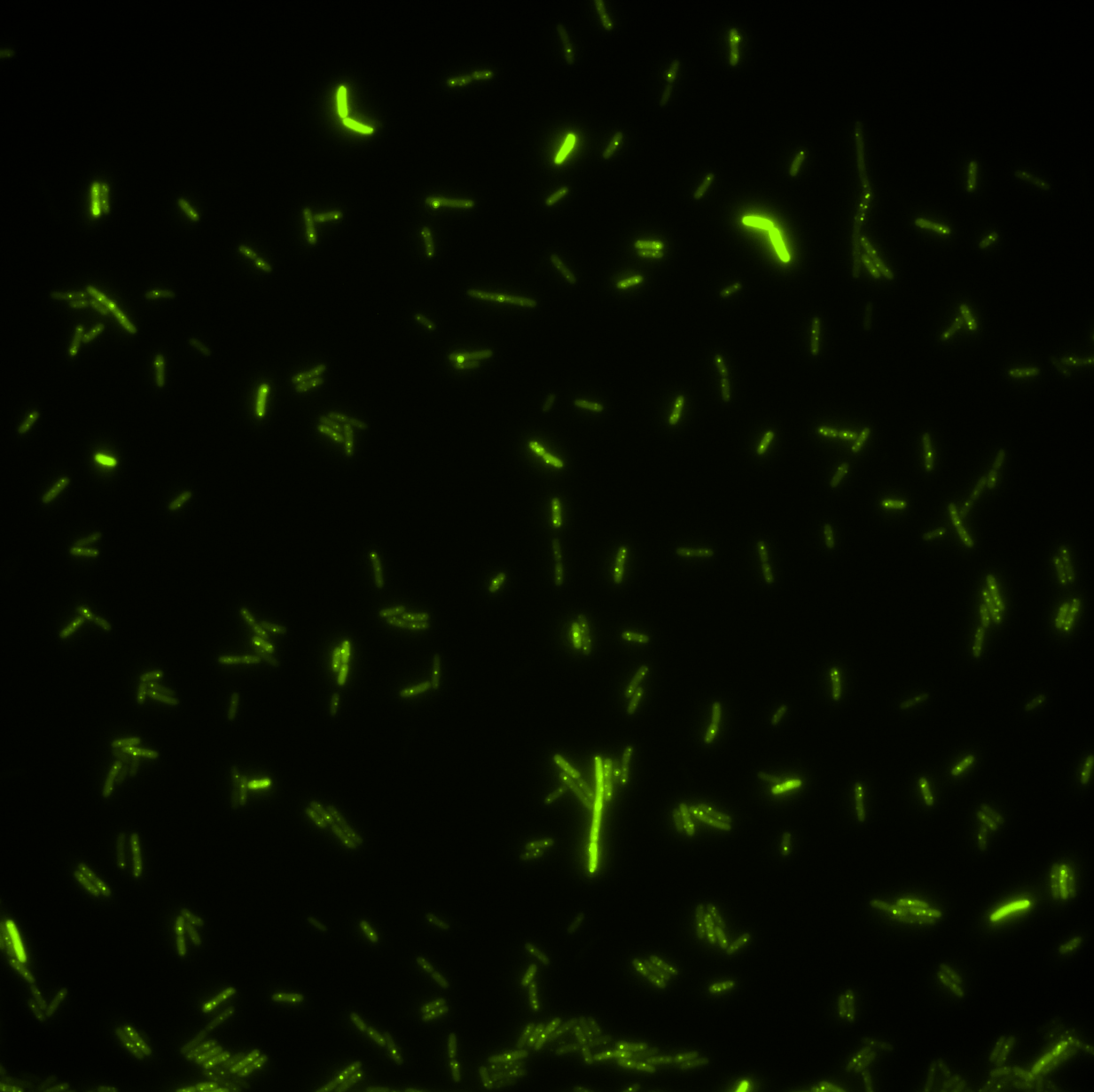

Supplement: Supplementary file 15 — Source data Fig. 3 [file 44321_2025_219_MOESM15_ESM.zip › Figure 3/3A/SP078 0_1 ara 60 min 0 sac041_RGB_eYFP.tif]

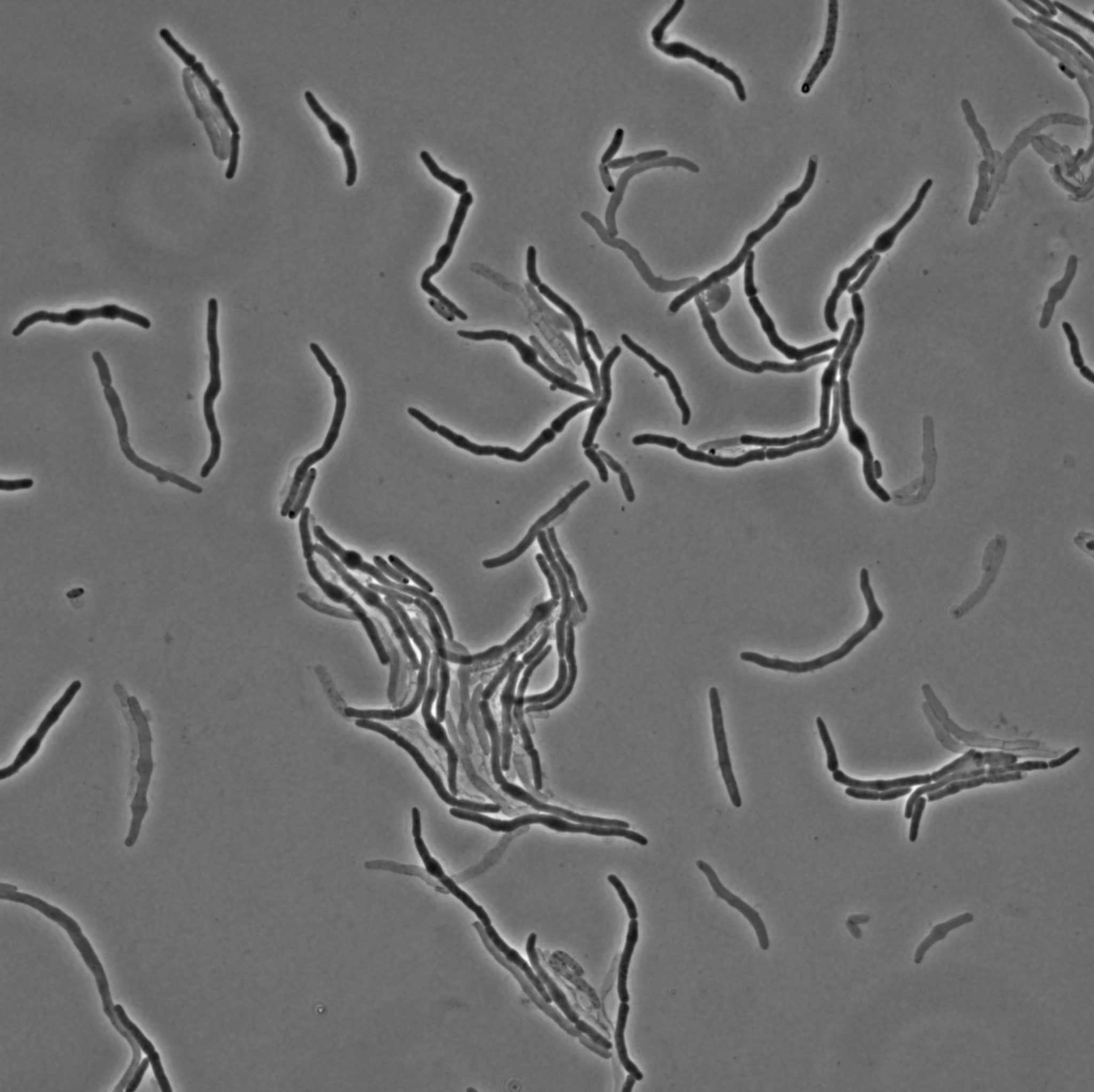

Supplement: Supplementary file 15 — Source data Fig. 3 [file 44321_2025_219_MOESM15_ESM.zip › Figure 3/3A/SP078 0_1 ara 60 min 1_4 sac042_RGB_Brightfield.tif]

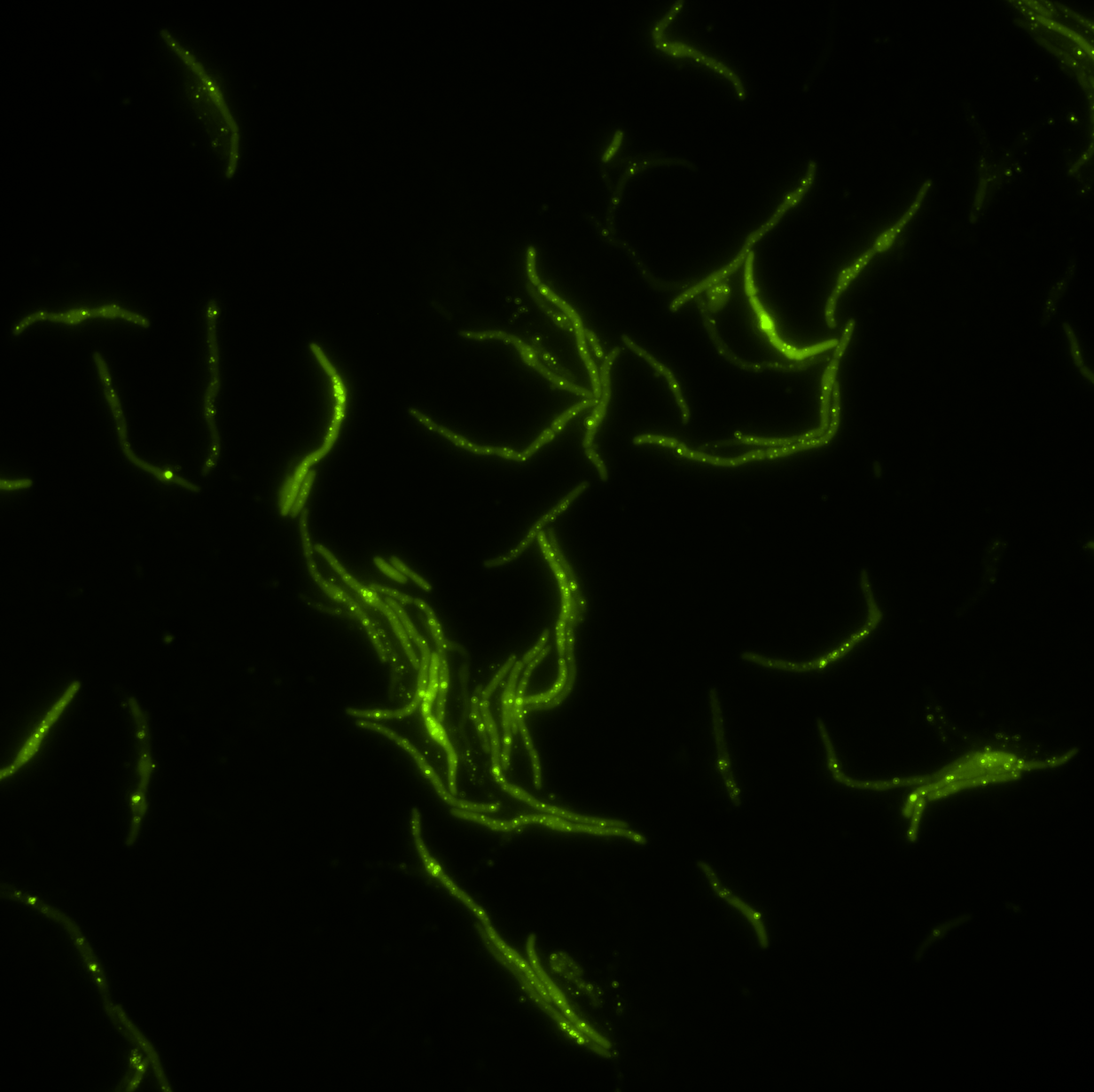

Supplement: Supplementary file 15 — Source data Fig. 3 [file 44321_2025_219_MOESM15_ESM.zip › Figure 3/3A/SP078 0_1 ara 60 min 1_4 sac042_RGB_eYFP.tif]

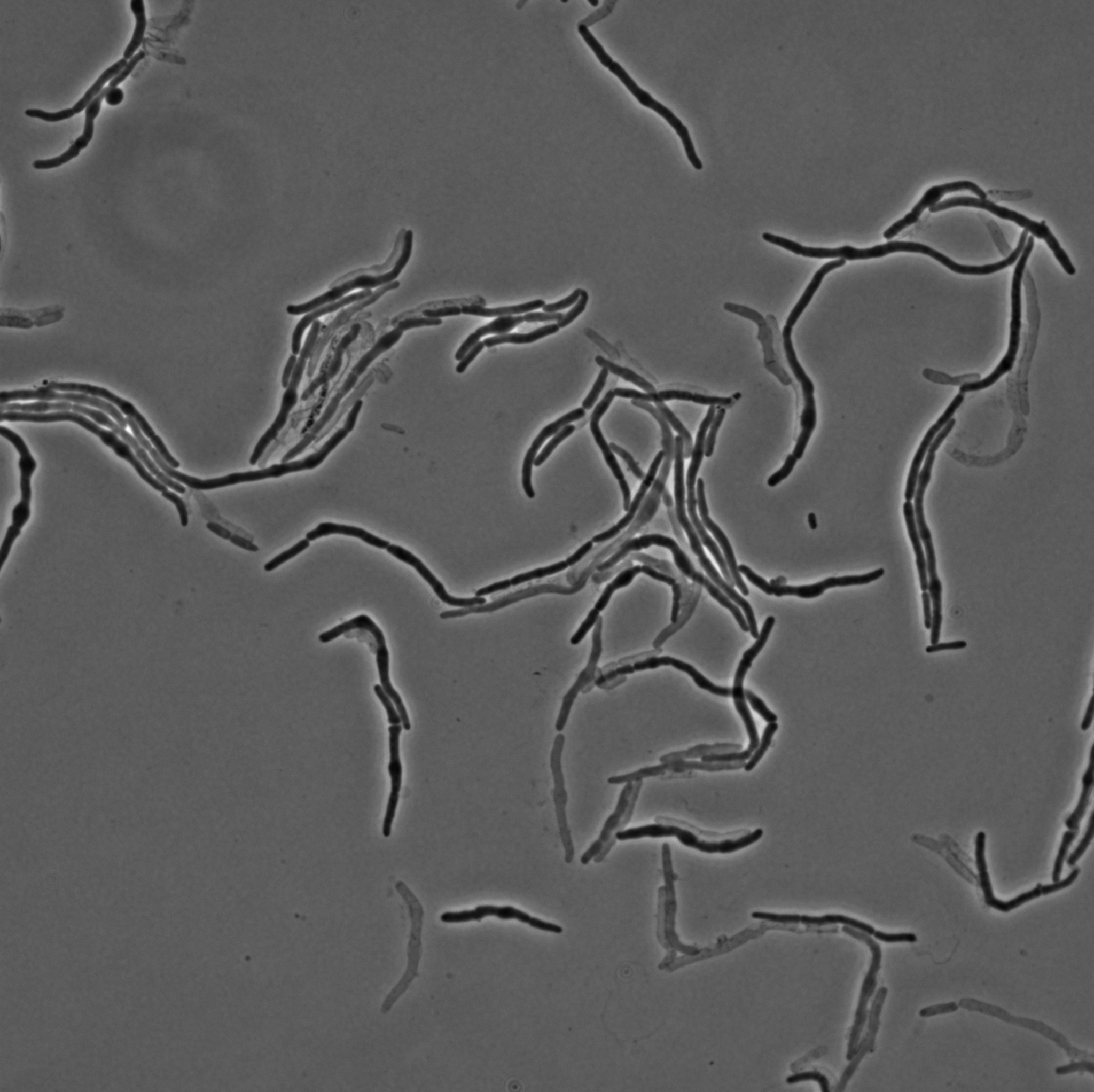

Supplement: Supplementary file 15 — Source data Fig. 3 [file 44321_2025_219_MOESM15_ESM.zip › Figure 3/3A/SP078 0_1 ara 60 min 1_4 sac043_RGB_Brightfield.tif]

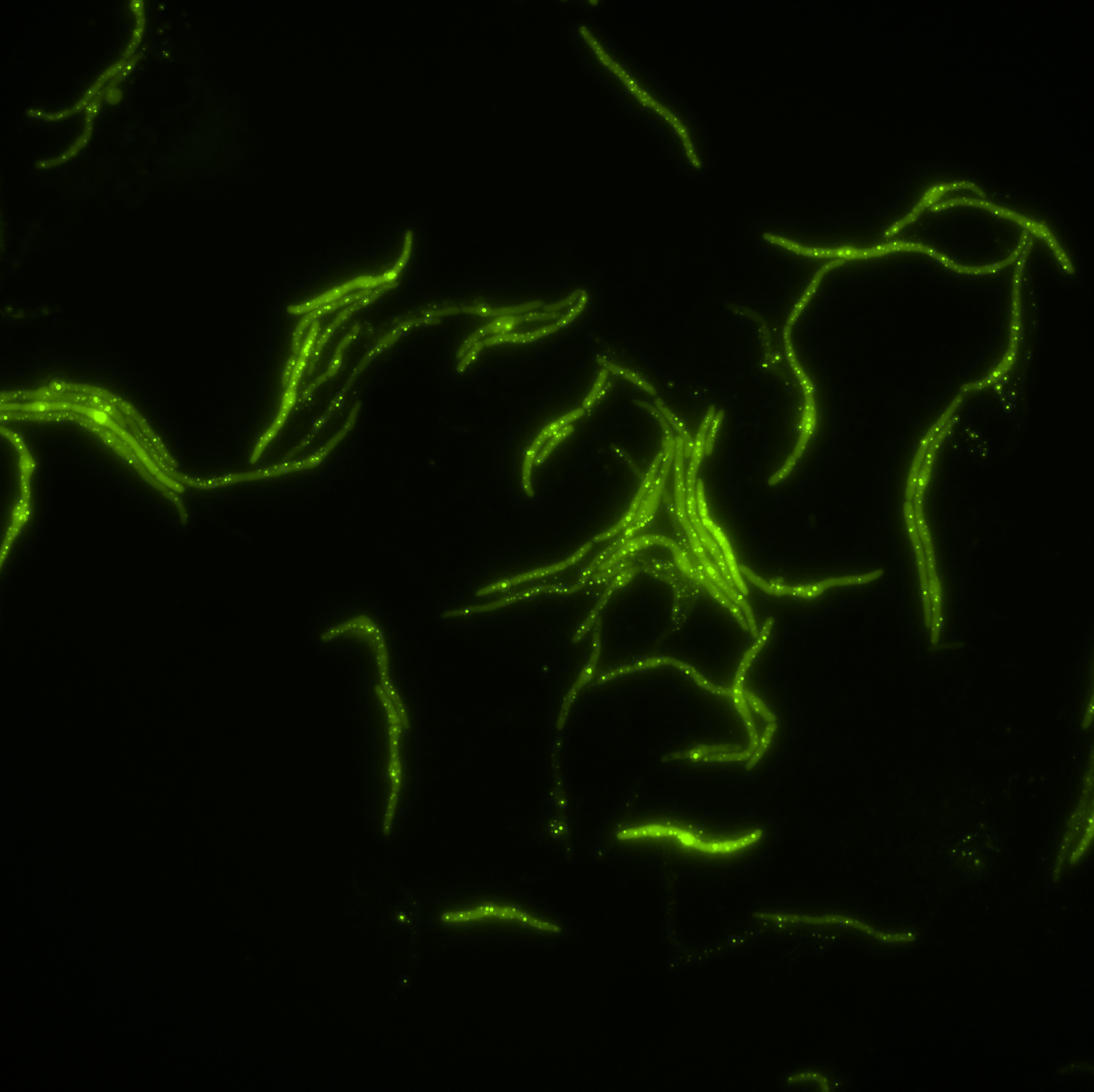

Supplement: Supplementary file 15 — Source data Fig. 3 [file 44321_2025_219_MOESM15_ESM.zip › Figure 3/3A/SP078 0_1 ara 60 min 1_4 sac043_RGB_eYFP.tif]

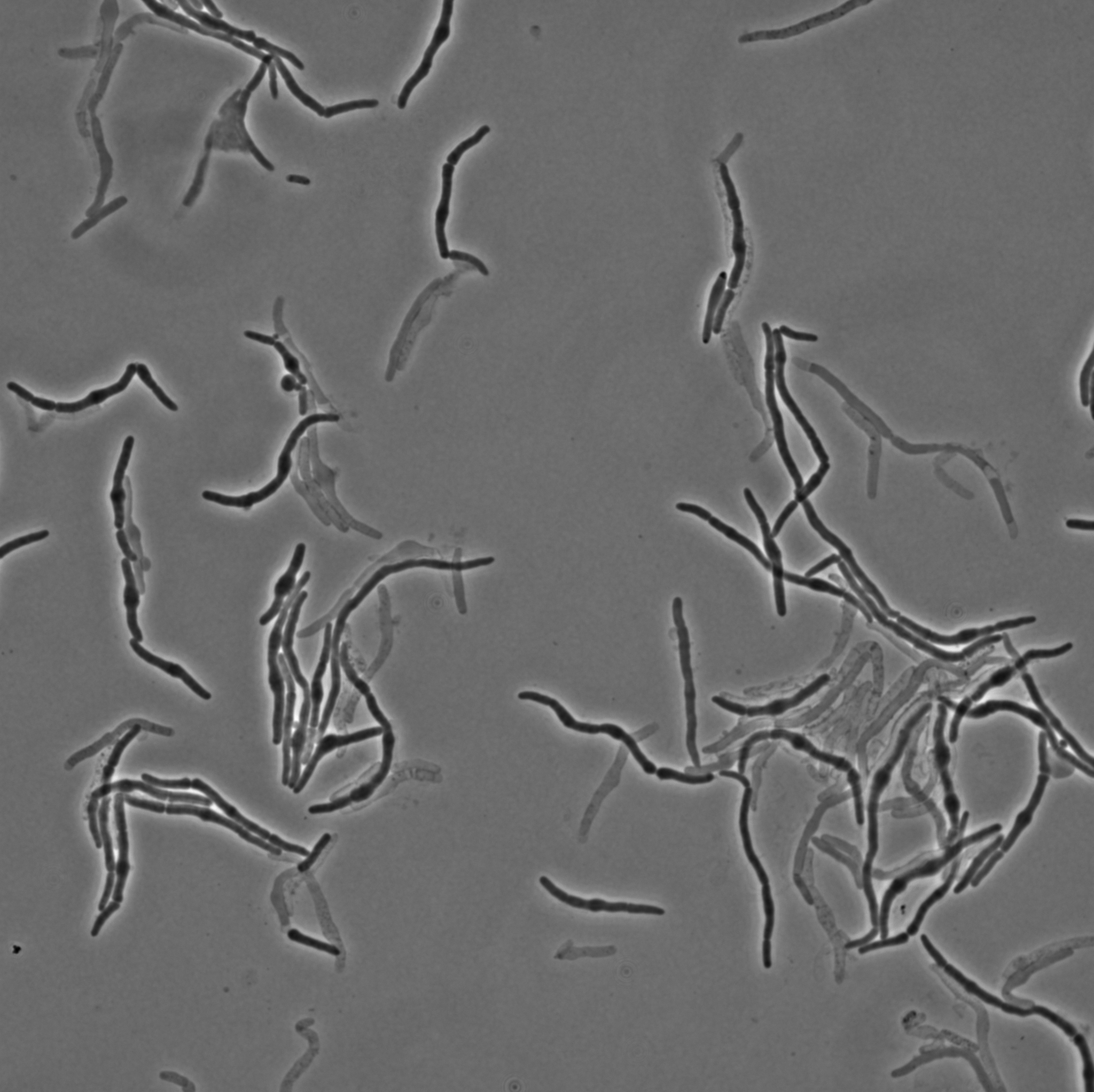

Supplement: Supplementary file 15 — Source data Fig. 3 [file 44321_2025_219_MOESM15_ESM.zip › Figure 3/3A/SP078 0_1 ara 60 min 1_4 sac044_RGB_Brightfield.tif]

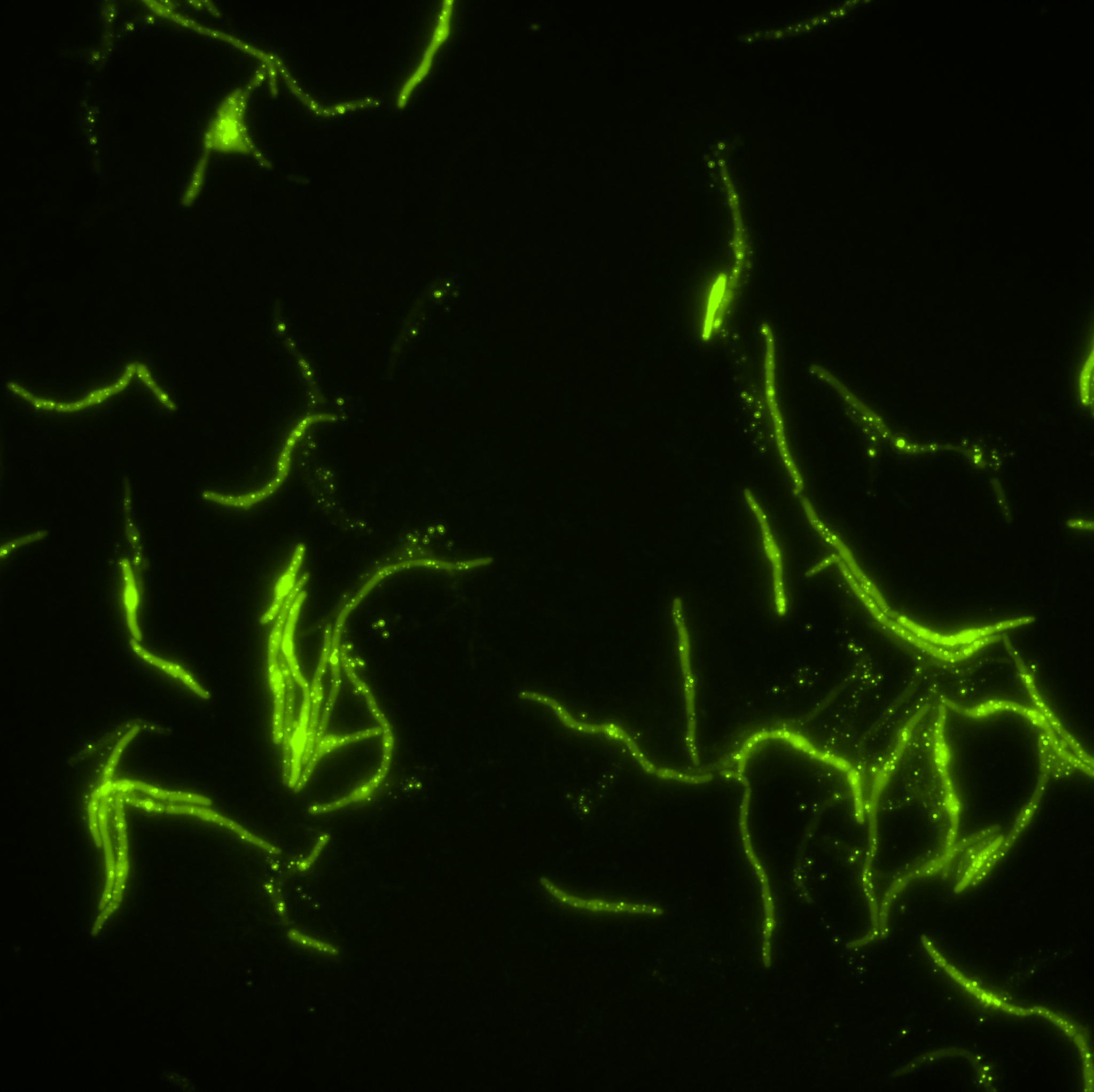

Supplement: Supplementary file 15 — Source data Fig. 3 [file 44321_2025_219_MOESM15_ESM.zip › Figure 3/3A/SP078 0_1 ara 60 min 1_4 sac044_RGB_eYFP.tif]

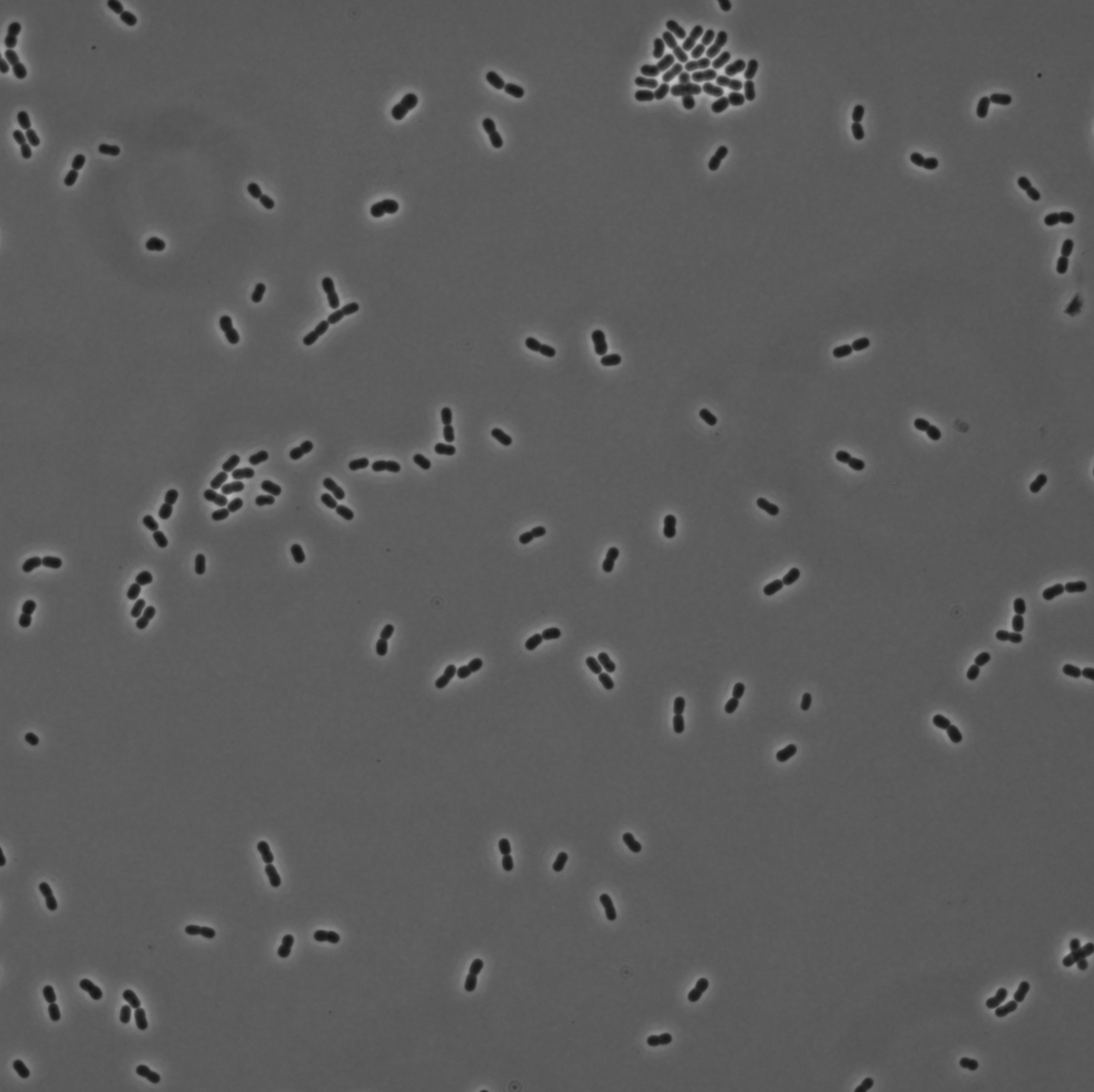

Supplement: Supplementary file 18 — Source data Fig. 6 [file 44321_2025_219_MOESM18_ESM.zip › Figure 6/6A/ab5075 mu 0 percent saccarinet01.tif]

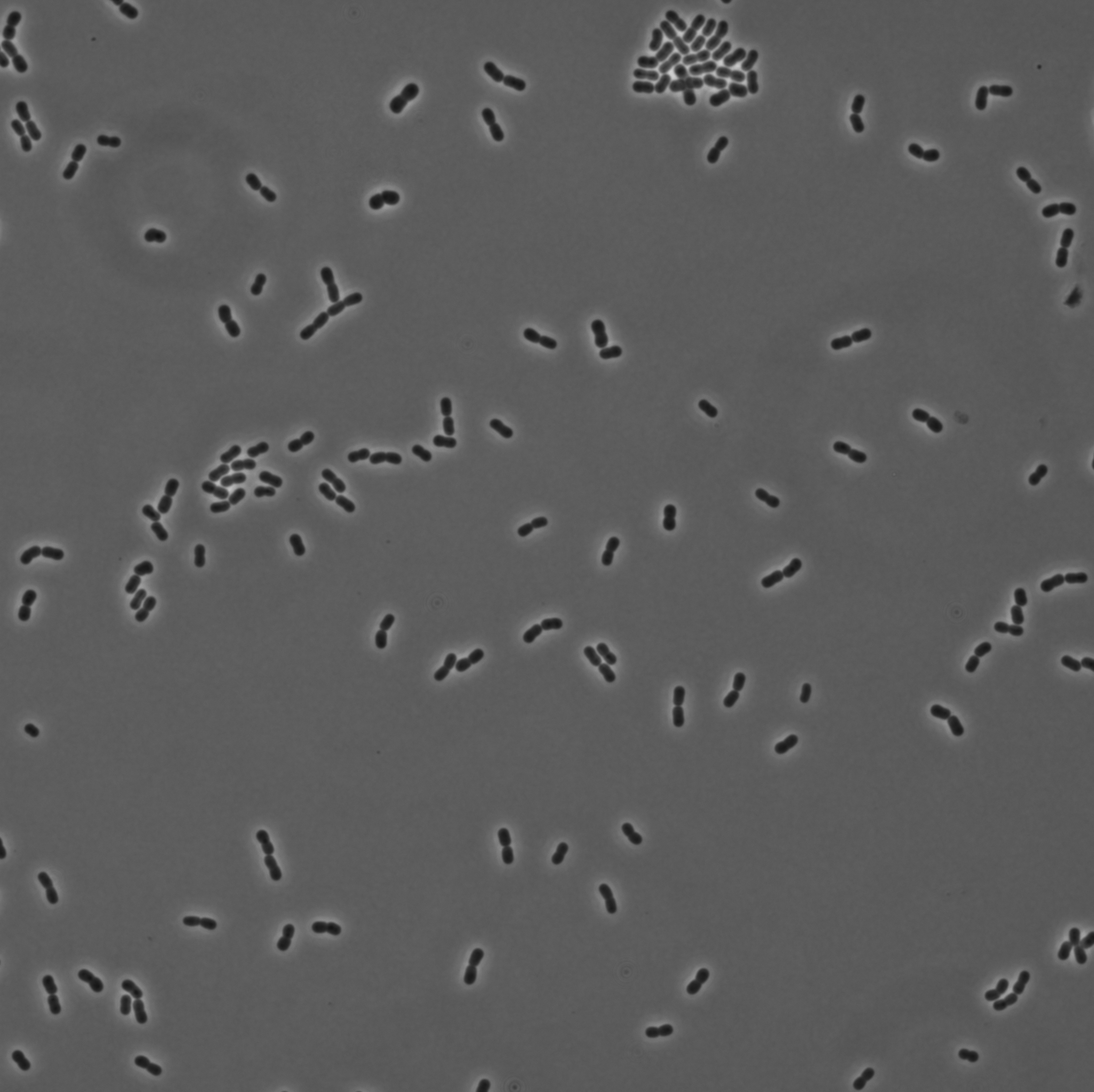

Supplement: Supplementary file 18 — Source data Fig. 6 [file 44321_2025_219_MOESM18_ESM.zip › Figure 6/6A/ab5075 mu 0 percent saccarinet02.tif]

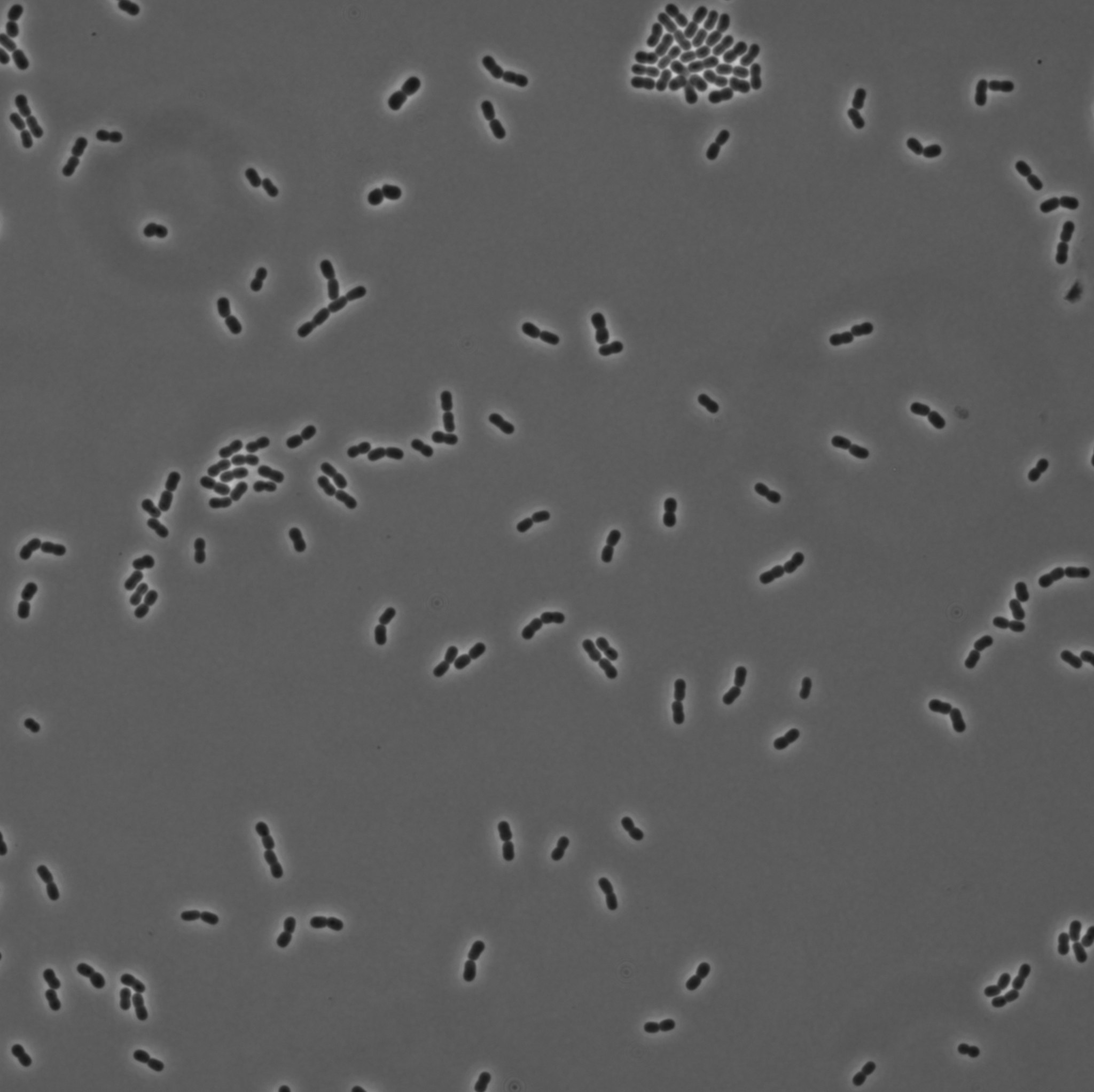

Supplement: Supplementary file 18 — Source data Fig. 6 [file 44321_2025_219_MOESM18_ESM.zip › Figure 6/6A/ab5075 mu 0 percent saccarinet03.tif]

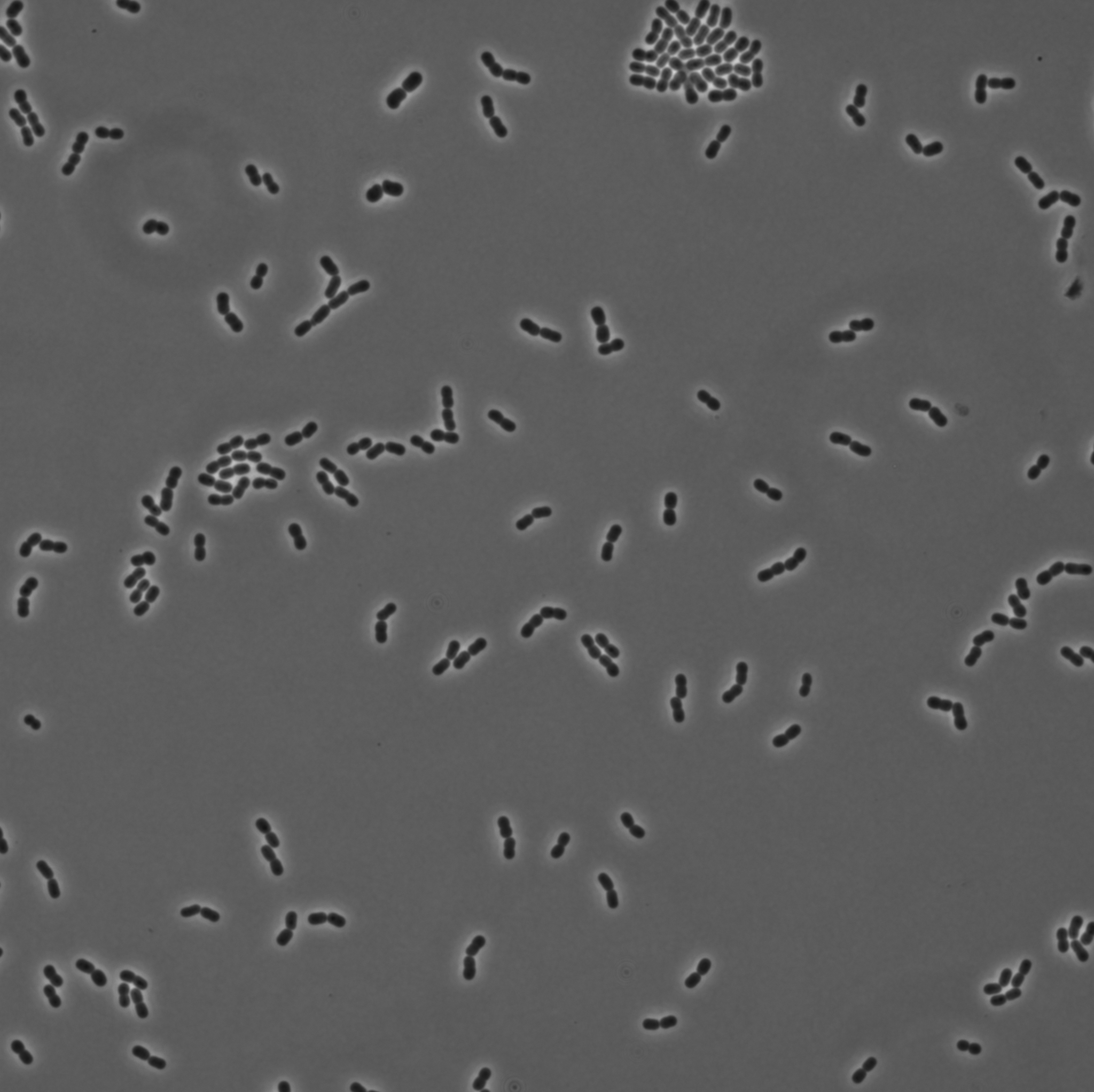

Supplement: Supplementary file 18 — Source data Fig. 6 [file 44321_2025_219_MOESM18_ESM.zip › Figure 6/6A/ab5075 mu 0 percent saccarinet04.tif]

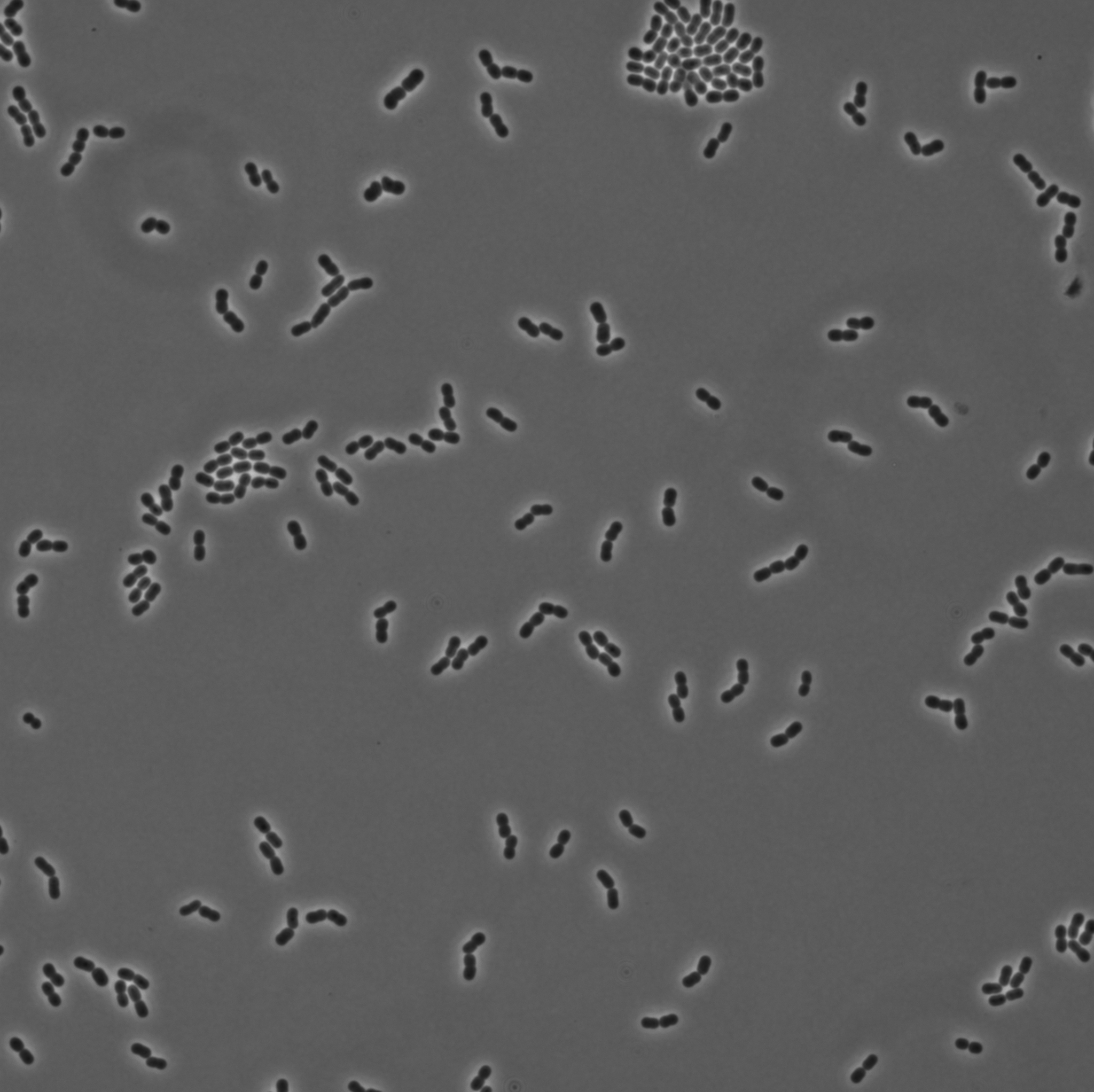

Supplement: Supplementary file 18 — Source data Fig. 6 [file 44321_2025_219_MOESM18_ESM.zip › Figure 6/6A/ab5075 mu 0 percent saccarinet05.tif]

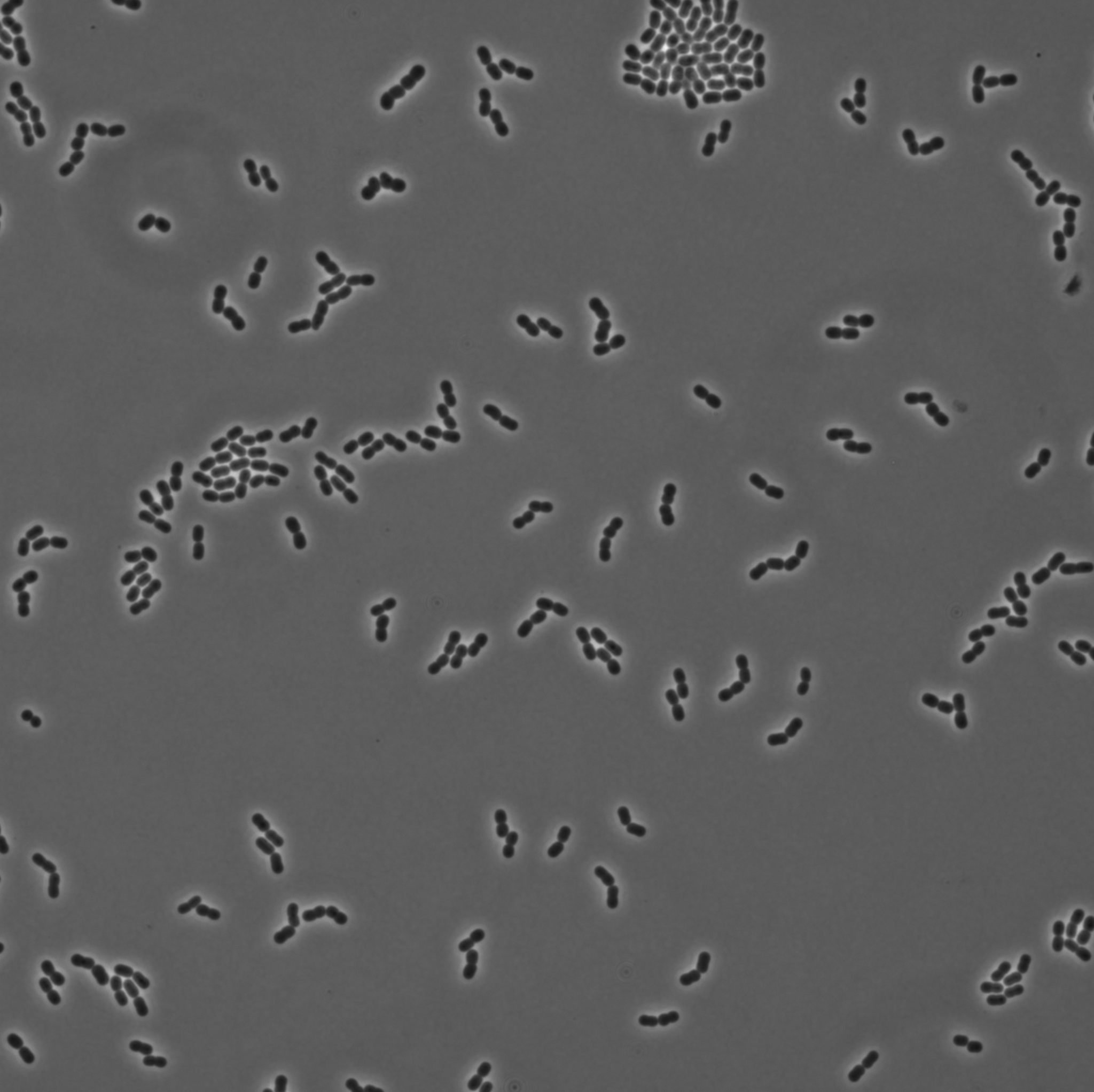

Supplement: Supplementary file 18 — Source data Fig. 6 [file 44321_2025_219_MOESM18_ESM.zip › Figure 6/6A/ab5075 mu 0 percent saccarinet06.tif]

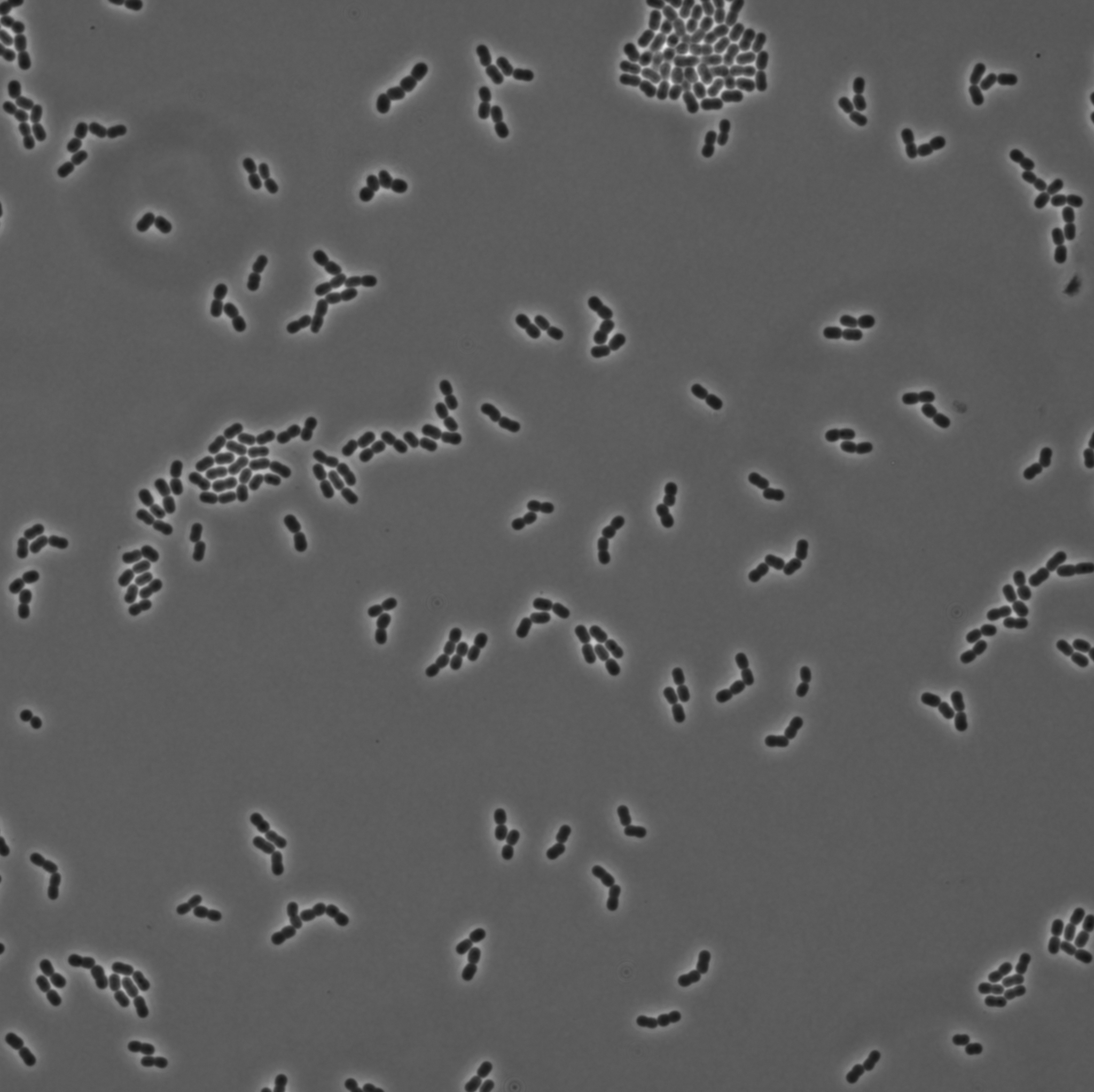

Supplement: Supplementary file 18 — Source data Fig. 6 [file 44321_2025_219_MOESM18_ESM.zip › Figure 6/6A/ab5075 mu 0 percent saccarinet07.tif]

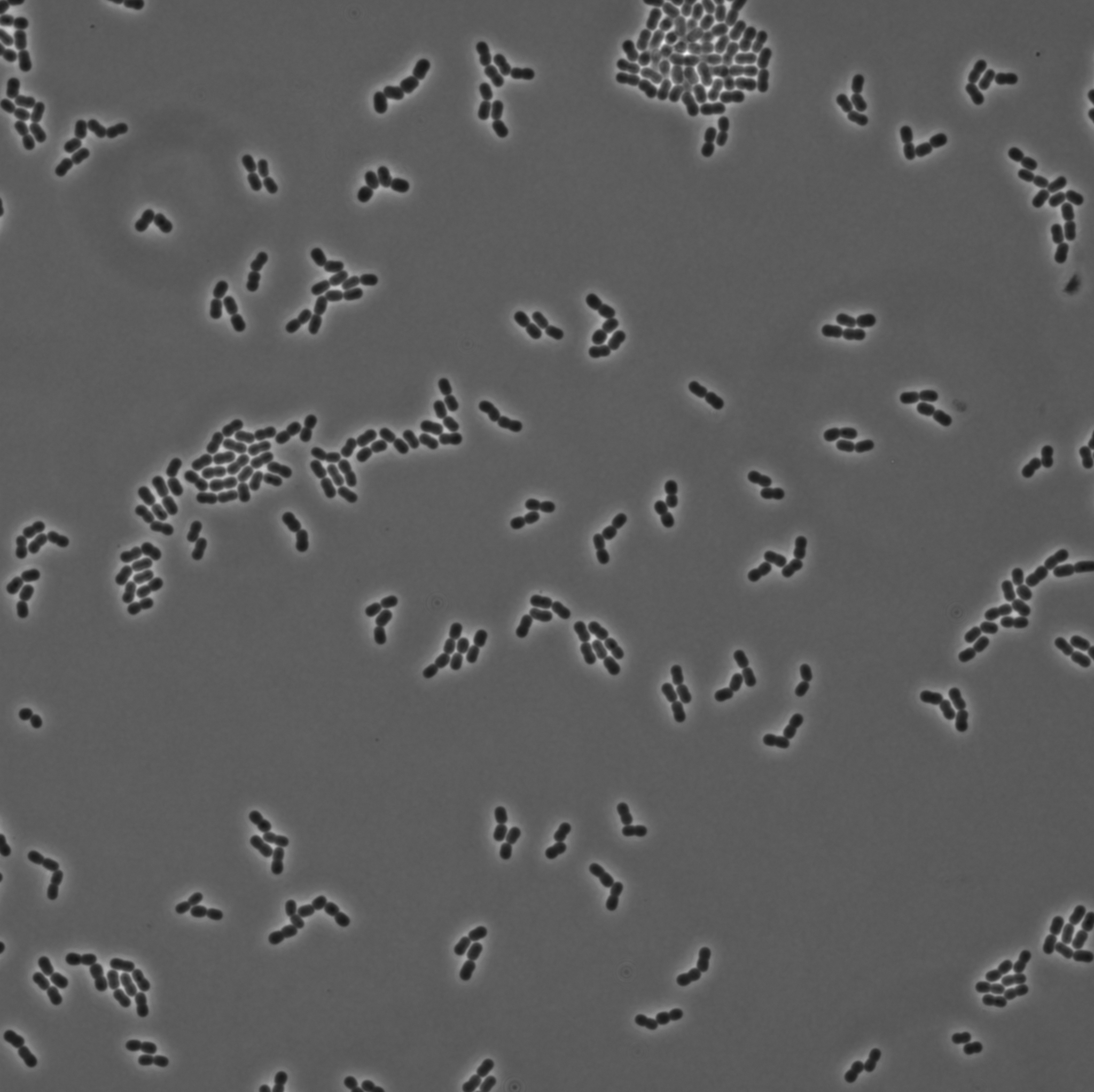

Supplement: Supplementary file 18 — Source data Fig. 6 [file 44321_2025_219_MOESM18_ESM.zip › Figure 6/6A/ab5075 mu 0 percent saccarinet08.tif]

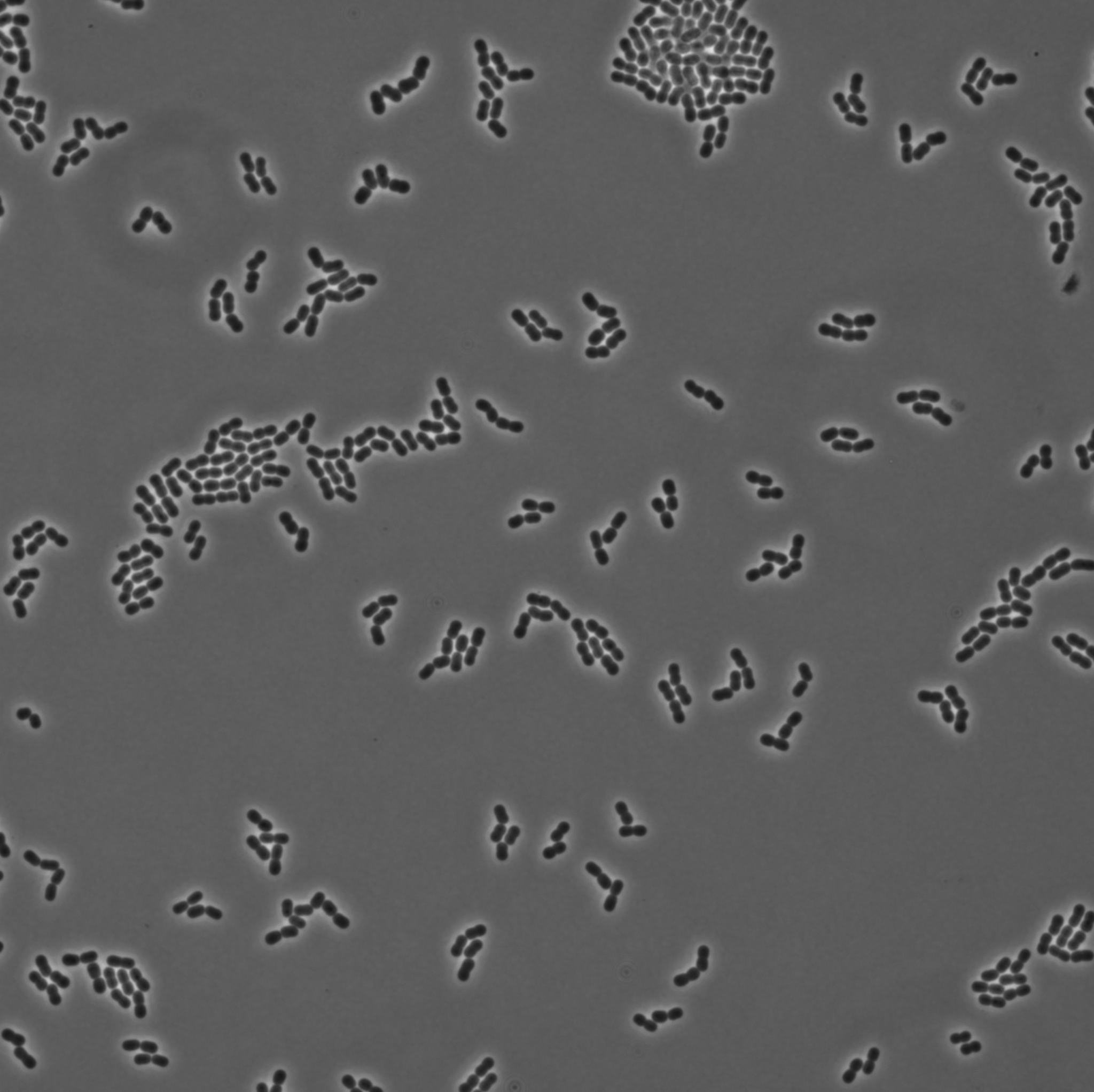

Supplement: Supplementary file 18 — Source data Fig. 6 [file 44321_2025_219_MOESM18_ESM.zip › Figure 6/6A/ab5075 mu 0 percent saccarinet09.tif]

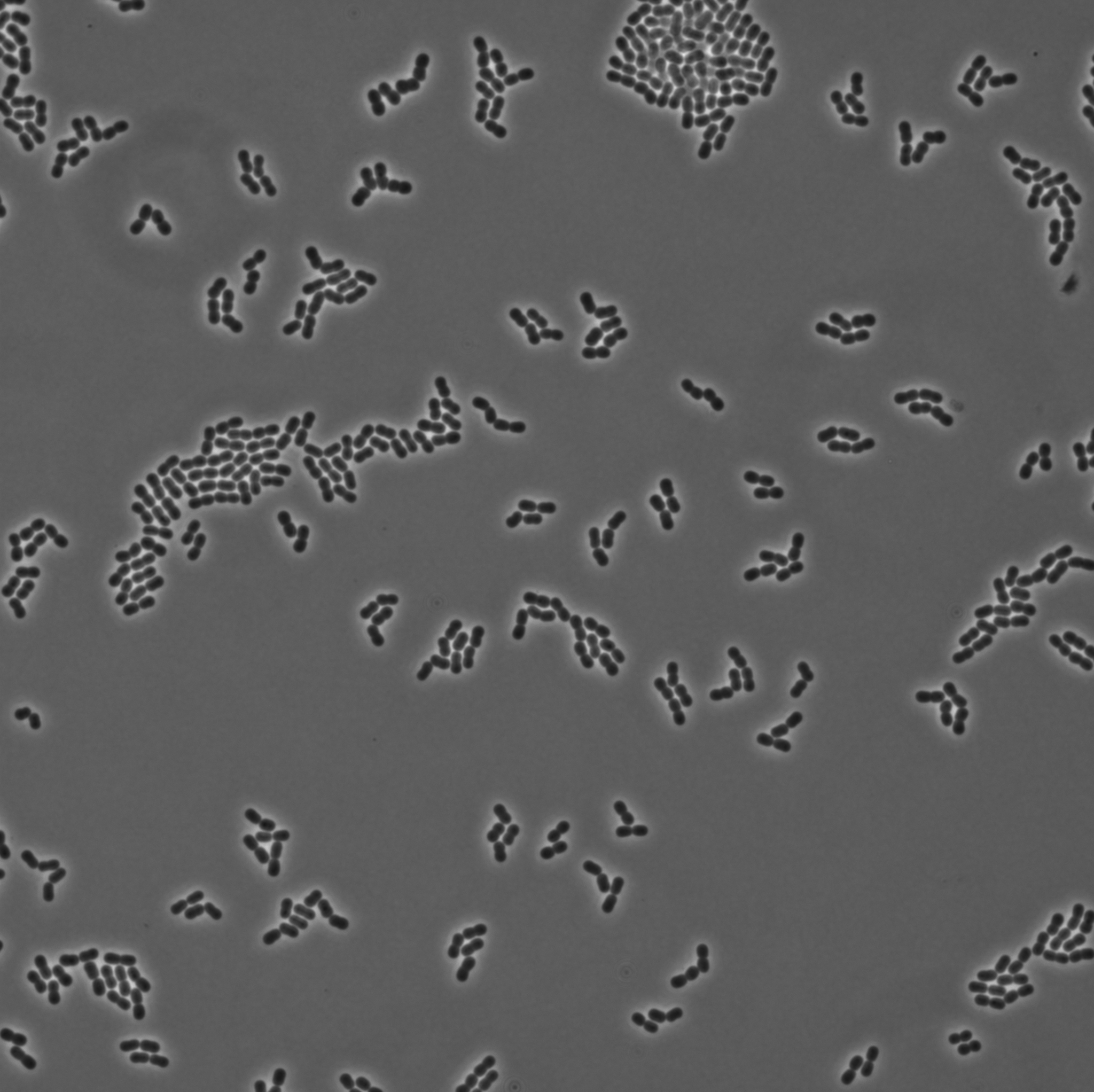

Supplement: Supplementary file 18 — Source data Fig. 6 [file 44321_2025_219_MOESM18_ESM.zip › Figure 6/6A/ab5075 mu 0 percent saccarinet10.tif]

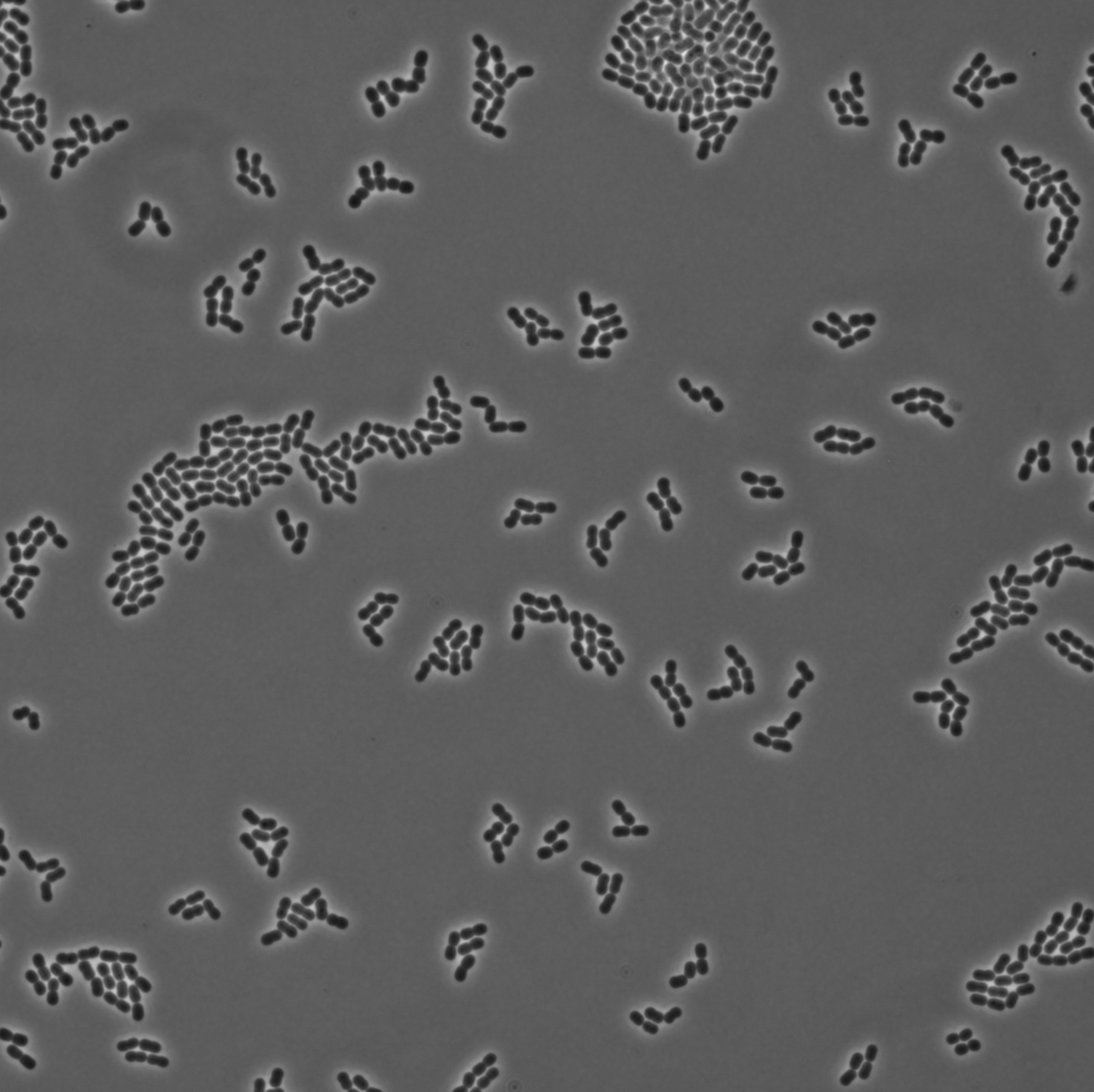

Supplement: Supplementary file 18 — Source data Fig. 6 [file 44321_2025_219_MOESM18_ESM.zip › Figure 6/6A/ab5075 mu 0 percent saccarinet11.tif]

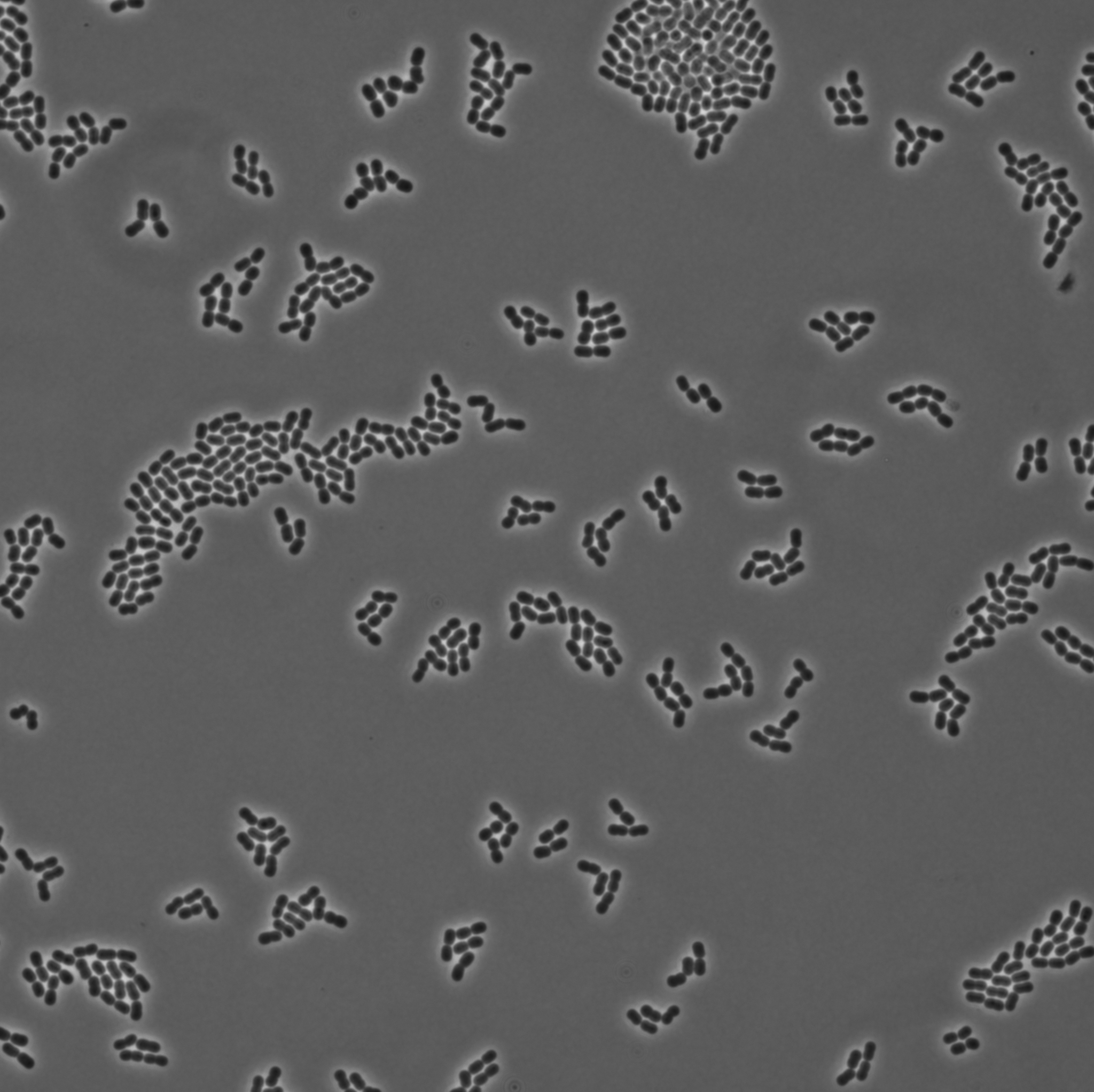

Supplement: Supplementary file 18 — Source data Fig. 6 [file 44321_2025_219_MOESM18_ESM.zip › Figure 6/6A/ab5075 mu 0 percent saccarinet12.tif]

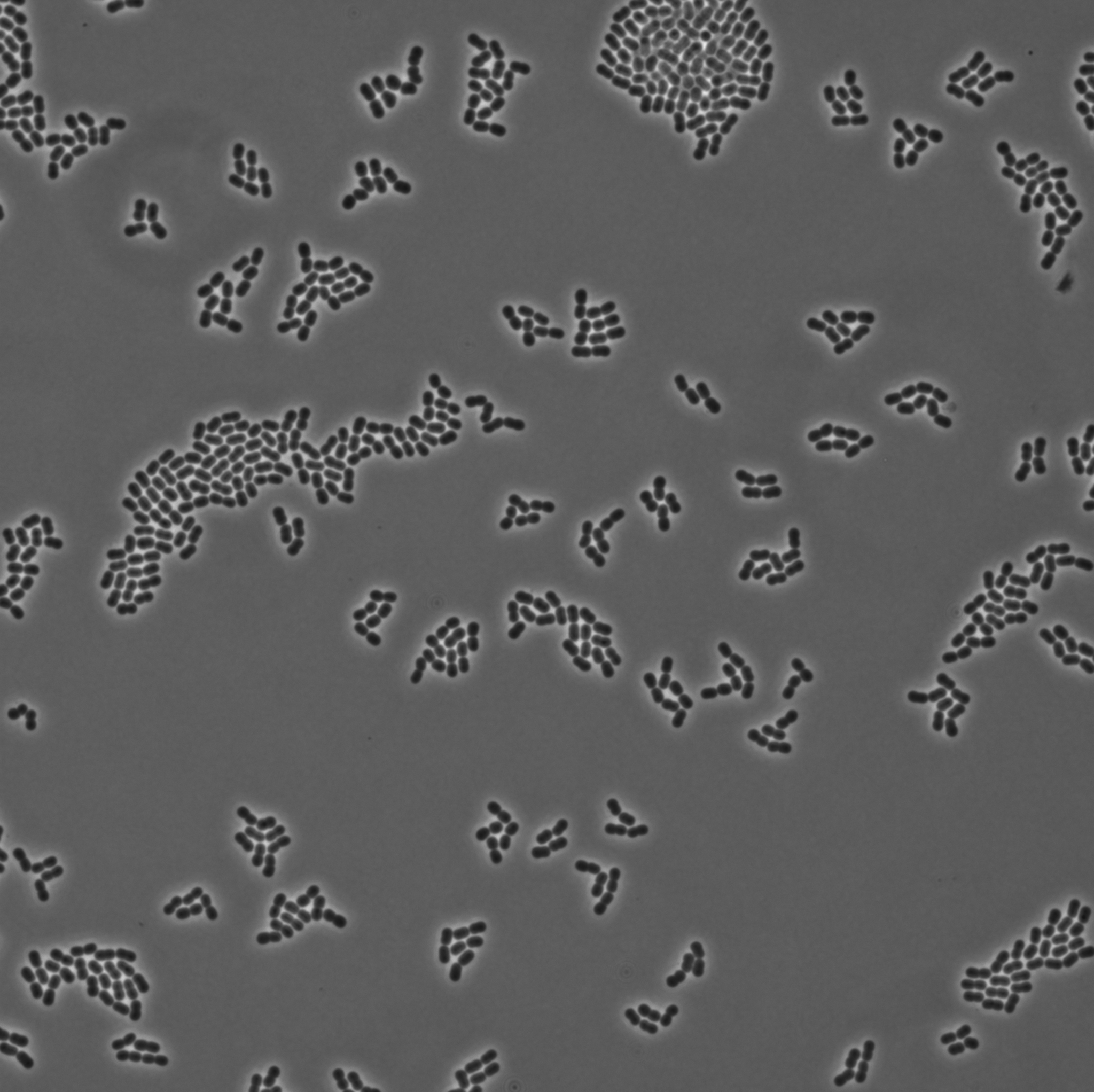

Supplement: Supplementary file 18 — Source data Fig. 6 [file 44321_2025_219_MOESM18_ESM.zip › Figure 6/6A/ab5075 mu 0 percent saccarinet13.tif]

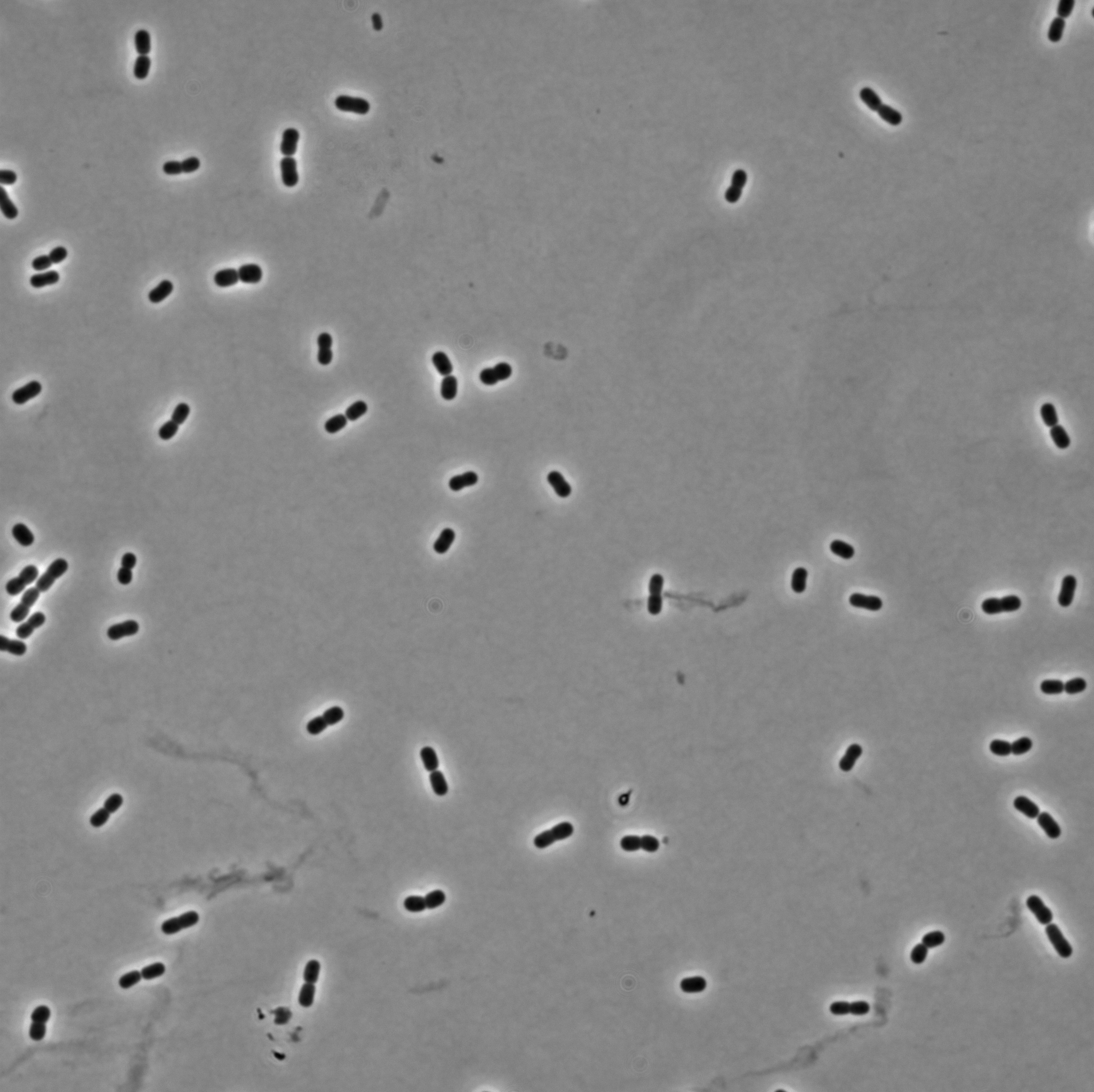

Supplement: Supplementary file 18 — Source data Fig. 6 [file 44321_2025_219_MOESM18_ESM.zip › Figure 6/6A/AB5075 Mu 2 percent saccarine002_RGB_Brightfield.tif]

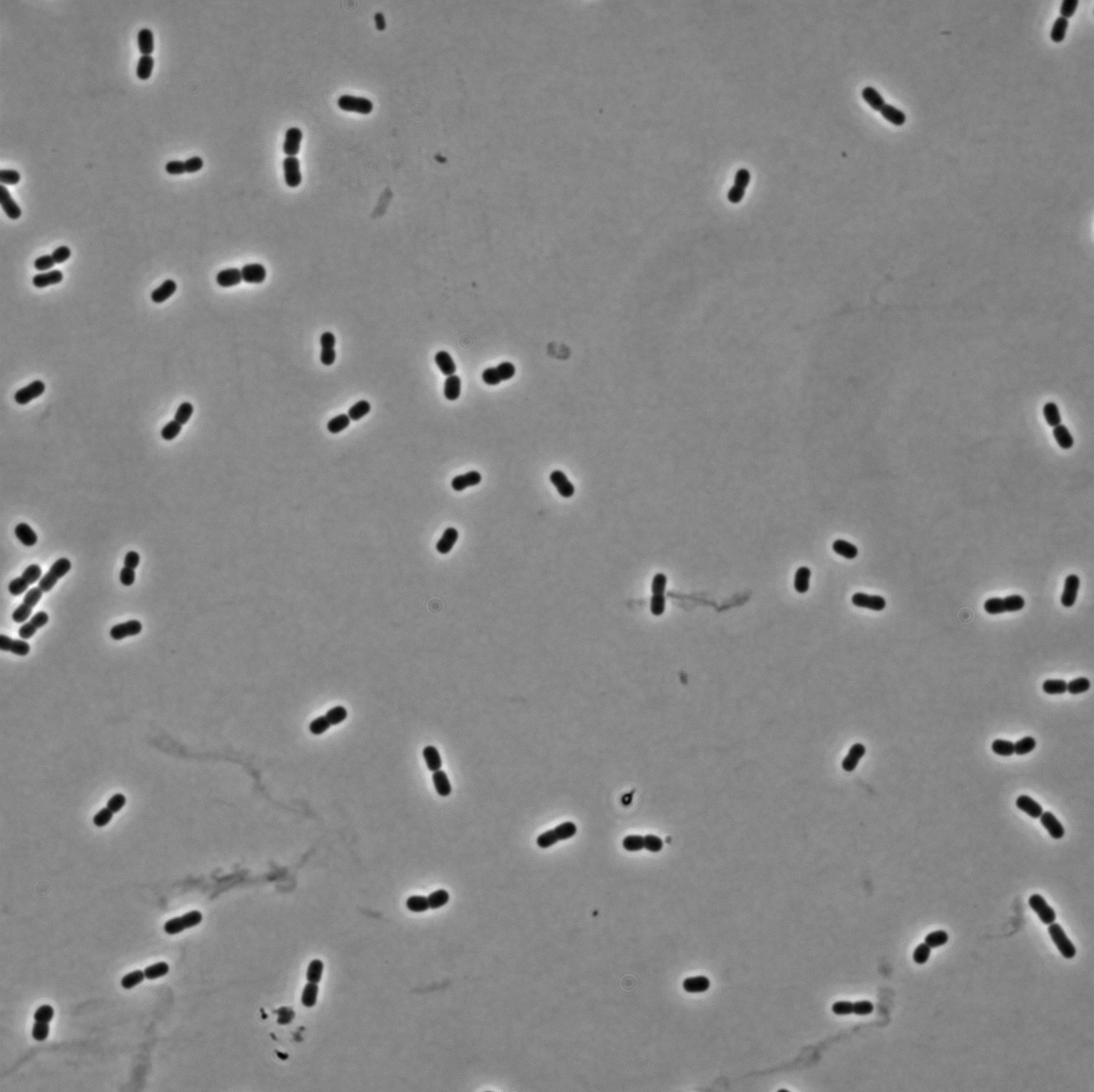

Supplement: Supplementary file 18 — Source data Fig. 6 [file 44321_2025_219_MOESM18_ESM.zip › Figure 6/6A/AB5075 Mu 2 percent saccarine004_RGB_Brightfield.tif]

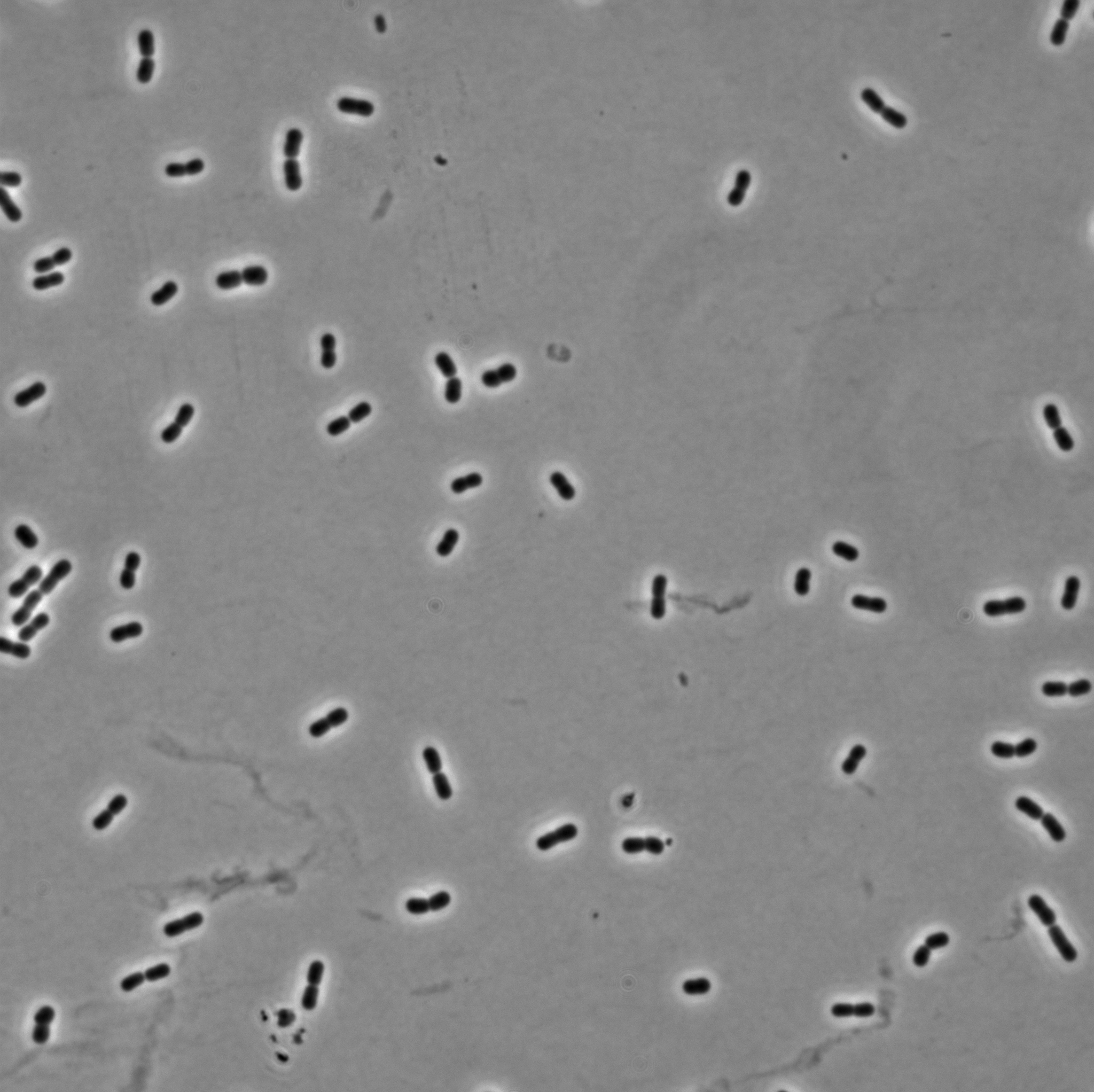

Supplement: Supplementary file 18 — Source data Fig. 6 [file 44321_2025_219_MOESM18_ESM.zip › Figure 6/6A/AB5075 Mu 2 percent saccarine006_RGB_Brightfield.tif]

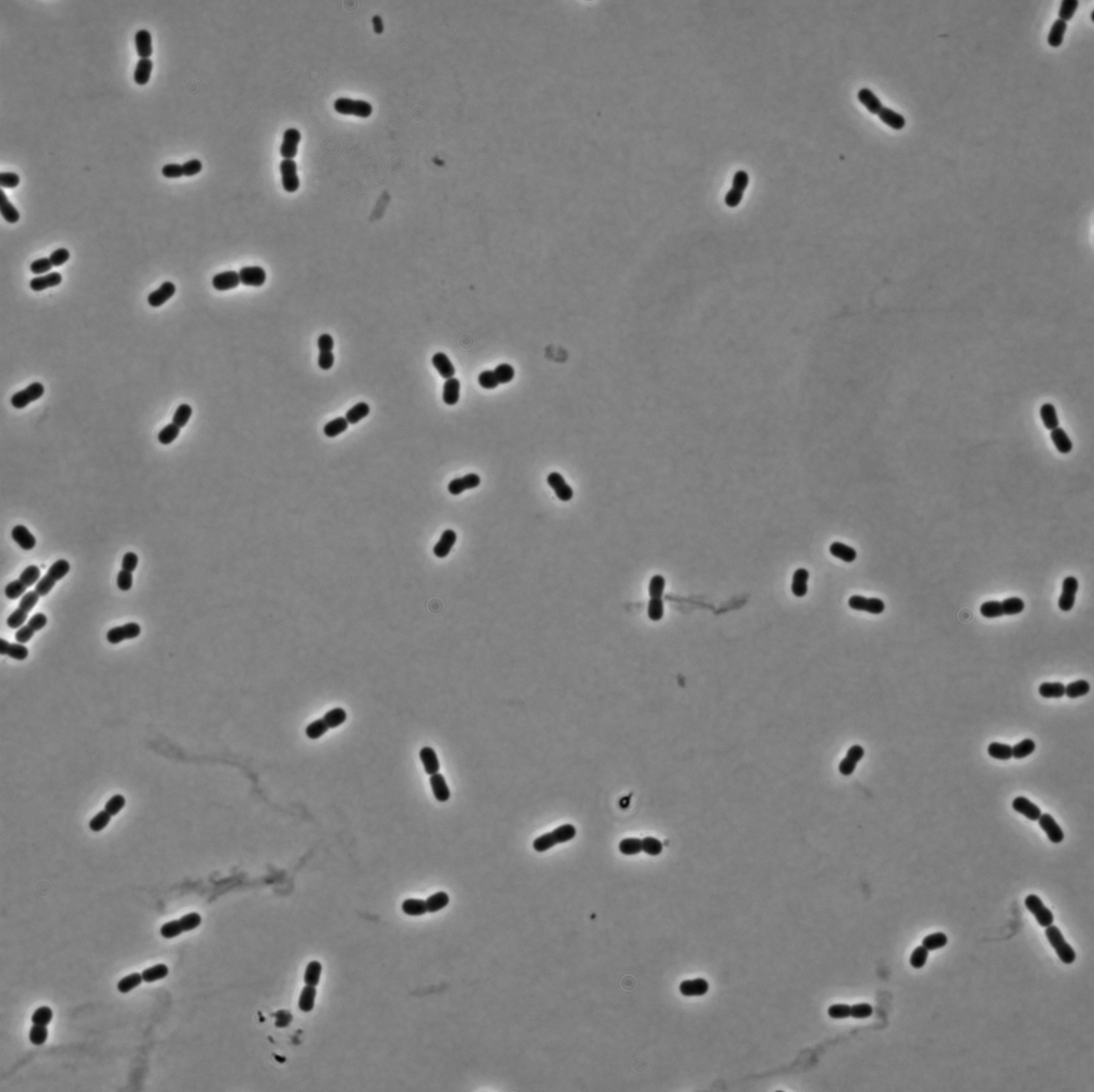

Supplement: Supplementary file 18 — Source data Fig. 6 [file 44321_2025_219_MOESM18_ESM.zip › Figure 6/6A/AB5075 Mu 2 percent saccarine008_RGB_Brightfield.tif]

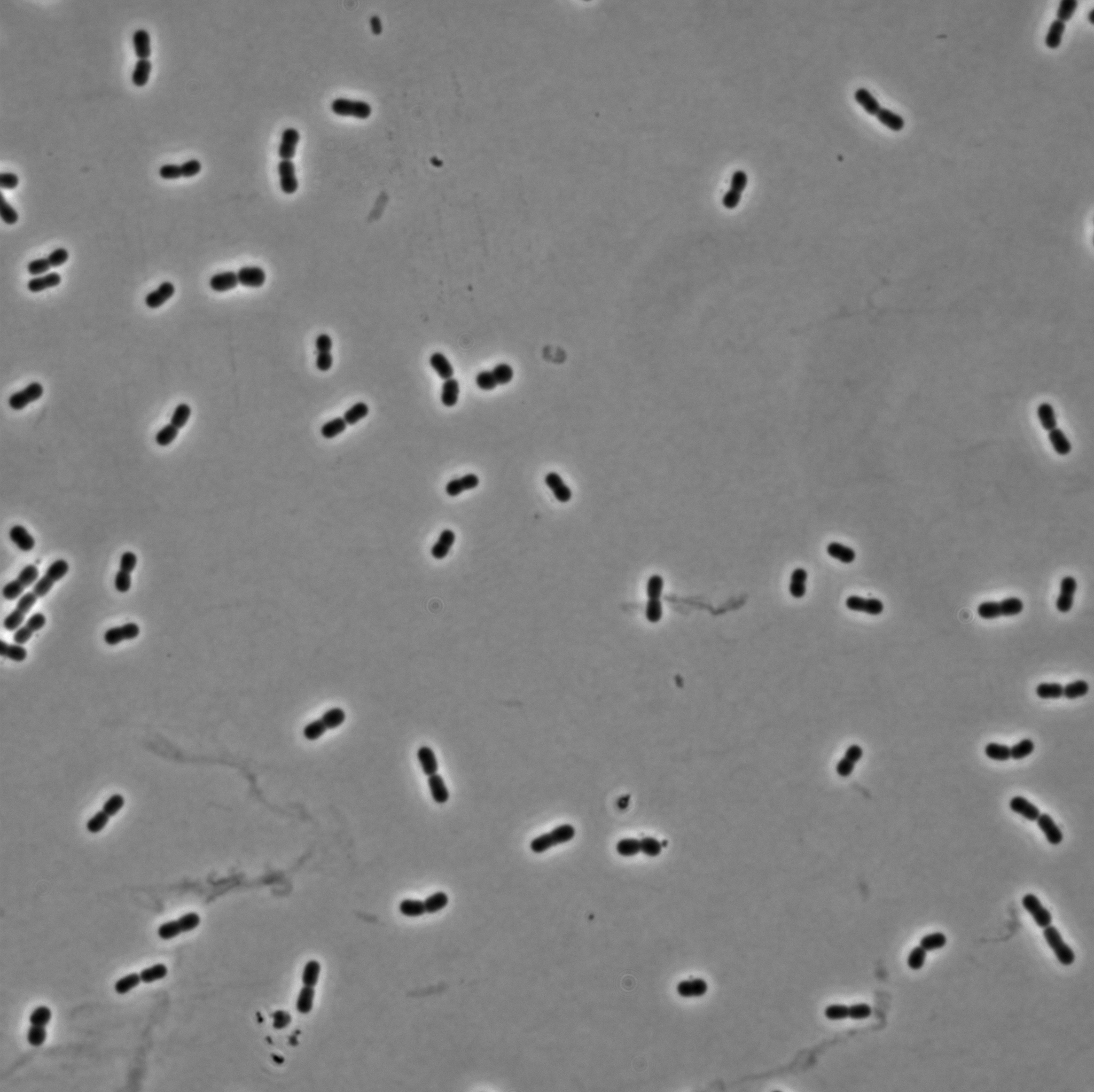

Supplement: Supplementary file 18 — Source data Fig. 6 [file 44321_2025_219_MOESM18_ESM.zip › Figure 6/6A/AB5075 Mu 2 percent saccarine010_RGB_Brightfield.tif]

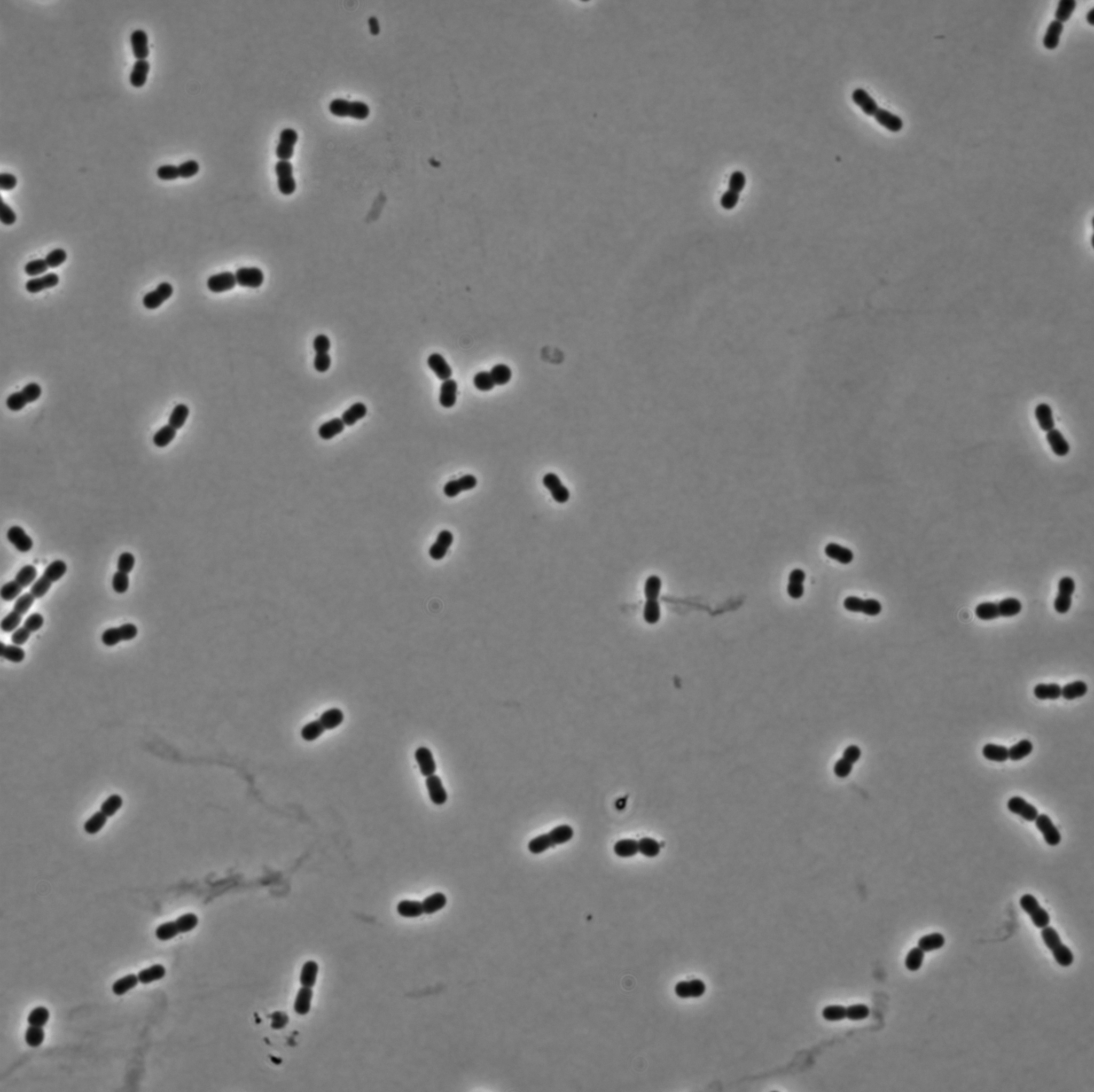

Supplement: Supplementary file 18 — Source data Fig. 6 [file 44321_2025_219_MOESM18_ESM.zip › Figure 6/6A/AB5075 Mu 2 percent saccarine012_RGB_Brightfield.tif]

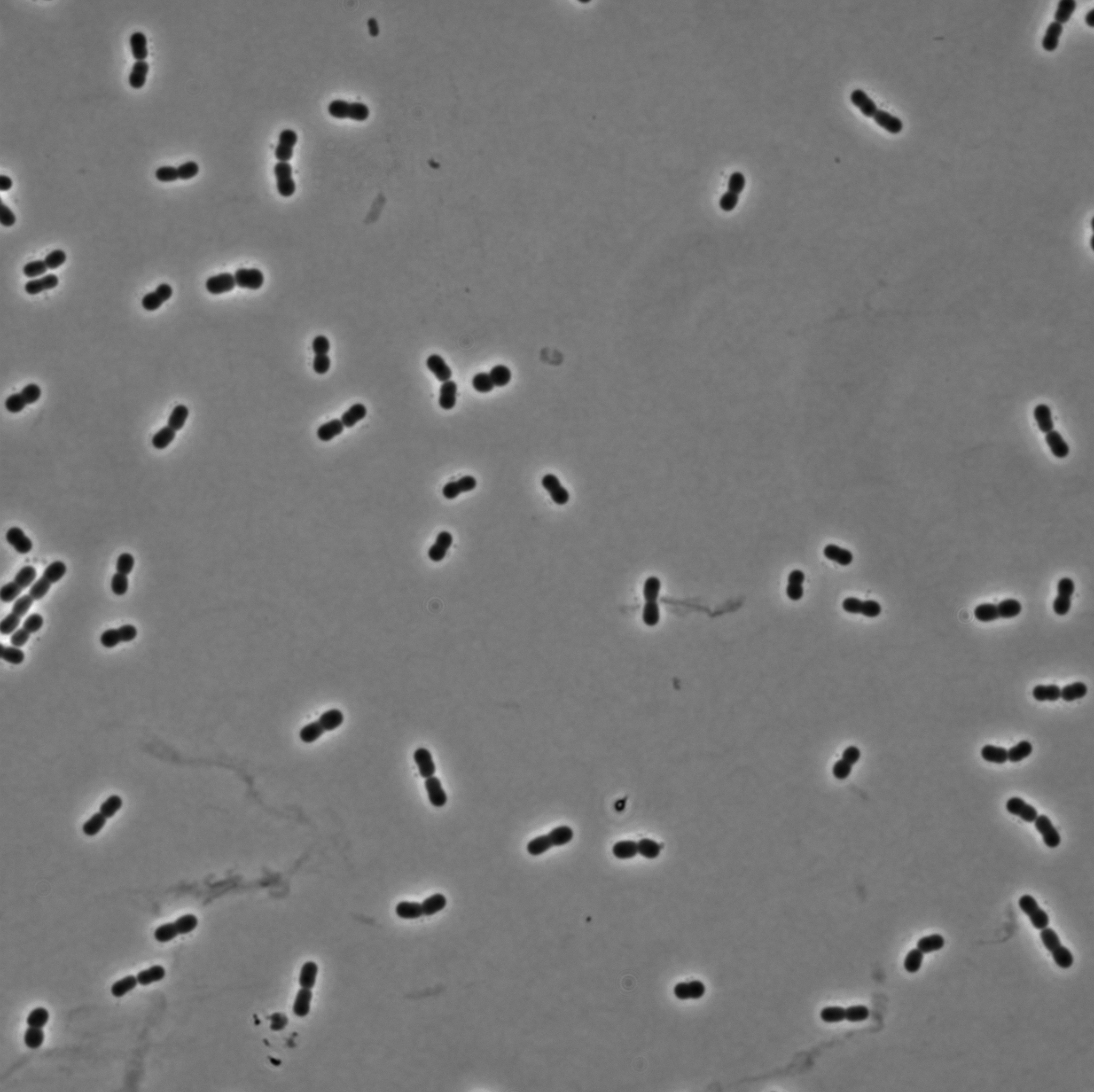

Supplement: Supplementary file 18 — Source data Fig. 6 [file 44321_2025_219_MOESM18_ESM.zip › Figure 6/6A/AB5075 Mu 2 percent saccarine014_RGB_Brightfield.tif]

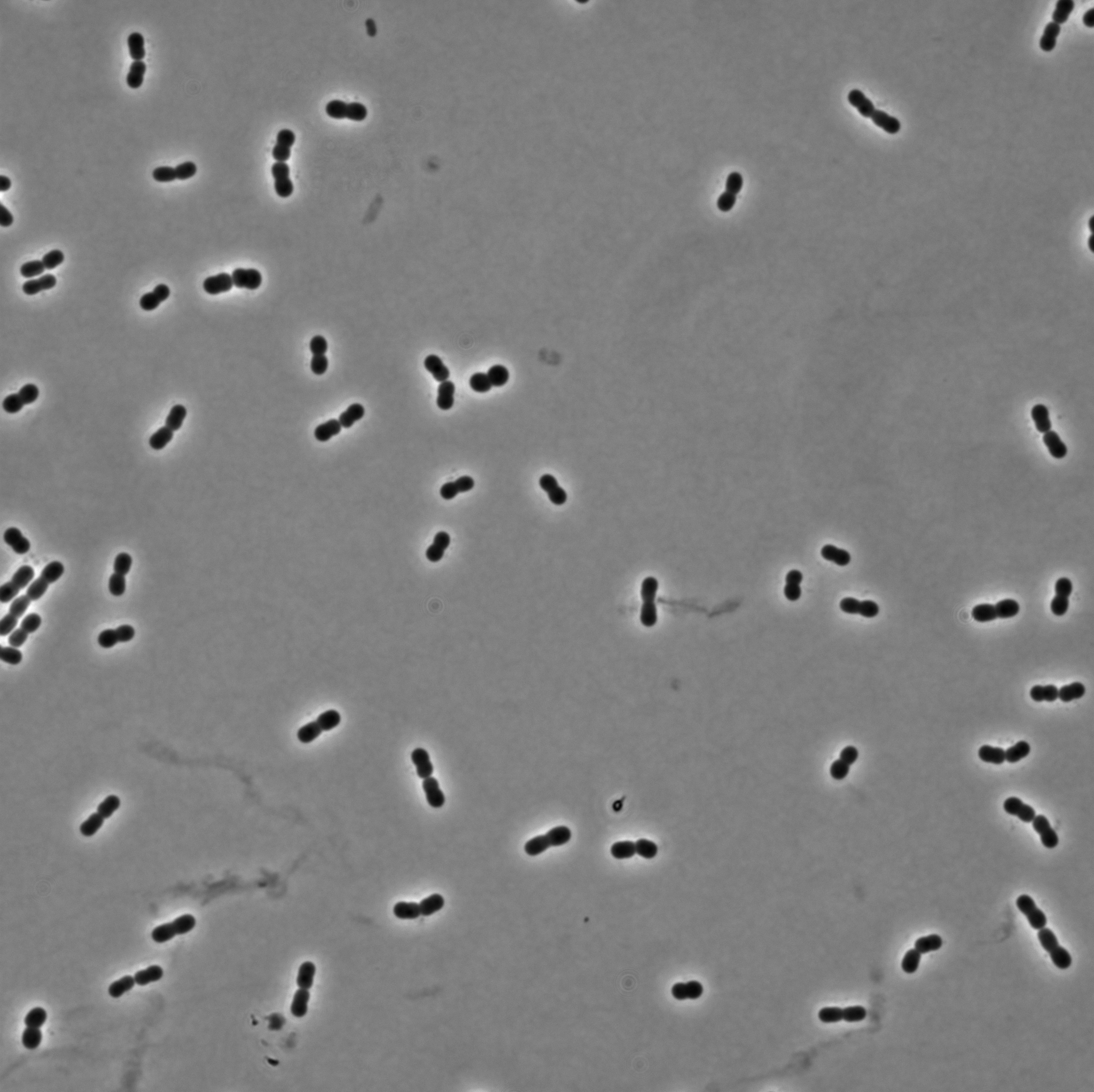

Supplement: Supplementary file 18 — Source data Fig. 6 [file 44321_2025_219_MOESM18_ESM.zip › Figure 6/6A/AB5075 Mu 2 percent saccarine016_RGB_Brightfield.tif]

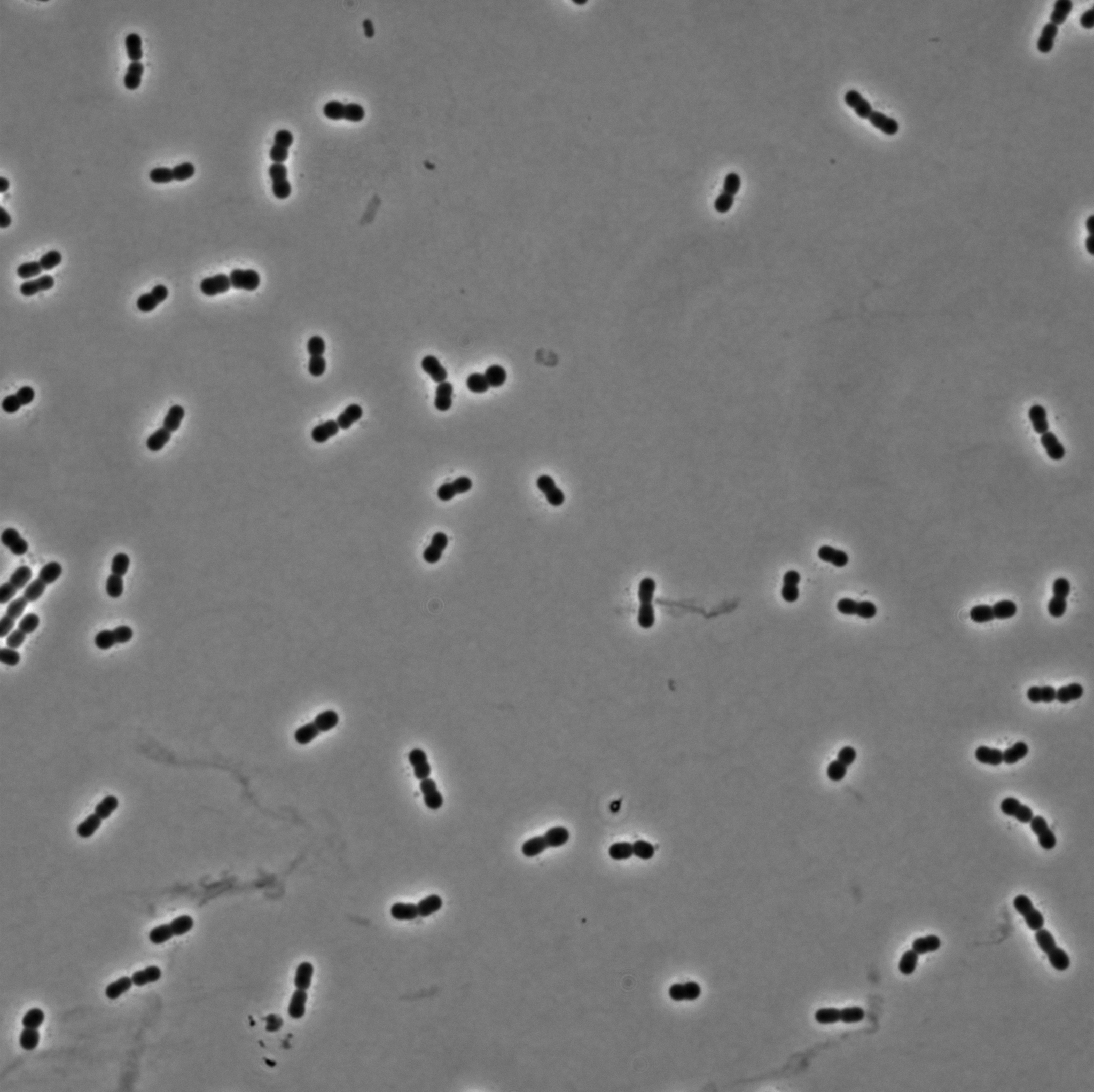

Supplement: Supplementary file 18 — Source data Fig. 6 [file 44321_2025_219_MOESM18_ESM.zip › Figure 6/6A/AB5075 Mu 2 percent saccarine018_RGB_Brightfield.tif]

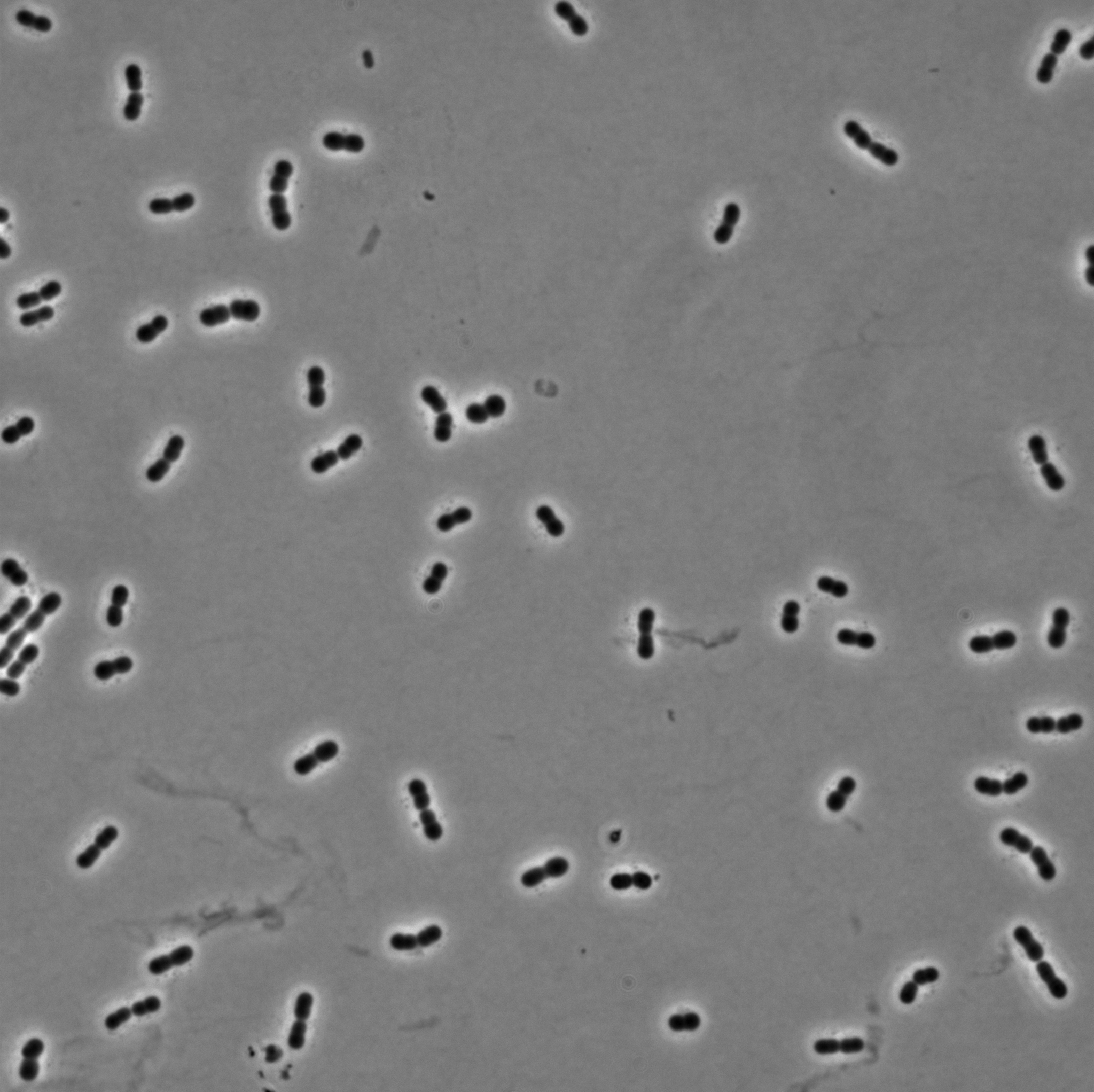

Supplement: Supplementary file 18 — Source data Fig. 6 [file 44321_2025_219_MOESM18_ESM.zip › Figure 6/6A/AB5075 Mu 2 percent saccarine020_RGB_Brightfield.tif]

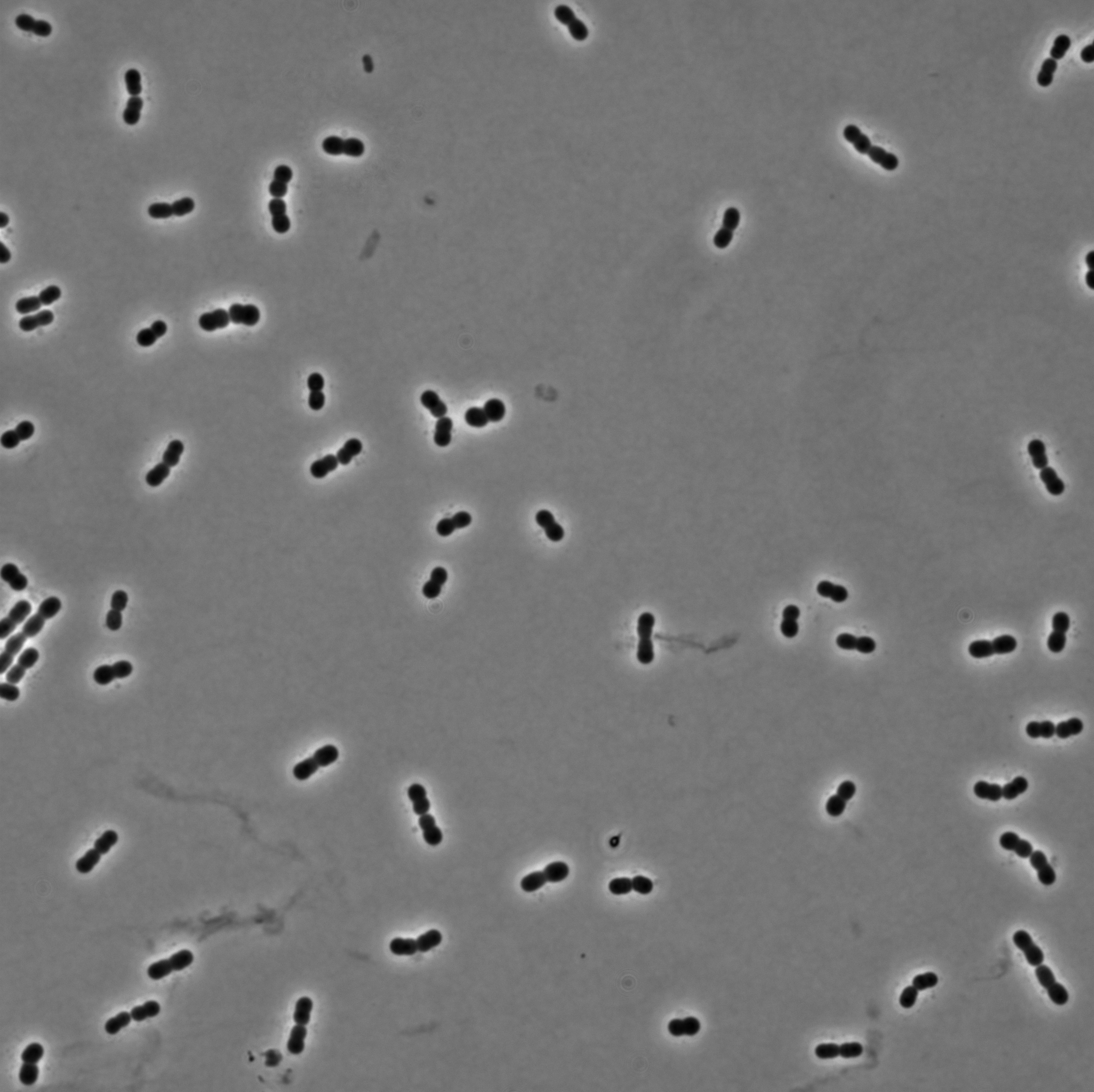

Supplement: Supplementary file 18 — Source data Fig. 6 [file 44321_2025_219_MOESM18_ESM.zip › Figure 6/6A/AB5075 Mu 2 percent saccarine022_RGB_Brightfield.tif]

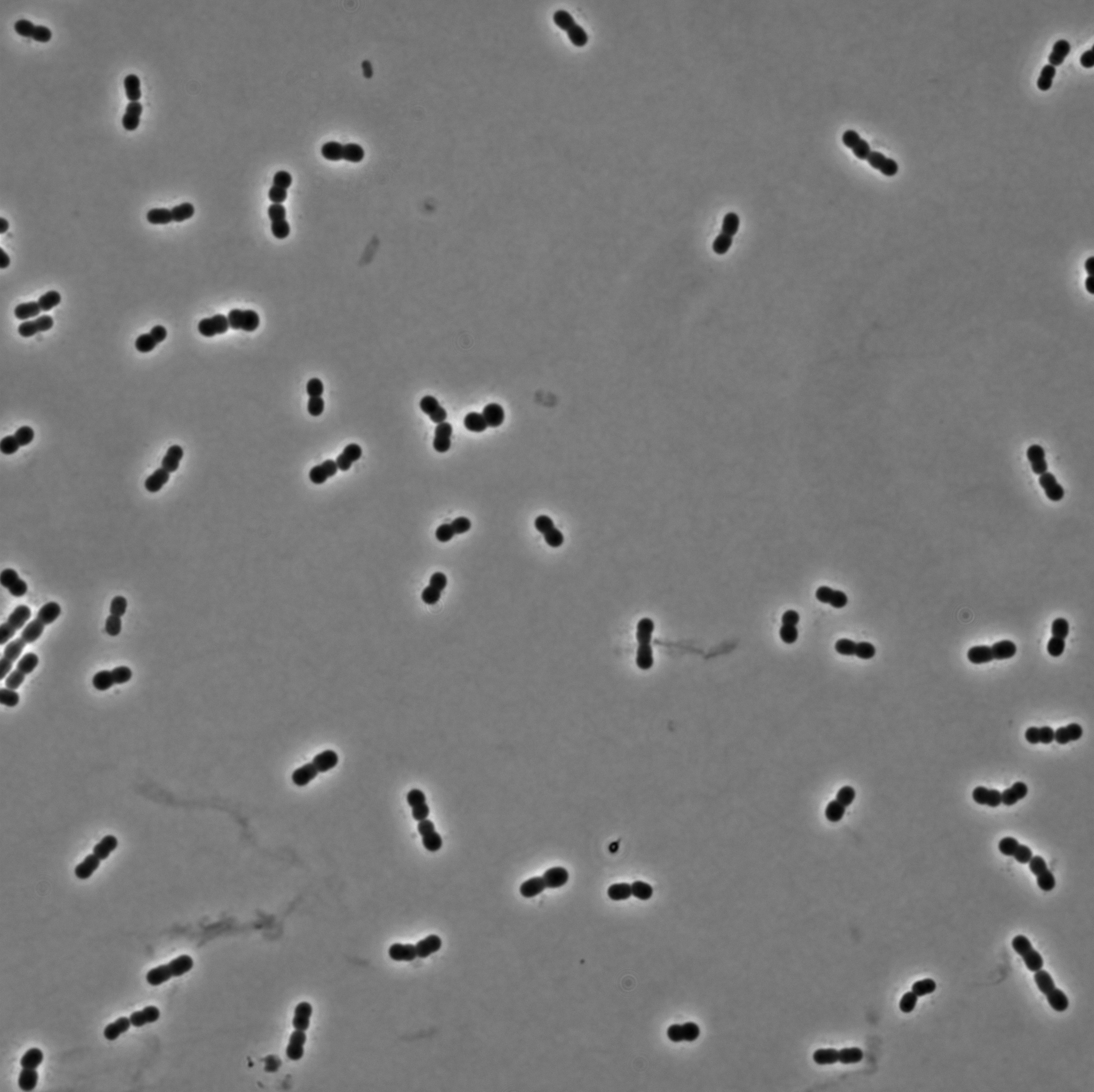

Supplement: Supplementary file 18 — Source data Fig. 6 [file 44321_2025_219_MOESM18_ESM.zip › Figure 6/6A/AB5075 Mu 2 percent saccarine024_RGB_Brightfield.tif]
